# Supplementary material for: Selective ring expansion and C−H functionalization of azulenes
Source: Nat Commun. 2023 Dec 1;14:7936. doi: 10.1038/s41467-023-43200-7 (PMC10692195; doi:10.1038/s41467-023-43200-7)

Supplementary Information

Selective Ring Expansion and C−H Functionalization of Azulenes

Sangjune Park[1,2], Cheol-Eui Kim[1,2], Jinhoon Jeong[3,4], Ho Ryu[3,4], Chanyoung Maeng[1,2], Dongwook Kim[3,4], Mu-Hyun Baik[3,4*], and Phil Ho Lee[1,2,5*]

[1]Department of Chemistry, Kangwon National University, Chuncheon 24341, Republic of Korea

[2]National Creative Research Initiative Center for Catalytic Organic Reactions, Chuncheon 24341, Republic of Korea

[3]Department of Chemistry, Korea Advanced Institute of Science and Technology (KAIST), Daejeon 34141, Republic of Korea

[4]Center for Catalytic Hydrocarbon Functionalizations, Institute for Basic Science (IBS), Daejeon 34141, Republic of Korea

[5]Institute for Molecular Science and Fusion Technology, Kangwon National University, Chuncheon, 24341, Republic of Korea

*Corresponding authors. E-mail: mbaik2805@kaist.ac.kr and phlee@kangwon.ac.kr

Table of Contents

General Information ……………………………………………………………….......... S3

Experimental Section

A. Substrate preparation ……………………………………..………………....... S4

B. Ring expansion of azulenes with alkyl and aryl diazo esters …........... S8

C. C-H Functionalization of azulenes with alkyl and aryl diazoesters S27

D. X-ray crystallographic data of 3o, 3y, and 4g …………………………..... S43

Computational Section

A. Mechanistic details…...…………………………………………….............… S82

B. Energy components……………………………………………….............… S84

C. XYZ coordinates…………………………………………………….............… S85

D. Vibrational frequencies…………………………………………….............… S100

^{1}H and ^{13}C NMR spectra…………………………………………..................…. S107

References ……………………………………………………………….............… S108

General Information

Reactions were carried out in oven-dried glassware under nitrogen atmosphere. $Cu(hfacac)_2$ and AgOTf were purchased and was used as received. Commercial available reagents were used without purification. 1,4-Dioxane, DCE, DCM, EtOAc, hexane, and toluene were dried with CaH_2. All reaction mixtures were stirred magnetically and were monitored by thin-layer chromatography using silica gel pre-coated glass plates, which were visualized with UV light and then developed using either iodine or a solution of anisaldehyde. Flash column chromatography was carried out using silica gel (230-400 mesh). ^1H NMR (400 MHz) and $^{13}C\{^1H\}$ NMR (100 MHz) spectra were recorded on NMR spectrometer. Deuterated chloroform and benzene were used as the solvents, and chemical shift values (δ) are reported in parts per million relative to the residual signals of these solvent [δ 7.26 for ^1H (chloroform-*d*), δ 7.16 for ^1H (benzene-d_6), δ 77.2 for $^{13}C\{^1H\}$ (chloroform-*d*) and δ 128.1 for $^{13}C\{^1H\}$ (benzene-d_6). Infrared spectra were recorded on FT-IR spectrometer as either a thin neat pressed between two sodium chloride plates or as a solid suspended in a potassium bromide disk. High resolution mass spectra (HRMS) were obtained by fast atom bombardment (FAB) using a double focusing magnetic sector mass spectrometer and electron impact (EI) ionization technique (magnetic sector – electric sector double focusing mass analyzer) from the KBSI (Korea Basic Science Institute Daegu Center). Melting points were determined in open capillar tube.

Experimental Section

A. Substrate preparation[1]

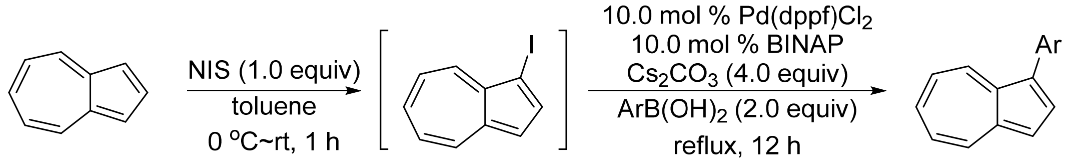

General procedure for the preparation of azulene derivatives: Azulene (256.0 mg, 2.0 mmol) and toluene (5.0 mL) were added to a 25 mL round-bottom flask with a stirring bar. The mixture was stirred at 0 °C for 10 min. After *N*-iodosuccinimide (450.0 mg, 2.0 mmol) was added in 5-portion to the mixture and then, the mixture was stirred at room temperature for 1 h. Completion of the reaction was indicated by the TLC. The residue was passed through a pad of Cellite to remove succinimide and eluted with toluene (5.0 mL) to a 25 mL round-bottom flask with a stirring bar, arylboronic acid (4.0 mmol), $Pd(dppf)Cl_2$ (14.6 mg, 0.02 mmol), BINAP (12.5 mg, 0.02 mmol), and Cs_2CO_3 (2.61 g, 8.0 mmol). The mixture was stirred at reflux for 12 h. The mixture was extracted with dichloromethane and was washed with H_2O. The solution was removed under reduced pressure and the residue was purified by column chromatography using hexane to give **1t-1v**.

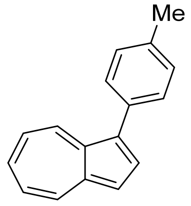

1t

1-(*p*-Tolyl)azulene (1t)

Blue solid; m.p. 38−40 °C; R_f = 0.3 (hexane); ^{1}H NMR (400 MHz, C_6D_6) δ 8.51 (d, J = 9.8 Hz, 1H), 8.02 (d, J = 3.9 Hz, 1H), 7.98 (d, J = 9.4 Hz, 1H), 7.51 (dd, J = 6.4 Hz, 1.6 Hz, 2H), 7.35 (d, J = 3.9 Hz, 1H), 7.17−7.13 (m, 3H), 6.72 (t, J = 9.8 Hz, 2H), 2.22 (s, 3H); $^{13}C\{^1H\}$ NMR (100 MHz, C_6D_6) δ 142.1, 137.9, 137.5, 137.2, 135.8, 135.7, 135.1, 132.0, 130.0, 129.6, 123.0, 122.8, 118.0, 21.1; IR (neat) 3023, 2917, 1569, 1524, 1493, 1392, 821 cm^{-1}; HRMS (EI) *m/z*: [M$^+$] Calcd for $C_{17}H_{14}$ 218.1096; Found 218.1094.

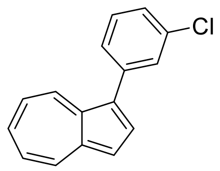

1u

1-(3-Chlorophenyl)azulene (1u)

Blue oil; R_f = 0.3 (EtOAc:hexane = 1:50) ^{1}H NMR (400 MHz, C_6D_6) δ 8.30 (d, J = 9.8 Hz, 1H), 7.93 (d, J = 9.4 Hz, 1H), 7.79 (d, J = 3.9 Hz, 1H), 7.60 (t, J = 1.8 Hz, 1H), 7.27−7.24 (m, 2H), 6.96 (t, J = 7.8 Hz, 1H), 6.71 (t, J = 9.7 Hz, 1H), 6.65 (t, J = 9.8 Hz, 1H); ^{13}C{^{1}H} NMR (100 MHz, C_6D_6) δ 142.2, 139.9, 138.0, 137.44, 137.41, 135.8, 135.4, 134.8, 130.02, 130.00, 129.9, 126.3, 123.7, 123.3, 118.0; IR (neat) 3060, 3024, 1592, 1575, 1561, 1475, 1394, 778 cm^{-1}; HRMS (EI) *m/z*: [M$^+$] Calcd for $C_{16}H_{11}Cl$ 238.0549; Found 238.0546.

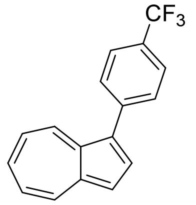

1v

1-(4-(Trifluoromethyl)phenyl)azulene (1v)

Blue solid; m.p. 45-47 °C; R_f = 0.3 (EtOAc:hexane = 1:50); ^{1}H NMR (400 MHz, $CDCl_3$) δ 8.53 (d, J = 9.8 Hz, 1H), 8.39 (d, J = 9.5 Hz, 1H), 8.02 (d, J = 3.9 Hz, 1H), 7.75−7.70 (m, 4H), 7.64 (t, J = 9.8 Hz, 1H), 7.45 (d, J = 3.9 Hz, 1H), 7.212 (t, J = 9.7 Hz, 1H), 7.206 (t, J = 9.8 Hz, 1H); ^{13}C{^{1}H} NMR (100 MHz, $CDCl_3$) δ 142.2, 141.3, 138.7, 137.8, 137.3, 135.6, 135.5, 129.9, 129.6, 128.2 (q, J = 32.3 Hz), 125.7 (q, J = 3.8 Hz), 124.7 (q, J = 271.7 Hz), 124.1, 123.9, 117.9; IR (neat) 2967, 1614, 1570, 1429, 1362, 1324, 1163, 1120, 1065, 1015, 845, 741 cm^{-1}; HRMS (EI) *m/z*: [M$^+$] Calcd for $C_{17}H_{11}F_3$ 272.0813; Found 272.0811.

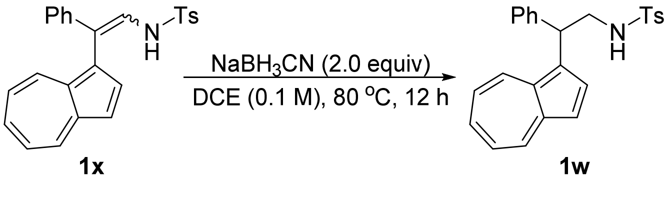

***N*-(2-(Azulen-1-yl)-2-phenylethyl)-4-methylbenzenesulfonamide (1w)**

A mixture of *N*-(2-(azulen-1-yl)-2-phenylvinyl)-tolylsulfonamide (**1x**)[2-3] (79.9 mg, 0.2 mmol) and $NaBH_3CN$ (25.1 mg, 0.4 mmol) in dichloroethane (2.0 mL) was stirred at 80 °C for 12 h under a nitrogen atmosphere. The resulting mixture was diluted with CH_2Cl_2 after cooling to room temperature and filtered through a pad of Celite. The filtrate was concentrated under reduced pressure, and the residue was purified via silica gel flash column chromatography to give the *N*-(2-(azulen-1-yl)-2-phenylethyl)-4-methyl-benzenesulfonamide (45.8 mg, 57%). R_f= 0.2 (EtOAc:hexane = 1:5); Blue solid, m.p. 57-61 °C; ^{1}H NMR (400 MHz, $CDCl_3$) δ 8.27 (d, J = 9.4 Hz, 1H), 8.11 (d, J = 9.7 Hz, 1H), 7.67 (d, J = 3.9 Hz, 1H), 7.66-7.63 (m, 2H), 7.56 (t, J = 9.9 Hz, 1H), 7.32 (d, J = 3.9 Hz, 1H), 7.28-7.26 (m, 2H), 7.25-7.21 (m, 2H), 7.18-7.10 (m, 4H), 7.07 (t, J = 10.0 Hz, 1H), 4.72 (t, J = 7.9 Hz, 1H), 4.40 (t, J = 6.2 Hz, 1H), 3.75-3.60 (m, 2H), 2.44 (s, 3H); ^{13}C NMR (100 MHz, $CDCl_3$) δ 143.6, 142.0, 141.0, 138.2, 137.3, 137.0, 136.3, 134.7, 133.6, 129.9, 128.9, 127.9, 127.3, 127.0, 123.2, 122.7, 117.4, 48.4, 43.4, 21.7; IR (film) : 3289, 3026, 1576, 1396, 1328, 1160, 1092, 739 cm^{-1}; HRMS (EI) *m/z*: [M$^+$] Calcd for $C_{25}H_{23}NO_2S$: 401.1449; Found 401.1448.

Preparation of diazo compound for intramolecular ring expansion reaction

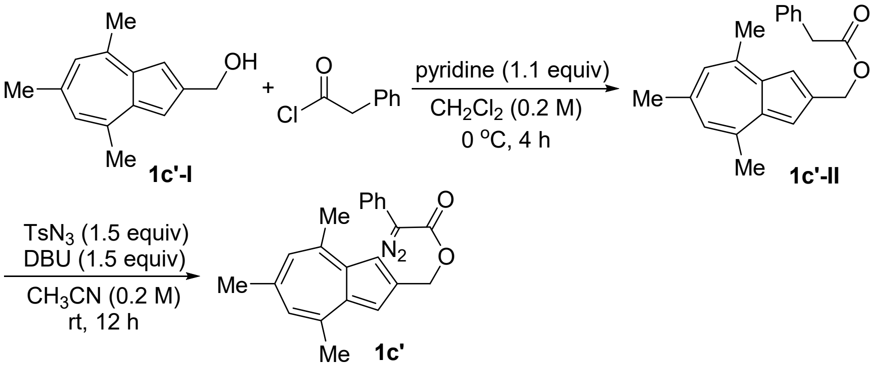

(4,6,8-Trimethylazulen-2-yl)methyl 2-phenylacetate (1c'-II)

4,6,8-Trimethylazulene-2-methanol (**1c'-I**)[4] (332.2 mg, 1.7 mmol) and dichloromethane (8.3 mL) were added to a 25 mL round-bottom flask equipped with a stirring bar. After phenylacetyl chloride (247.3 μL, 1.9 mmol) and pyridine (153.7 μL, 1.9 mmol) were added to the mixture at 0 °C for 10 min. The mixture was stirred at 0 °C for 4 h. Completion of the reaction was indicated by the TLC. The mixture was extracted with dichloromethane and was washed with H_2O. The organic layer was dried over $MgSO_4$

and concentrated *in vacuo*. The filtrate was concentrated under reduced pressure and the residue was purified by Silica gel fresh column chromatography (EtOAc:hexane) to give **1c'-II** (382.0 mg, 72%) as violet oil. R_f = 0.2 (EtOAc:hexane = 1:3); ^{1}H NMR (400 MHz, $CDCl_3$) δ 7.36−7.27 (m, 5H), 7.20 (s, 2H), 7.07 (s, 2H), 5.49 (s, 2H), 3.73 (s, 2H), 2.82 (s, 6H), 2.62 (s, 3H); $^{13}C\{^1H\}$ NMR (100 MHz, $CDCl_3$) δ 171.6, 146.4, 145.8, 141.6, 136.2, 134.2, 129.4, 128.6, 127.7, 127.1, 114.5, 63.3, 41.5, 28.8, 25.1; IR (neat) 3087, 3060, 3030, 2999, 2947, 2918, 1736, 1577, 1453, 1332, 1241, 1146 cm^{-1}; HRMS (EI) Calcd for *m/z*: [M$^+$] $C_{22}H_{22}O_2$ 318.1620; Found 316.1621.

(4,6,8-Trimethylazulen-2-yl)methyl 2-diazo-2-phenylacetate (1c')

(4,6,8-Trimethylazulen-2-yl)methyl 2-phenylacetate **1c'-II** (382.0 mg, 1.2 mmol) and acetonitrile (6.0 mL) were added to a 25 mL round-bottom flask equipped with a stirring bar. After tosyl azide (236.7 mg, 1.2 mmol) and DBU (269.2 μL, 1.8 mmol) were added to the mixture at 0 ^{o}C, the mixture was stirred at room temperature for 12 h. Completion of the reaction was indicated by the TLC. The mixture was extracted with dichloromethane and was washed with H_2O. The organic layer was dried over $MgSO_4$ and concentrated *in vacuo*. The filtrate was concentrated under reduced pressure and the residue was purified by Silica gel fresh column chromatography to give **1c'** (70.3 mg, 17%) as violet solid. m.p. 80-82 ^{o}C; R_f = 0.3 (EtOAc:hexane = 1:20); ^{1}H NMR (400 MHz, $CDCl_3$) δ 7.53 (d, J = 8.1 Hz, 2H), 7.39 (t, J = 7.6 Hz, 2H), 7.30 (s, 2H), 7.18 (t, J = 7.0 Hz, 1H), 7.08 (s, 2H), 5.67 (s, 2H), 2.86 (s, 6H), 2.62 (s, 3H); $^{13}C\{^1H\}$ NMR (100 MHz, $CDCl_3$) δ 165.3, 146.5, 146.0, 141.5, 136.3, 128.9, 127.8, 125.8, 125.7, 124.1, 114.6, 63.3, 28.8, 25.1; IR (neat) 3058, 3024, 3000, 2951, 2920, 2086, 1703, 1577, 1498, 1448, 1330, 1243, 1149, 1014 cm^{-1}; HRMS (EI) *m/z*: [M$^+$] Calcd for $C_{22}H_{20}N_2O_2$ 344.1525; Found 344.1525.

B. Ring expansion of azulenes with alkyl and aryl diazo esters

Screening of reactions conditions

Screening experiments for optimization were carried out using azulene (**1a**) and methyl 2-diazo-2-phenylacetate (**2a**). The conditions for each trial are specified in the footnote. All yields were determined by analysis of the crude ^{1}H NMR spectrum using CH_2Br_2 as the internal standard after filtration of the reaction mixture through a pad of silica gel unless otherwise noted.

Supplementary Table 1. Examination of Dirhodium Catalyst and Solvent in the Reaction of Azulene with Methyl Diazo Phenylacetate[a]

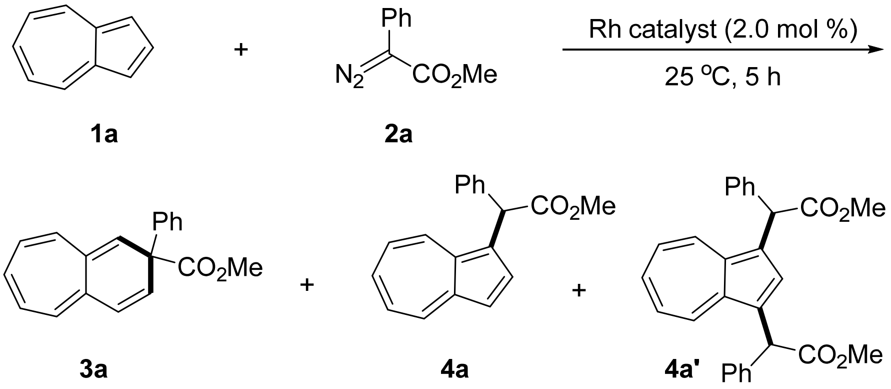

Entry	Catalyst (2.0 mol %)	Solvent	Yield (%)[b] 3a	4a	4a'
1	$Rh_2(OAc)_4$	DCE	16	40	0
2	$Rh_2(Oct)_4$	DCE	15	22	0
3	$Rh_2(TFA)_4$	DCE	0	25	6
4	$Rh_2(esp)_2$	DCE	0	9	0
5	$Rh_2(OAc)_4$	DCM	29	32	0
6	$Rh_2(OAc)_4$	MeCN	30	7	0
7	$Rh_2(OAc)_4$	toluene	21	33	2
8	$Rh_2(OAc)_4$	hexane	17	10	3
9	$Rh_2(OAc)_4$	THF	14	11	0

[a]**1a** (0.2 mmol, 1.0 equiv), **2a** (1.0 equiv), and Rh catalyst (2.0 mol %) were used in solvent (0.05 M) at 25 °C for 5 h under a N_2 atmosphere. [b]NMR yield with CH_2Br_2 as an internal standard.

Supplementary Table 2. Examination of Diazo Compounds in Reaction with Azulene in the Presence of $Cu(hfacac)_2$[a]

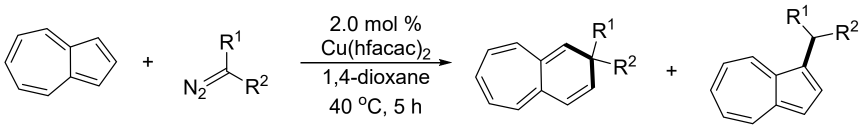

Entry	Diazo Compounds	Products
1	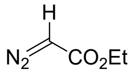	0
2	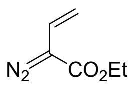	0
3	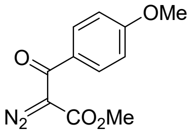	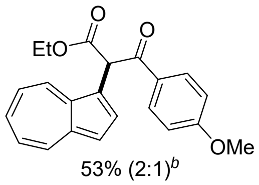 53% (2:1)[b]
4	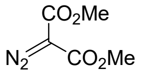	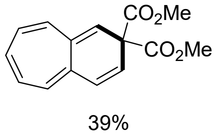 39% 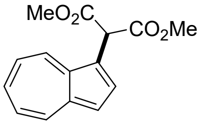 25%
5	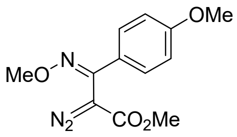	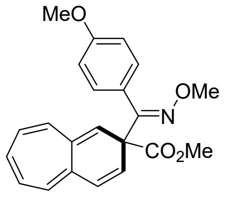 83%

[a]Azulene (0.2 mmol, 1.0 equiv) was reacted with diazo compound (1.0 equiv) in the presence of $Cu(hfacac)_2$ (2.0 mol %) in 1,4-dioxane (0.1 M) at 40 °C for 5 h under a N_2 atmosphere. [b]Ratio of keto and enol form.

Supplementary Table 3. Examination of Diazo Compounds in Reaction with Azulene in the Presence of AgOTf[a]

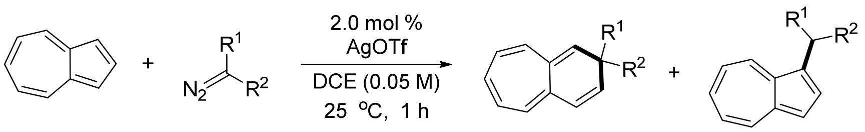

Entry	Diazo Compounds	Products
1	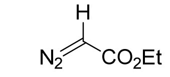	0
2	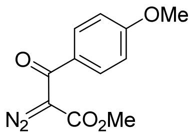	0
3[b]	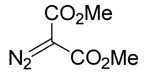	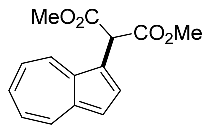 trace
4	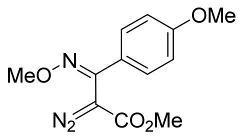	0
5	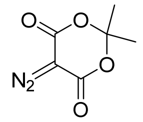	0
6	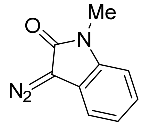	0

[a]Azulene (0.2 mmol, 1.0 equiv) was reacted with diazo compound (1.5 equiv) in the presence of AgOTf (2.0 mol %) in DCE (0.05 M) at 25 °C for 1 h under a N_2 atmosphere. [b]Reaction was carried out at 70 °C for 12 h.

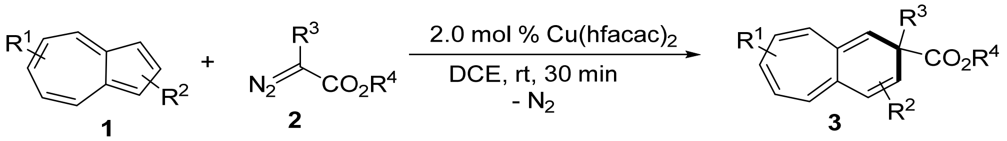

Cu(hfacac)$_2$ (1.9 mg, 2.0 mol %), azulene derivatives (0.2 mmol) and DCE (3.0 mL) were added to a test tube equipped with a stirring bar. To the solution was added aryl diazoacetate derivatives (0.2 mmol) in DCE (1.0 mL). The mixture was stirred at room temperature for 30 min. The residue was passed through

a pad of Cellite to remove Cu(hfacac)$_2$ and eluted with CH_2Cl_2. The filtrate was concentrated under reduced pressure and the residue was purified by Silica gel fresh column chromatography (EtOAc:hexane) to give **3**.

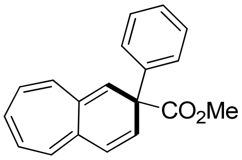

3a

Methyl 2-phenyl-2*H*-benzo[7]annulene-2-carboxylate (3a)

Yield (51.4 mg, 93%); Red solid; m.p. 99−101 °C; R_f = 0.7 (EtOAc:hexane = 1:10); ^{1}H NMR (400 MHz, C_6D_6) δ 7.37−7.32 (m, 2H), 7.24−7.18 (m, 3H), 6.10 (d, J = 9.8 Hz, 1H), 6.05−6.00 (m, 2H), 5.66−5.62 (m, 2H), 5.58−5.56 (m, 1H), 5.53−5.48 (m, 1H), 5.44 (s, 1H), 3.75 (s, 3H); $^{13}C\{^1H\}$ NMR (100 MHz, $CDCl_3$) δ 173.0, 145.0, 139.7, 136.7, 136.3, 130.8, 130.0, 129.5, 129.3, 128.8, 126.8, 126.6, 126.4, 121.9, 56.5, 52.6; IR (neat) 3025, 2950, 1730, 1431, 1217 cm^{-1}; HRMS (FAB) *m/z*: $[M + H]^+$ Calcd for $C_{19}H_{17}O_2$ 277.1229; Found 277.1227.

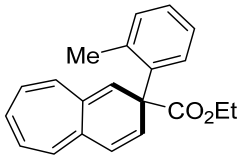

3b

Ethyl 2-(*o*-tolyl)-2*H*-benzo[7]annulene-2-carboxylate (3b)

The compound **3b** was prepared according to the same procedure as that of the synthesis of **3a**. The crude residue was purified by Silica gel column chromatography to give **3b** (50.3 mg, 82%) as a red solid. m.p. 114−116 °C; R_f = 0.5 (EtOAc:hexane = 1:10); ^{1}H NMR (400 MHz, C_6D_6) δ 7.39 (d, J = 7.6 Hz, 1H), 7.11−7.07 (m, 1H), 7.00−6.98 (m, 2H), 6.00 (ddd, J = 9.7 Hz, 2.2 Hz, 0.5 Hz, 1H), 5.84 (d, J = 8.9 Hz, 1H), 5.78 (d, J = 12.2 Hz, 1H), 5.47 (d, J = 0.5 Hz, 1H), 5.36−5.29 (m, 2H), 5.20−5.14 (m, 2H), 3.94 (qd, J = 7.1 Hz, 1.2 Hz, 2H), 2.28 (s, 3H), 0.86 (t, J = 7.1 Hz, 3H); $^{13}C\{^1H\}$ NMR (100 MHz, C_6D_6) δ 172.2, 143.4, 139.9, 137.9, 137.0, 134.7, 132.2, 130.9, 130.72, 130.70, 130.2, 129.7, 129.3, 126.9, 126.7, 126.6, 122.6, 61.2, 57.6, 20.3, 13.8; IR (neat) 3024, 2979, 1726, 1578, 1458, 1210, 1029, 859 cm^{-1}; HRMS (EI) *m/z*: $[M^+]$ Calcd $C_{21}H_{20}O_2$ 304.1463; Found 304.1465.

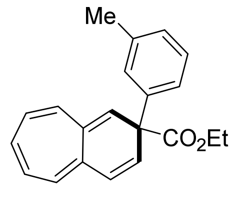

3c

Ethyl 2-(*m*-tolyl)-2*H*-benzo[7]annulene-2-carboxylate (3c)

The compound **3c** was prepared according to the same procedure as that of the synthesis of **3a**. The crude residue was purified by Silica gel column chromatography to give **3c** (46.2 mg, 76%) as a red oil. R_f = 0.7 (EtOAc:hexane = 1:10); ^{1}H NMR (400 MHz, C_6D_6) δ 7.27 (s, 1H), 7.22 (d, J = 8.0 Hz, 1H), 7.10 (t, J = 7.6 Hz, 1H), 6.84 (d, J = 7.5 Hz, 1H), 6.25 (dd, J = 9.7 Hz, 2.0 Hz, 1H), 5.88 (d, J = 10.0 Hz, 1H), 5.85 (d, J = 12.7 Hz, 1H), 5.74 (s, 1H), 5.36−5.30 (m, 2H), 5.23−5.14 (m, 2H), 3.99−3.91 (m, 2H), 2.05 (s, 3H), 0.86 (t, J = 7.1 Hz, 3H); ^{13}C{^{1}H} NMR (100 MHz, C_6D_6) δ 172.2, 145.8, 140.1, 138.5, 136.9, 136.8, 131.9, 130.7, 129.9, 129.5, 129.3, 128.9, 127.6, 126.5, 123.8, 123.2, 61.1, 56.8, 21.3, 13.9; IR (neat) 3024, 2980, 1727, 1445, 1213, 1033 cm^{-1}; HRMS (EI) *m/z*: [M$^+$] Calcd $C_{21}H_{20}O_2$ 304.1463; Found 304.1462.

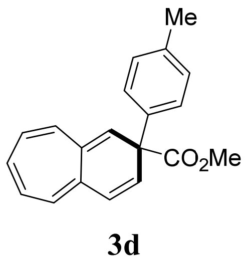

3d

Methyl 2-(*p*-tolyl)-2*H*-benzo[7]annulene-2-carboxylate (3d)

The compound **3d** was prepared according to the same procedure as that of the synthesis of **3a**. The crude residue was purified by Silica gel column chromatography to give **3d** (41.4 mg, 70%) as a red oil. R_f = 0.7 (EtOAc:hexane = 1:7); ^{1}H NMR (400 MHz, C_6D_6) δ 7.26 (d, J = 8.1 Hz, 2H), 6.98 (d, J = 8.1 Hz, 2H), 6.21 (dd, J = 9.7 Hz, 2.1 Hz, 1H), 5.85 (d, J = 9.8 Hz, 1H), 5.83 (d, J = 12.1 Hz, 1H), 5.69 (s, 1H), 5.35−5.30 (m, 2H), 5.22−5.15 (m, 2H), 3.28 (s, 3H), 2.04 (s, 3H); ^{13}C{^{1}H} NMR (100 MHz, C_6D_6) δ 172.7, 143.0, 140.0, 136.83, 136.80, 136.3, 131.9, 130.7, 129.8, 129.6, 129.5, 129.3, 126.7, 126.5, 123.1, 56.6, 51.7, 20.7; IR (neat) 3026, 2923, 1733, 1510, 1434, 1243, 1022 cm^{-1}; HRMS (FAB) *m/z*: [M + H]$^+$ Calcd $C_{20}H_{19}O_2$ 291.1385; Found 291.1381.

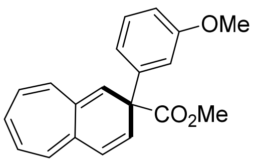

3e

Methyl 2-(3-methoxyphenyl)-2*H*-benzo[7]annulene-2-carboxylate (3e)

The compound **3e** was prepared according to the same procedure as that of the synthesis of **3a**. The crude residue was purified by Silica gel column chromatography to give **3e** (44.0 mg, 71%) as a red solid. m.p. 110−112 °C; R_f = 0.3 (EtOAc:hexane = 1:7); ^{1}H NMR (400 MHz, C_6D_6) δ 7.29−7.25 (m, 1H), 6.79−6.75 (m, 3H), 6.10 (d, J = 9.8 Hz, 1H), 6.04 (d, J = 9.1 Hz, 1H), 6.02−6.00 (m, 1H), 5.66−5.64 (m, 2H), 5.59−5.56 (m 1H), 5.53−5.48 (m, 1H), 5.43 (s, 1H), 3.80 (s, 3H), 3.75 (s, 3H); ^{13}C{^{1}H} NMR (100 MHz, $CDCl_3$) δ 172.9, 159.9, 146.5, 139.7, 136.7, 136.3, 130.8, 130.6, 130.1, 129.8, 129.44, 129.40, 126.6, 121.7, 118.6, 113.0, 111.7, 56.4, 55.3, 52.6; IR (neat) 3027, 2979, 2933, 2899, 2869, 1727, 1487, 1212 cm^{-1}; HRMS (FAB) *m/z*: [M + H]$^{+}$ Calcd for $C_{20}H_{19}O_3$ 307.1334; Found 307.1335.

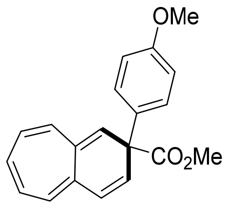

3f

Methyl 2-(4-methoxyphenyl)-2*H*-benzo[7]annulene-2-carboxylate (3f)

The compound **3f** was prepared according to the same procedure as that of the synthesis of **3a**. The crude residue was purified by Silica gel column chromatography to give **3f** (45.2 mg, 73%) as a red solid. m.p. 115−117 °C; R_f = 0.2 (EtOAc:hexane = 1:20); ^{1}H NMR (400 MHz, C_6D_6) δ 7.25−7.21 (m, 2H), 6.76−6.73 (m, 2H), 6.20 (ddd, J = 9.8 Hz, 2.2 Hz, 0.5 Hz, 1H), 5.87−5.84 (m, 2H), 5.68 (d, J = 0.6 Hz, 1H), 5.39−5.31 (m, 2H), 5.24−5.16 (m, 2H), 3.30 (s, 3H), 3.25 (s, 3H); ^{13}C{^{1}H} NMR (100 MHz, $CDCl_3$) δ 172.7, 158.8, 140.0, 138.1, 136.8, 136.6, 132.0, 130.7, 129.6, 129.5, 129.3, 126.5, 123.2, 114.4, 56.1, 54.7, 51.8; IR (neat) 3003, 2952, 2837, 1731, 1607, 1509, 1252, 1182, 1033 cm^{-1}; HRMS (EI) *m/z*: [M^{+}] Calcd for $C_{20}H_{18}O_3$ 306.1256; Found 306.1253.

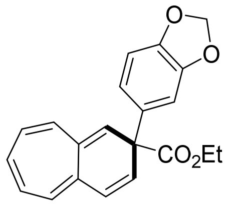

3g

Ethyl 2-(benzo[d][1,3]dioxol-5-yl)-2*H*-benzo[7]annulene-2-carboxylate (3g)

The compound **3g** was prepared according to the same procedure as that of the synthesis of **3a**. The crude residue was purified by Silica gel column chromatography to give **3g** (60.1 mg, 91%) as a red solid. m.p. 117−119 °C; R_f = 0.3 (EtOAc:hexane = 1:10); ^{1}H NMR (400 MHz, C_6D_6) δ 7.01 (d, J = 1.9 Hz, 1H), 6.78 (d, J = 1.9 Hz, 1H), 6.76 (d, J = 1.9 Hz, 1H), 6.62 (d, J = 8.1 Hz, 1H), 6.18 (ddd, J = 9.8 Hz, 2.2 Hz, 0.5 Hz, 1H), 5.85−5.81 (m, 2H), 5.67 (d, J = 0.6 Hz, 1H), 5.37−5.30 (m, 2H), 5.24 (s, 2H), 5.23−5.15 (m, 2H), 3.94−3.86 (m, 2H), 0.84 (t, J = 7.1 Hz, 3H); ^{13}C{^{1}H} NMR (100 MHz, $CDCl_3$) δ 172.1, 148.5, 146.7, 140.1, 140.0, 136.72, 136.70, 131.7, 130.7, 129.8, 129.5, 129.4, 126.5, 123.0, 120.3, 108.5, 107.5, 101.0, 61.1, 56.3, 13.8; IR (neat) 2981, 2899, 1726, 1504, 1487, 1240, 1038 cm^{-1}; HRMS (EI) *m/z*: [M$^+$] Calcd for $C_{21}H_{18}O_4$ 334.1205; Found 334.1203.

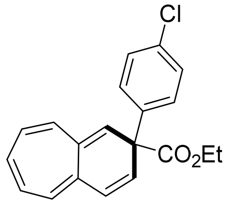

3h

Ethyl 2-(4-chlorophenyl)-2*H*-benzo[7]annulene-2-carboxylate (3h)

The compound **3h** was prepared according to the same procedure as that of the synthesis of **3a**. The crude residue was purified by Silica gel column chromatography to give **3h** (64.5 mg, 99%) as a red solid. m.p. 87−89 °C; R_f = 0.6 (EtOAc:hexane = 1:10); ^{1}H NMR (400 MHz, C_6D_6) δ 7.11−7.06 (m, 4H), 6.06 (ddd, J = 9.8 Hz, 2.2 Hz, 0.6 Hz, 1H), 5.84−5.81 (m, 2H), 5.56 (d, J = 0.6 Hz, 1H), 5.38−5.31 (m, 2H), 5.23−5.17 (m, 2H), 3.95−3.83 (m, 2H), 0.83 (t, J = 7.1 Hz, 3H); ^{13}C{^{1}H} NMR (100 MHz, $CDCl_3$) δ 171.7, 144.1, 139.9, 137.1, 136.5, 132.8, 131.1, 130.9, 130.2, 129.7, 129.5, 129.0, 128.3, 126.8, 122.2, 61.2, 56.3, 13.8; IR (neat) 3027, 2980, 1727, 1579, 1490, 1213, 1093, 1030 cm^{-1}; HRMS (EI) *m/z*: [M$^+$] Calcd for $C_{20}H_{17}ClO_2$ 324.0917; Found 324.0914.

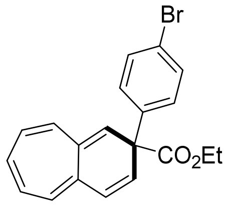

3i

Ethyl 2-(4-bromophenyl)-2*H*-benzo[7]annulene-2-carboxylate (3i)

The compound **3i** was prepared according to the same procedure as that of the synthesis of **3a**. The crude residue was purified by Silica gel column chromatography to give **3i** (65.1 mg, 91%) as a red solid. m.p. 105−107 °C; R_f = 0.7 (EtOAc:hexane = 1:7); ^{1}H NMR (400 MHz, C_6D_6) δ 7.47−7.44 (m, 2H), 7.10−7.06 (m, 2H), 6.10 (d, J = 9.8 Hz, 1H), 6.04 (d, J = 12.2 Hz, 1H), 5.97 (ddd, J = 9.7 Hz, 2.2 Hz, 6.5 Hz, 1H), 5.68−5.64 (m, 2H), 5.60−5.58 (m, 1H), 5.55−5.50 (m, 1H), 5.39 (s, 1H), 4.21 (q, J = 7.1 Hz, 2H), 1.25 (t, J = 7.1 Hz, 3H); ^{13}C{^{1}H} NMR (100 MHz, $CDCl_3$) δ 172.1, 144.1, 139.6, 136.9, 136.1, 131.8, 130.9, 130.3, 130.2, 129.6, 129.5, 128.3, 126.7, 121.3, 120.9, 61.6, 56.0, 14.0; IR (neat) 3024, 2979, 2932, 2899, 1727, 1487, 1212, 1030 cm^{-1}; HRMS (FAB) *m/z*: [M + H]$^+$ Calcd for $C_{20}H_{18}O_2Br$ 369.0490; Found 369.0488.

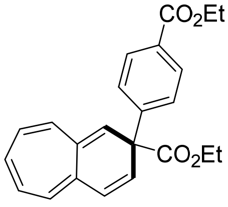

3j

Ethyl 2-(4-(ethoxycarbonyl)phenyl)-2*H*-benzo[7]annulene-2-carboxylate (3j)

The compound **3j** was prepared according to the same procedure as that of the synthesis of **3a**. The crude residue was purified by Silica gel column chromatography to give **3j** (65.3 mg, 90%) as a red solid. m.p. 108−110 °C; R_f = 0.6 (EtOAc:hexane = 1:10); ^{1}H NMR (400 MHz, C_6D_6) δ 8.15−8.13 (m, 2H), 7.34−7.32 (m, 2H), 6.10 (dd, J = 9.8 Hz, 1.7 Hz, 1H), 5.84 (d, J = 9.6 Hz, 1H), 5.82 (d, J = 11.7 Hz, 1H), 5.60 (d, J = 0.5 Hz, 1H), 5.38−5.31 (m, 2H), 5.22−5.16 (m, 2H), 4.09 (q, J = 7.1 Hz, 2H), 3.94−3.86 (m, 2H), 0.99 (t, J = 7.1 Hz, 3H), 0.83 (t, J = 7.1 Hz, 3H); ^{13}C{^{1}H} NMR (100 MHz, $CDCl_3$) δ 171.6, 165.8, 149.8, 139.9, 137.3, 136.5, 130.93, 130.90, 130.5, 130.4, 129.8, 129.54, 129.50, 126.9, 126.8, 122.0, 61.3, 60.7, 56.9, 14.1, 13.8; IR (neat) 2981, 2939, 1718, 1607, 1367, 1278, 1214, 1107, 1021 cm^{-1}; HRMS (EI) *m/z*: [M$^+$] Calcd for $C_{23}H_{22}O_4$ 362.1518; Found 362.1514.

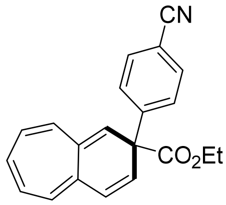

3k

Ethyl 2-(4-cyanophenyl)-2*H*-benzo[7]annulene-2-carboxylate (3k)

Cu(hfacac)$_2$ (1.9 mg, 2.0 mol %), azulene (25.6 mg, 0.2 mmol) and DCE (3.0 mL) were added to an oven-dried test tube equipped with a stirring bar. To the solution was added ethyl 2-(4-cyanophenyl)-2-diazoacetate (64.6 mg, 0.3 mmol) in DCE (1.0 mL). The mixture was stirred at room temperature for 30 min. The residue was passed through a pad of Cellite and eluted with CH_2Cl_2 to remove Cu(hfacac)$_2$. The filtrate was concentrated under reduced pressure and the residue was purified by Silica gel fresh column chromatography to give **3k** (51.5 mg, 81%) as a red oil. R_f = 0.5 (EtOAc:hexane = 1:10); ^{1}H NMR (400 MHz, C_6D_6) δ 7.05−6.99 (m, 4H), 5.92 (ddd, J = 9.8 Hz, 2.2 Hz, 0.6 Hz, 1H), 5.83 (d, J = 5.8 Hz, 1H), 5,80 (d, J = 9.6 Hz, 1H), 5.42 (d, J = 0.6 Hz, 1H), 5.36−5.34 (m, 2H), 5.24−5.19 (m, 2H), 3.90−3.84 (m, 2H); ^{13}C{^{1}H} NMR (100 MHz, $CDCl_3$) δ 171.2, 149.3, 139.6, 137.6, 136.2, 132.4, 131.2, 130.8, 130.13, 130.10, 129.6, 127.3, 127.1, 121.1, 118.5, 111.1, 61.4, 56.8, 13.8; IR (neat) 3031, 2981, 2228, 1728, 1499, 1215, 1029 cm^{-1}; HRMS (EI) *m/z*: [M$^+$] Calcd for $C_{21}H_{17}NO_2$ 315.1259; Found 315.1258.

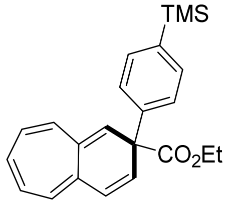

3l

Ethyl 2-(4-(trimethylsilyl)phenyl)-2*H*-benzo[7]annulene-2-carboxylate (3l)

The compound **3l** was prepared according to the same procedure as that of the synthesis of **3a**. The crude residue was purified by Silica gel column chromatography to give **3l** (70.1 mg, 97%) as a red oil. R_f = 0.5 (EtOAc:hexane = 1:10); ^{1}H NMR (400 MHz, C_6D_6) δ 7.45−7.39 (m, 4H), 6.27 (dt, J = 9.7 Hz, 1.0 Hz, 1H), 5.90 (d, J = 10.2 Hz, 1H), 5.87 (d, J = 12.8 Hz, 1H), 5.75 (s, 1H), 5.39−5.32 (m, 2H), 5.25−5.17 (m, 2H), 4.00−3.94 (m, 2H), 0.88 (t, J = 7.1 Hz, 3H), 0.19 (s, 9H); ^{13}C{^{1}H} NMR (100 MHz,

$CDCl_3$) δ 173.3, 147.5, 141.3, 139.7, 138.14, 138.11, 135.4, 133.0, 132.0, 131.3, 130.8, 130.6, 127.8, 127.5, 124.3, 62.4, 58.2, 15.2, 0.004; IR (neat) 3020, 2955, 2898, 1730, 1249, 1211, 1108, 1031, 842 cm^{-1}; HRMS (EI) *m/z*: [M^+] Calcd for $C_{23}H_{26}O_2Si$ 362.1702; Found 362.1703.

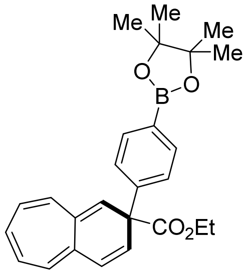

3m

Ethyl 2-(4-(4,4,5,5-tetramethyl-1,3,2-dioxaborolan-2-yl)phenyl)-2*H*-benzo[7]-annulene- 2-carboxylate (3m)

The compound **3m** was prepared according to the same procedure as that of the synthesis of **3a**. The crude residue was purified by Silica gel column chromatography to give **3m** (64.2 mg, 77%) as a red oil. R_f = 0.6 (EtOAc:hexane = 1:5); ^1H NMR (400 MHz, C_6D_6) δ 8.17 (d, J = 8.2 Hz, 2H), 7.45−7.43 (m, 2H), 6.16 (dd, J = 9.7 Hz, 1.8 Hz, 1H), 5.83 (d, J = 9.8 Hz, 1H), 5.79 (d, J = 12.2 Hz, 1H), 5.66 (s, 1H), 5.36−7.29 (m, 2H), 5.19−5.13 (m, 2H), 3.93−3.85 (m, 2H), 1.10 (s, 12H), 0.82 (t, J = 7.1 Hz, 3H); $^{13}C\{^1H\}$ NMR (100 MHz, $CDCl_3$) δ 171.9, 148.7, 140.1, 137.0, 136.8, 135.9, 135.1, 131.4, 130.7, 130.2, 129.5, 129.4, 126.5, 126.3, 122.8, 83.6, 61.1, 57.1, 24.8, 13.8; IR (neat) 2979, 1730, 1607, 1362, 1212, 1145, 1091 cm^{-1}; HRMS (EI) *m/z*: [M^+] Calcd for $C_{26}H_{29}O_4B$ 416.2159; Found 416.2159.

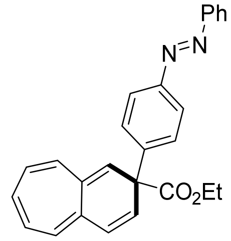

3n

(*E*)-Ethyl 2-(4-(phenyldiazenyl)phenyl)-2*H*-benzo[7]annulene-2-carboxylate (3n)

The compound **3n** was prepared according to the same procedure as that of the synthesis of **3a**. The crude residue was purified by Silica gel column chromatography to give **3n** (60.2 mg, 76%) as a red solid. m.p. 67−69 °C; R_f = 0.6 (EtOAc:hexane = 1:5); ^1H NMR (400 MHz, C_6D_6) δ 8.05−8.02 (m, 2H),

8.01−7.98 (m, 2H), 7.46−7.43 (m, 2H), 7.18−7.13 (m, 4H), 7.10−7.06 (m, 1H), 6.16 (dd, J = 9.7 Hz, 1.8 Hz, 1H), 5.87 (d, J = 9.6 Hz, 1H), 5.85 (d, J = 12.2 Hz, 1H), 5.66 (d, J = 0.4 Hz, 1H), 5.39−5.32 (m, 2H), 5.24−5.17 (m, 2H), 3.97−3.89 (m, 2H), 0.86 (t, J = 7.1 Hz, 3H); $^{13}C\{^1H\}$ NMR (100 MHz, $CDCl_3$) δ 171.8, 153.1, 151.6, 148.1, 140.0, 137.2, 136.6, 131.1, 131.0, 130.9, 130.4, 129.7, 129.6, 129.3, 129.1, 126.8, 123.7, 123.2, 122.3, 61.3, 56.9, 13.9; IR (neat) 2980, 1728, 1213, 1029 cm^{-1}; HRMS (EI) *m/z*: [M^+] Calcd for $C_{26}H_{22}N_2O_2$ 394.1681; Found 394.1682.

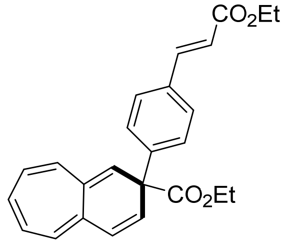

3o

(*E*)-Ethyl 2-(4-(3-ethoxy-3-oxoprop-1-en-1-yl)phenyl)-2*H*-benzo[7]annulene-2-carboxylate (3o)

The compound **3o** was prepared according to the same procedure as that of the synthesis of **3a**. The crude residue was purified by Silica gel column chromatography to give **3o** (67.1 mg, 86%) as a red solid. m.p. 138−140 °C; R_f = 0.6 (EtOAc:hexane = 1:5); ^1H NMR (400 MHz, C_6D_6) δ 7.81 (d, J = 16.0 Hz, 1H), 7.22−7.21 (m, 2H), 7.15−7.11 (m, 2H), 6.45 (d, J = 16.0 Hz, 1H), 6.12 (ddd, J = 9.8 Hz, 2.2 Hz, 0.6 Hz, 1H), 5.87−5.82 (m, 2H), 5.61 (q, J = 0.7 Hz, 1H), 5.38−5.31 (m, 2H); $^{13}C\{^1H\}$ NMR (100 MHz, $CDCl_3$) δ 171.7, 166.3, 147.2, 143.9, 139.9, 137.1, 136.6, 133.2, 131.1, 130.9, 130.3, 129.7, 129.5, 128.6, 127.3, 126.8, 122.3, 118.9, 61.2, 60.2, 56.8, 14.2, 13.8; IR (neat) 3027, 2980, 2936, 1713, 1636, 1312, 1256, 1210, 1176, 1031 cm^{-1}; HRMS (EI) *m/z*: [M^+] Calcd for $C_{25}H_{24}O_4$ 388.1675; Found 388.1671.

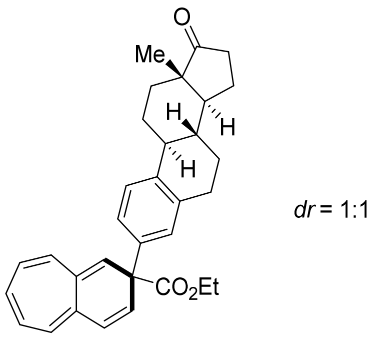

3p

Ethyl 2-((8*R*,9*S*,13*S*,14*S*)-13-methyl-17-oxo-7,8,9,11,12,13,14,15,16,17-decahydro-6*H*-cyclopenta[a]-phenanthren-3-yl)-2*H*-benzo[7]annulene-2-carboxylate (3p)

The compound **3p** was prepared according to the same procedure as that of the synthesis of **3a**. The crude residue was purified by Silica gel column chromatography to give **3p** (48.3 mg, 51%) as mixture (*dr* = 1:1) of diastereomer. Red solid. m.p. 107−109 °C; R_f = 0.3 (MeOH:$CHCl_3$ = 1:30); ^{1}H NMR (400 MHz, C_6D_6) δ 7.29 (dd, *J* = 8.1 Hz, 1.4 Hz, 1H), 7.24 (t, *J* = 2.0 Hz, 1H), 7.15-7.14 (m, 1H), 6.37-6.33 (m, 1H), 5.93 (d, *J* = 9.7 Hz, 1H), 5.90 (d, *J* = 12.2 Hz, 1H), 5.83 (t, *J* = 0.6 Hz, 1H), 5.37-5.31 (m, 2H), 5.26-5.15 (m, 2H), 4.04-3.96 (m, 2H), 2.64 (m, 2H), 2.13-2.10 (m, 2H), 1.95-1.89 (m, 2H), 1.76 (dt, *J* = 18.4 Hz, 9.2 Hz, 1H), 1.57-1.52 (m, 1H), 1.44-1.12 (m, 5H), 1.07-0.94 (m, 2H), 0.89 (td, *J* = 10.7 Hz, 0.7 Hz, 3H), 0.58 (s, 3H); ^{13}C{^{1}H} NMR (100 MHz, C_6D_6) δ 217.6, 172.3, 143.4, 140.1, 138.5, 137.1, 136.9, 136.8, 136.7, 132.0, 131.9, 130.7, 130.0, 129.9, 129.5, 129.3, 127.45, 127.41, 126.6, 126.2, 124.1, 124.0, 123.33, 123.30, 61.1, 56.5, 50.1, 47.5, 44.3, 38.1, 35.4, 32.0, 29.7, 26.5, 25.8, 21.3, 13.9, 13.5; IR (neat) 3053, 2930, 2860, 1731, 1581, 1454, 1261, 1216, 1032 cm^{-1}; HRMS (EI) *m/z*: [M$^+$] Calcd for $C_{32}H_{34}O_3$ 466.2508; Found 466.2510.

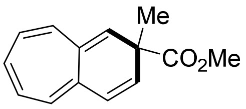

3q

Methyl 2-methyl-2*H*-benzo[7]annulene-2-carboxylate (3q)

The compound **3q** was prepared according to the same procedure as that of the synthesis of **3a**. The crude residue was purified by Silica gel column chromatography to give **3q** (36.2 mg, 84%) as a red solid. m.p. 57−59 °C; R_f = 0.4 (EtOAc:hexane = 1:5); ^{1}H NMR (400 MHz, C_6D_6) δ 5.75−5.69 (m, 3H), 5.31−5.25 (m, 2H), 5.18 (s, 1H), 5.16−5.09 (m, 2H), 3.26 (s, 3H), 1.32 (s, 3H); ^{13}C{^{1}H} NMR (100 MHz, $CDCl_3$) δ 173.5, 139.8, 137.5, 136.9, 132.7, 130.4, 130.3, 129.7, 128.5, 126.6, 124.4, 51.7, 48.2, 29.7; IR (neat) 3025, 2951, 1729, 1582, 1450, 1220, 1124 cm^{-1}; HRMS (EI) *m/z*: [M$^+$] Calcd for $C_{14}H_{14}O_2$ 214.0994; Found 214.0995.

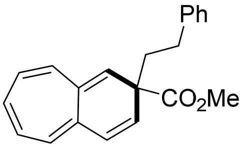

3r

Methyl 2-phenethyl-2*H*-benzo[7]annulene-2-carboxylate (3r)

The compound **3r** was prepared according to the same procedure as that of the synthesis of **3a**. The crude residue was purified by Silica gel column chromatography to give **3r** (53.1 mg, 87%) as a red oil. R_f = 0.6 (Et_2O:CH_2Cl_2:hexane = 1:2:5); ^{1}H NMR (400 MHz, C_6D_6) δ 7.13−7.11 (m, 2H), 7.07−7.03 (m, 1H), 7.01−6.99 (m, 2H), 5.85 (d, J = 9.9 Hz, 1H), 5.79−5.75 (m, 2H), 5.32−5.27 (m, 2H), 5.23 (s, 1H), 5.18−5.13 (m, 2H), 3.27 (s, 3H), 2.67−2.50 (m, 2H), 2.07−1.94 (m, 2H); $^{13}C\{^1H\}$ NMR (100 MHz, $CDCl_3$) δ 173.1, 142.0, 139.9, 138.3, 137.6, 131.8, 131.3, 130.5, 129.7, 128.8, 128.6, 128.5, 126.5, 126.0, 122.8, 52.6, 51.7, 44.0, 31.6; IR (neat) 3026, 2950, 1729, 1455, 1218, 1053 cm^{-1}; HRMS (EI) *m/z*: [M$^+$] Calcd for $C_{21}H_{20}O_2$ 304.1463; Found 304.1465.

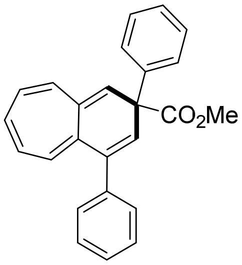

3s

Methyl 2,4-diphenyl-2*H*-benzo[7]annulene-2-carboxylate (3s)

The compound **3s** was prepared according to the same procedure as that of the synthesis of **3a**. The crude residue was purified by Silica gel column chromatography to give **3s** (50.4 mg, 70%) as a red solid. m.p. 61−63 °C; R_f = 0.3 (EtOAc:hexane = 1:20); ^{1}H NMR (400 MHz, C_6D_6) δ 7.43−7.41 (m, 2H), 7.19−7.17 (m, 2H), 7.14−7.11 (m, 2H), 7.05−7.02 (m, 3H), 7.02−7.97 (m, 1H), 6.44 (dd, J = 2.2 Hz, 0.6 Hz, 1H), 6.00 (dd, J = 12.0 Hz, 0.7 Hz, 1H), 5.91 (q, J = 0.7 Hz, 1H), 5.79 (d, J = 8.3 Hz, 1H), 5.41 (dd, J = 11.1 Hz, 7.4 Hz, 1H), 5.33−5.22 (m, 2H), 3.25 (s, 3H); $^{13}C\{^1H\}$ NMR (100 MHz, C_6D_6) δ 172.6, 145.4, 140.9, 140.3, 139.8, 137.7, 137.4, 133.0, 131.0, 129.7, 129.6, 129.2, 129.1, 128.3, 127.4, 126.9, 126.8, 126.7, 124.2, 56.8, 51.9; IR (neat) 3057, 3027, 2952, 1732, 1598, 1491, 1445, 1223, 1029 cm^{-1}; HRMS (EI) *m/z*: [M$^+$] Calcd for $C_{25}H_{20}O_2$ 352.1463; Found 352.1461.

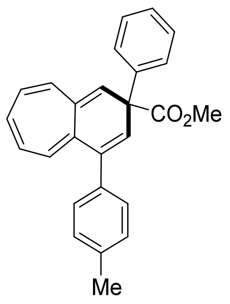

3t

Methyl 2-phenyl-4-(*p*-tolyl)-2*H*-benzo[7]annulene-2-carboxylate (3t)

The compound **3t** was prepared according to the same procedure as that of the synthesis of **3a**. The crude residue was purified by Silica gel column chromatography to give **3t** (68.0 mg, 93%) as a red solid. m.p. 56−58 °C; R_f = 0.2 (EtOAc:hexane = 1:40); ^{1}H NMR (400 MHz, C_6D_6) δ 7.45−7.42 (m, 2H), 7.15−7.11 (m, 4H), 7.01−6.97 (m, 1H), 6.88 (d, J = 7.7 Hz, 2H), 6.49 (d, J = 1.6 Hz, 1H), 6.01 (d, J = 12.7 Hz, 1H), 5.93 (s, 1H), 5.88 (d, J = 8.2 Hz, 1H), 5.43 (dd, J = 11.3 Hz, 7.1 Hz, 1H), 5.34−5.28 (m, 2H), 3.25 (s, 3H), 2.07 (s, 3H); ^{13}C{^{1}H} NMR (100 MHz, C_6D_6) δ 172.7, 145.5, 140.3, 139.8, 138.0, 137.7, 137.6, 136.9, 132.7, 131.0, 129.6, 129.5, 129.2, 129.1, 129.0, 126.9, 126.8, 126.7, 124.3, 56.8, 51.9, 20.9; IR (neat) 3025, 2951, 2921, 1732, 1585, 1491, 1434, 1221, 1032 cm^{-1}; HRMS (EI) *m/z*: [M$^+$] Calcd for $C_{26}H_{22}O_2$ 366.1620; Found 366.1622.

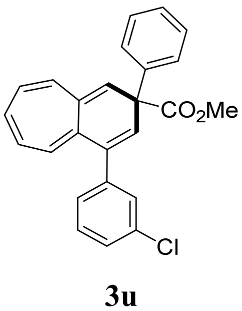

3u

Methyl 4-(3-chlorophenyl)-2-phenyl-2*H*-benzo[7]annulene-2-carboxylate (3u)

The compound **3u** was prepared according to the same procedure as that of the synthesis of **3a**. The crude residue was purified by Silica gel column chromatography to give **3u** (59.4mg, 76%) as a red solid. m.p. 51−53 °C; R_f = 0.4 (EtOAc:hexane = 1:15); ^{1}H NMR (400 MHz, C_6D_6) δ 7.38−7.35 (m, 2H), 7.22 (t, J = 1.8 Hz, 1H), 7.14−7.10 (m, 2H), 7.01−6.97 (m, 2H), 6.85 (dt, J = 7.7 Hz, 1.3 Hz, 1H), 6.70 (t, J = 7.8 Hz, 1H), 6.33 (dd, J = 2.1 Hz, 0.6 Hz, 1H), 5.96 (dd, J = 12.0 Hz, 0.7 Hz, 1H), 5.86 (q, J = 0.7 Hz, 1H), 5.59 (d, J = 8.3 Hz, 1H), 5.39 (dd, J = 11.2 Hz, 7.3 Hz, 1H), 5.29 (ddd, J = 7.4 Hz, 12.0 Hz, 0.7 Hz,

1H), 5.20 (ddt, J = 8.4 Hz, 11.2 Hz, 1.0 Hz, 1H), 3.25 (s, 3H); $^{13}C\{^1H\}$ NMR (100 MHz, C_6D_6) δ 172.4, 145.1, 142.7, 139.8, 139.0, 137.6, 136.9, 134.3, 133.4, 131.2, 129.63, 129.60, 129.1, 128.0, 127.83, 127.80, 127.1, 126.9, 126.7, 124.1, 56.7, 51.9; IR (neat) 3060, 3027, 2951, 1732, 1593, 1433, 1223, 1032 cm^{-1}; HRMS (EI) *m/z*: [M$^+$] Calcd for $C_{25}H_{19}ClO_2$ 386.1074; Found 386.1074.

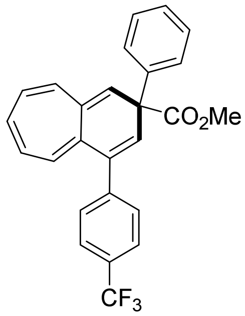

3v

Methyl 2-phenyl-4-(4-(trifluoromethyl)phenyl)-2*H*-benzo[7]annulene-2-carboxylate (3v)

The compound **3v** was prepared according to the same procedure as that of the synthesis of **3a**. The crude residue was purified by Silica gel column chromatography to give **3v** (72.1 mg, 85%) as a red solid. m.p. 27−29 °C; R_f = 0.4 (EtOAc:hexane = 1:7); ^{1}H NMR (400 MHz, C_6D_6) δ 7.41−7.38 (m, 2H), 7.20 (d, J = 8.0 Hz, 2H), 7.03−6.99 (m, 1H), 6.91 (d, J = 8.0 Hz, 2H), 6.31 (d, J = 2.0 Hz, 1H), 5.98 (d, J = 12.0 Hz, 1H), 5.87 (s, 1H), 5.43 (dd, J = 11.1 Hz, 7.4 Hz, 1H), 5.33−5.24 (m, 2H), 3.28 (s, 3H); $^{13}C\{^1H\}$ NMR (100 MHz, C_6D_6) δ 173.0, 144.5, 144.3, 139.7, 139.1, 137.2, 136.5, 132.9, 131.5, 129.9, 129.7 (q, J = 32.5 Hz), 129.4, 129.0, 129.0, 127.1, 126.9, 126.4, 125.2 (q, J = 3.7 Hz), 124.3 (q, J = 272.1 Hz), 123.4, 56.4, 52.8; IR (neat) 3058, 3030, 2953, 1733, 1615, 1435, 1325, 1223, 1167, 1124, 1065 cm^{-1}; HRMS (EI) *m/z*: [M$^+$] Calcd for $C_{26}H_{19}F_3O_2$ 420.1337; Found 420.1335.

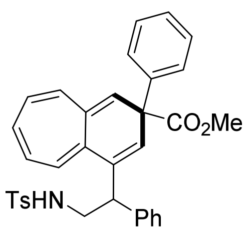

3w

Methyl 4-(2-(4-methylphenylsulfonamido)-1-phenylethyl)-2-phenyl-2*H*-benzo[7]annulene-2-carboxylate (3w)

The compound **3w** was prepared according to the same procedure as that of the synthesis of **3a**. The crude residue was purified by Silica gel column chromatography to give **3w** (97.2 mg, 88%) as a red solid. m.p. 59−61 °C; R_f = 0.4 (acetone/hexane = 1:3); ^{1}H NMR (400 MHz, C_6D_6) δ 7.90 (d, J = 8.2 Hz, 2H), 7.24−7.22 (m, 2H), 7.00−6.95 (m, 1H), 6.91−6.85 (m, 3H), 6.82−6.79 (m, 4H), 6.02 (d, J = 1.5 Hz, 1H), 5.82 (d, J = 12.7 Hz, 1H), 5.75 (dd, J = 9.8 Hz, 3.0 Hz, 1H), 5.65 (d, J = 8.5 Hz, 1H), 5.59 (s, 1H), 5.33−5.29 (m, 2H), 5.22−5.14 (m, 2H), 3.89 (dd, J = 10.3 Hz, 5.5 Hz, 1H), 3.58 (dq, J = 13.5 Hz, 5.1 Hz, 1H), 3.39 (s, 3H), 3.15 (ddd, J = 13.3 Hz, 10.3 Hz, 3.1 Hz, 1H), 1.89 (s, 3H); ^{13}C{^{1}H} NMR (100 MHz, C_6D_6) δ 173.5, 144.7, 142.6, 140.5, 139.7, 138.9, 138.8, 135.4, 133.2, 132.5, 131.2, 129.7, 129.1, 129.04, 129.00, 127.8, 127.6, 127.4, 127.1, 126.81, 126.80, 126.4, 122.1, 56.5, 52.5, 48.1, 46.4, 21.1; IR (neat) 3060, 3028, 2952, 2926, 1731, 1433, 1331, 1231, 1150, 1392 cm^{-1}; HRMS (EI) *m/z*: [M$^+$] Calcd for $C_{34}H_{31}NO_4S$ 549.1974; Found 549.1974.

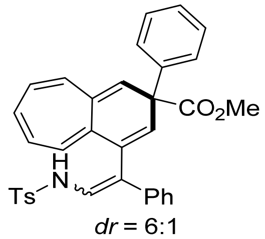

3x

Methyl 4-(2-(4-methylphenylsulfonamido)-1-phenylvinyl)-2-phenyl-2*H*-benzo[7]-annulene-2-carboxylate (3x)

Cu(hfacac)$_2$ (1.9 mg, 4.0 mol %), *N*-(2-(azulen-1-yl)-2-phenylvinyl)-4-methylbenzenesulfonamide (79.9 mg, 0.2 mmol), and DCE (3.0 mL) were added to a test tube equipped with a stirring bar. To the solution was added methyl 2-diazo-2-phenylacetate (35.2 mg, 0.2 mmol) in DCE (1.0 mL). The mixture was stirred at room temperature for 30 min. The residue was passed through a pad of Cellite to remove Cu(hfacac)$_2$ and eluted with CH_2Cl_2. The filtrate was concentrated under reduced pressure and the residue was purified by Silica gel fresh column chromatography (EtOAc:hexanes) to give **3x** (69.2 mg, 63%) as a red solid. m.p. 85−88 °C; R_f = 0.5 (EtOAc:hexane = 1:7); ^{1}H NMR (400 MHz, C_6D_6) δ 8.33 (d, J = 11.7 Hz, 1H), 7.76 (d, J = 8.2 Hz, 2H), 7.18 (d, J = 11.7 Hz, 1H), 6.98−6.96 (m, 6H), 6.92−6.81 (m, 2H), 6.78−6.69 (m, 3H), 6.51 (d, J = 8.1 Hz, 1H), 5.74 (d, J = 11.8 Hz, 1H), 5.59 (d, J = 8.4 Hz, 1H), 5.50 (s, J = 1.6 Hz, 1H), 5.42 (s, 1H), 5.18−5.08 (m, 2H), 4.94−4.89 (m, 1H), 3.19 (s, 3H), 1.65 (s, 3H); ^{13}C{^{1}H} NMR (100 MHz, C_6D_6) δ 174.3, 144.4, 142.9, 139.1, 138.9, 138.4, 137.4, 137.2, 134.2, 133.9, 131.4, 129.7, 129.5, 128.88, 128.85, 128.7, 127.3, 127.12, 127.10, 127.0, 126.9, 125.1, 124.9, 122.8,

122.2, 56.8, 52.8, 21.0; IR (neat) 3028, 2951, 1732, 1706, 1632, 1597, 1407, 1339, 1224, 1166 cm^{-1}; HRMS (EI) *m/z*: [M^+] Calcd for $C_{34}H_{29}NO_4S$ 547.1817; Found 547.1816.

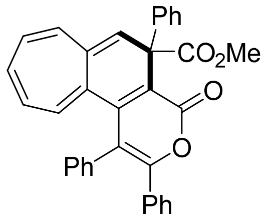

3y

Methyl 4-oxo-1,2,5-triphenyl-4,5-dihydrocyclohepta[*f*]isochromene-5-carboxylate (3y)

Cu(hfacac)$_2$ (1.9 mg, 2.0 mol %), methyl 3,4-diphenylazulenolactone (69.7 mg, 0.2 mmol), and 1,4-dioxane (3.0 mL) were added to a test tube equipped with a stirring bar. To the solution was added methyl 2-diazo-2-phenylacetate (35.3mg, 0.2 mmol) in 1,4-dioxane (1.0 mL). The mixture was stirred at 40 °C for 3 h. The residue was passed through a pad of Cellite to remove Cu(hfacac)$_2$ and eluted with CH_2Cl_2. The filtrate was concentrated under reduced pressure and the residue was purified by Silica gel fresh column chromatography to give to give **3y** (94.3 mg, 95%) as a brown solid. m.p. 233−235 °C; R_f = 0.3 (EtOAc:hexane = 1:5); ^{1}H NMR (400 MHz, $CDCl_3$) δ 7.67 (d, J = 7.2 Hz, 2H), 7.33-7.29 (m, 2H), 7.26-7.19 (m, 2H), 7.16-7.11 (m, 5H), 6.28 (d, J = 11.1 Hz, 1H), 6.01-5.92 (m, 2H), 5.61 (s, 1H), 5.56-5.51 (m, 1H), 5.39 (d, J = 7.8 Hz, 1H) 3.82 (s, 3H); ^{13}C{^{1}H} NMR (100 MHz, $CDCl_3$) δ 172.4, 159.8, 157.1, 146.6, 139.0, 137.6, 135.2, 135.0, 134.4, 133.3, 133.0, 131.84, 131.83, 130.1, 129.5, 129.3, 129.0, 128.72, 128.71, 128.6, 128.4, 128.1, 127.9, 127.7, 127.4, 116.6, 55.8, 53.1; IR (neat): 3059, 2925, 2854, 1711, 1487, 1232, 786, 697 cm^{-1}; HRMS (EI) *m/z*: [M^+] Calcd for $C_{34}H_{24}O_4$ 496.1675; Found 496.1675.

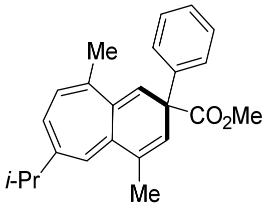

3z

Methyl 6-isopropyl-4,9-dimethyl-2-phenyl-2H-benzo[7]annulene-2-carboxylate (3z)

The compound **3z** was prepared according to the same procedure as that of the synthesis of **3a**. The crude residue was purified by Silica gel column chromatography to give **3z** (54.3 mg, 71%) as a red solid. m.p. 85−88 °C; R_f = 0.5 (EtOAc:hexane = 1:40); ^{1}H NMR (400 MHz, C_6D_6) δ 7.44−7.42 (m, 2H),

7.14−7.09 (m, 2H), 7.00−6.96 (m, 1H), 6.43 (t, J = 0.9 Hz, 1H), 6.18 (t, J = 1.5 Hz, 1H), 6.11 (s, 1H), 5.86 (d, J = 8.1 Hz, 1H), 5.66 (d, J = 8.0 Hz, 1H), 3.29 (s, 3H), 2.12 (quintet, J = 6.8 Hz, 1H), 1.89 (d, J = 0.6 Hz, 3H), 1.86 (d, J = 1.2 Hz, 3H), 0.93 (d, J = 6.8 Hz, 6H); $^{13}C\{^1H\}$ NMR (100 MHz, C_6D_6) δ 173.1, 146.4, 145.4, 139.8, 138.0, 136.2, 133.4, 132.0, 128.9, 127.6, 126.8, 126.7, 126.1, 124.5, 122.7, 56.4, 51.8, 37.0, 26.6, 22.5, 22.4, 21.0 cm^{-1}; IR (neat) 3058, 2958, 2870, 1732, 1238 cm^{-1}; HRMS (FAB) *m/z*: $[M + H]^+$ Calcd for $C_{24}H_{27}O_2$ 347.2011; Found 347.2013.

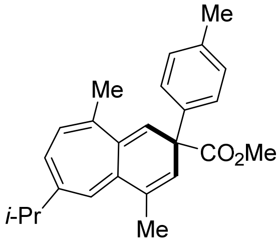

3a'

Methyl 6-isopropyl-4,9-dimethyl-2-(*p*-tolyl)-2*H*-benzo[7]annulene-2-carboxylate (3a')

The compound **3a'** was prepared according to the same procedure as that of the synthesis of **3a**. The crude residue was purified by Silica gel column chromatography to give **3a'** (61.1 mg, 85%) as a aurore solid. m.p. 83−86 °C; R_f = 0.6 (EtOAc:hexane = 1:40); ^1H NMR (400 MHz, C_6D_6) δ 7.39−7.36 (m, 2H), 6.98−6.96 (m, 2H), 6.47 (s, 1H), 6.22 (t, J = 1.4 Hz, 1H), 6.12 (s, 1H), 5.86 (d, J = 8.0 Hz, 1H), 5.66 (d, J = 8.0 Hz, 1H), 3.31 (s, 3H), 2.12 (quintet, J = 6.8 Hz, 1H), 2.03 (s, 3H), 1.91 (s, 3H), 1.88 (d, J = 1.2 Hz, 3H), 0.94 (d, J = 6.8 Hz, 6H); ^{13}C NMR (100 MHz, C_6D_6) δ 173.3, 146.4, 142.6, 139.9, 137.8, 136.3, 136.2, 133.3, 132.3, 129.6, 127.5, 126.6, 126.0, 124.4, 123.0, 56.1, 51.8, 37.0, 26.6, 22.5, 22.4, 21.0, 20.7; IR (neat) 3055, 2960, 2929, 2872, 1734, 1511, 1436, 1243, 1038 cm^{-1}; HRMS (FAB) *m/z*: $[M + H]^+$ Calcd for $C_{25}H_{29}O_2$ 361.2168; Found 361.2169.

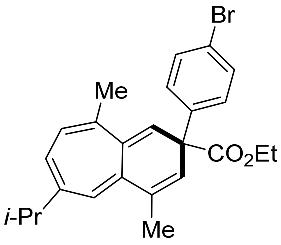

3b'

Ethyl 2-(4-bromophenyl)-6-isopropyl-4,9-dimethyl-2*H*-benzo[7]annulene-2-carboxylate (3b')

The compound **3b'** was prepared according to the same procedure as that of the synthesis of **3a**. The crude residue was purified by Silica gel column chromatography to give **3b'** (60.1 mg, 68%) as a aurore solid. m.p. 62−65 °C; R_f = 0.4 (EtOAc:hexane = 1:3); ^{1}H NMR (400 MHz, C_6D_6) δ 7.23−7.20 (m, 2H), 7.14−7.12 (m, 2H), 6.35 (s, 1H), 6.12−6.10 (m, 2H), 5.88 (d, J = 8.0 Hz, 1H), 5.67 (d, J = 8.0 Hz, 1H), 3.95−3.87 (m, 2H), 2.12 (quintet, J = 6.8 Hz, 1H), 1.91 (s, 3H), 1.87 (d, J = 1.1 Hz, 3H), 0.93 (d, J = 6.8 Hz, 6H), 0.84 (t, J = 7.1 Hz, 4H); ^{13}C{^{1}H} NMR (100 MHz, C_6D_6) δ 172.2, 146.5, 144.2, 139.7, 138.3, 136.0, 133.9, 131.9, 131.4, 128.6, 126.3, 124.6, 122.1, 121.0, 61.2, 55.8, 37.0, 26.6, 22.5, 22.4, 21.0, 13.9; IR (neat) 3055, 2963, 2936, 2869, 1730, 1488, 1385, 1237, 1010cm^{-1}; HRMS (FAB) *m/z*: [M + H]$^+$ Calcd for $C_{25}H_{28}O_2Br$ 439.1273; Found 439.1270.

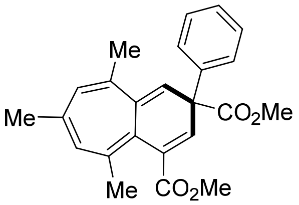

3c'

Dimethyl 5,7,9-trimethyl-2-phenyl-2*H*-benzo[7]annulene-2,4-dicarboxylate (3c')

Cu(hfacac)$_2$ (1.9 mg, 2.0 mol %), methyl 4,6,8-trimethylazulene-1-carboxylate (75.3 mg, 0.2 mmol), and 1,4-dioxane (3.0 mL) were added to a test tube equipped with a stirring bar. To the solution was added methyl 2-diazo-2-phenylacetate (35.3mg, 0.2 mmol) in 1,4-dioxane (1.0 mL). The mixture was stirred at room temperature for 30 min. The residue was passed through a pad of Cellite to remove Cu(hfacac)$_2$ and eluted with CH_2Cl_2. The filtrate was concentrated under reduced pressure and the residue was purified by Silica gel fresh column chromatography to give to give **3c'** (66.4 mg, 88%) as a pale orange solid. m.p. 109−111 °C; R_f = 0.2 (EtOAc:hexanes = 1:15); ^{1}H NMR (400 MHz, C_6D_6) δ 8.06 (d, J = 1.5 Hz, 1H), 7.48−7.46 (m, 2H), 7.04−7.00 (m, 2H), 6.93−6.89 (m, 1H), 6.60 (d, J = 1.4 Hz, 1H), 5.79 (s, 1H), 5.62 (s, 1H), 3.34 (s, 3H), 3.19 (s, 3H), 1.96 (s, 3H), 1.73 (s, 3H), 1.63 (d, J = 0.8 Hz, 3H); ^{13}C{^{1}H} NMR (100 MHz, C_6D_6) δ 172.4, 167.0, 145.0, 142.1, 141.7, 140.4, 138.6, 135.0, 134.0, 130.4, 129.2, 128.9, 127.3, 126.9, 124.7, 124.5, 56.5, 52.3, 51.5, 26.0, 25.7, 24.5; IR (neat) 3059, 2978, 2951, 2917,

2845, 1732, 1579, 1435, 1270, 1235, 1193, 1093 cm^{-1}; HRMS (EI) *m/z*: [M^+] Calcd for $C_{24}H_{24}O_4$ 376.1675; Found 376.1674.

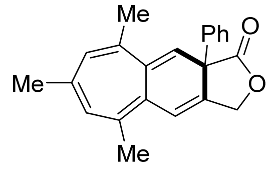

3d'

5,7,9-Trimethyl-10a-phenyl-3,10a-dihydro-1*H*-cyclohepta[f]isobenzofuran-1-one (3d')

Cu(hfacac)$_2$ (1.9 mg, 2.0 mol %), **1c'** (68.9 mg, 0.2 mmol), and DCE (4.0 mL) were added to a test tube equipped with a stirring bar. The mixture was stirred at 40 °C for 30 min. The residue was passed through a pad of Cellite to remove Cu(hfacac)$_2$ and eluted with CH_2Cl_2. The filtrate was concentrated under reduced pressure and the residue was purified by Silica gel fresh column chromatography to give **3d'** (60.3 mg, 95%) as an orange solid. m.p. 139−141 °C; R_f = 0.2 (EtOAc:hexane = 1:20); ^{1}H NMR (400 MHz, C_6D_6) δ 7.52−7.50 (m, 2H), 7.04−7.00 (m, 2H), 6.93−6.89 (m, 1H), 6.43 (s, 1H), 6.22 (s, 1H), 5.71 (s, 1H), 5.69 (s, 1H), 4.32 (dd, *J* = 2.1 Hz, 12.3 Hz, 1H), 4.17 (d, *J* = 12.4 Hz, 1H), 1.82 (s, 3H), 1.72 (s, 3H), 1.60 (s, 3H); $^{13}C\{^1H\}$ NMR (100 MHz, C_6D_6) δ 175.6, 141.6, 139.5, 138.7, 138.6, 138.2, 133.8, 130.7, 129.8, 129.3, 128.2, 127.7, 126.1, 123.5, 123.1, 68.4, 53.7, 26.3, 25.6, 22.8; IR (neat) 3058, 3024, 2972, 2934, 2884, 1773, 1577, 1446, 1372, 1140, 999 cm^{-1}; HRMS (EI) *m/z*: [M^+] Calcd for $C_{22}H_{20}O_2$ 316.1463; Found 316.1461.

C. C-H Functionalization of azulenes with alkyl and aryl diazo esters

Screening of reactions conditions

Screening experiments for optimization were carried out using azulene (**1a**) and methyl 2-diazo-2-phenylacetate (**2a**). The conditions for each trial are specified in the footnote. All yields were determined by analysis of the crude ^{1}H NMR spectrum using CH_2Br_2 as the internal standard after filtration of the reaction mixture through a pad of silica gel unless otherwise noted.

Supplementary Table 4. Optimization of C-H Functionalization

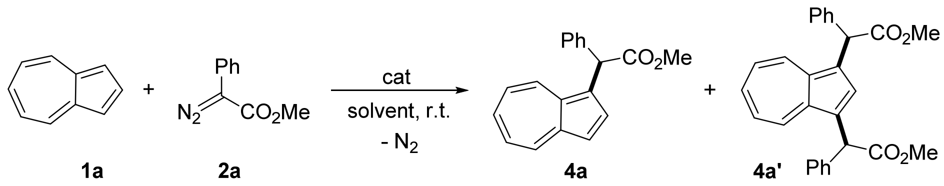

Entry	Catalyst (mol %)	Solvent	Azulene (equiv)	Time (h)	Conv. (%)	Yield (%)	
						4a	4a'
1	(IPr)AuCl (2)/$AgBF_4$ (2)	DCE	1	4	100	24	0
2	(IPr)AuCl (2)/$AgNTf_2$ (2)	DCE	1	4	100	42	0
3	(IPr)AuCl (2)/AgOTf (2)	DCE	1	1	100	40	21
4	(IPr)AuCl (2)/$AgPF_6$ (2)	DCE	1	20	85	14	0
5	(IPr)AuCl (2)/$AgSbF_6$ (2)	DCE	1	20	80	13	0
7	(IPr)AuCl (2)/AgOTf (2)	DCE	1.5	1	100	31	5
8	(IPr)AuCl (2)	DCE	1.5	1	0	0	0
9	AgOTf (2)	DCE	1.5	1	100	50	8
10	AgOTf (2)	DCE	1.5	1	100	62	16
11[a]	AgOTf (2)	DCE	1.5	1	100	59	14
12[b]	AgOTf (2)	DCE	1.5	1	100	57	15
13	AgOTf (2)	DCM	1.5	3	100	60	7
14	AgOTf (2)	toluene	1.5	3	80	0	0
15	AgOTf (2)	MeCN	1.5	3	100	51	8
16	AgOTf (2)	$CHCl_3$	1.5	3	50	33	0
17[c]	AgOTf (2)	DCE	2	1	100	74	5
18	AgOTf (5)	DCE	2	1	100	61	10

Conversions and yields were determined by ^1H NMR intergration methods with CH_2Br_2 as an internal standard. Entries 1-9 and 10-18 were conducted in 0.1 M and 0.05 M solutions, respectively. [a]Reaction temperature: 0 °C. [b]Methyl phenyl diazoacetate was added dropwise. [c]Isolated yield.

General procedure

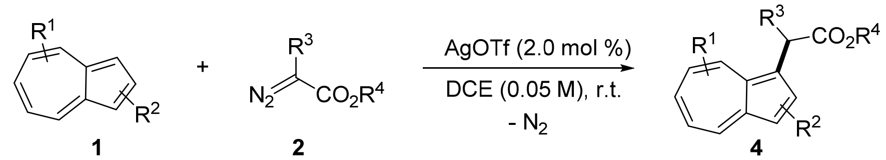

AgOTf (2.0 mol %), azulene derivatives **1** (0.2 mmol), and DCE (3.0 mL) were added to a test tube equipped with a stirring bar. To the solution was added aryl diazoacetate derivatives **2** (0.3 mmol) in DCE (1.0 mL). The mixture was stirred at room temperature. Completion of the reaction was indicated by the TLC. The residue was passed through a pad of Cellite to remove AgOTf and eluted with CH_2Cl_2. The filtrate was concentrated under reduced pressure and the residue was purified by Silica gel fresh column chromatography to give **4**.

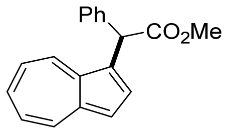

4a

Methyl 2-(azulen-1-yl)-2-phenylacetate (4a)

AgOTf (1.0 mg, 2.0 mol %), azulene **1a** (25.6 mg, 0.2 mmol), and DCE (3.0 mL) were added to a test tube equipped with a stirring bar. To the solution was added methyl phenyl diazoacetate **2a** (0.3 mmol) in DCE (1.0 mL). The mixture was stirred at room temperature. Completion of the reaction was indicated by the TLC. The residue was passed through a pad of Cellite to remove AgOTf and eluted with CH_2Cl_2. The filtrate was concentrated under reduced pressure and the residue was purified by Silica gel fresh column chromatography to give **4a** (41.1 mg, 74%).

Blue oil; R_f = 0.3 (EtOAc:hexane = 1:5); ^{1}H NMR (400 MHz, $CDCl_3$) δ 8.32 (d, J = 5.4 Hz, 1H), 8.30 (d, J = 5.7 Hz, 1H), 7.99 (d, J = 3.9 Hz, 1H), 7.58 (t, J = 9.9 Hz, 1H), 7.38 (d, J = 4.0 Hz, 1H), 7.36−7.34 (m, 2H), 7.32−7.27 (m, 2H), 7.25−7.22 (m, 1H), 7.149 (t, J = 9.8 Hz, 1H), 7.146 (t, J = 10.0 Hz, 1H), 5.71 (s, 1H), 3.75 (s, 3H); ^{13}C{^{1}H} NMR (100 MHz, $CDCl_3$) δ 173.5, 14101, 139.4, 137.8, 137.2, 137.1, 135.8, 133.2, 128.6, 128.4, 127.1, 125.7, 123.2, 122.7, 117.2, 52.4, 50.1; IR (neat) 3027, 2950, 1737, 1577, 1496, 1454, 1433, 1396, 1309, 1193, 1153 cm^{-1}; HRMS (EI) m/z: [M$^+$] Calcd for $C_{19}H_{16}O_2$ 276.1150; Found 276.1149.

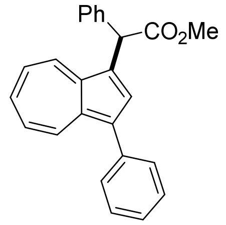

4b

Methyl 2-phenyl-2-(3-phenylazulen-1-yl)acetate (4b)

The compound **4b** was prepared according to the same procedure as that of the synthesis of **4a**. The crude residue was purified by Silica gel column chromatography to give **4b** (56.0 mg, 80%) as a blue oil. R_f = 0.5 (EtOAc:hexane = 1:5); ^{1}H NMR (400 MHz, $CDCl_3$) δ 8.50 (d, J = 9.4 Hz, 1H), 8.29 (d, J = 9.4 Hz, 1H), 8.09 (s, 1H), 7.598 (d, J = 8.2 Hz, 1H), 7.595 (d, J = 8.0 Hz, 1H), 7.55 (d, J = 9.8 Hz, 1H), 7.47 (t, J = 7.7 Hz, 2H), 7.39 (d, J = 7.2 Hz, 2H), 7.35−7.29 (m, 3H), 7.24 (t, J = 7.2 Hz, 1H), 7.11 (t, J = 9.9 Hz, 2H), 5.72 (s, 1H), 3.76 (s, 3H); ^{13}C{^{1}H} NMR (100 MHz, $CDCl_3$) δ 173.4, 139.2, 138.6, 137.4,

137.17, 137.09, 136.1, 135.9, 133.6, 130.2, 129.8, 128.6, 128.4, 127.2, 126.4, 125.1, 123.7, 122.8, 52.4, 50.0; IR (neat) 3026, 2949, 1736, 1570, 1493, 1430, 1153 cm^{-1}; HRMS (EI) *m/z*: [M^+] Calcd for $C_{25}H_{20}O_2$ 352.1463; Found 352.1461.

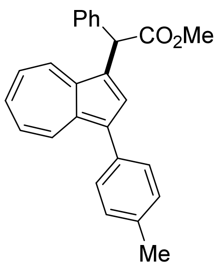

4c

Methyl 2-phenyl-2-(3-(*p*-tolyl)azulen-1-yl)acetate (4c)

The compound **4c** was prepared according to the same procedure as that of the synthesis of **4a**. The crude residue was purified by Silica gel column chromatography to give **4c** (63.4 mg, 86%) as a blue solid. m.p. 39−41 °C; R_f = 0.3 (EtOAc:pentane = 1:5); ^{1}H NMR (400 MHz, $CDCl_3$) δ 8.50 (d, J = 9.7 Hz, 1H), 8.28 (d, J = 9.6 Hz, 1H), 8.07 (s, 1H), 7.55 (t, J = 9.8 Hz, 1H), 7.49 (d, J = 8.0 Hz, 2H), 7.39 (d, J = 7.5 Hz, 2H), 7.33−7.22 (m, 5H), 7.09 (t, J = 9.8 Hz, 2H), 5.72 (s, 1H), 3.76 (s, 3H), 2.42 (s, 3H); ^{13}C{^{1}H} NMR (100 MHz, $CDCl_3$) δ 173.4, 139.2, 138.5, 137.3, 137.1, 136.15, 136.11, 136.0, 134.2, 133.6, 130.3, 129.7, 129.4, 128.6, 128.5, 127.2, 125.0, 123.5, 122.7, 52.4, 50.0, 21.2; IR (neat) 3026, 2948, 2921, 1737, 1571, 1432, 1194, 1154 cm^{-1}; HRMS (EI) *m/z*: [M^+] Calcd for $C_{26}H_{22}O_2$ 366.1620; Found 366.1620.

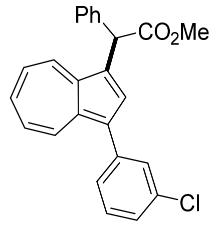

4d

Methyl 2-(3-(3-chlorophenyl)azulen-1-yl)-2-phenylacetate (4d)

AgOTf (1.0 mg, 2.0 mol %), 1-(3-chlorophenyl)azulene (47.7 mg, 0.2 mmol) and DCE (3.0 mL) were added to a test tube equipped with a stirring bar. To the solution was added methyl 2-diazo-2-phenylacetate (52.9 mg, 0.3 mmol) in DCE (1.0 mL). The mixture was stirred at 50 °C for 1 h.

Completion of the reaction was indicated by the TLC. The residue was passed through a pad of Cellite to remove AgOTf and eluted with CH_2Cl_2. The filtrate was concentrated under reduced pressure and the residue was purified by Silica gel fresh column chromatography to give **4d** (55.2 mg, 72%) as a green oil. R_f = 0.4 (EtOAc:hexane = 1:5); ^{1}H NMR (400 MHz, $CDCl_3$) δ 8.49 (d, J = 9.4 Hz, 1H), 8.31 (d, J = 9.4 Hz, 1H), 8.06 (s, 1H), 7.61 (t, J = 9.6 Hz, 1H), 7.57 (t, J = 1.8 Hz, 1H), 7.47 (td, J = 7.6 Hz, 1.3 Hz, 1H), 7.42–7.38 (m, 3H), 7.34–7.27 (m, 4H), 7.17 (dd, J = 9.8 Hz, 3.5 Hz, 1H), 7.15 (dd, J = 9.6 Hz, 3.6 Hz, 1H), 5.71 (s, 1H), 3.77 (s, 3H); ^{13}C{^{1}H} NMR (100 MHz, $CDCl_3$) δ 173.3, 139.0, 138.9, 138.8, 137.3, 137.2, 136.2, 135.7, 134.4, 133.8, 129.8, 129.6, 128.6, 128.5, 128.3, 127.9, 127.2, 126.3, 125.3, 124.1, 123.3, 52.5, 49.9; IR (neat) 3025, 2949, 2850, 1735, 1591, 1432, 1154, 727 cm^{-1}; HRMS (EI) *m/z*: [M$^+$] Calcd for $C_{25}H_{19}ClO_2$ 386.1074; Found 386.1071.

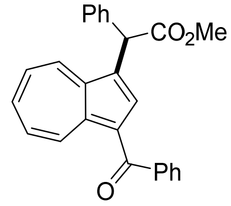

4e

Methyl 2-(3-benzoylazulen-1-yl)-2-phenylacetate (4e)

AgOTf (1.0 mg, 2.0 mol %), azulen-1-yl(phenyl)methanone (46.5 mg, 0.2 mmol) and DCE (3.0 mL) were added to a test tube equipped with a stirring bar. To the solution was added methyl 2-diazo-2-phenylacetate (105.7 mg, 0.6 mmol) in DCE (1.0 mL). The mixture was stirred at 70 °C for 15 h. Completion of the reaction was indicated by the TLC. The residue was passed through a pad of Cellite to remove AgOTf and eluted with CH_2Cl_2. The filtrate was concentrated under reduced pressure and the residue was purified by Silica gel fresh column chromatography to give **4e** (47.1 mg, 61%) as a purple solid. m.p. 48–50 °C; R_f = 0.3 (EtOAc:hexane = 1:3); ^{1}H NMR (400 MHz, $CDCl_3$) δ 9.71 (dd, J = 9.9 Hz, 0.7 Hz, 1H), 8.45 (d, J = 9.7 Hz, 1H), 7.87 (m, 3H), 7.62 (t, J = 9.8 Hz, 1H), 7.58–7.54 (m, 1H), 7.51–7.46 (m, 3H), 7.33–7.23 (m, 5H), 5.65 (s, 1H), 3.19 (s, 3H); ^{13}C{^{1}H} NMR (100 MHz, $CDCl_3$) δ 192.7, 173.0, 142.6, 142.3, 141.1, 141.0, 140.0, 139.4, 138.6, 135.2, 131.4, 129.7, 129.4, 128.7, 128.3, 128.2, 127.41, 127.40, 125.3, 123.7, 52.5, 49.9; IR (neat) 3058, 3028, 2950, 1736, 1594, 1424, 1396, 1239, 1155 cm^{-1}; HRMS (EI) *m/z*: [M$^+$] Calcd for $C_{26}H_{20}O_3$ 380.1412; Found 380.1410.

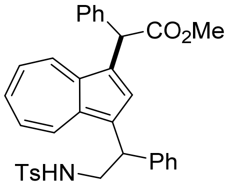

dr =1.5 : 1

4f

Methyl 2-(3-(2-(4-methylphenylsulfonamido)-1-phenylethyl)azulen-1-yl)-2-phenyl-acetate (4f)

The compound **4f** was prepared according to the same procedure as that of the synthesis of **4a**. The crude residue was purified by Silica gel column chromatography to give **4f-1** (53.4 mg, 48.6%) and **4f-2** (35.6 mg, 32.4%) as a blue solid. Major isomer; m.p. 69−71 °C; R_f = 0.2 (EtOAc:hexane = 1:3); ^{1}H NMR (400 MHz, $CDCl_3$) δ 8.24 (d, J = 9.7 Hz, 1H), 8.09 (d, J = 9.6 Hz, 1H), 7.84 (s, 1H), 7.62 (d, J = 8.3 Hz, 2H), 7.54 (t, J = 9.8 Hz, 1H), 7.35−7.11 (m, 12H), 7.09 (t, J = 9.6 Hz, 1H), 7.04 (t, J = 9.7 Hz, 1H), 5.66 (s, 1H), 4.72 (t, J = 7.8 Hz, 1H), 4.35 (s, 1H), 3.71 (s, 3H), 3.71−3.56 (m, 2H), 2.42 (s, 3H); ^{13}C{^{1}H} NMR (100 MHz, $CDCl_3$) δ 172.2, 142.4, 140.6, 138.2, 137.4, 136.2, 135.8, 135.4, 133.7, 132.8, 132.6, 128.8, 127.8, 127.6, 127.2, 126.8, 126.2, 126.0, 125.9, 123.8, 121.9, 51.3, 48.8, 47.3, 42.4, 20.5; IR (neat) 3060, 3027, 2951, 2924, 1735, 1710, 1645, 1431, 1329, 1159 cm^{-1}; HRMS (EI) *m/z*: [M$^+$] Calcd for $C_{34}H_{31}NO_4S$ 549.1974; Found 549.1976.

Minor isomer; m.p. 67−69 °C; R_f = 0.15 (EtOAc:hexane = 1:3); ^{1}H NMR (400 MHz, $CDCl_3$) δ 8.24 (d, J = 9.6 Hz, 1H), 8.09 (d, J = 9.6 Hz, 1H), 7.86 (s, 1H), 7.67 (dd, J = 6.6 Hz, 1.7 Hz, 2H), 7.56 (t, J = 9.8 Hz, 1H), 7.33−7.10 (m, 13H), 7.05 (t, J = 9.4 Hz, 1H), 5.67 (s, 1H), 4.72 (t, J = 7.9 Hz, 1H), 4.40 (t, J = 5.9 Hz, 1H), 3.74 (s, 3H), 3.72−3.59 (m, 2H), 2.44 (s, 3H); ^{13}C{^{1}H} NMR (100 MHz, $CDCl_3$) δ 173.4, 143.5, 141.7, 139.2, 138.5, 137.3, 137.0, 136.5, 134.7, 133.9, 133.6, 129.8, 128.8, 128.6, 128.2, 127.8, 127.2, 126.95, 126.92, 124.8, 123.0, 52.5, 49.8, 48.4, 43.4, 21.6; IR (neat) 3060, 3028, 2926, 2853, 1712, 1587, 1490, 1432, 1261, 1212, 1153 cm^{-1}; HRMS (EI) *m/z*: [M$^+$] Calcd for $C_{34}H_{31}NO_4S$ 549.1974; Found 549.1977.

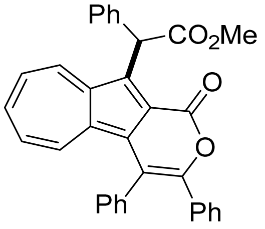

4g

Methyl -2-(1-oxo-3,4-diphenyl-1*H*-azuleno[2,1-*c*]pyran-10-yl)-2-phenylacetate (4g)

The compound **4g** was prepared according to the same procedure as that of the synthesis of **4d**. The crude residue was purified by Silica gel column chromatography to give **4g** (69.5 mg, 70%) as a blue solid. m.p. 195−197 °C; R_f = 0.3 (EtOAc:hexane = 1:5); ^{1}H NMR (400 MHz, $CDCl_3$) δ 8.44 (d, J = 10.4 Hz, 1H), 7.58 (d, J = 9.5 Hz, 1H), 7.50-7.45 (m, 4H), 7.41-7.39 (m, 4H), 7.36-7.33 (m, 2H), 7.30-7.26 (m, 2H), 7.23-7.15 (m, 4H), 6.98 (t, J = 9.8 Hz, 1H), 6.93 (s, 1H), 6.69 (t, J = 9.9 Hz, 1H), 3.77 (s, 3H); ^{13}C{^{1}H} NMR (100 MHz, $CDCl_3$) δ 173.6, 161.4, 149.6, 143.0, 141.8, 140.4, 138.7, 137.0, 136.7, 135.9, 133.6, 131.4, 130.3, 129.44, 129.40, 128.9, 128.53, 128.49, 128.4, 127.9, 127.0, 125.4, 125.1, 124.8, 124.6, 116.6, 52.6, 48.1; IR (neat): 3059, 2924, 2283, 1716, 1495, 1199, 699 cm^{-1}; HRMS (EI) *m/z*: [M$^+$] Calcd for $C_{34}H_{24}O_4$ 496.1675; Found 496.1673.

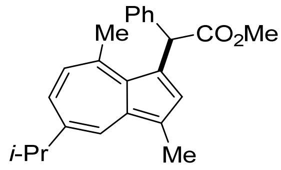

4h

Methyl 2-phenyl-2-(3-(*p*-tolyl)azulen-1-yl)acetate (4h)

The compound **4h** was prepared according to the same procedure as that of the synthesis of **4d**. The crude residue was purified by Silica gel column chromatography to give **4h** (52.2 mg, 74%) as a blue oil. R_f = 0.2 (EtOAc:hexane = 1:20); ^{1}H NMR (400 MHz, $CDCl_3$) δ 8.12 (d, J = 2.1 Hz, 1H), 7.61 (s, 1H), 7.31−7.27 (m, 3H), 7.24−7.20 (m, 1H), 7.18−7.16 (m, 2H), 6.87 (d, J = 10.8 Hz, 1H), 6.10 (s, 1H), 3.74 (s, 3H), 3.03 (sextet, J = 6.9 Hz, 1H), 2.91 (s, 3H), 2.61 (s, 3H), 1.33 (dd, J = 6.9 Hz, 0.7 Hz, 6H); ^{13}C{^{1}H} NMR (100 MHz, $CDCl_3$) δ 174.4, 144.7, 140.8, 140.1, 138.8, 138.2, 134.9, 134.0, 132.9, 128.8, 128.5, 127.4, 126.8, 124.7, 122.5, 52.3, 52.0, 37.7, 27.4, 24.6, 13.1; IR (neat) 2957, 2929, 2869, 1739, 1545, 1450, 1159 cm^{-1}; HRMS (EI) *m/z*: [M$^+$] Calcd for $C_{24}H_{26}O_2$ 346.1933; Found 346.1933.

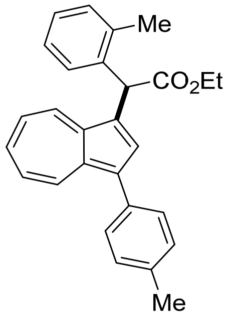

4i

Ethyl 2-(*o*-tolyl)-2-(3-(*p*-tolyl)azulen-1-yl)acetate (4i)

The compound **4i** was prepared according to the same procedure as that of the synthesis of **4a**. The crude residue was purified by Silica gel column chromatography to give **4i** (55.3 mg, 70%) as a blue oil. R_f = 0.2 (EtOAc:hexane = 1:20); ^{1}H NMR (400 MHz, $CDCl_3$) δ 8.50 (d, J = 9.6 Hz, 1H), 8.20 (d, J = 9.6 Hz, 1H), 7.91 (s, 1H), 7.55 (t, J = 9.8 Hz, 1H), 7.48 (d, J = 8.0 Hz, 2H), 7.28 (d, J = 7.9 Hz, 2H), 7.25−7.13 (m, 4H), 7.09 (t, J = 9.7 Hz, 1H), 7.08 (J = 9.7 Hz, 1H), 5.82 (s, 1H), 4.30−4.18 (m, 2H), 2.42 (s, 3H), 2.39 (s, 3H), 1.26 (t, J = 7.1 Hz, 3H); ^{13}C{^{1}H} NMR (100 MHz, $CDCl_3$) δ 173.0, 138.4, 137.9, 137.7, 137.2, 136.12, 136.10, 136.02, 136.00, 134.2, 133.4, 130.5, 130.2, 129.7, 129.3, 128.3, 127.1, 126.3, 124.9, 123.4, 122.5, 61.2, 47.1, 21.2, 19.9, 14.3; IR (neat) 3021, 2978, 2924, 1734, 1572, 1461, 1367, 1155, 1031 cm^{-1}; HRMS (EI) *m/z*: [M$^+$] Calcd for $C_{28}H_{26}O_2$ 394.1933; Found 394.1931.

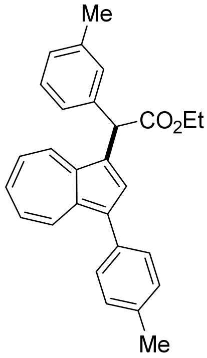

4j

Ethyl 2-(*m*-tolyl)-2-(3-(*p*-tolyl)azulen-1-yl)acetate (4j)

The compound **4j** was prepared according to the same procedure as that of the synthesis of **4d**. The crude residue was purified by Silica gel column chromatography to give **4j** (57.0 mg, 72%) as a blue oil. R_f = 0.5 (EtOAc:hexane = 1:10); ^{1}H NMR (400 MHz, $CDCl_3$) δ 8.49 (d, J = 9.8 Hz, 1H), 8.29 (d, J = 9.7 Hz, 1H), 8.09 (s, 1H), 7.55−7.48 (m, 3H), 7.28 (d, J = 8.0 Hz, 2H), 7.24−7.19 (m, 3H), 7.10−7.04 (m, 3H), 5.65 (s, 1H), 4.22 (q, J = 7.1 Hz, 2H), 2.42 (s, 3H), 2.30 (s, 3H), 1.26 (t, J = 7.1 Hz, 3H); ^{13}C{^{1}H}

NMR (100 MHz, $CDCl_3$) δ 173.0, 139.3, 138.4, 138.2, 137.4, 137.1, 136.1, 135.9, 134.3, 133.6, 130.3, 129.7, 129.5, 129.4, 129.2, 128.5, 127.9, 125.5, 125.4, 123.4, 122.6, 61.2, 50.1, 21.5, 21.2, 14.3; IR (neat) 3021, 2979, 2921, 2860, 1733, 1571, 1429, 1367, 1156, 1032 cm^{-1}; HRMS (EI) *m/z*: [M^+] Calcd for $C_{28}H_{26}O_2$ 394.1933; Found 394.1935.

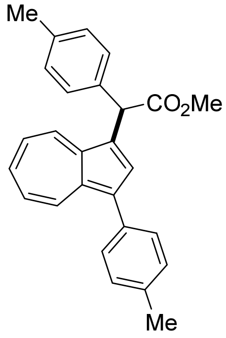

4k

Methyl 2-(*p*-tolyl)-2-(3-(*p*-tolyl)azulen-1-yl)acetate (4k)

The compound **4k** was prepared according to the same procedure as that of the synthesis of **4d**. The crude residue was purified by Silica gel column chromatography to give **4k** (50.1 mg, 70%) as a blue oil. R_f = 0.5 (EtOAc:hexane = 1:5); ^{1}H NMR (400 MHz, $CDCl_3$) δ 8.49 (d, *J* = 9.4 Hz, 1H), 8.27 (d, *J* = 9.4 Hz, 1H), 8.06 (s, 1H), 7.55 (t, *J* = 9.8 Hz, 1H), 7.49 (d, *J* = 8.0 Hz, 2H), 7.29−7.26 (m, 4H), 7.12−7.06 (m, 4H), 5.67 (s, 1H), 3.75 (s, 3H), 2.42 (s, 3H), 2.30 (s, 3H); $^{13}C\{^1H\}$ NMR (100 MHz, $CDCl_3$) δ 173.5, 138.4, 137.3, 137.0, 136.8, 136.2, 136.1, 136.0, 135.9, 134.1, 133.5, 130.2, 129.7, 129.34, 129.30, 129.3, 125.2, 123.4, 122.6, 52.4, 49.6, 21.2, 21.0; IR (neat) 3021, 2948, 2919, 1737, 1569, 1509, 1431, 1191, 1154 cm^{-1}; HRMS (EI) *m/z*: [M^+] Calcd for $C_{27}H_{24}O_2$ 380.1776; Found 380.1776.

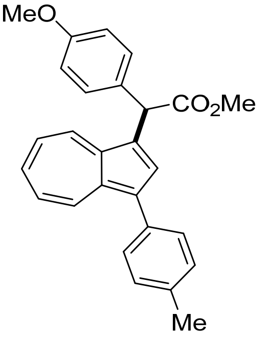

4l

Methyl 2-(4-methoxyphenyl)-2-(3-(*p*-tolyl)azulen-1-yl)acetate (4l)

The compound **4l** was prepared according to the same procedure as that of the synthesis of **4a**. The crude residue was purified by Silica gel column chromatography to give **4l** (65.2 mg, 82%) as a green oil. R_f = 0.2 (EtOAc:hexane = 1:5); ^{1}H NMR (400 MHz, $CDCl_3$) δ 8.49 (d, J = 9.5 Hz, 1H), 8.26 (d, J = 9.4 Hz, 1H), 8.05 (s, 1H), 7.55 (t, J = 9.8 Hz, 1H), 7.49 (d, J = 8.0 Hz, 2H), 7.32−7.28 (m, 4H), 7.08 (t, J = 9.8 Hz, 2H), 6.84 (d, J = 8.8 Hz, 2H), 5.66 (s, 1H), 3.77 (s, 3H), 3.75 (s, 3H), 2.43 (s, 3H); $^{13}C\{^1H\}$ NMR (100 MHz, $CDCl_3$) δ 173.6, 158.6, 138.5, 137.2, 136.9, 136.1, 136.0, 135.9, 134.1, 133.5, 131.3, 130.2, 129.7, 129.4, 129.3, 125.3, 123.4, 122.6, 113.9, 55.2, 52.4, 49.2, 21.2; IR (neat) 3020, 2949, 2927, 1736, 1570, 1509, 1251, 1155 cm^{-1}; HRMS (EI) *m/z*: [M^+] Calcd for $C_{27}H_{24}O_3$ 396.1725; Found 396.1722.

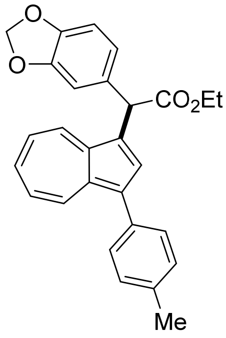

4m

Ethyl 2-(benzo[d][1,3]dioxol-5-yl)-2-(3-(*p*-tolyl)azulen-1-yl)acetate (4m)

The compound **4m** was prepared according to the same procedure as that of the synthesis of **4a**. The crude residue was purified by Silica gel column chromatography to give **4m** (61.1 mg, 72%) as a blue solid. m.p. 51−53 °C; R_f = 0.6 (EtOAc:hexane = 1:4); ^{1}H NMR (400 MHz, $CDCl_3$) δ 8.49 (d, J = 9.8 Hz, 1H), 8.25 (d, J = 9.7 Hz, 1H), 8.09 (s, 1H), 7.54 (t, J = 9.8 Hz, 1H), 7.49 (d, J = 7.9 Hz, 2H), 7.29 (d, J = 8.1 Hz, 2H), 7.08 (t, J = 9.9 Hz, 2H), 6.88−6.84 (m, 2H), 6.73 (d, J = 8.0 Hz, 1H), 5.93−5.89 (m, 2H), 5.60 (s, 1H), 4.22 (q, J = 7.1 Hz, 2H), 2.42 (s, 3H), 1.26 (t, J = 7.1 Hz, 3H); $^{13}C\{^1H\}$ NMR (100 MHz, $CDCl_3$) δ 172.9, 147.8, 146.7, 138.5, 137.1, 137.0, 136.14, 136.10, 136.0, 134.2, 133.6, 133.3, 130.2, 129.7, 129.4, 125.2, 123.4, 122.6, 121.6, 109.1, 108.2, 101.0, 61.3, 49.8, 21.2, 14.2; IR (neat) 3024, 2980, 1731, 1502, 1488, 1248, 1154, 1038 cm^{-1}; HRMS (EI) *m/z*: [M^+] Calcd for $C_{28}H_{24}O_4$ 424.1675; Found 424.1672.

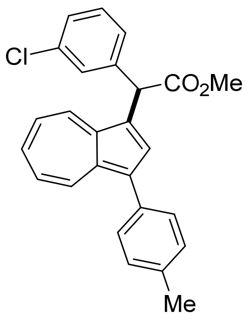

4n

Methyl 2-(3-chlorophenyl)-2-(3-(*p*-tolyl)azulen-1-yl)acetate (4n)

The compound **4n** was prepared according to the same procedure as that of the synthesis of **4d**. The crude residue was purified by Silica gel column chromatography to give **4n** (57.0 mg, 71%) as a green oil. R_f = 0.5 (EtOAc:hexane = 1:5); ^{1}H NMR (400 MHz, $CDCl_3$) δ 8.51 (d, J = 9.6 Hz, 1H), 8.24 (d, J = 9.6 Hz, 1H), 8.04 (s, 1H), 7.57 (t, J = 9.8 Hz, 1H), 7.49 (d, J = 8.0 Hz, 2H), 7.37 (s, 1H), 7.30 (d, J = 8.0 Hz, 2H), 7.29−7.22 (m, 3H), 7.11 (t, J = 9.8 Hz, 2H), 5.67 (s, 1H), 3.77 (s, 3H), 2.43 (s, 3H); ^{13}C{^{1}H} NMR (100 MHz, $CDCl_3$) δ 172.8, 141.1, 138.7, 137.08, 137.04, 136.2, 136.19, 136.16, 134.4, 134.0, 133.5, 130.4, 129.8, 129.7, 129.4, 128.6, 127.4, 126.6, 124.0, 123.7, 122.8, 52.5, 49.6, 21.2; IR (neat) 3021, 2949, 2919, 1737, 1571, 1430, 1193, 1157 cm^{-1}; HRMS (EI) *m/z*: [M$^+$] Calcd for $C_{26}H_{21}ClO_2$ 400.1230; Found 400.1230.

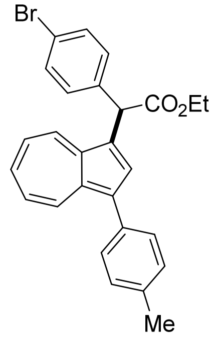

4o

Ethyl 2-(4-bromophenyl)-2-(3-(*p*-tolyl)azulen-1-yl)acetate (4o)

The compound **4o** was prepared according to the same procedure as that of the synthesis of **4e** with diazo compound (1.5 equiv) at 70 °C. The crude residue was purified by Silica gel column chromatography to give **4o** (60.5 mg, 65%) as a blue oil. R_f = 0.4 (EtOAc:hexane = 1:5); ^{1}H NMR (400 MHz, $CDCl_3$) δ 8.50 (d, J = 9.6 Hz, 1H), 8.23 (d, J = 9.6 Hz, 1H), 8.04 (s, 1H), 7.56 (t, J = 9.8 Hz, 1H),

7.48 (d, J = 8.0 Hz, 2H), 7.42 (d, J = 8.4 Hz, 2H), 7.31−7.25 (m, 4H), 7.11 (dd, J = 9.7 Hz, 3.7 Hz, 1H), 7.09 (dd, J = 9.7 Hz,, 3.6 Hz, 1H), 5.63 (s, 1H), 4.21 (qd, J = 11.8 Hz, 1.6 Hz, 2H), 2.43 (s, 3H), 1.26 (t, J = 7.1 Hz, 3H); $^{13}C\{^1H\}$ NMR (100 MHz, $CDCl_3$) δ 172.4, 138.6, 138.4, 137.06, 137.02, 136.2, 136.1, 134.0, 133.5, 131.6, 130.3, 130.2, 129.6, 129.4, 124.4, 123.6, 122.7, 121.1, 61.4, 49.6, 21.2, 14.2; IR (neat) 3021, 2978, 2922, 1732, 1571, 1486, 1367, 1154, 1010 cm^{-1}; HRMS (EI) *m/z*: [M^+] Calcd for $C_{27}H_{23}BrO_2$ 458.0881; Found 458.0883.

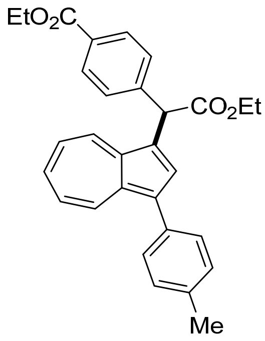

4p

Ethyl 4-(2-ethoxy-2-oxo-1-(3-(*p*-tolyl)azulen-1-yl)ethyl)benzoate (4p)

The compound **4p** was prepared according to the same procedure as that of the synthesis of **4d**. The crude residue was purified by Silica gel column chromatography to give **4p** (66.3 mg, 73%) as a blue oil. R_f = 0.4 (EtOAc:hexane = 1:10); ^1H NMR (400 MHz, $CDCl_3$) δ 8.51 (d, J = 9.6 Hz, 1H), 8.25 (d, J = 9.6 Hz, 1H), 8.05 (s, 1H), 8.00−7.97 (m, 2H), 7.55 (t, J = 9.8 Hz, 1H), 7.48 (d, J = 7.8 Hz, 2H), 7.46 (d, J = 8.0 Hz, 2H), 7.29 (d, J = 7.8 Hz, 2H), 7.10 (t, J = 9.7 Hz, 1H), 7.09 (t, J = 9.7 Hz, 1H), 5.74 (s, 1H), 4.34 (q, J = 7.1 Hz, 2H), 4.24 (qd, J = 7.1 Hz, 1.4 Hz, 2H), 2.42 (s, 3H), 1.36 (t, J = 7.1 Hz, 3H), 1.26 (t, J = 7.1 Hz, 3H); $^{13}C\{^1H\}$ NMR (100 MHz, $CDCl_3$) δ 172.3, 166.4, 144.4, 138.6, 137.2, 137.1, 136.22, 136.13, 134.0, 133.6, 130.4, 129.8, 129.7, 129.4, 124.4, 123.6, 122.8, 61.5, 60.9, 50.2, 21.2, 14.3, 14.2; IR (neat) 2980, 1716, 1572, 1366, 1277, 1155, 1106, 1022 cm^{-1}; HRMS (EI) *m/z*: [M^+] Calcd for $C_{30}H_{28}O_4$ 452.1988; Found 452.1986.

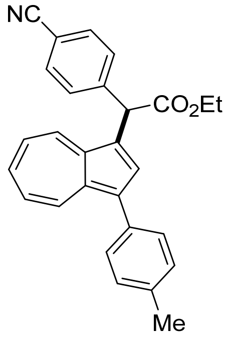

4q

Ethyl 2-(4-cyanophenyl)-2-(3-(*p*-tolyl)azulen-1-yl)acetate (4q)

The compound **4q** was prepared according to the same procedure as that of the synthesis of **4d**. The crude residue was purified by Silica gel column chromatography to give **4q** (72.1 mg, 91%) as a blue solid. m.p. 62−64 °C; R_f = 0.5 (EtOAc:hexane = 1:4); ^{1}H NMR (400 MHz, $CDCl_3$) δ 8.53 (d, J = 9.4 Hz, 1H), 8.22 (d, J = 9.5 Hz, 1H), 8.03 (s, 1H), 7.61−7.56 (m, 3H), 7.50−7.47 (m, 4H), 7.30 (d, J = 7.8 Hz, 2H), 7.13 (t, J = 9.6 Hz, 1H), 7.11 (t, J = 9.5 Hz, 1H), 5.72 (s, 1H), 4.30−4.19 (m, 2H), 2.43 (s, 3H), 1.26 (t, J = 7.1 Hz, 3H); ^{13}C{^{1}H} NMR (100 MHz, $CDCl_3$) δ 171.9, 144.7, 138.9, 137.1, 136.9, 136.4, 136.3, 136.2, 133.9, 133.5, 132.4, 130.6, 129.7, 129.5, 129.3, 123.9, 123.5, 122.9, 118.8, 111.0, 61.7, 50.1, 21.2, 14.2; IR (neat) 2979, 2921, 2227, 1732, 1572, 1572, 1505, 1368, 1308, 1156, 1030 cm^{-1}; HRMS (EI) *m/z*: [M$^+$] Calcd for $C_{28}H_{23}NO_2$ 405.1729; Found 405.1728.

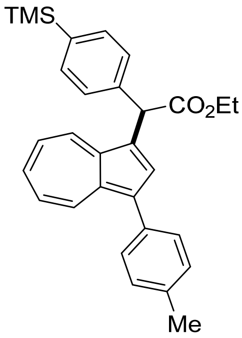

4r

Ethyl 2-(3-(*p*-tolyl)azulen-1-yl)-2-(4-(trimethylsilyl)phenyl)acetate (4r)

The compound **4r** was prepared according to the same procedure as that of the synthesis of **4d** The crude residue was purified by Silica gel column chromatography to give **4r** (68.2 mg, 75%) as a blue solid. m.p. 62−64 °C; R_f = 0.3 (EtOAc:hexane = 1:20); ^{1}H NMR (400 MHz, $CDCl_3$) δ 8.48 (d, J = 9.6 Hz, 1H), 8.30 (d, J = 9.6 Hz, 1H), 8.11 (s, 1H), 7.52 (t, J = 9.9 Hz, 1H), 7.49 (d, J = 8.0 Hz, 2H), 7.46 (d, J = 8.2

Hz, 2H), 7.39 (d, J = 7.8 Hz, 2H), 7.28 (d, J = 7.8 Hz, 2H), 7.07 (t, J = 9.7 Hz, 1H), 7.06 (t, J = 9.6 Hz, 1H), 5.68 (s, 1H), 4.22 (q, J = 7.1 Hz, 2H), 2.42 (s, 3H), 1.26 (t, J = 7.1 Hz, 3H), 0.22 (s, 9H); $^{13}C\{^1H\}$ NMR (100 MHz, $CDCl_3$) δ 174.0, 141.0, 140.1, 139.6, 138.5, 138.2, 137.2, 137.0, 135.3, 134.73, 134.70, 131.4, 130.5, 128.9, 126.3, 124.5, 123.7, 62.4, 51.2, 22.3, 15.4, 0.004; IR (neat) 3019, 2954, 1735, 1572, 1506, 1367, 1248, 1152, 1110, 1032, 839, 741 cm^{-1}; HRMS (EI) *m/z*: [M^+] Calcd for $C_{30}H_{32}O_2Si$ 452.2172; Found 452.2170.

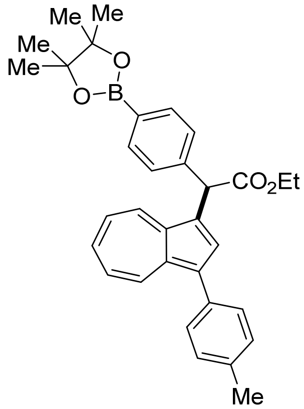

4s

Ethyl 2-(4-(4,4,5,5-tetramethyl-1,3,2-dioxaborolan-2-yl)phenyl)-2-(3-(*p*-tolyl)azulen-1-yl)acetate (4s)

The compound **4s** was prepared according to the same procedure as that of the synthesis of **4d**. The crude residue was purified by Silica gel column chromatography to give **4s** (50.1 mg, 50%) as a blue oil. R_f = 0.2 (EtOAc:hexane = 1:10); ^1H NMR (400 MHz, $CDCl_3$) δ 8.49 (d, J = 9.5 Hz, 1H), 8.26 (d, J = 9.6 Hz, 1H), 8.04 (s, 1H), 7.75 (d, J = 8.1 Hz, 2H), 7.54 (t, J = 9.7 Hz, 1H), 7.47 (d, J = 8.0 Hz, 2H), 7.40 (d, J = 8.0 Hz, 2H), 7.28 (d, J = 7.8 Hz, 2H), 7.07 (t, J = 10.1 Hz, 2H), 5.69 (s, 1H), 4.22 (q, J = 7.1 Hz, 2H), 2.42 (s, 3H), 1.31 (s, 12H), 1.25 (t, J = 7.0 Hz, 3H); $^{13}C\{^1H\}$ NMR (100 MHz, $CDCl_3$) δ 172.6, 142.43, 140.2, 138.4, 137.4, 137.1, 136.0, 135.9, 135.0, 134.1, 133.5, 130.2, 129.7, 129.3, 127.8, 123.4, 122.6, 83.7, 61.2, 50.4, 24.8, 21.2, 14.2; IR (neat) 3025, 2948, 1730, 1610, 1427, 1320, 1160, 1043, 861, 740 cm^{-1}; HRMS (EI) *m/z*: [M^+] Calcd for $C_{33}H_{35}BO_4$ 506.2628; Found 506.2631.

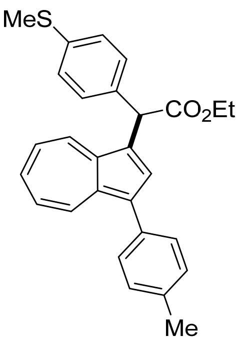

4t

Ethyl 2-(4-(methylthio)phenyl)-2-(3-(*p*-tolyl)azulen-1-yl)acetate (4t)

The compound **4t** was prepared according to the same procedure as that of the synthesis of **4d**. The crude residue was purified by Silica gel column chromatography to give **4t** (63.1 mg, 74%) as a blue oil. R_f = 0.2 (EtOAc:hexane = 1:20); ^{1}H NMR (400 MHz, $CDCl_3$) δ 8.49 (d, J = 9.7 Hz, 1H), 8.25 (d, J = 9.7 Hz, 1H), 8.07 (s, 1H), 7.53 (t, J = 9.8 Hz, 1H), 7.49 (d, J = 8.0 Hz, 2H), 7.31 (d, J = 8.4Hz, 2H), 7.29 (d, J = 8.7 Hz, 2H), 7.19 (d, J = 8.4 Hz, 2H), 7.07 (t J = 9.8 Hz, 2H), 5.64 (s, 1H), 4.22 (q, J = 7.1 Hz, 2H), 2.43 (s, 3H), 2.42 (s, 3H), 1.25 (t, J = 7.1 Hz, 3H); ^{13}C{^{1}H} NMR (100 MHz, $CDCl_3$) δ 172.8, 138.5, 137.25, 137.20, 137.1, 136.3, 136.1, 136.0, 134.2, 133.6, 130.3, 129.7, 129.4, 128.9, 126.8, 125.1, 123.5, 122.7, 61.3, 49.7, 21.2, 15.9, 14.3; IR (neat) 3020, 2979, 2919, 1732, 1572, 1493, 1428, 1367, 1313, 1153 cm^{-1}; HRMS (EI) *m/z*: [M$^+$] Calcd for $C_{28}H_{26}O_2S$ 426.1654; Found 426.1655.

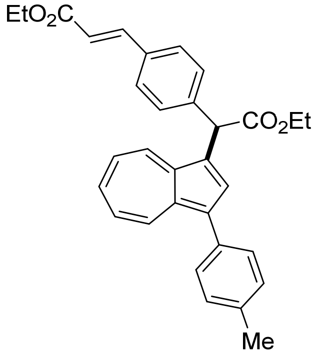

4u

Ethyl 3-(4-(2-ethoxy-2-oxo-1-(3-(*p*-tolyl)azulen-1-yl)ethyl)phenyl)acrylate (4u)

The compound **4u** was prepared according to the same procedure as that of the synthesis of **4a**. The crude residue was purified by Silica gel column chromatography to give **4u** (74.5 mg, 74%) as a blue oil. R_f = 0.4 (EtOAc:hexane = 1:10); ^{1}H NMR (400 MHz, $CDCl_3$) δ 8.50 (d, J = 9.6 Hz, 1H), 8.26 (d, J = 9.6 Hz, 1H), 8.07 (s, 1H), 7.64 (d, J = 16.0 Hz, 1H), 7.56 (t, J = 9.8 Hz, 1H), 7.50-7.45 (m, 4H), 7.40 (d, J =

8.3 Hz, 2H), 7.29 (d, J = 7.9 Hz, 2H), 7.08 (td, J = 14.8, 1.2 Hz, 2H), 6.38 (d, J = 16.0 Hz, 1H), 5.69 (s, 1H), 4.27-4.22 (m, 4H), 2.43 (s, 3H), 1.32 (t, J = 7.1 Hz, 3H), 1.26 (t, J = 7.1 Hz, 3H); $^{13}C\{^1H\}$ NMR (100 MHz, $CDCl_3$) δ 173.5, 167.0, 144.1, 141.6, 138.6, 137.1, 137.0, 136.2, 136.12, 136.09, 134.0, 133.5, 133.3, 130.3, 129.6, 129.3, 128.9, 128.2, 124.5, 123.5, 122.7, 118.1, 61.4, 60.5, 50.0, 21.2, 14.3, 14.2; IR (neat) 3027, 2947, 2846, 1734, 1598, 1428, 1151, 831, 727 cm^{-1}; HRMS (EI) *m/z*: [M^+] Calcd for $C_{32}H_{30}O_4$ 478.2144; Found 478.2146.

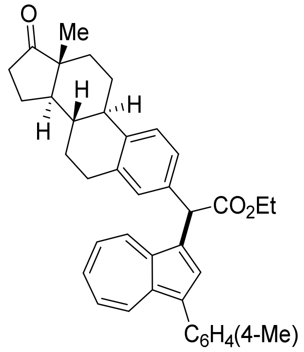

4v

Ethyl 2-((8*R*,9*S*,13*S*,14*S*)-13-methyl-17-oxo-7,8,9,11,12,13,14,15,16,17-decahydro-6*H*-cyclopenta[a]-phenanthren-3-yl)-2-(3-(*p*-tolyl)azulen-1-yl)acetate (4v)

The compound **4v** was prepared according to the same procedure as that of the synthesis of **4a**. The crude residue was purified by Silica gel column chromatography to give **4v** (44.7 mg, 48%) as a blue solid. m.p. 166−168 °C; R_f = 0.2 (EtOAc:hexane = 1:20); ^1H NMR (400 MHz, $CDCl_3$) δ 8.49 (d, J = 9.8 Hz, 1H), 8.31 (d, J = 9.7 Hz, 9.7 Hz, 1H), 8.10 (d, J = 1.4 Hz, 1H), 7.55 (t, J = 9.8 Hz, 1H), 7.50 (d, J = 8.0 Hz, 2H), 7.29 (d, J = 7.8 Hz, 2H), 7.24−7.18 (m, 3H), 5.63 (s, 1H), 4.22 (q, J = 7.1 Hz, 2H), 2.86 (dd, J = 3.4 Hz, 8.1 Hz, 2H), 2.49 (dd, J = 18.9 Hz, 8.5 Hz, 1H), 2.43 (s, 3H), 2.42−2.36 (m ,1H), 2.29−2.24 (m, 1H), 2.17−1.93 (m, 4H); $^{13}C\{^1H\}$ NMR (100 MHz, $CDCl_3$) δ 173.0, 138.6, 138.4, 137.4, 137.1, 136.8, 136.7, 136.11, 136.10, 135.9, 134.2, 133.5, 130.2, 129.7, 129.3, 128.9, 125.8, 125.6, 125.4, 123.3, 122.6, 61.2, 50.5, 49.6, 48.0, 44.3, 38.0, 35.9, 31.6, 29.5, 26.5, 25.6, 21.6, 21.2, 14.3, 13.8; IR (neat) 2981, 2931, 1734, 1644, 1369, 1154 cm^{-1}; HRMS (EI) *m/z*: [M^+] Calcd for $C_{39}H_{40}O_3$ 556.2977; Found 556.2979.

D. X-ray crystallographic data of 3o, 3y, and 4g

3o (19.4 mg) was dissolved in CH_2Cl_2 (1.0 mL) in a 4 mL glass vial and hexane (2 mL) was slowly added to form a separate layer. The 4 mL vial was open cap. Vapor diffusion afforded crystals of the composition **3o** suitable for Xray diffraction within 2 day at room temperature. A block-like specimen of $C_{25}H_{24}O_4$, approximate dimensions 0.360 mm x 0.240 mm x 0.100 mm, was used for the X-ray crystallographic analysis. The X-ray intensity data were measured. (ellipsoid = 40%, CCDC = 1435091).

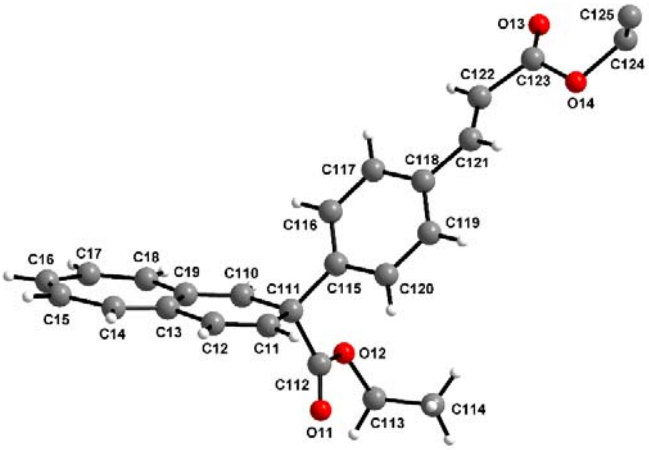

Supplementary Table 5. Crystal data and structure refinement for 3o.

Empirical formula	$C_{25}H_{19}O_4$	
Formula weight	383.40	
Temperature	296(2) K	
Wavelength	0.71073 Å	
Crystal system	Triclinic	
Space group	P -1	
Unit cell dimensions	a = 8.3196(4) Å	α= 89.980(3) °.
	b = 11.5152(6) Å	β= 89.980(3)°.
	c = 44.227(2) Å	γ = 89.990(3)°.
Volume	4237.0(4) $Å^3$	
Z	8	
Density (calculated)	1.202 Mg/m^3	
Absorption coefficient	0.081 mm^{-1}	
F(000)	1608	
Crystal size	0.360 x 0.240 x 0.100 mm^3	
θ range for data collection	1.381 to 28.271°.	

Index ranges	$-11 \leq h \leq 11$, $-15 \leq k \leq 15$, $-58 \leq l \leq 58$
Reflections collected	89703
Independent reflections	20228 [R(int) = 0.2026]
Completeness to theta = 25.242°	98.8 %
Absorption correction	multi-scan
Refinement method	Full-matrix least-squares on F^2
Data / restraints / parameters	20228 / 35 / 1034
Goodness-of-fit on F^2	0.938
Final R indices [I>2sigma(I)]	$R_1 = 0.1238$, $wR_2 = 0.2949$
R indices (all data)	$R_1 = 0.3406$, $wR_2 = 0.3639$
Extinction coefficient	0.026(2)
Largest diff. peak and hole	0.587 and -0.426 e·Å^{-3}

Supplementary Table 6. Atomic coordinates (x 10^4) and equivalent isotropic displacement parameters (Å^{2}x 10^3) for 3o. U(eq) is defined as one third of the trace of the orthogonalized U^{ij} tensor.

	x	y	z	U(eq)
C(11)	-795(6)	8527(5)	6646(1)	61(2)
C(12)	-1044(6)	7986(5)	6907(1)	58(2)
C(13)	-403(6)	8337(4)	7193(1)	55(2)
C(14)	-778(6)	7681(5)	7438(1)	58(2)
C(15)	-347(8)	7820(6)	7753(2)	76(2)
C(16)	495(7)	8652(6)	7893(2)	71(2)
C(17)	1238(7)	9628(5)	7747(1)	64(2)
C(18)	1279(6)	9948(5)	7452(1)	60(2)
C(19)	569(5)	9429(4)	7188(1)	45(1)
C(110)	843(6)	9974(4)	6920(1)	53(1)
C(111)	215(6)	9596(4)	6613(1)	52(1)
C(112)	-854(7)	10596(5)	6502(1)	54(1)
C(113)	-834(7)	12579(5)	6332(2)	75(2)
C(114)	-1035(10)	12500(7)	6003(2)	124(3)
C(115)	1545(6)	9403(4)	6381(1)	51(1)
C(116)	3135(6)	9438(4)	6449(1)	56(2)
C(117)	4307(7)	9267(5)	6227(2)	64(2)
C(118)	3882(8)	9045(5)	5931(2)	71(2)
C(119)	2300(8)	8989(6)	5860(1)	79(2)
C(120)	1117(7)	9167(5)	6082(1)	71(2)
C(121)	5092(9)	8905(6)	5687(2)	88(2)
C(122)	6506(11)	9197(8)	5688(2)	121(3)
C(123)	7629(8)	9106(8)	5413(2)	125(3)

C(124)	8080(30)	8748(14)	4891(6)	155(8)
C(126)	8470(20)	8110(20)	4985(4)	160(8)
C(125)	8106(17)	7475(12)	4785(4)	99(4)
C(127)	7640(40)	8720(30)	4736(7)	249(19)
C(21)	5789(7)	3520(5)	3354(1)	65(2)
C(22)	6046(6)	2976(5)	3093(1)	59(2)
C(23)	5401(6)	3345(4)	2801(1)	52(1)
C(24)	5759(6)	2701(5)	2559(1)	63(2)
C(25)	5365(7)	2810(5)	2243(2)	73(2)
C(26)	4500(7)	3656(6)	2110(1)	70(2)
C(27)	3768(6)	4639(5)	2253(1)	65(2)
C(28)	3740(6)	4953(5)	2546(1)	55(1)
C(29)	4415(6)	4421(4)	2815(1)	50(1)
C(210)	4150(6)	4967(4)	3082(1)	52(1)
C(211)	4797(6)	4612(4)	3385(1)	50(1)
C(212)	5850(7)	5588(5)	3503(1)	55(1)
C(213)	5830(8)	7581(5)	3669(2)	75(2)
C(214)	6059(10)	7499(6)	3996(2)	114(3)
C(215)	3472(6)	4398(4)	3621(1)	48(1)
C(216)	1866(7)	4432(4)	3555(1)	60(2)
C(217)	675(6)	4280(5)	3772(2)	60(2)
C(218)	1111(7)	4042(5)	4071(2)	67(2)
C(219)	2719(8)	3972(6)	4139(2)	81(2)
C(220)	3892(7)	4164(5)	3915(1)	72(2)
C(221)	-96(8)	3915(6)	4320(2)	89(2)
C(222)	-1507(10)	4213(7)	4314(2)	109(3)
C(223)	-2630(9)	4117(8)	4586(2)	121(3)
C(224)	-2680(20)	3967(16)	5173(2)	135(7)
C(226)	-3322(14)	3525(13)	5029(3)	110(5)
C(225)	-3030(40)	2683(17)	5244(6)	171(12)
C(227)	-3240(40)	2333(16)	5153(7)	185(13)
C(31)	4169(6)	9976(4)	1918(1)	53(1)
C(32)	4416(6)	9416(4)	2183(1)	46(1)
C(33)	3738(6)	9946(5)	2456(1)	58(1)
C(34)	3766(7)	9640(5)	2748(1)	66(2)
C(35)	4507(6)	8650(6)	2892(1)	66(2)
C(36)	5356(7)	7818(5)	2757(2)	73(2)
C(37)	5754(6)	7705(5)	2442(2)	69(2)
C(38)	5408(6)	8356(5)	2194(1)	57(1)
C(39)	6025(6)	7981(5)	1905(1)	61(2)
C(310)	5812(6)	8518(5)	1650(1)	62(2)
C(311)	4797(6)	9604(4)	1615(1)	47(1)
C(312)	5878(7)	10593(5)	1497(1)	52(1)
C(313)	5857(8)	12584(5)	1336(2)	77(2)
C(314)	6051(10)	12511(7)	1004(2)	120(3)
C(315)	3470(6)	9392(4)	1381(1)	51(1)
C(316)	3896(7)	9154(5)	1079(1)	72(2)
C(317)	2734(8)	8977(5)	868(1)	74(2)
C(318)	1122(8)	9038(5)	933(2)	67(2)
C(319)	682(6)	9277(4)	1230(2)	61(2)
C(320)	1846(6)	9442(4)	1445(1)	55(1)
C(321)	-73(9)	8919(6)	674(2)	104(3)
C(322)	-1507(11)	9210(8)	674(2)	120(3)
C(323)	-2646(9)	9100(8)	422(2)	123(3)
C(324)	-2760(30)	8974(19)	-162(3)	168(9)
C(326)	-3308(14)	8453(14)	-35(3)	104(5)

C(325)	-3070(40)	7687(19)	-235(7)	180(12)
C(327)	-3210(40)	7244(17)	-148(8)	211(14)
C(41)	9152(6)	5024(4)	1920(1)	51(1)
C(42)	9420(6)	5579(4)	2189(1)	47(1)
C(43)	8733(6)	5059(5)	2457(1)	58(2)
C(44)	8785(6)	5373(5)	2748(1)	58(2)
C(45)	9492(7)	6347(6)	2888(1)	66(2)
C(46)	10344(7)	7183(6)	2750(2)	72(2)
C(47)	10756(6)	7314(5)	2439(2)	60(2)
C(48)	10400(6)	6655(5)	2197(1)	54(1)
C(49)	11035(6)	7013(5)	1909(1)	61(2)
C(410)	10820(6)	6484(5)	1651(1)	61(2)
C(411)	9792(6)	5407(4)	1613(1)	51(1)
C(412)	10864(8)	4407(5)	1502(1)	60(2)
C(413)	10858(8)	2429(5)	1336(2)	77(2)
C(414)	11039(10)	2499(7)	1002(2)	124(3)
C(415)	8458(6)	5608(4)	1382(1)	52(1)
C(416)	8886(7)	5851(5)	1075(2)	80(2)
C(417)	7725(8)	6009(5)	864(1)	74(2)
C(418)	6123(8)	5967(5)	929(2)	68(2)
C(419)	5697(7)	5734(5)	1230(2)	66(2)
C(420)	6862(6)	5548(4)	1445(1)	56(1)
C(421)	4904(9)	6090(6)	679(2)	97(2)
C(422)	3490(11)	5795(7)	674(2)	119(3)
C(423)	2368(9)	5894(8)	424(2)	118(3)
C(424)	2340(30)	6020(20)	-170(3)	178(9)
C(426)	1779(16)	6594(14)	-45(3)	123(6)
C(425)	1840(40)	7270(20)	-245(6)	191(12)
C(427)	1950(30)	7771(16)	-176(6)	166(10)
O(11)	-2269(5)	10538(4)	6461(1)	86(1)
O(12)	-12(4)	11563(3)	6448(1)	65(1)
O(13)	8935(10)	9632(9)	5442(2)	94(3)
O(13*)	9073(11)	8813(15)	5423(4)	180(6)
O(14)	6867(13)	8938(12)	5150(2)	112(4)
O(14*)	7143(15)	8324(10)	5202(3)	122(4)
O(21)	7264(5)	5527(4)	3540(1)	85(1)
O(22)	5014(4)	6546(3)	3550(1)	62(1)
O(23)	-3908(10)	4679(10)	4557(2)	104(3)
O(23*)	-4109(10)	3950(15)	4568(3)	159(5)
O(24)	-1922(10)	3958(12)	4855(2)	118(4)
O(24*)	-2108(13)	3320(9)	4790(2)	120(4)
O(31)	7262(5)	10538(4)	1464(1)	86(1)
O(32)	4995(4)	11540(3)	1453(1)	64(1)
O(33)	-3884(11)	9709(10)	448(2)	106(3)
O(33*)	-4134(10)	8987(14)	441(3)	141(5)
O(34)	-1965(10)	8897(11)	152(2)	109(4)
O(34*)	-2154(13)	8297(10)	216(2)	124(4)
O(41)	12264(5)	4459(4)	1460(1)	86(1)
O(42)	10002(4)	3438(3)	1448(1)	62(1)
O(43)	891(10)	6068(15)	442(3)	156(5)
O(43*)	1100(11)	5314(9)	444(2)	100(3)
O(44)	3034(10)	6087(11)	153(2)	108(4)
O(44*)	2854(14)	6703(10)	220(2)	132(5)

Supplementary Table 7. Bond lengths [Å] and angles [°] for 3o.

C(11)-C(12)	1.328(7)
C(11)-C(111)	1.498(7)
C(12)-C(13)	1.432(7)
C(13)-C(14)	1.355(7)
C(13)-C(19)	1.494(7)
C(14)-C(15)	1.447(8)
C(15)-C(16)	1.339(8)
C(16)-C(17)	1.434(8)
C(17)-C(18)	1.357(7)
C(18)-C(19)	1.441(7)
C(19)-C(110)	1.358(7)
C(110)-C(111)	1.521(7)
C(111)-C(115)	1.524(7)
C(111)-C(112)	1.536(7)
C(112)-O(11)	1.193(6)
C(112)-O(12)	1.336(6)
C(113)-O(12)	1.451(6)
C(113)-C(114)	1.466(9)
C(115)-C(116)	1.357(7)
C(115)-C(120)	1.398(7)
C(116)-C(117)	1.397(7)
C(117)-C(118)	1.383(8)
C(118)-C(119)	1.354(8)
C(118)-C(121)	1.483(9)
C(119)-C(120)	1.406(7)
C(121)-C(122)	1.224(9)
C(122)-C(123)	1.535(10)
C(123)-O(13*)	1.249(2)
C(123)-O(13)	1.251(2)
C(123)-O(14)	1.341(2)
C(123)-O(14*)	1.358(2)
C(124)-C(127)	0.78(3)
C(124)-C(126)	0.91(3)
C(124)-C(125)	1.541(2)
C(124)-O(14)	1.541(2)
C(124)-O(14*)	1.66(2)
C(126)-C(125)	1.19(2)
C(126)-C(127)	1.480(2)
C(126)-O(14*)	1.482(2)
C(126)-O(14)	1.79(2)
C(125)-C(127)	1.50(3)
C(21)-C(22)	1.329(7)
C(21)-C(211)	1.511(7)
C(22)-C(23)	1.462(8)
C(23)-C(24)	1.336(7)
C(23)-C(29)	1.487(7)
C(24)-C(25)	1.442(8)
C(25)-C(26)	1.346(8)
C(26)-C(27)	1.432(8)
C(27)-C(28)	1.349(7)
C(28)-C(29)	1.448(7)
C(29)-C(210)	1.358(7)
C(210)-C(211)	1.501(7)

C(211)-C(212)	1.516(7)
C(211)-C(215)	1.537(7)
C(212)-O(21)	1.190(6)
C(212)-O(22)	1.321(6)
C(213)-C(214)	1.461(8)
C(213)-O(22)	1.468(6)
C(215)-C(216)	1.369(7)
C(215)-C(220)	1.371(7)
C(216)-C(217)	1.393(7)
C(217)-C(218)	1.399(8)
C(218)-C(219)	1.372(8)
C(218)-C(221)	1.495(8)
C(219)-C(220)	1.407(8)
C(221)-C(222)	1.224(9)
C(222)-C(223)	1.529(9)
C(223)-O(23*)	1.248(2)
C(223)-O(23)	1.252(2)
C(223)-O(24)	1.342(2)
C(223)-O(24*)	1.358(2)
C(224)-C(226)	0.98(2)
C(224)-C(225)	1.539(2)
C(224)-O(24)	1.541(2)
C(224)-O(24*)	1.913(12)
C(224)-C(227)	1.94(3)
C(226)-C(225)	1.38(3)
C(226)-C(227)	1.480(2)
C(226)-O(24*)	1.481(2)
C(226)-O(24)	1.482(2)
C(225)-C(227)	0.59(4)
C(31)-C(32)	1.356(7)
C(31)-C(311)	1.501(7)
C(32)-C(33)	1.465(7)
C(32)-C(38)	1.474(7)
C(33)-C(34)	1.336(7)
C(34)-C(35)	1.445(8)
C(35)-C(36)	1.332(8)
C(36)-C(37)	1.436(8)
C(37)-C(38)	1.359(8)
C(38)-C(39)	1.443(8)
C(39)-C(310)	1.301(7)
C(310)-C(311)	1.518(7)
C(311)-C(315)	1.532(7)
C(311)-C(312)	1.541(7)
C(312)-O(31)	1.162(6)
C(312)-O(32)	1.330(6)
C(313)-C(314)	1.476(9)
C(313)-O(32)	1.494(6)
C(315)-C(320)	1.382(7)
C(315)-C(316)	1.407(7)
C(316)-C(317)	1.360(7)
C(317)-C(318)	1.373(8)
C(318)-C(319)	1.392(8)
C(318)-C(321)	1.523(10)
C(319)-C(320)	1.370(7)
C(321)-C(322)	1.239(9)
C(322)-C(323)	1.470(10)

C(323)-O(33*)	1.248(2)
C(323)-O(33)	1.252(2)
C(323)-O(34)	1.341(2)
C(323)-O(34*)	1.359(2)
C(324)-C(326)	0.94(2)
C(324)-C(325)	1.539(2)
C(324)-O(34)	1.541(2)
C(324)-O(34*)	1.912(12)
C(324)-C(327)	2.03(3)
C(326)-C(325)	1.26(3)
C(326)-C(327)	1.480(2)
C(326)-O(34*)	1.481(2)
C(326)-O(34)	1.482(2)
C(325)-C(327)	0.65(4)
C(41)-C(42)	1.369(7)
C(41)-C(411)	1.524(7)
C(42)-C(43)	1.443(7)
C(42)-C(48)	1.485(7)
C(43)-C(44)	1.336(7)
C(44)-C(45)	1.412(8)
C(45)-C(46)	1.343(8)
C(46)-C(47)	1.422(8)
C(47)-C(48)	1.346(7)
C(48)-C(49)	1.438(7)
C(49)-C(410)	1.307(7)
C(410)-C(411)	1.516(7)
C(411)-C(415)	1.525(7)
C(411)-C(412)	1.538(7)
C(412)-O(41)	1.180(6)
C(412)-O(42)	1.347(7)
C(413)-O(42)	1.451(6)
C(413)-C(414)	1.488(9)
C(415)-C(420)	1.358(7)
C(415)-C(416)	1.432(8)
C(416)-C(417)	1.357(8)
C(417)-C(418)	1.364(8)
C(418)-C(419)	1.406(8)
C(418)-C(421)	1.506(9)
C(419)-C(420)	1.372(7)
C(421)-C(422)	1.225(9)
C(422)-C(423)	1.451(10)
C(423)-O(43)	1.248(2)
C(423)-O(43*)	1.251(2)
C(423)-O(44)	1.341(2)
C(423)-O(44*)	1.358(2)
C(424)-C(426)	0.98(3)
C(424)-C(425)	1.539(2)
C(424)-O(44)	1.541(2)
C(426)-C(425)	1.18(3)
C(426)-C(427)	1.480(2)
C(426)-O(44*)	1.481(2)
C(426)-O(44)	1.483(2)
C(425)-C(427)	0.66(4)
O(13)-O(13*)	0.954(16)
O(14)-O(14*)	0.778(15)
O(23)-O(23*)	0.857(16)

O(24)-O(24*)	0.806(15)
O(33)-O(33*)	0.857(15)
O(34)-O(34*)	0.764(15)
O(43)-O(43*)	0.885(15)
O(44)-O(44*)	0.784(15)

C(12)-C(11)-C(111)	123.9(5)
C(11)-C(12)-C(13)	125.3(5)
C(14)-C(13)-C(12)	117.5(5)
C(14)-C(13)-C(19)	127.3(6)
C(12)-C(13)-C(19)	115.1(5)
C(13)-C(14)-C(15)	130.5(6)
C(16)-C(15)-C(14)	130.9(6)
C(15)-C(16)-C(17)	125.3(6)
C(18)-C(17)-C(16)	130.9(6)
C(17)-C(18)-C(19)	131.1(6)
C(110)-C(19)-C(18)	116.5(5)
C(110)-C(19)-C(13)	119.6(5)
C(18)-C(19)-C(13)	123.9(5)
C(19)-C(110)-C(111)	126.0(5)
C(11)-C(111)-C(110)	109.9(5)
C(11)-C(111)-C(115)	110.7(4)
C(110)-C(111)-C(115)	113.1(4)
C(11)-C(111)-C(112)	108.8(4)
C(110)-C(111)-C(112)	105.7(4)
C(115)-C(111)-C(112)	108.4(4)
O(11)-C(112)-O(12)	122.5(5)
O(11)-C(112)-C(111)	125.3(5)
O(12)-C(112)-C(111)	112.2(5)
O(12)-C(113)-C(114)	110.9(5)
C(116)-C(115)-C(120)	117.6(5)
C(116)-C(115)-C(111)	123.7(5)
C(120)-C(115)-C(111)	118.7(5)
C(115)-C(116)-C(117)	121.4(5)
C(118)-C(117)-C(116)	120.9(5)
C(119)-C(118)-C(117)	118.5(6)
C(119)-C(118)-C(121)	119.1(7)
C(117)-C(118)-C(121)	122.4(6)
C(118)-C(119)-C(120)	120.7(6)
C(115)-C(120)-C(119)	120.8(6)
C(122)-C(121)-C(118)	128.4(8)
C(121)-C(122)-C(123)	124.4(8)
O(13*)-C(123)-O(13)	44.9(8)
O(13*)-C(123)-O(14)	116.5(11)
O(13)-C(123)-O(14)	124.7(9)
O(13*)-C(123)-O(14*)	97.5(12)
O(13)-C(123)-O(14*)	130.5(9)
O(14)-C(123)-O(14*)	33.5(7)
O(13*)-C(123)-C(122)	125.2(10)
O(13)-C(123)-C(122)	114.5(8)
O(14)-C(123)-C(122)	114.2(8)
O(14*)-C(123)-C(122)	114.0(8)
C(127)-C(124)-C(126)	122(3)
C(127)-C(124)-C(125)	72(3)
C(126)-C(124)-C(125)	50.3(15)
C(127)-C(124)-O(14)	111(4)

C(126)-C(124)-O(14)	90.4(17)
C(125)-C(124)-O(14)	111.8(12)
C(127)-C(124)-O(14*)	120(3)
C(126)-C(124)-O(14*)	62.9(12)
C(125)-C(124)-O(14*)	88.9(12)
O(14)-C(124)-O(14*)	27.9(6)
C(124)-C(126)-C(125)	94(2)
C(124)-C(126)-C(127)	26.5(18)
C(125)-C(126)-C(127)	67.3(18)
C(124)-C(126)-O(14*)	84.0(15)
C(125)-C(126)-O(14*)	113.6(17)
C(127)-C(126)-O(14*)	93.2(18)
C(124)-C(126)-O(14)	59.2(11)
C(125)-C(126)-O(14)	116.3(15)
C(127)-C(126)-O(14)	72.7(17)
O(14*)-C(126)-O(14)	25.3(7)
C(126)-C(125)-C(127)	65.7(12)
C(126)-C(125)-C(124)	36.0(14)
C(127)-C(125)-C(124)	29.7(13)
C(124)-C(127)-C(126)	31.2(17)
C(124)-C(127)-C(125)	78(2)
C(126)-C(127)-C(125)	47.0(12)
C(22)-C(21)-C(211)	124.0(5)
C(21)-C(22)-C(23)	124.8(5)
C(24)-C(23)-C(22)	117.8(5)
C(24)-C(23)-C(29)	128.1(6)
C(22)-C(23)-C(29)	114.1(5)
C(23)-C(24)-C(25)	132.7(6)
C(26)-C(25)-C(24)	127.4(6)
C(25)-C(26)-C(27)	127.3(6)
C(28)-C(27)-C(26)	130.2(6)
C(27)-C(28)-C(29)	132.0(5)
C(210)-C(29)-C(28)	117.0(5)
C(210)-C(29)-C(23)	120.7(5)
C(28)-C(29)-C(23)	122.2(5)
C(29)-C(210)-C(211)	126.5(5)
C(210)-C(211)-C(21)	109.9(4)
C(210)-C(211)-C(212)	108.2(4)
C(21)-C(211)-C(212)	109.4(4)
C(210)-C(211)-C(215)	113.1(4)
C(21)-C(211)-C(215)	108.7(4)
C(212)-C(211)-C(215)	107.5(4)
O(21)-C(212)-O(22)	123.1(5)
O(21)-C(212)-C(211)	125.2(5)
O(22)-C(212)-C(211)	111.7(5)
C(214)-C(213)-O(22)	111.1(5)
C(216)-C(215)-C(220)	117.3(5)
C(216)-C(215)-C(211)	123.3(5)
C(220)-C(215)-C(211)	119.4(5)
C(215)-C(216)-C(217)	122.8(5)
C(216)-C(217)-C(218)	119.6(5)
C(219)-C(218)-C(217)	118.0(5)
C(219)-C(218)-C(221)	119.3(6)
C(217)-C(218)-C(221)	122.6(6)
C(218)-C(219)-C(220)	121.0(6)
C(215)-C(220)-C(219)	121.3(6)

C(222)-C(221)-C(218)	126.9(7)
C(221)-C(222)-C(223)	123.2(8)
O(23*)-C(223)-O(23)	40.1(8)
O(23*)-C(223)-O(24)	118.0(10)
O(23)-C(223)-O(24)	122.3(9)
O(23*)-C(223)-O(24*)	104.7(12)
O(23)-C(223)-O(24*)	133.6(9)
O(24)-C(223)-O(24*)	34.7(7)
O(23*)-C(223)-C(222)	124.3(10)
O(23)-C(223)-C(222)	113.6(8)
O(24)-C(223)-C(222)	116.2(7)
O(24*)-C(223)-C(222)	112.2(7)
C(226)-C(224)-C(225)	61.8(17)
C(226)-C(224)-O(24)	67.9(4)
C(225)-C(224)-O(24)	105.0(15)
C(226)-C(224)-O(24*)	49.7(5)
C(225)-C(224)-O(24*)	81.6(13)
O(24)-C(224)-O(24*)	24.0(5)
C(226)-C(224)-C(227)	48.2(14)
C(225)-C(224)-C(227)	14.6(15)
O(24)-C(224)-C(227)	92.8(11)
O(24*)-C(224)-C(227)	68.9(10)
C(224)-C(226)-C(225)	79.6(15)
C(224)-C(226)-C(227)	102(2)
C(225)-C(226)-C(227)	23.7(18)
C(224)-C(226)-O(24*)	100.2(9)
C(225)-C(226)-O(24*)	105.2(16)
C(227)-C(226)-O(24*)	94.8(15)
C(224)-C(226)-O(24)	74.5(5)
C(225)-C(226)-O(24)	117.2(16)
C(227)-C(226)-O(24)	117.8(16)
O(24*)-C(226)-O(24)	31.6(6)
C(227)-C(225)-C(226)	88(3)
C(227)-C(225)-C(224)	125(4)
C(226)-C(225)-C(224)	38.6(9)
C(225)-C(227)-C(226)	69(3)
C(225)-C(227)-C(224)	41(3)
C(226)-C(227)-C(224)	29.4(9)
C(32)-C(31)-C(311)	125.8(5)
C(31)-C(32)-C(33)	117.2(5)
C(31)-C(32)-C(38)	120.4(5)
C(33)-C(32)-C(38)	122.3(5)
C(34)-C(33)-C(32)	132.6(5)
C(33)-C(34)-C(35)	129.9(6)
C(36)-C(35)-C(34)	126.5(6)
C(35)-C(36)-C(37)	128.5(6)
C(38)-C(37)-C(36)	133.1(6)
C(37)-C(38)-C(39)	118.3(5)
C(37)-C(38)-C(32)	127.0(6)
C(39)-C(38)-C(32)	114.7(5)
C(310)-C(39)-C(38)	125.4(5)
C(39)-C(310)-C(311)	123.7(5)
C(31)-C(311)-C(310)	109.8(5)
C(31)-C(311)-C(315)	113.4(4)
C(310)-C(311)-C(315)	109.8(4)
C(31)-C(311)-C(312)	107.1(4)

C(310)-C(311)-C(312)	108.5(4)
C(315)-C(311)-C(312)	108.1(4)
O(31)-C(312)-O(32)	125.0(5)
O(31)-C(312)-C(311)	125.5(5)
O(32)-C(312)-C(311)	109.4(4)
C(314)-C(313)-O(32)	110.6(5)
C(320)-C(315)-C(316)	116.7(5)
C(320)-C(315)-C(311)	124.0(5)
C(316)-C(315)-C(311)	119.3(5)
C(317)-C(316)-C(315)	120.1(5)
C(316)-C(317)-C(318)	123.0(6)
C(317)-C(318)-C(319)	117.6(6)
C(317)-C(318)-C(321)	118.5(6)
C(319)-C(318)-C(321)	123.8(6)
C(320)-C(319)-C(318)	119.8(5)
C(319)-C(320)-C(315)	122.9(5)
C(322)-C(321)-C(318)	127.1(8)
C(321)-C(322)-C(323)	126.6(9)
O(33*)-C(323)-O(33)	40.1(7)
O(33*)-C(323)-O(34)	117.5(9)
O(33)-C(323)-O(34)	121.8(9)
O(33*)-C(323)-O(34*)	105.8(11)
O(33)-C(323)-O(34*)	133.7(9)
O(34)-C(323)-O(34*)	32.8(7)
O(33*)-C(323)-C(322)	126.6(9)
O(33)-C(323)-C(322)	114.3(8)
O(34)-C(323)-C(322)	114.8(7)
O(34*)-C(323)-C(322)	111.9(7)
C(326)-C(324)-C(325)	55.1(17)
C(326)-C(324)-O(34)	68.6(4)
C(325)-C(324)-O(34)	102.0(15)
C(326)-C(324)-O(34*)	49.2(5)
C(325)-C(324)-O(34*)	80.6(14)
O(34)-C(324)-O(34*)	22.4(5)
C(326)-C(324)-C(327)	42.6(15)
C(325)-C(324)-C(327)	14.0(17)
O(34)-C(324)-C(327)	89.8(12)
O(34*)-C(324)-C(327)	67.8(11)
C(324)-C(326)-C(325)	87.5(17)
C(324)-C(326)-C(327)	112(2)
C(325)-C(326)-C(327)	26(2)
C(324)-C(326)-O(34*)	102.3(10)
C(325)-C(326)-O(34*)	110.0(18)
C(327)-C(326)-O(34*)	95.9(16)
C(324)-C(326)-O(34)	75.4(6)
C(325)-C(326)-O(34)	121.0(17)
C(327)-C(326)-O(34)	118.1(17)
O(34*)-C(326)-O(34)	29.9(6)
C(327)-C(325)-C(326)	96(4)
C(327)-C(325)-C(324)	131(4)
C(326)-C(325)-C(324)	37.4(11)
C(325)-C(327)-C(326)	58(3)
C(325)-C(327)-C(324)	35(3)
C(326)-C(327)-C(324)	25.3(10)
C(42)-C(41)-C(411)	125.6(5)
C(41)-C(42)-C(43)	117.0(5)

C(41)-C(42)-C(48)	120.0(5)
C(43)-C(42)-C(48)	123.0(5)
C(44)-C(43)-C(42)	131.6(5)
C(43)-C(44)-C(45)	130.8(6)
C(46)-C(45)-C(44)	126.0(6)
C(45)-C(46)-C(47)	130.0(6)
C(48)-C(47)-C(46)	130.9(6)
C(47)-C(48)-C(49)	117.5(5)
C(47)-C(48)-C(42)	127.7(5)
C(49)-C(48)-C(42)	114.8(5)
C(410)-C(49)-C(48)	126.1(5)
C(49)-C(410)-C(411)	123.8(5)
C(410)-C(411)-C(41)	109.5(5)
C(410)-C(411)-C(415)	111.2(4)
C(41)-C(411)-C(415)	112.7(4)
C(410)-C(411)-C(412)	108.7(5)
C(41)-C(411)-C(412)	105.8(4)
C(415)-C(411)-C(412)	108.7(4)
O(41)-C(412)-O(42)	122.7(5)
O(41)-C(412)-C(411)	125.7(6)
O(42)-C(412)-C(411)	111.5(5)
O(42)-C(413)-C(414)	110.3(5)
C(420)-C(415)-C(416)	116.4(5)
C(420)-C(415)-C(411)	124.6(5)
C(416)-C(415)-C(411)	118.9(5)
C(417)-C(416)-C(415)	120.3(6)
C(416)-C(417)-C(418)	123.0(6)
C(417)-C(418)-C(419)	117.0(6)
C(417)-C(418)-C(421)	120.0(6)
C(419)-C(418)-C(421)	122.9(6)
C(420)-C(419)-C(418)	120.5(5)
C(415)-C(420)-C(419)	122.9(6)
C(422)-C(421)-C(418)	129.4(8)
C(421)-C(422)-C(423)	127.7(9)
O(43)-C(423)-O(43*)	41.5(8)
O(43)-C(423)-O(44)	116.0(10)
O(43*)-C(423)-O(44)	119.9(9)
O(43)-C(423)-O(44*)	103.0(12)
O(43*)-C(423)-O(44*)	131.5(9)
O(44)-C(423)-O(44*)	33.7(7)
O(43)-C(423)-C(422)	126.7(10)
O(43*)-C(423)-C(422)	116.6(8)
O(44)-C(423)-C(422)	115.4(7)
O(44*)-C(423)-C(422)	111.6(7)
C(426)-C(424)-C(425)	50.2(17)
C(426)-C(424)-O(44)	67.9(4)
C(425)-C(424)-O(44)	104.7(16)
C(424)-C(426)-C(425)	90.3(18)
C(424)-C(426)-C(427)	111(2)
C(425)-C(426)-C(427)	25.6(19)
C(424)-C(426)-O(44*)	102.5(10)
C(425)-C(426)-O(44*)	121(2)
C(427)-C(426)-O(44*)	100.1(14)
C(424)-C(426)-O(44)	74.4(6)
C(425)-C(426)-O(44)	132(2)
C(427)-C(426)-O(44)	121.6(15)

O(44*)-C(426)-O(44)	30.7(6)
C(427)-C(425)-C(426)	103(4)
C(427)-C(425)-C(424)	133(4)
C(426)-C(425)-C(424)	39.5(12)
C(425)-C(427)-C(426)	51(3)
C(112)-O(12)-C(113)	119.2(4)
O(13*)-O(13)-C(123)	67.4(4)
O(13)-O(13*)-C(123)	67.7(4)
O(14*)-O(14)-C(123)	74.5(4)
O(14*)-O(14)-C(124)	84.2(17)
C(123)-O(14)-C(124)	110.9(14)
O(14*)-O(14)-C(126)	54.4(13)
C(123)-O(14)-C(126)	94.5(10)
C(124)-O(14)-C(126)	30.4(11)
O(14)-O(14*)-C(123)	72.0(4)
O(14)-O(14*)-C(126)	100.4(18)
C(123)-O(14*)-C(126)	109.6(13)
O(14)-O(14*)-C(124)	67.9(14)
C(123)-O(14*)-C(124)	103.7(10)
C(126)-O(14*)-C(124)	33.1(10)
C(212)-O(22)-C(213)	119.4(4)
O(23*)-O(23)-C(223)	69.7(4)
O(23)-O(23*)-C(223)	70.2(4)
O(24*)-O(24)-C(223)	73.7(4)
O(24*)-O(24)-C(226)	74.2(4)
C(223)-O(24)-C(226)	99.2(8)
O(24*)-O(24)-C(224)	104.8(10)
C(223)-O(24)-C(224)	129.0(10)
C(226)-O(24)-C(224)	37.6(8)
O(24)-O(24*)-C(223)	71.5(4)
O(24)-O(24*)-C(226)	74.3(4)
C(223)-O(24*)-C(226)	98.5(8)
O(24)-O(24*)-C(224)	51.2(7)
C(223)-O(24*)-C(224)	104.2(9)
C(226)-O(24*)-C(224)	30.1(7)
C(312)-O(32)-C(313)	116.4(4)
O(33*)-O(33)-C(323)	69.7(4)
O(33)-O(33*)-C(323)	70.2(4)
O(34*)-O(34)-C(323)	74.9(4)
O(34*)-O(34)-C(326)	74.9(4)
C(323)-O(34)-C(326)	103.8(8)
O(34*)-O(34)-C(324)	107.3(10)
C(323)-O(34)-C(324)	127.5(11)
C(326)-O(34)-C(324)	36.0(10)
O(34)-O(34*)-C(323)	72.3(4)
O(34)-O(34*)-C(326)	75.2(4)
C(323)-O(34*)-C(326)	103.0(8)
O(34)-O(34*)-C(324)	50.3(8)
C(323)-O(34*)-C(324)	103.1(10)
C(326)-O(34*)-C(324)	28.6(8)
C(412)-O(42)-C(413)	117.5(4)
O(43*)-O(43)-C(423)	69.4(4)
O(43)-O(43*)-C(423)	69.1(4)
O(44*)-O(44)-C(423)	74.4(4)
O(44*)-O(44)-C(426)	74.6(4)
C(423)-O(44)-C(426)	107.6(9)

O(44*)-O(44)-C(424)	109.1(11)
C(423)-O(44)-C(424)	131.6(12)
C(426)-O(44)-C(424)	37.7(10)
O(44)-O(44*)-C(423)	71.9(4)
O(44)-O(44*)-C(426)	74.8(4)
C(423)-O(44*)-C(426)	106.7(9)

Symmetry transformations used to generate equivalent atoms:

Supplementary Table 8. Anisotropic displacement parameters ($Å^2 x 10^3$) for 3o. The anisotropic displacement factor exponent takes the form: $-2\pi^2[h^2 a^{*2}U^{11} + ... + 2 h k a^* b^* U^{12}]$

	U^{11}	U^{22}	U^{33}	U^{23}	U^{13}	U^{12}
C(11)	61(4)	53(4)	69(4)	-6(3)	4(3)	-15(3)
C(12)	65(4)	44(3)	66(4)	-2(3)	0(3)	-18(3)
C(13)	50(3)	41(3)	75(4)	2(3)	8(3)	2(3)
C(14)	55(3)	43(3)	75(5)	10(3)	14(3)	8(3)
C(15)	83(5)	70(5)	76(5)	33(4)	6(4)	-7(4)
C(16)	59(4)	78(5)	76(5)	26(4)	-1(3)	5(3)
C(17)	80(4)	66(4)	45(4)	1(3)	-10(3)	11(3)
C(18)	63(4)	51(4)	67(5)	-3(3)	-6(3)	-2(3)
C(19)	38(3)	35(3)	63(4)	-2(3)	4(3)	1(2)
C(110)	62(3)	45(3)	51(4)	-2(3)	-9(3)	-12(3)
C(111)	50(3)	46(3)	60(4)	2(3)	6(3)	7(3)
C(112)	47(4)	57(4)	58(4)	-5(3)	4(3)	-3(3)
C(113)	80(4)	50(4)	96(5)	-2(3)	-11(4)	19(3)
C(114)	161(8)	112(7)	100(7)	31(5)	-41(6)	16(6)
C(115)	51(4)	47(3)	54(4)	-1(3)	-2(3)	3(2)
C(116)	55(4)	43(3)	72(4)	7(3)	-3(3)	-2(3)
C(117)	46(3)	56(4)	92(5)	9(3)	0(3)	-7(3)
C(118)	59(4)	64(4)	90(5)	-3(4)	13(4)	-3(3)
C(119)	75(5)	100(5)	62(4)	-8(4)	9(4)	-1(4)
C(120)	59(4)	94(5)	61(4)	-10(3)	15(3)	-2(3)
C(121)	77(5)	105(6)	82(5)	5(4)	22(4)	9(4)
C(122)	102(6)	169(9)	92(6)	8(5)	38(5)	1(6)
C(123)	74(5)	185(9)	115(7)	33(6)	31(5)	-10(5)
C(21)	79(4)	54(4)	61(4)	15(3)	0(3)	13(3)
C(22)	61(3)	39(3)	78(5)	12(3)	3(3)	8(3)
C(23)	46(3)	40(3)	72(4)	0(3)	3(3)	-5(2)
C(24)	66(4)	50(4)	73(5)	-5(3)	11(3)	4(3)
C(25)	82(4)	60(4)	76(5)	-16(4)	9(4)	4(3)
C(26)	66(4)	80(5)	64(4)	-8(4)	-3(3)	-4(3)
C(27)	60(4)	72(4)	62(4)	4(3)	-11(3)	5(3)
C(28)	53(3)	58(4)	53(4)	5(3)	-1(3)	3(3)
C(29)	45(3)	39(3)	65(4)	4(3)	-5(3)	3(2)
C(210)	57(3)	45(3)	55(4)	2(3)	-10(3)	10(2)
C(211)	46(3)	49(3)	56(4)	-1(3)	1(3)	5(3)
C(212)	61(4)	56(4)	49(4)	10(3)	-6(3)	5(3)
C(213)	88(4)	53(4)	83(5)	-1(3)	-17(4)	-8(3)
C(214)	171(8)	97(6)	75(6)	-15(4)	-19(5)	-16(5)

C(215)	45(3)	45(3)	54(4)	9(3)	5(3)	1(2)
C(216)	68(4)	41(3)	71(4)	6(3)	-2(3)	-2(3)
C(217)	42(3)	56(4)	83(5)	-5(3)	8(3)	1(3)
C(218)	62(4)	60(4)	79(5)	4(3)	20(4)	-3(3)
C(219)	72(5)	103(5)	67(4)	23(4)	7(4)	3(4)
C(220)	74(4)	87(5)	57(4)	21(3)	4(3)	4(3)
C(221)	73(5)	104(6)	90(5)	9(4)	19(4)	11(4)
C(222)	95(6)	140(7)	93(6)	24(5)	22(5)	7(5)
C(223)	93(6)	167(9)	103(7)	-10(6)	39(5)	20(5)
C(31)	52(3)	41(3)	67(4)	-9(3)	5(3)	2(2)
C(32)	49(3)	33(3)	56(4)	1(3)	-2(3)	5(2)
C(33)	56(3)	55(4)	61(4)	3(3)	-4(3)	-3(3)
C(34)	74(4)	61(4)	63(4)	-3(3)	6(3)	1(3)
C(35)	54(4)	84(5)	61(4)	12(4)	-1(3)	-9(3)
C(36)	83(4)	57(4)	80(5)	27(4)	-5(4)	4(3)
C(37)	56(4)	57(4)	95(5)	9(4)	-17(4)	2(3)
C(38)	60(3)	45(3)	65(4)	7(3)	-7(3)	-5(3)
C(39)	66(4)	41(3)	77(5)	-12(3)	-11(3)	29(3)
C(310)	68(4)	60(4)	59(4)	-10(3)	-3(3)	17(3)
C(311)	47(3)	36(3)	59(4)	-8(3)	-1(3)	3(2)
C(312)	41(3)	64(4)	52(4)	6(3)	-4(3)	5(3)
C(313)	82(4)	51(4)	97(5)	13(3)	23(4)	-10(3)
C(314)	181(8)	100(6)	80(6)	34(4)	30(5)	-22(5)
C(315)	68(4)	34(3)	53(4)	-5(2)	3(3)	4(2)
C(316)	57(4)	80(5)	78(5)	-12(4)	-13(4)	6(3)
C(317)	76(5)	92(5)	53(4)	-2(3)	-9(4)	3(4)
C(318)	66(4)	66(4)	70(5)	-2(3)	-22(4)	0(3)
C(319)	45(3)	51(4)	86(5)	8(3)	-12(3)	1(3)
C(320)	48(3)	58(4)	59(4)	0(3)	5(3)	0(3)
C(321)	70(5)	81(5)	163(9)	-14(5)	2(5)	4(4)
C(322)	110(7)	133(8)	116(7)	8(5)	-19(6)	-1(6)
C(323)	104(6)	180(9)	86(6)	10(6)	-22(5)	32(6)
C(41)	51(3)	39(3)	63(4)	13(3)	1(3)	-6(2)
C(42)	49(3)	30(3)	61(4)	6(3)	-3(3)	-4(2)
C(43)	53(3)	45(3)	76(5)	4(3)	-2(3)	-4(2)
C(44)	64(4)	64(4)	46(4)	1(3)	17(3)	2(3)
C(45)	61(4)	86(5)	53(4)	-18(4)	0(3)	9(3)
C(46)	70(4)	63(5)	83(5)	-24(4)	-1(4)	3(3)
C(47)	48(3)	46(4)	87(5)	-10(3)	-5(3)	2(3)
C(48)	56(3)	42(3)	64(4)	8(3)	-3(3)	7(3)
C(49)	69(4)	53(4)	62(4)	9(3)	-8(3)	-22(3)
C(410)	63(4)	52(4)	68(4)	15(3)	0(3)	-13(3)
C(411)	48(3)	48(3)	59(4)	13(3)	-7(3)	-2(3)
C(412)	57(4)	73(5)	50(4)	3(3)	-7(3)	0(3)
C(413)	87(4)	56(4)	88(5)	0(3)	23(4)	14(3)
C(414)	169(8)	93(6)	110(7)	-25(5)	32(6)	11(5)
C(415)	50(3)	48(3)	58(4)	9(3)	4(3)	-2(2)
C(416)	57(4)	89(5)	94(5)	20(4)	-11(4)	1(3)
C(417)	74(5)	87(5)	60(4)	16(3)	-14(4)	0(3)
C(418)	77(5)	64(4)	64(5)	5(3)	-16(4)	4(3)
C(419)	45(3)	60(4)	94(5)	-7(3)	-12(4)	-2(3)
C(420)	53(4)	56(4)	59(4)	-1(3)	4(3)	0(3)
C(421)	70(5)	88(6)	133(7)	9(5)	-9(5)	-7(4)
C(422)	99(7)	133(8)	124(7)	9(5)	-33(6)	-9(5)
C(423)	83(6)	171(9)	101(7)	-12(6)	-17(5)	-26(5)
O(11)	47(3)	88(3)	121(4)	9(3)	-9(2)	-2(2)

O(12)	54(2)	50(3)	91(3)	9(2)	-9(2)	3(2)
O(21)	52(3)	92(3)	110(4)	-3(3)	-5(2)	0(2)
O(22)	58(2)	46(2)	80(3)	-4(2)	-13(2)	3(2)
O(31)	47(3)	90(3)	120(4)	16(3)	2(2)	3(2)
O(32)	60(2)	49(2)	82(3)	9(2)	12(2)	-2(2)
O(41)	51(3)	99(4)	107(4)	-10(3)	5(2)	-9(2)
O(42)	53(2)	52(3)	82(3)	-3(2)	13(2)	-1(2)

Supplementary Table 9. Hydrogen coordinates (x 10^4) and isotropic displacement parameters (Å^{2}x 10^3) for 3o.

H(11)	-1277	8223	6474	73
H(12)	-1686	7326	6904	70
H(14)	-1416	7038	7396	69
H(15)	-717	7233	7879	91
H(16)	609	8592	8101	86
H(17)	1783	10125	7877	76
H(18)	1866	10620	7414	73
H(110)	1473	10641	6926	63
H(11A)	-220	13269	6381	91
H(11B)	-1880	12646	6427	91
H(11C)	-11	12597	5906	186
H(11D)	-1757	13098	5936	186
H(11E)	-1470	11753	5951	186
H(116)	3451	9579	6647	68
H(117)	5388	9303	6280	77
H(119)	1993	8831	5662	95
H(120)	36	9127	6029	86
H(121)	4729	8549	5511	106
H(122)	6929	9493	5866	145
H(21)	6248	3209	3528	78
H(22)	6682	2313	3097	71
H(24)	6387	2054	2603	76
H(25)	5753	2230	2116	87
H(26)	4362	3595	1902	84
H(27)	3227	5139	2123	78
H(28)	3184	5639	2584	66
H(210)	3504	5626	3077	63
H(21A)	6867	7664	3571	90
H(21B)	5196	8265	3622	90
H(21C)	5033	7533	4095	171
H(21D)	6716	8132	4063	171
H(21E)	6576	6777	4044	171
H(216)	1558	4562	3355	72
H(217)	-404	4337	3719	72
H(219)	3038	3796	4335	97
H(220)	4973	4132	3967	87
H(221)	264	3573	4498	107
H(222)	-1917	4513	4134	131
H(31)	3555	10650	1924	64

H(33)	3178	10631	2419	69
H(34)	3225	10141	2877	79
H(35)	4378	8587	3100	79
H(36)	5742	7234	2883	88
H(37)	6374	7053	2399	83
H(39)	6624	7300	1902	74
H(310)	6312	8218	1478	75
H(31A)	5255	13279	1387	92
H(31B)	6906	12639	1430	92
H(31C)	6568	11792	953	180
H(31D)	6696	13148	935	180
H(31E)	5014	12543	910	180
H(316)	4974	9118	1024	86
H(317)	3046	8806	671	89
H(319)	-399	9324	1282	73
H(320)	1528	9595	1643	66
H(321)	315	8592	496	125
H(322)	-1904	9530	852	143
H(41)	8525	4355	1926	61
H(43)	8148	4386	2420	70
H(44)	8271	4864	2879	69
H(45)	9356	6417	3096	80
H(46)	10722	7767	2876	87
H(47)	11378	7965	2397	73
H(49)	11657	7684	1907	74
H(410)	11328	6785	1481	73
H(41A)	10272	1730	1389	92
H(41B)	11911	2388	1429	92
H(41C)	9997	2473	909	186
H(41D)	11671	1855	932	186
H(41E)	11565	3212	949	186
H(416)	9963	5901	1021	96
H(417)	8036	6153	665	89
H(419)	4619	5707	1285	79
H(420)	6543	5372	1641	67
H(421)	5279	6441	503	116
H(422)	3088	5469	851	142

3y (24.8 mg) was dissolved in CH_2Cl_2 (1.0 mL) in a 4 mL glass vial and hexane (2 mL) was slowly added to form a separate layer. The 4 mL vial was open cap. Vapor diffusion afforded crystals of the composition **3y** suitable for Xray diffraction within 2 day at room temperature. A block-like specimen of $C_{34}H_{24}O_4$, approximate dimensions 0.102 mm x 0.052 mm x 0.048 mm, was used for the X-ray crystallographic analysis. The X-ray intensity data were measured. (ellipsoid = 40%, CCDC = 1916356).

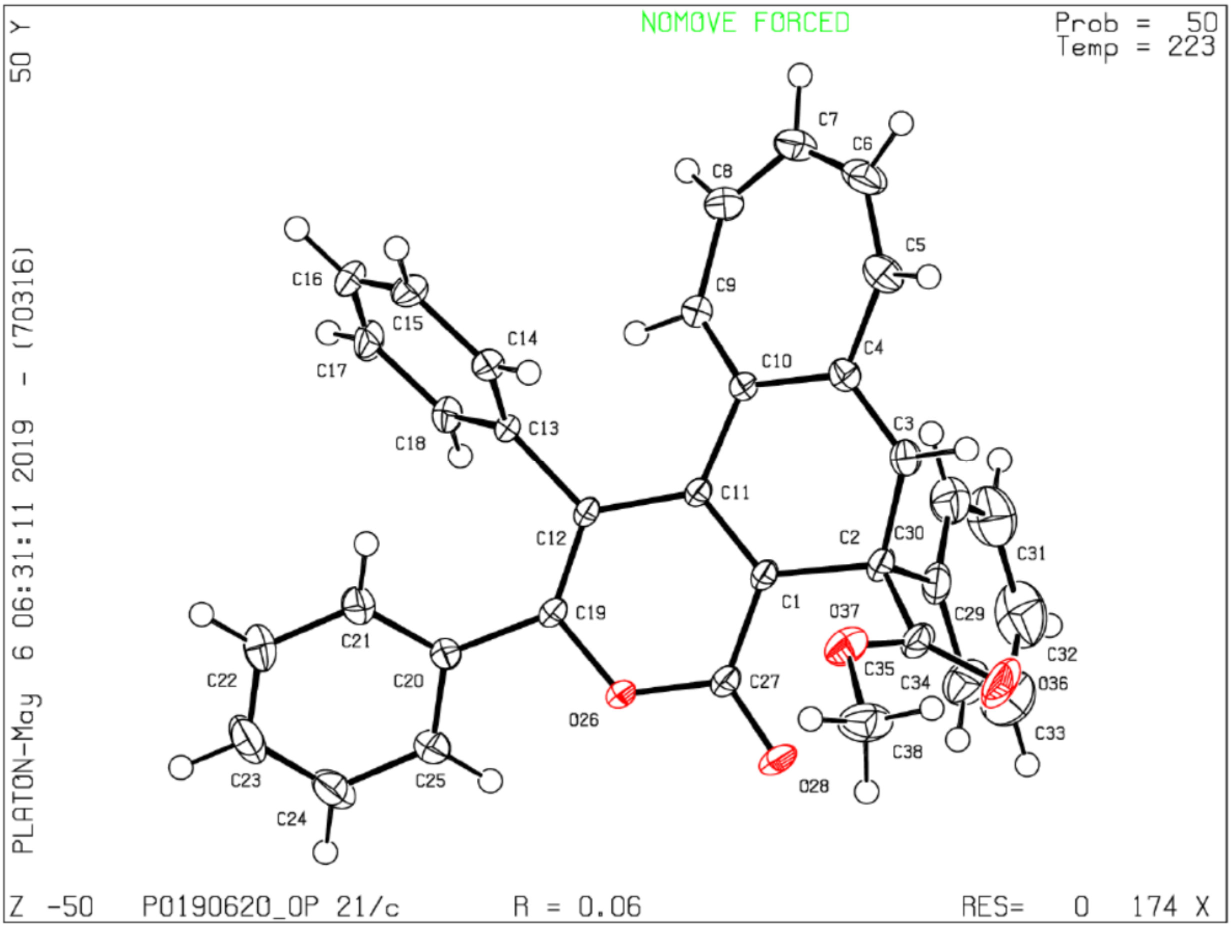

Supplementary Table 10. Crystal data and structure refinement for 3y.

Empirical formula	$C_{34}H_{24}O_4$	
Formula weight	496.53	
Temperature	223(2) K	
Wavelength	0.730 Å	
Crystal system	Monoclinic	
Space group	*P*2$_1$/*c*	
Unit cell dimensions	a = 6.0670(12) Å	α = 90°
	b = 22.895(5) Å	β = 90.85(3)°

	c = 17.994(4) Å	γ = 90°
Volume	2499.2(9) Å^3	
Z	4	
Density (calculated)	1.320 Mg/m^3	
Absorption coefficient	0.086 mm^{-1}	
F(000)	1040	
Crystal size	0.102 x 0.052 x 0.048 mm^3	
Theta range for data collection	1.478 to 29.998°.	
Index ranges	−8<=h<=8, −31<=k<=31, −24<=l<=24	
Reflections collected	23501	
Independent reflections	6717 [R(int) = 0.0799]	
Completeness to theta = 25.976°	99.9 %	
Absorption correction	Empirical	
Max. and min. transmission	1.000 and 0.925	
Refinement method	Full-matrix least-squares on F^2	
Data / restraints / parameters	6717 / 0 / 344	
Goodness-of-fit on F^2	1.132	
Final R indices [I>2sigma(I)]	R1 = 0.0614, wR2 = 0.1770	
R indices (all data)	R1 = 0.0708, wR2 = 0.1842	
Largest diff. peak and hole	0.568 and −0.308 e·Å^{-3}	

Supplementary Table 11. Atomic coordinates (x 10^4) and equivalent isotropic displacement parameters (Å^2 x 10^3) for 3y. U(eq) is defined as one third of the trace of the orthogonalized U^{ij} tensor.

	x	y	z	U(eq)
C(1)	6541(2)	7092(1)	2858(1)	17(1)
C(2)	7926(2)	7584(1)	3184(1)	20(1)
C(3)	9951(2)	7334(1)	3561(1)	24(1)
C(4)	10040(2)	6791(1)	3842(1)	20(1)
C(5)	12043(2)	6625(1)	4259(1)	27(1)
C(6)	12330(2)	6237(1)	4812(1)	31(1)
C(7)	10753(3)	5846(1)	5127(1)	33(1)
C(8)	8707(3)	5731(1)	4869(1)	30(1)
C(9)	7611(2)	5950(1)	4209(1)	22(1)

C(10)	8153(2)	6387(1)	3737(1)	17(1)
C(11)	6650(2)	6525(1)	3100(1)	16(1)
C(12)	5260(2)	6086(1)	2735(1)	16(1)
C(13)	5361(2)	5450(1)	2917(1)	17(1)
C(14)	7252(2)	5128(1)	2761(1)	22(1)
C(15)	7292(3)	4528(1)	2889(1)	32(1)
C(16)	5461(3)	4250(1)	3175(1)	36(1)
C(17)	3589(3)	4567(1)	3334(1)	31(1)
C(18)	3534(2)	5165(1)	3211(1)	23(1)
C(19)	3855(2)	6258(1)	2181(1)	18(1)
C(20)	2284(2)	5908(1)	1724(1)	19(1)
C(21)	2721(3)	5337(1)	1494(1)	27(1)
C(22)	1174(3)	5031(1)	1072(1)	36(1)
C(23)	-805(3)	5292(1)	867(1)	37(1)
C(24)	-1234(3)	5860(1)	1077(1)	32(1)
C(25)	286(2)	6168(1)	1507(1)	25(1)
O(26)	3711(2)	6832(1)	1968(1)	23(1)
C(27)	4984(2)	7262(1)	2279(1)	21(1)
O(28)	4714(2)	7753(1)	2040(1)	33(1)
C(29)	6520(2)	7936(1)	3743(1)	25(1)
C(30)	6419(3)	7753(1)	4478(1)	36(1)
C(31)	5030(4)	8028(1)	4977(1)	50(1)
C(32)	3745(4)	8492(1)	4751(1)	56(1)
C(33)	3827(4)	8676(1)	4025(1)	54(1)
C(34)	5201(3)	8404(1)	3521(1)	39(1)
C(35)	8810(2)	7969(1)	2551(1)	24(1)
O(36)	9128(2)	8486(1)	2590(1)	39(1)
O(37)	9332(2)	7636(1)	1970(1)	29(1)
C(38)	9977(3)	7942(1)	1308(1)	38(1)

Supplementary Table 12. Bond lengths [Å] and angles [°] for 3y.

C(1)-C(11)	1.3694(16)
C(1)-C(27)	1.4490(17)

C(1)-C(2)	1.5192(17)
C(2)-C(3)	1.5077(19)
C(2)-C(35)	1.5427(19)
C(2)-C(29)	1.553(2)
C(3)-C(4)	1.3428(18)
C(3)-H(3)	0.9400
C(4)-C(5)	1.4688(18)
C(4)-C(10)	1.4821(17)
C(5)-C(6)	1.344(2)
C(5)-H(5)	0.9400
C(6)-C(7)	1.433(2)
C(6)-H(6)	0.9400
C(7)-C(8)	1.345(2)
C(7)-H(7)	0.9400
C(8)-C(9)	1.4423(19)
C(8)-H(8)	0.9400
C(9)-C(10)	1.3553(18)
C(9)-H(9)	0.9400
C(10)-C(11)	1.4883(16)
C(11)-C(12)	1.4625(16)
C(12)-C(19)	1.3611(17)
C(12)-C(13)	1.4916(16)
C(13)-C(14)	1.3961(19)
C(13)-C(18)	1.3969(19)
C(14)-C(15)	1.3927(19)
C(14)-H(14)	0.9400
C(15)-C(16)	1.386(3)
C(15)-H(15)	0.9400
C(16)-C(17)	1.381(3)
C(16)-H(16)	0.9400
C(17)-C(18)	1.3877(19)
C(17)-H(17)	0.9400
C(18)-H(18)	0.9400
C(19)-O(26)	1.3698(14)
C(19)-C(20)	1.4836(17)
C(20)-C(21)	1.3985(19)

C(20)-C(25)	1.4007(19)
C(21)-C(22)	1.388(2)
C(21)-H(21)	0.9400
C(22)-C(23)	1.385(3)
C(22)-H(22)	0.9400
C(23)-C(24)	1.381(2)
C(23)-H(23)	0.9400
C(24)-C(25)	1.3881(19)
C(24)-H(24)	0.9400
C(25)-H(25)	0.9400
O(26)-C(27)	1.3677(15)
C(27)-O(28)	1.2122(15)
C(29)-C(30)	1.390(2)
C(29)-C(34)	1.393(2)
C(30)-C(31)	1.391(3)
C(30)-H(30)	0.9400
C(31)-C(32)	1.376(3)
C(31)-H(31)	0.9400
C(32)-C(33)	1.375(3)
C(32)-H(32)	0.9400
C(33)-C(34)	1.389(3)
C(33)-H(33)	0.9400
C(34)-H(34)	0.9400
C(35)-O(36)	1.2000(17)
C(35)-O(37)	1.3372(19)
O(37)-C(38)	1.4409(18)
C(38)-H(38A)	0.9700
C(38)-H(38B)	0.9700
C(38)-H(38C)	0.9700
C(11)-C(1)-C(27)	120.83(11)
C(11)-C(1)-C(2)	123.77(11)
C(27)-C(1)-C(2)	115.35(10)
C(3)-C(2)-C(1)	109.56(10)
C(3)-C(2)-C(35)	105.05(11)
C(1)-C(2)-C(35)	109.60(11)

C(3)-C(2)-C(29)	110.92(11)
C(1)-C(2)-C(29)	109.19(10)
C(35)-C(2)-C(29)	112.44(11)
C(4)-C(3)-C(2)	123.28(12)
C(4)-C(3)-H(3)	118.4
C(2)-C(3)-H(3)	118.4
C(3)-C(4)-C(5)	117.35(12)
C(3)-C(4)-C(10)	120.25(11)
C(5)-C(4)-C(10)	122.38(12)
C(6)-C(5)-C(4)	130.28(14)
C(6)-C(5)-H(5)	114.9
C(4)-C(5)-H(5)	114.9
C(5)-C(6)-C(7)	128.73(14)
C(5)-C(6)-H(6)	115.6
C(7)-C(6)-H(6)	115.6
C(8)-C(7)-C(6)	127.19(14)
C(8)-C(7)-H(7)	116.4
C(6)-C(7)-H(7)	116.4
C(7)-C(8)-C(9)	128.90(15)
C(7)-C(8)-H(8)	115.6
C(9)-C(8)-H(8)	115.6
C(10)-C(9)-C(8)	131.25(13)
C(10)-C(9)-H(9)	114.4
C(8)-C(9)-H(9)	114.4
C(9)-C(10)-C(4)	125.08(11)
C(9)-C(10)-C(11)	119.24(11)
C(4)-C(10)-C(11)	115.35(10)
C(1)-C(11)-C(12)	118.93(11)
C(1)-C(11)-C(10)	118.24(11)
C(12)-C(11)-C(10)	122.81(10)
C(19)-C(12)-C(11)	118.69(11)
C(19)-C(12)-C(13)	117.88(10)
C(11)-C(12)-C(13)	123.39(10)
C(14)-C(13)-C(18)	119.21(12)
C(14)-C(13)-C(12)	120.16(11)
C(18)-C(13)-C(12)	120.56(11)

C(15)-C(14)-C(13)	120.02(14)
C(15)-C(14)-H(14)	120.0
C(13)-C(14)-H(14)	120.0
C(16)-C(15)-C(14)	120.16(15)
C(16)-C(15)-H(15)	119.9
C(14)-C(15)-H(15)	119.9
C(17)-C(16)-C(15)	120.08(13)
C(17)-C(16)-H(16)	120.0
C(15)-C(16)-H(16)	120.0
C(16)-C(17)-C(18)	120.23(14)
C(16)-C(17)-H(17)	119.9
C(18)-C(17)-H(17)	119.9
C(17)-C(18)-C(13)	120.29(14)
C(17)-C(18)-H(18)	119.9
C(13)-C(18)-H(18)	119.9
C(12)-C(19)-O(26)	121.30(11)
C(12)-C(19)-C(20)	129.72(11)
O(26)-C(19)-C(20)	108.98(10)
C(21)-C(20)-C(25)	118.80(12)
C(21)-C(20)-C(19)	123.11(12)
C(25)-C(20)-C(19)	118.08(12)
C(22)-C(21)-C(20)	120.28(14)
C(22)-C(21)-H(21)	119.9
C(20)-C(21)-H(21)	119.9
C(23)-C(22)-C(21)	120.31(15)
C(23)-C(22)-H(22)	119.8
C(21)-C(22)-H(22)	119.8
C(24)-C(23)-C(22)	119.94(14)
C(24)-C(23)-H(23)	120.0
C(22)-C(23)-H(23)	120.0
C(23)-C(24)-C(25)	120.33(14)
C(23)-C(24)-H(24)	119.8
C(25)-C(24)-H(24)	119.8
C(24)-C(25)-C(20)	120.31(14)
C(24)-C(25)-H(25)	119.8
C(20)-C(25)-H(25)	119.8

C(27)-O(26)-C(19)	122.94(10)
O(28)-C(27)-O(26)	116.75(12)
O(28)-C(27)-C(1)	126.03(12)
O(26)-C(27)-C(1)	117.21(10)
C(30)-C(29)-C(34)	118.08(15)
C(30)-C(29)-C(2)	119.54(13)
C(34)-C(29)-C(2)	122.16(13)
C(29)-C(30)-C(31)	120.98(17)
C(29)-C(30)-H(30)	119.5
C(31)-C(30)-H(30)	119.5
C(32)-C(31)-C(30)	120.25(19)
C(32)-C(31)-H(31)	119.9
C(30)-C(31)-H(31)	119.9
C(33)-C(32)-C(31)	119.38(18)
C(33)-C(32)-H(32)	120.3
C(31)-C(32)-H(32)	120.3
C(32)-C(33)-C(34)	120.84(19)
C(32)-C(33)-H(33)	119.6
C(34)-C(33)-H(33)	119.6
C(33)-C(34)-C(29)	120.45(18)
C(33)-C(34)-H(34)	119.8
C(29)-C(34)-H(34)	119.8
O(36)-C(35)-O(37)	124.63(14)
O(36)-C(35)-C(2)	125.31(14)
O(37)-C(35)-C(2)	109.88(11)
C(35)-O(37)-C(38)	116.01(12)
O(37)-C(38)-H(38A)	109.5
O(37)-C(38)-H(38B)	109.5
H(38A)-C(38)-H(38B)	109.5
O(37)-C(38)-H(38C)	109.5
H(38A)-C(38)-H(38C)	109.5
H(38B)-C(38)-H(38C)	109.5

Symmetry transformations used to generate equivalent atoms:

Supplementary Table 13. Anisotropic displacement parameters ($Å^2$ x 10^3) for 3y. The anisotropic displacement factor exponent takes the form: $-2\pi^2$[$h^2a^{*2}U^{11}$ + ... + 2 h k a* b* U^{12}]

	U^{11}	U^{22}	U^{33}	U^{23}	U^{13}	U^{12}
C(1)	18(1)	10(1)	22(1)	1(1)	-2(1)	-2(1)
C(2)	22(1)	11(1)	28(1)	0(1)	-3(1)	-2(1)
C(3)	20(1)	20(1)	31(1)	2(1)	-6(1)	-7(1)
C(4)	18(1)	19(1)	22(1)	-1(1)	-3(1)	-1(1)
C(5)	18(1)	34(1)	30(1)	0(1)	-5(1)	0(1)
C(6)	25(1)	38(1)	29(1)	-2(1)	-10(1)	9(1)
C(7)	44(1)	29(1)	25(1)	3(1)	-14(1)	6(1)
C(8)	42(1)	24(1)	24(1)	7(1)	-9(1)	-3(1)
C(9)	26(1)	16(1)	22(1)	0(1)	-6(1)	-1(1)
C(10)	18(1)	13(1)	21(1)	-1(1)	-5(1)	2(1)
C(11)	16(1)	11(1)	20(1)	1(1)	-2(1)	-1(1)
C(12)	18(1)	9(1)	21(1)	0(1)	-2(1)	-1(1)
C(13)	22(1)	10(1)	19(1)	1(1)	-7(1)	-1(1)
C(14)	25(1)	17(1)	24(1)	-1(1)	-7(1)	3(1)
C(15)	42(1)	17(1)	35(1)	-4(1)	-15(1)	11(1)
C(16)	59(1)	12(1)	37(1)	2(1)	-20(1)	-4(1)
C(17)	44(1)	19(1)	29(1)	6(1)	-9(1)	-15(1)
C(18)	26(1)	18(1)	25(1)	1(1)	-5(1)	-6(1)
C(19)	20(1)	11(1)	23(1)	2(1)	-4(1)	0(1)
C(20)	22(1)	17(1)	19(1)	2(1)	-4(1)	-3(1)
C(21)	32(1)	22(1)	26(1)	-6(1)	-8(1)	2(1)
C(22)	48(1)	28(1)	32(1)	-10(1)	-11(1)	-5(1)
C(23)	39(1)	43(1)	28(1)	-6(1)	-11(1)	-14(1)
C(24)	26(1)	42(1)	28(1)	3(1)	-10(1)	-4(1)
C(25)	24(1)	24(1)	26(1)	2(1)	-6(1)	-1(1)
O(26)	26(1)	12(1)	30(1)	5(1)	-12(1)	-2(1)
C(27)	22(1)	13(1)	28(1)	3(1)	-5(1)	-2(1)
O(28)	36(1)	14(1)	48(1)	11(1)	-16(1)	-2(1)
C(29)	26(1)	17(1)	32(1)	-6(1)	-3(1)	-4(1)
C(30)	43(1)	32(1)	33(1)	-5(1)	-3(1)	-2(1)
C(31)	59(1)	56(1)	34(1)	-15(1)	5(1)	-2(1)

C(32)	53(1)	59(1)	56(1)	-29(1)	7(1)	7(1)
C(33)	57(1)	43(1)	63(1)	-16(1)	3(1)	20(1)
C(34)	45(1)	28(1)	44(1)	-4(1)	0(1)	10(1)
C(35)	23(1)	15(1)	36(1)	6(1)	-4(1)	-5(1)
O(36)	45(1)	16(1)	56(1)	7(1)	2(1)	-11(1)
O(37)	33(1)	22(1)	33(1)	8(1)	4(1)	-1(1)
C(38)	36(1)	43(1)	35(1)	18(1)	0(1)	-1(1)

Supplementary Table 14. Hydrogen coordinates (x 10^4) and isotropic displacement parameters ($Å^2$ x 10^3) for 3y.

	x	y	z	U(eq)
H(3)	11215	7570	3602	29
H(5)	13329	6822	4117	33
H(6)	13759	6219	5021	37
H(7)	11196	5649	5562	40
H(8)	7878	5472	5158	36
H(9)	6289	5757	4085	26
H(14)	8498	5316	2569	27
H(15)	8564	4312	2782	38
H(16)	5493	3845	3260	43
H(17)	2349	4377	3526	37
H(18)	2262	5379	3326	28
H(21)	4068	5159	1625	32
H(22)	1470	4645	924	43
H(23)	-1853	5082	585	44
H(24)	-2563	6039	928	39
H(25)	-27	6553	1654	30
H(30)	7302	7439	4641	43
H(31)	4970	7896	5471	60
H(32)	2820	8681	5090	67
H(33)	2942	8991	3867	65

H(34)	5240	8536	3026	47
H(38A)	10361	7662	927	57
H(38B)	11241	8188	1421	57
H(38C)	8762	8183	1131	57

4g (19.9 mg) was dissolved in CH_2Cl_2 (1.0 mL) in a 4 mL glass vial and hexane (2 mL) was slowly added to form a separate layer. The 4 mL vial was open cap. Vapor diffusion afforded crystals of the composition **4g** suitable for Xray diffraction within 2 day at room temperature. A block-like specimen of $C_{34}H_{24}O_4$, approximate dimensions 0.082 mm x 0.032 mm x 0.017 mm, was used for the X-ray crystallographic analysis. The X-ray intensity data were measured. (ellipsoid = 40%, CCDC = 1916358).

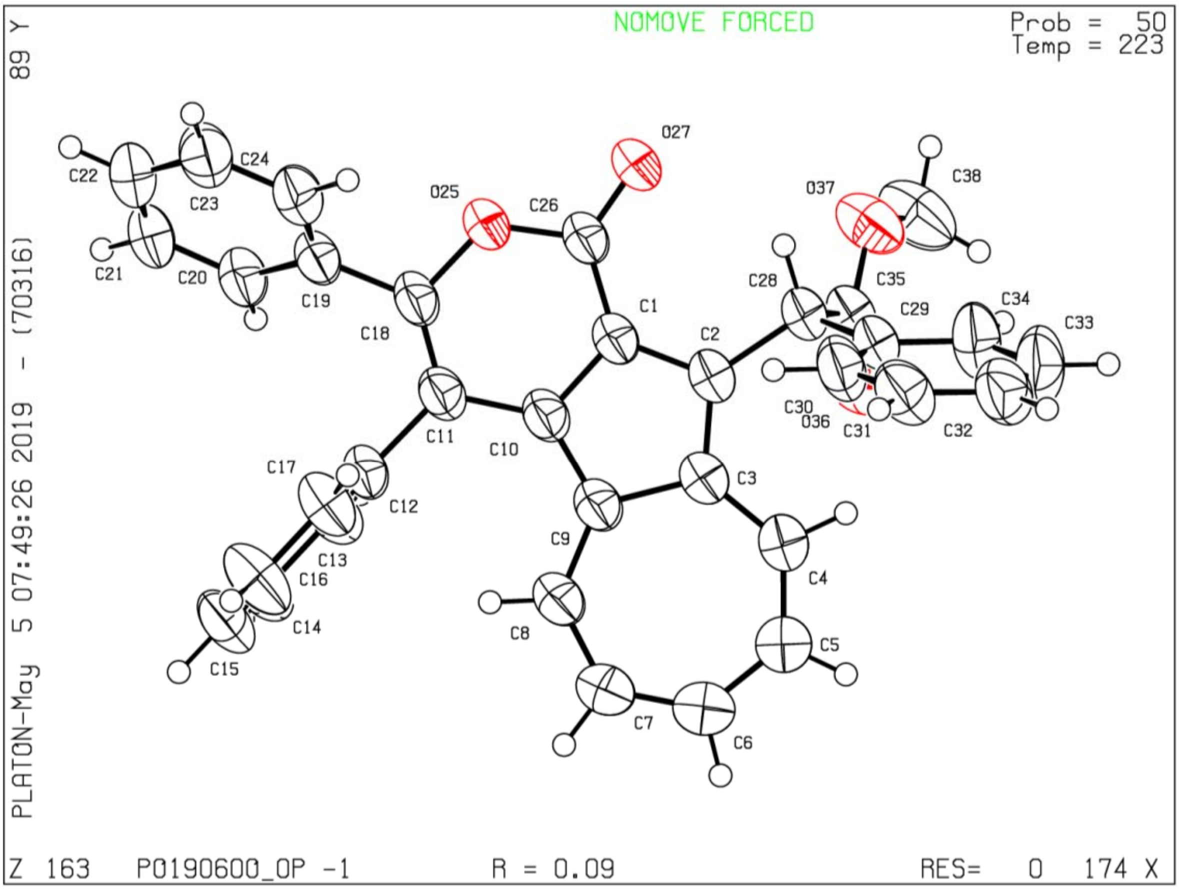

Supplementary Table 15. Crystal data and structure refinement for 4g

Empirical formula	$C_{34}H_{24}O_4$	
Formula weight	496.53	
Temperature	223(2) K	
Wavelength	0.730 Å	
Crystal system	Triclinic	
Space group	*P*-1	
Unit cell dimensions	a = 9.3460(19) Å	α = 95.94(3)°

	b = 10.173(2) Å	β = 102.85(3)°
	c = 14.036(3) Å	γ = 94.02(3)°
Volume	1288.1(5) $Å^3$	
Z	2	
Density (calculated)	1.280 Mg/m^3	
Absorption coefficient	0.087 mm^{-1}	
F(000)	520	
Crystal size	0.082 x 0.032 x 0.017 mm^3	
Theta range for data collection	1.540 to 29.999°.	
Index ranges	−12<=h<=12, −13<=k<=13, −19<=l<=19	
Reflections collected	13532	
Independent reflections	6851 [R(int) = 0.0292]	
Completeness to theta = 25.976°	99.8 %	
Absorption correction	Empirical	
Max. and min. transmission	1.000 and 0.917	
Refinement method	Full-matrix least-squares on F^2	
Data / restraints / parameters	6851 / 0 / 344	
Goodness-of-fit on F^2	1.107	
Final R indices [I>2sigma(I)]	R1 = 0.0863, wR2 = 0.2796	
R indices (all data)	R1 = 0.1267, wR2 = 0.3119	
Largest diff. peak and hole	0.337 and –0.368 e·$Å^{-3}$	

Supplementary Table 16. Atomic coordinates (x 10^4) and equivalent isotropic displacement parameters ($Å^2$ x 10^3) for 4g. U(eq) is defined as one third of the trace of the orthogonalized U^{ij} tensor.

	x	y	z	U(eq)
C(1)	6713(3)	4473(2)	3192(2)	53(1)
C(2)	6269(3)	3392(3)	2447(2)	56(1)
C(3)	6263(3)	2241(3)	2912(2)	57(1)
C(4)	5957(3)	950(3)	2427(2)	67(1)
C(5)	5918(4)	-243(3)	2816(2)	73(1)
C(6)	6147(3)	-463(3)	3801(2)	72(1)
C(7)	6558(3)	416(3)	4638(2)	68(1)

C(8)	6819(3)	1787(3)	4722(2)	61(1)
C(9)	6699(3)	2630(3)	3996(2)	54(1)
C(10)	6974(3)	4034(2)	4132(2)	53(1)
C(11)	7442(3)	4984(3)	5007(2)	54(1)
C(12)	7773(3)	4567(3)	6017(2)	56(1)
C(13)	6648(3)	4272(3)	6486(2)	66(1)
C(14)	6965(4)	3862(3)	7422(2)	75(1)
C(15)	8394(4)	3745(4)	7885(2)	80(1)
C(16)	9525(4)	4033(4)	7425(2)	89(1)
C(17)	9205(3)	4446(3)	6493(2)	70(1)
C(18)	7678(3)	6270(3)	4880(2)	55(1)
C(19)	8183(3)	7412(3)	5638(2)	57(1)
C(20)	7481(3)	7683(3)	6409(2)	63(1)
C(21)	7959(4)	8777(3)	7098(2)	74(1)
C(22)	9139(4)	9615(3)	7027(2)	79(1)
C(23)	9843(4)	9362(3)	6268(2)	77(1)
C(24)	9362(3)	8277(3)	5570(2)	67(1)
O(25)	7459(2)	6678(2)	3945(1)	59(1)
C(26)	6938(3)	5855(2)	3082(2)	54(1)
O(27)	6734(2)	6378(2)	2329(1)	66(1)
C(28)	5895(3)	3506(3)	1349(2)	60(1)
C(29)	6827(3)	2685(3)	788(2)	61(1)
C(30)	8284(4)	2611(3)	1199(2)	73(1)
C(31)	9182(4)	1903(4)	699(2)	79(1)
C(32)	8596(4)	1251(4)	-221(2)	83(1)
C(33)	7142(5)	1321(4)	-646(2)	97(1)
C(34)	6249(4)	2039(4)	-148(2)	85(1)
C(35)	4251(3)	3202(3)	926(2)	65(1)
O(36)	3514(3)	2247(3)	1042(2)	97(1)
O(37)	3684(2)	4133(2)	411(2)	83(1)
C(38)	2129(4)	3917(5)	-49(3)	101(1)

Supplementary Table 17. Bond lengths [Å] and angles [°] for 4g.

C(1)-C(2)	1.407(3)
C(1)-C(10)	1.411(3)
C(1)-C(26)	1.436(3)
C(2)-C(3)	1.398(4)
C(2)-C(28)	1.521(3)
C(3)-C(4)	1.395(4)
C(3)-C(9)	1.488(3)
C(4)-C(5)	1.383(4)
C(4)-H(4)	0.9400
C(5)-C(6)	1.396(4)
C(5)-H(5)	0.9400
C(6)-C(7)	1.364(4)
C(6)-H(6)	0.9400
C(7)-C(8)	1.388(4)
C(7)-H(7)	0.9400
C(8)-C(9)	1.389(3)
C(8)-H(8)	0.9400
C(9)-C(10)	1.419(4)
C(10)-C(11)	1.446(3)
C(11)-C(18)	1.348(4)
C(11)-C(12)	1.495(3)
C(12)-C(17)	1.376(4)
C(12)-C(13)	1.391(4)
C(13)-C(14)	1.395(4)
C(13)-H(13)	0.9400
C(14)-C(15)	1.368(4)
C(14)-H(14)	0.9400
C(15)-C(16)	1.386(5)
C(15)-H(15)	0.9400
C(16)-C(17)	1.391(4)
C(16)-H(16)	0.9400
C(17)-H(17)	0.9400
C(18)-O(25)	1.394(3)
C(18)-C(19)	1.465(3)
C(19)-C(24)	1.387(4)
C(19)-C(20)	1.400(3)

C(20)-C(21)	1.374(4)
C(20)-H(20)	0.9400
C(21)-C(22)	1.373(5)
C(21)-H(21)	0.9400
C(22)-C(23)	1.383(5)
C(22)-H(22)	0.9400
C(23)-C(24)	1.376(4)
C(23)-H(23)	0.9400
C(24)-H(24)	0.9400
O(25)-C(26)	1.370(3)
C(26)-O(27)	1.215(3)
C(28)-C(35)	1.516(4)
C(28)-C(29)	1.532(4)
C(28)-H(28)	0.9900
C(29)-C(30)	1.367(4)
C(29)-C(34)	1.380(4)
C(30)-C(31)	1.400(4)
C(30)-H(30)	0.9400
C(31)-C(32)	1.365(4)
C(31)-H(31)	0.9400
C(32)-C(33)	1.368(5)
C(32)-H(32)	0.9400
C(33)-C(34)	1.399(5)
C(33)-H(33)	0.9400
C(34)-H(34)	0.9400
C(35)-O(36)	1.198(4)
C(35)-O(37)	1.319(3)
O(37)-C(38)	1.444(4)
C(38)-H(38A)	0.9700
C(38)-H(38B)	0.9700
C(38)-H(38C)	0.9700

C(2)-C(1)-C(10)	110.7(2)
C(2)-C(1)-C(26)	128.0(2)
C(10)-C(1)-C(26)	121.2(2)
C(3)-C(2)-C(1)	107.1(2)

C(3)-C(2)-C(28)	128.1(2)
C(1)-C(2)-C(28)	124.8(2)
C(4)-C(3)-C(2)	124.9(2)
C(4)-C(3)-C(9)	126.5(2)
C(2)-C(3)-C(9)	108.5(2)
C(5)-C(4)-C(3)	129.3(3)
C(5)-C(4)-H(4)	115.3
C(3)-C(4)-H(4)	115.3
C(4)-C(5)-C(6)	128.8(3)
C(4)-C(5)-H(5)	115.6
C(6)-C(5)-H(5)	115.6
C(7)-C(6)-C(5)	130.0(3)
C(7)-C(6)-H(6)	115.0
C(5)-C(6)-H(6)	115.0
C(6)-C(7)-C(8)	128.3(3)
C(6)-C(7)-H(7)	115.8
C(8)-C(7)-H(7)	115.8
C(7)-C(8)-C(9)	130.0(3)
C(7)-C(8)-H(8)	115.0
C(9)-C(8)-H(8)	115.0
C(8)-C(9)-C(10)	127.3(2)
C(8)-C(9)-C(3)	126.9(2)
C(10)-C(9)-C(3)	105.8(2)
C(1)-C(10)-C(9)	107.8(2)
C(1)-C(10)-C(11)	120.1(2)
C(9)-C(10)-C(11)	132.1(2)
C(18)-C(11)-C(10)	117.4(2)
C(18)-C(11)-C(12)	120.3(2)
C(10)-C(11)-C(12)	122.2(2)
C(17)-C(12)-C(13)	118.8(2)
C(17)-C(12)-C(11)	120.2(2)
C(13)-C(12)-C(11)	120.9(2)
C(12)-C(13)-C(14)	120.6(3)
C(12)-C(13)-H(13)	119.7
C(14)-C(13)-H(13)	119.7
C(15)-C(14)-C(13)	119.9(3)

C(15)-C(14)-H(14)	120.0
C(13)-C(14)-H(14)	120.0
C(14)-C(15)-C(16)	120.1(3)
C(14)-C(15)-H(15)	120.0
C(16)-C(15)-H(15)	120.0
C(15)-C(16)-C(17)	119.8(3)
C(15)-C(16)-H(16)	120.1
C(17)-C(16)-H(16)	120.1
C(12)-C(17)-C(16)	120.8(3)
C(12)-C(17)-H(17)	119.6
C(16)-C(17)-H(17)	119.6
C(11)-C(18)-O(25)	121.6(2)
C(11)-C(18)-C(19)	128.1(2)
O(25)-C(18)-C(19)	110.3(2)
C(24)-C(19)-C(20)	118.9(2)
C(24)-C(19)-C(18)	119.0(2)
C(20)-C(19)-C(18)	122.0(3)
C(21)-C(20)-C(19)	120.7(3)
C(21)-C(20)-H(20)	119.6
C(19)-C(20)-H(20)	119.6
C(22)-C(21)-C(20)	119.6(3)
C(22)-C(21)-H(21)	120.2
C(20)-C(21)-H(21)	120.2
C(21)-C(22)-C(23)	120.4(3)
C(21)-C(22)-H(22)	119.8
C(23)-C(22)-H(22)	119.8
C(24)-C(23)-C(22)	120.4(3)
C(24)-C(23)-H(23)	119.8
C(22)-C(23)-H(23)	119.8
C(23)-C(24)-C(19)	119.9(3)
C(23)-C(24)-H(24)	120.0
C(19)-C(24)-H(24)	120.0
C(26)-O(25)-C(18)	124.63(19)
O(27)-C(26)-O(25)	116.5(2)
O(27)-C(26)-C(1)	128.6(2)
O(25)-C(26)-C(1)	114.93(19)

C(35)-C(28)-C(2)	110.5(2)
C(35)-C(28)-C(29)	113.2(2)
C(2)-C(28)-C(29)	112.7(2)
C(35)-C(28)-H(28)	106.7
C(2)-C(28)-H(28)	106.7
C(29)-C(28)-H(28)	106.7
C(30)-C(29)-C(34)	118.1(3)
C(30)-C(29)-C(28)	120.1(2)
C(34)-C(29)-C(28)	121.8(3)
C(29)-C(30)-C(31)	121.8(3)
C(29)-C(30)-H(30)	119.1
C(31)-C(30)-H(30)	119.1
C(32)-C(31)-C(30)	119.7(3)
C(32)-C(31)-H(31)	120.1
C(30)-C(31)-H(31)	120.1
C(31)-C(32)-C(33)	119.3(3)
C(31)-C(32)-H(32)	120.4
C(33)-C(32)-H(32)	120.4
C(32)-C(33)-C(34)	120.9(3)
C(32)-C(33)-H(33)	119.6
C(34)-C(33)-H(33)	119.6
C(29)-C(34)-C(33)	120.2(3)
C(29)-C(34)-H(34)	119.9
C(33)-C(34)-H(34)	119.9
O(36)-C(35)-O(37)	122.2(3)
O(36)-C(35)-C(28)	125.4(3)
O(37)-C(35)-C(28)	112.4(3)
C(35)-O(37)-C(38)	116.6(3)
O(37)-C(38)-H(38A)	109.5
O(37)-C(38)-H(38B)	109.5
H(38A)-C(38)-H(38B)	109.5
O(37)-C(38)-H(38C)	109.5
H(38A)-C(38)-H(38C)	109.5
H(38B)-C(38)-H(38C)	109.5

Symmetry transformations used to generate equivalent atoms:

Supplementary Table 18. Anisotropic displacement parameters ($Å^2$ x 10^3) for 4g. The anisotropic displacement factor exponent takes the form: $-2\pi^2[h^2a^{*2}U^{11} + ... + 2 h k a^* b^* U^{12}]$

	U^{11}	U^{22}	U^{33}	U^{23}	U^{13}	U^{12}
C(1)	67(1)	57(1)	36(1)	8(1)	14(1)	8(1)
C(2)	71(2)	60(1)	38(1)	6(1)	13(1)	6(1)
C(3)	70(2)	59(1)	42(1)	9(1)	13(1)	7(1)
C(4)	87(2)	63(2)	47(1)	4(1)	14(1)	4(1)
C(5)	95(2)	61(2)	61(2)	8(1)	16(1)	7(1)
C(6)	86(2)	62(2)	71(2)	18(1)	16(1)	10(1)
C(7)	86(2)	63(2)	58(2)	20(1)	17(1)	11(1)
C(8)	77(2)	65(2)	44(1)	13(1)	16(1)	11(1)
C(9)	66(1)	59(1)	40(1)	9(1)	15(1)	10(1)
C(10)	65(1)	60(1)	37(1)	8(1)	15(1)	11(1)
C(11)	64(1)	63(1)	38(1)	8(1)	15(1)	12(1)
C(12)	73(2)	61(1)	35(1)	4(1)	15(1)	12(1)
C(13)	73(2)	82(2)	49(1)	20(1)	20(1)	20(1)
C(14)	90(2)	95(2)	50(1)	22(1)	28(1)	16(2)
C(15)	98(2)	102(2)	43(1)	24(1)	14(1)	16(2)
C(16)	78(2)	136(3)	56(2)	36(2)	7(1)	16(2)
C(17)	70(2)	97(2)	46(1)	18(1)	15(1)	10(1)
C(18)	70(1)	64(2)	34(1)	6(1)	15(1)	11(1)
C(19)	72(2)	64(1)	35(1)	7(1)	11(1)	12(1)
C(20)	77(2)	71(2)	43(1)	4(1)	17(1)	9(1)
C(21)	103(2)	81(2)	39(1)	1(1)	22(1)	9(2)
C(22)	107(2)	78(2)	43(1)	-4(1)	10(1)	0(2)
C(23)	88(2)	83(2)	53(2)	3(1)	11(1)	-5(2)
C(24)	82(2)	75(2)	44(1)	5(1)	18(1)	5(1)
O(25)	84(1)	57(1)	36(1)	9(1)	15(1)	10(1)
C(26)	70(2)	59(1)	36(1)	8(1)	15(1)	10(1)
O(27)	99(1)	62(1)	38(1)	13(1)	16(1)	9(1)
C(28)	84(2)	60(1)	36(1)	6(1)	14(1)	3(1)
C(29)	85(2)	62(2)	37(1)	6(1)	15(1)	5(1)
C(30)	89(2)	89(2)	41(1)	2(1)	15(1)	10(2)

C(31)	89(2)	99(2)	57(2)	18(2)	22(1)	27(2)
C(32)	106(2)	92(2)	58(2)	8(2)	28(2)	30(2)
C(33)	120(3)	107(3)	56(2)	-20(2)	18(2)	19(2)
C(34)	93(2)	105(3)	49(2)	-13(2)	13(1)	14(2)
C(35)	84(2)	70(2)	40(1)	10(1)	13(1)	6(1)
O(36)	90(2)	89(2)	109(2)	34(2)	10(1)	0(1)
O(37)	84(1)	100(2)	67(1)	35(1)	8(1)	8(1)
C(38)	83(2)	136(3)	84(2)	47(2)	4(2)	8(2)

Supplementary Table 19. Hydrogen coordinates (x 10^4) and isotropic displacement parameters ($Å^2$ x 10^3) for 4g.

	x	y	z	U(eq)
H(4)	5746	881	1736	80
H(5)	5708	-1009	2354	87
H(6)	5993	-1356	3904	87
H(7)	6681	46	5234	82
H(8)	7125	2212	5371	73
H(13)	5665	4350	6170	79
H(14)	6198	3666	7734	90
H(15)	8608	3469	8516	96
H(16)	10506	3950	7742	107
H(17)	9975	4644	6184	84
H(20)	6673	7109	6458	76
H(21)	7482	8952	7613	89
H(22)	9470	10364	7497	94
H(23)	10656	9936	6229	92
H(24)	9831	8121	5047	80
H(28)	6142	4447	1278	72
H(30)	8694	3047	1835	88
H(31)	10185	1876	995	95
H(32)	9186	760	-558	99

H(33)	6736	882	-1282	116
H(34)	5253	2081	-451	101
H(38A)	1845	4639	-430	152
H(38B)	1570	3883	454	152
H(38C)	1929	3084	-481	152

Computational Sections

A. Mechanistic details

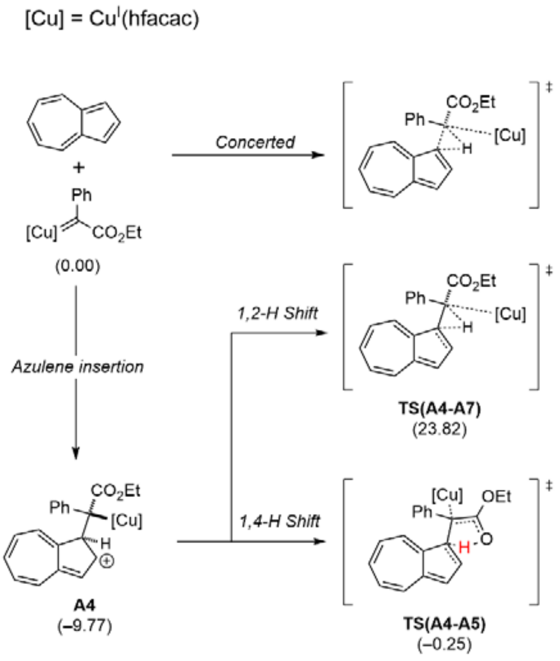

Supplementary Figure 1. Possible pathways for C–H insertion reaction. Numbers in parenthesis indicate a relative solvation phase free energy.

For the generation of the C–H insertion product, three possible pathways were considered. A C–H bond at the C3 position in azulene can be cleaved by the copper carbene intermediate in a concerted manner. We tried to obtain the concerted transition state, but it seems to be located too high in energy that we were not able to find the transition state. Alternatively, C–C coupling between azulene and copper carbene species can occur first to form alkyl copper(I) intermediate **A4** with a negligible barrier. Next, this intermediate can undergo either 1,2-H shift traversing **TS(A4-A7)** or 1,4-H shift via **TS(A4-A5)**. The former pathway directly generates the C–H insertion product and the latter one first form enol intermediate followed by tautomerization to make the product. The calculated activation barrier for the 1,4-H shift, **TS(A4-A5)** is 24.1 kcal/mol lower than the barrier for the 1,2-H shift, **TS(A4-A7)**. Based on our computed results, we suggest the reaction affording the C–H insertion product would proceed *via* the 1,4-H shift followed by tautomerization in a stepwise fashion.

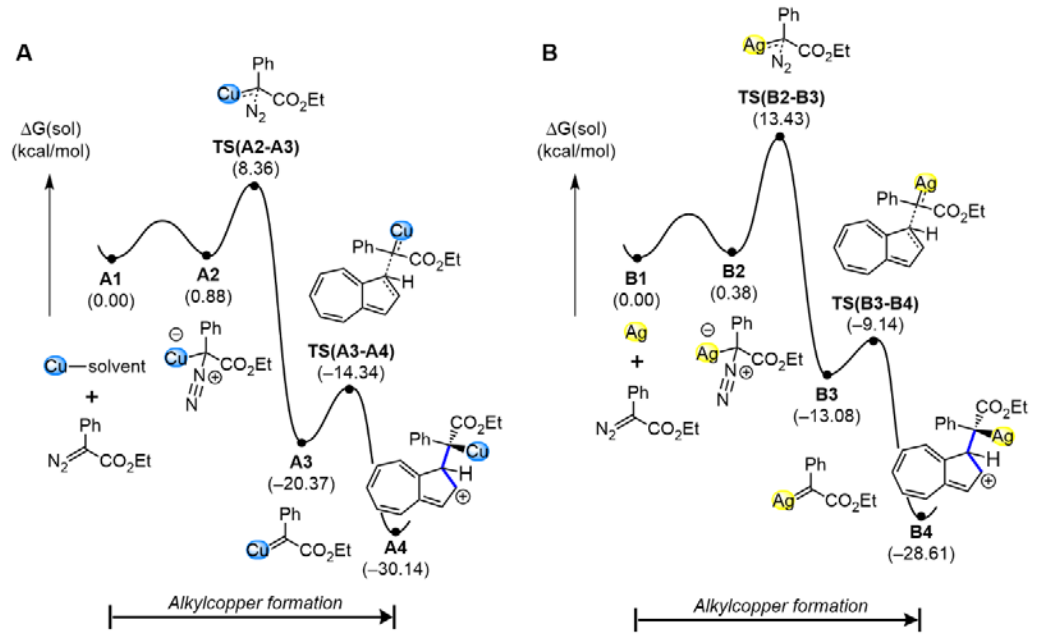

Supplementary Figure 2. Energy profiles of the formation of the metal alkyl intermediate with A, Cu^{I}(hfacac) and B, Ag^{I}OTf.

Supplementary Figure 2 shows the energy profile for the formation of the metal alkyl intermediate with Cu^{I}(hfacac) and Ag^{I}OTf. In the case of the copper(I) catalyst, one additional solvent coordinates the copper center, which is favored thermodynamically. As shown above, a dinitrogen extruding process is associated with a barrier of 8.4 kcal/mol for **TS(A2-A3)** and 13.4 kcal/mol for **TS(B2-B3)**. In both cases of copper and silver, after generation of the metal carbene intermediate **A3** and **B3**, the subsequent azulene insertion proceeds with a negligible barrier, giving the metal alkyl intermediate **A4** and **B4** which are located at –30.1 and –28.6 kcal/mol, respectively.

B. Energy components

Supplementary Table 20.

	**E(SCF)** (eV)	**[a]ZPE** (kcal/mol)	**[a]S** (cal/mol·K)	**G(solv)** (kcal/mol)
azulene	-10493.617	91.86	81.83	-3.90
DCE	-27183.706	36.64	71.86	-3.48
N_2	-2979.520	3.51	45.79	0.00
**Ethyl phenyl diazoacetate**	-17599.218	117.24	116.63	-6.17
**A1**	-58117.999	78.88	163.46	-12.52
**A2**	-48533.651	159.20	206.21	-11.42
**TS(A2-A3)**	-48533.305	157.81	203.59	-11.31
**A3**	-45554.564	153.63	189.94	-11.83
**TS(A3-A4)**	-56048.535	246.32	228.59	-15.24
**A4**	-56049.147	247.35	225.76	-18.80
**TS(A4-A8)**	-56049.292	246.94	219.81	-10.68
**A8**	-56049.767	247.85	224.85	-12.56
**TS(A8-A9)**	-56048.933	246.63	220.14	-11.90
**A9**	-56049.889	247.91	224.13	-11.07
**TS(A4-A5)**	-56048.780	244.72	225.00	-15.34
**A5**	-56049.817	247.70	225.29	-10.23
**A6**	-112100.317	496.82	381.97	-14.24
**TS(A6-A7)**	-112099.697	494.03	384.36	-15.09
**TS(A4-A7)**	-56047.802	244.01	225.11	-13.08
**A7**	-56050.471	247.99	230.32	-12.92
**B1**	-30128.880	17.56	99.33	-29.30
**B2**	-47729.387	135.72	175.49	-18.35
**TS(B2-B3)**	-47728.430	133.36	184.96	-22.18
**B3**	-44749.634	129.14	172.14	-21.46
**TS(B3-B4)**	-55243.832	222.26	206.48	-23.44
**B4**	-55244.627	223.52	201.36	-27.36
**TS(B4-B8)**	-55244.235	222.32	208.17	-20.76
**B8**	-55245.199	224.08	201.14	-18.08
**TS(B8-B9)**	-55244.298	222.66	200.27	-18.93
**B9**	-55245.120	223.90	207.95	-19.53
**TS(B4-B5)**	-55244.079	220.46	205.75	-25.93
**B5**	-55244.960	223.23	211.67	-19.65
**B6**	-110491.280	447.90	359.85	-26.84
**TS(B6-B7)**	-110490.833	446.02	347.20	-27.29
**B7**	-55245.92	223.97	205.42	-20.05

[a]ZPE and entropy are taken from optimized structures with B3LYP-D3/LACVP** level of theory.

C. XYZ coordinates

```
===============================
DCE
===============================
 C   -6.779649734   2.187753201  -0.150554001
 H   -6.309525967   2.580531836   0.751594722
 H   -6.299746513   2.617558956  -1.030397654
 C   -6.740863323   0.670018196  -0.181871623
 H   -7.220804691   0.240217492   0.697953284
 H   -7.210945129   0.277233303  -1.084040403
 Cl  -8.509690285   2.734156370  -0.148698464
 Cl  -5.010819912   0.123624213  -0.183647543

===============================
Et-ph-diazoacetate
===============================
 C    0.239486337   4.113012314   1.684016228
 C    0.122072332   2.781214952   2.120745420
 C    0.402388364   4.357161999   0.306983262
 H   -0.002943419   2.574728012   3.174613237
 H    0.496271223   5.373653412  -0.064376652
 C    0.167907551   1.736224413   1.199196577
 C    0.446376920   3.305043459  -0.602745652
 H    0.075342968   0.714883327   1.557901144
 H    0.573123455   3.520634890  -1.659875035
 C    0.329394042   1.985294461  -0.163450480
 H    0.363742918   1.164664507  -0.873751223
 C    0.191635847   5.237116814   2.642455101
 O   -0.067897774   4.118446350   4.739296913
 C    0.040366609   5.150682926   4.103608608
 O    0.034358963   6.381149292   4.671402931
 C   -0.098444700   7.861377716   6.532256126
 H   -0.207267791   7.935860634   7.618525028
 H    0.842318237   8.339095116   6.243907928
 H   -0.921107709   8.408167839   6.062579632
 C   -0.112539448   6.404833317   6.110378742
 H   -1.048198104   5.903638840   6.379054546
 H    0.707016408   5.834297180   6.559386730
 N    0.295610428   6.460599899   2.164358616
 N    0.386857957   7.505878448   1.722882390

===============================
N2
===============================
 N   -0.215999663   6.817708969   1.818311691
 N   -0.293459624   7.804716587   1.326685190

===============================
azulene
===============================
 C    4.700373173   5.496909618  -4.930182457
 C    4.700371742   6.763971329  -4.337148190
 C    4.700372696   7.092273712  -2.977604628
 C    4.700376034   4.229862213  -4.337121010
 C    4.700372696   6.247210979  -1.871740580
 C    4.700376987   3.901599407  -2.977576017
 C    4.700373173   4.746660709  -1.871744990
 H    4.700370789   5.496898174  -6.018956184
 H    4.700368404   7.606145859  -5.025147438
 H    4.700372696   8.157588005  -2.747503757
 H    4.700377464   3.387663126  -5.025089264
 H    4.700376511   2.836293697  -2.747431040
 C    4.700376987   6.647090435  -0.525150418
 H    4.700379848   7.673511505  -0.179878592
 C    4.700374126   5.496888161   0.280778170
 H    4.700372696   5.496886253   1.365219951
 C    4.700369835   4.346719742  -0.525185466
 H    4.700364113   3.320288897  -0.179956481

===============================
A1
===============================
 Cu  -2.912740469   0.013645723  -0.109367654
 O   -1.571329236  -1.476780415  -0.155006155
 O   -1.591008782   1.524490356  -0.134229213
 C   -0.337440640  -1.210598588  -0.186598271
 C   -0.353938282   1.274946094  -0.164647773
 C    0.307279319   0.036572520  -0.192539483
 H    1.386064410   0.043995492  -0.220043272
 C    0.548431098  -2.474215746  -0.221642092
 F    1.870726705  -2.201475143  -0.258398712
 F    0.318318665  -3.230853319   0.869258285
 F    0.254622906  -3.215495110  -1.307583809
 C    0.515767395   2.550163031  -0.163461491
 F    0.209184527   3.321737528  -1.223681450
 F    0.279583067   3.269061565   0.951721072
 F    1.841474175   2.296379328  -0.212214455
 C   -6.209748268   0.378436714   0.631881893
 H   -6.168712616   1.459094763   0.490927398
 H   -7.089752674   0.110164173   1.220727563
 C   -6.237316132  -0.364176035  -0.687578797
 H   -6.171725273  -1.443904281  -0.548724174
 H   -7.150724888  -0.110790424  -1.230347037
 Cl  -4.764543533  -0.075531207   1.641811609
 Cl  -4.853053093   0.111798294  -1.770366788

===============================
A2
===============================
 Cu  -2.982491255  -0.317647904  -0.239530355
 O   -1.546597481  -1.623613000   0.012549818
 O   -1.810094714   1.236688852  -0.757095635
 C   -0.335439801  -1.295619488  -0.145901442
 C   -0.557772219   1.089620590  -0.792383015
 C    0.208213821  -0.058831047  -0.523212969
 H    1.281509161   0.016051110  -0.609275997
 C    0.632366955  -2.461957693   0.140068576
 F    1.927016139  -2.144665241  -0.071016841
 F    0.511023223  -2.855565548   1.423342824
 F    0.335559249  -3.515041113  -0.644411743
 C    0.187345043   2.384373665  -1.178115129
 F   -0.246246323   2.829900503  -2.371987581
 F   -0.057588413   3.344526291  -0.264126122
 F    1.525246263   2.227415562  -1.259611607
 C   -5.465316772  -0.409414142  -1.292643309
 C   -5.265375137  -1.729879618  -1.739608645
 C   -6.039147377   0.531012058  -2.161422014
 H   -4.821529865  -2.459156513  -1.072933674
 H   -6.202785015   1.555369020  -1.837862611
 C   -5.650493145  -2.093367815  -3.028236151
 C   -6.413393974   0.156216606  -3.451066256
```

H	-5.493248463	-3.115821362	-3.359509706
H	-6.859460354	0.893517911	-4.112311363
C	-6.225110054	-1.155923843	-3.888427734
H	-6.521013260	-1.444596052	-4.892656803
C	-5.030233860	-0.043783322	0.099272691
O	-5.631327152	-2.109491587	1.161972404
C	-5.327066898	-0.942094386	1.271505237
O	-5.196579456	-0.286509812	2.439530373
C	-5.347576141	-0.147912234	4.822425365
H	-5.518776894	-0.707979858	5.746846676
H	-4.361253262	0.321017742	4.878804684
H	-6.104015350	0.639356315	4.750560760
C	-5.432991028	-1.085193396	3.634145260
H	-6.415229797	-1.559038877	3.546104670
H	-4.679594517	-1.878627777	3.669985771
N	-5.077141285	1.272707343	0.408243537
N	-5.107158184	2.375109911	0.653226674

================================

TS(A2-A3)

================================

Cu	-2.954651594	-0.483784318	-0.387578607
O	-1.326517582	-1.573303938	-0.164739221
O	-1.889137030	1.254252315	-0.540063083
C	-0.156873658	-1.100154281	-0.107274048
C	-0.633985341	1.295225859	-0.427839965
C	0.260789156	0.232966766	-0.224357158
H	1.314896107	0.451768398	-0.148713380
C	0.915073216	-2.179687500	0.145509571
F	2.172630310	-1.687706828	0.152202576
F	0.700488031	-2.768282652	1.338727951
F	0.852605879	-3.131299973	-0.802830398
C	-0.055821247	2.722272873	-0.524522066
F	-0.379249275	3.274731159	-1.709635973
F	-0.575369000	3.497733355	0.447467148
F	1.288129330	2.762845755	-0.406404793
C	-5.720379829	-0.758843720	-1.497312069
C	-6.990636826	-1.364698410	-1.473222375
C	-5.203374863	-0.307504445	-2.726011276
H	-7.392780781	-1.734678745	-0.537283838
H	-4.222411633	0.161585867	-2.745027542
C	-7.709920406	-1.529556751	-2.653359652
C	-5.933470726	-0.455848187	-3.901103497
H	-8.682318687	-2.012563467	-2.627714157
H	-5.522373676	-0.103296012	-4.842122078
C	-7.187160969	-1.072237134	-3.866204262
H	-7.754430771	-1.200531602	-4.783759117
C	-4.901936054	-0.579910815	-0.274724483
O	-5.567203045	-2.510561705	0.931577921
C	-5.261415482	-1.334645391	0.964884400
O	-5.100567341	-0.620152354	2.095163822
C	-5.051357269	-0.375130266	4.469517231
H	-5.159615517	-0.888150275	5.430278778
H	-4.050358295	0.063654080	4.422029972
H	-5.787548542	0.433211684	4.424478531
C	-5.260126114	-1.359683514	3.336094618
H	-6.258454323	-1.808814645	3.350952387
H	-4.526869297	-2.172595739	3.349581003
N	-5.199375153	1.083980083	0.177667230
N	-5.120622158	2.193072557	0.209713727

================================

A3

================================

Cu	-0.071497835	0.044500161	-0.075056992
O	1.357054591	-0.638948619	-1.273602605
O	1.278564215	0.617619872	1.287859797
C	2.592910051	-0.624414921	-1.016898274
C	2.525120497	0.452999145	1.180444360
C	3.238108158	-0.127248213	0.123133510
H	4.313088417	-0.192081660	0.188023567
C	3.456822872	-1.241440058	-2.134922266
F	4.778188705	-1.201732635	-1.860265493
F	3.116232634	-2.528918982	-2.325810671
F	3.257119179	-0.581175685	-3.290652275
C	3.317145586	0.980583251	2.393745422
F	3.079217434	2.294712543	2.564416647
F	2.932541609	0.339353383	3.512668610
F	4.650784492	0.817956448	2.263246775
C	-2.739176273	1.321842551	-0.369051278
C	-4.160144329	1.319435835	-0.304345071
C	-2.086619854	2.522586346	-0.758215725
H	-4.675933838	0.402352810	-0.041613307
H	-1.001715183	2.524524689	-0.814874709
C	-4.880802155	2.463382959	-0.608967960
C	-2.811331511	3.667762041	-1.049385905
H	-5.965322018	2.450917959	-0.563360870
H	-2.299094200	4.579850674	-1.338721037
C	-4.209205151	3.637455225	-0.975861907
H	-4.779597759	4.531984329	-1.210015059
C	-1.940353036	0.183479965	-0.039490961
O	-3.364550591	-1.690186262	-0.356500506
C	-2.639362097	-1.052045584	0.387074947
O	-2.309919834	-1.415599823	1.636070251
C	-2.330421925	-2.903802872	3.501219749
H	-2.721187592	-3.847103119	3.896024704
H	-1.238082051	-2.957724810	3.490429163
H	-2.629259109	-2.095423937	4.174689770
C	-2.869442463	-2.671474695	2.104268312
H	-3.962147474	-2.599421263	2.085933208
H	-2.575414896	-3.462887049	1.407614350

================================

TS(A3-A4)

================================

Cu	-0.107704163	-0.154054374	-0.249513179
O	1.429311752	-1.137602925	-1.133666515
O	1.222186804	0.897785962	0.897892475
C	2.649817705	-0.949688673	-0.899537325
C	2.478862047	0.793445170	0.823117077
C	3.238718033	-0.052302953	0.008374442
H	4.314917564	-0.013629428	0.077528037
C	3.582940340	-1.840599537	-1.747171640
F	4.893042564	-1.650928020	-1.472744465
F	3.306027651	-3.140706539	-1.531209826
F	3.400949478	-1.591241717	-3.057700396
C	3.224221706	1.747315526	1.775454521
F	2.856418133	3.022802830	1.535170197
F	2.907118320	1.466873646	3.056039333
F	4.567508698	1.680369020	1.662124634
C	-2.688496351	0.824305177	-1.378963232
C	-3.986043930	0.624037802	-1.905599475
C	-2.010644197	2.004031658	-1.750446796
H	-4.530476093	-0.275396615	-1.647634268

H -1.016373277 2.177115440 -1.348128557
C -4.556005955 1.543479085 -2.776334763
C -2.584627867 2.931158066 -2.618135452
H -5.547256470 1.357964158 -3.180939913
H -2.032707453 3.824572563 -2.896017075
C -3.860074759 2.703311205 -3.135008335
H -4.307921886 3.418732405 -3.819558144
C -2.035980225 -0.113400981 -0.451959580
O -2.953563452 -2.101560593 -1.391209483
C -2.581099033 -1.493057609 -0.400646418
O -2.525687218 -2.093275547 0.823685467
C -2.708053827 -3.972501040 2.272469521
H -2.935846567 -5.040789127 2.341333151
H -1.688695550 -3.811539888 2.635123491
H -3.396206856 -3.431193113 2.930100203
C -2.846198320 -3.502606153 0.836865485
H -3.861999750 -3.643089056 0.451013684
H -2.163155079 -4.027310371 0.161559790
C -6.849253654 1.480084538 0.671605468
C -6.302924156 2.752464056 0.486889273
C -4.980773926 3.169153452 0.663223326
C -6.219350338 0.292128354 1.061939478
C -3.875992775 2.412644863 1.046989679
C -4.870450974 0.060854804 1.344812274
C -3.817110300 0.970342219 1.322448969
H -7.918380260 1.400733590 0.486420453
H -7.001911163 3.525206804 0.175912097
H -4.783657074 4.223876953 0.479720563
H -6.869155407 -0.575977564 1.144154549
H -4.593696117 -0.960686445 1.591924548
C -2.555108309 2.926169157 1.235014796
H -2.283566236 3.967130899 1.112897396
C -1.708699822 1.891561508 1.558233142
H -0.640427709 1.958785057 1.727188826
C -2.431476116 0.646315038 1.518273711
H -2.097300529 -0.261127889 1.997265816

==============================

A4

==============================

Cu -0.531255543 -0.861626327 0.341582060
O -0.128762811 -2.513431072 -0.647343636
O 1.669196606 -0.597327948 0.557372570
C 0.918284595 -2.733148098 -1.316031814
C 2.409944773 -1.029990911 -0.360786140
C 2.157458782 -2.079209805 -1.259569526
H 2.927111387 -2.363825798 -1.960899591
C 0.742833316 -3.859871626 -2.354037523
F 1.875298619 -4.138134480 -3.037409782
F 0.342608750 -4.995473862 -1.751444697
F -0.200489506 -3.518751860 -3.255198956
C 3.718412638 -0.231661975 -0.530200601
F 3.421838999 1.019855976 -0.963868618
F 4.366301060 -0.105437808 0.643456936
F 4.578043938 -0.772881806 -1.417671919
C -1.185331702 1.846049428 -0.212585017
C -2.158102036 2.649321318 -0.836470306
C 0.160382941 2.086301327 -0.548773468
H -3.207520247 2.486831188 -0.612062693
H 0.938159943 1.477079153 -0.102546461
C -1.804820657 3.632534981 -1.760109067
C 0.516918182 3.075082302 -1.465677023
H -2.583014965 4.227092266 -2.232730389
H 1.566991687 3.213946581 -1.709610224
C -0.463404685 3.856648684 -2.078829765
H -0.189245090 4.619882584 -2.801938772
C -1.547305107 0.812621474 0.832809031
O -3.604393959 -0.026407417 1.787628889
C -2.907569170 0.238155067 0.803829730
O -3.335477829 -0.032231547 -0.459542394
C -4.789467812 -1.021627307 -2.052665710
H -5.710612774 -1.593344688 -2.205042124
H -4.875196934 -0.072538219 -2.590646982
H -3.954667330 -1.583716750 -2.481185198
C -4.560091019 -0.781051099 -0.572110951
H -4.457477093 -1.720068455 -0.016847525
H -5.377737999 -0.214587584 -0.111357592
C -2.917639971 5.506104946 2.823359251
C -1.545844078 5.761151791 2.964294195
C -0.481234729 4.865231037 2.971621513
C -3.562307835 4.280598640 2.655289173
C -0.519821584 3.471829891 2.803565502
C -3.009962797 2.996835947 2.550971270
C -1.668490887 2.637625694 2.586141109
H -3.563416958 6.380174637 2.858606100
H -1.277247667 6.807073593 3.096151829
H 0.507049561 5.294451237 3.122274399
H -4.647172928 4.325996399 2.588515759
H -3.702031851 2.170196772 2.408035755
C 0.645901859 2.621405363 2.821403980
H 1.653156638 2.984687805 2.985415936
C 0.281888127 1.341948152 2.554645300
H 0.954714060 0.502727270 2.426058054
C -1.183592796 1.242529869 2.307931900
H -1.685750842 0.502819598 2.942130327

==============================

TS(A4-A5)

==============================

Cu -0.128181875 -0.272916377 -0.062127866
O 0.000850542 -2.265865326 -0.274277508
O 1.761433125 0.014536960 -0.791070342
C 0.992200077 -2.853526831 -0.778041482
C 2.482759714 -0.933589160 -1.207935095
C 2.200936317 -2.305579901 -1.241405606
H 2.946906328 -2.973868132 -1.643549323
C 0.801465869 -4.381413460 -0.881487012
F 1.879343390 -5.023646832 -1.383408308
F 0.547621787 -4.903036118 0.334972620
F -0.247783899 -4.665657520 -1.677205205
C 3.849674225 -0.461056381 -1.737700105
F 3.678215981 0.435708135 -2.730564833
F 4.542682648 0.148324668 -0.753623486
F 4.618281364 -1.460704088 -2.219352961
C -2.593155146 0.906263888 -0.500061154
C -3.654729128 -0.006072505 -0.318886727
C -2.580774546 1.644091249 -1.703722596
H -3.696247339 -0.606963634 0.578927815
H -1.769097805 2.341252327 -1.890778422
C -4.651637554 -0.148491174 -1.283369422
C -3.579332829 1.496547818 -2.663410664
H -5.457118988 -0.857075453 -1.106144190
H -3.532178879 2.086660862 -3.575296164
C -4.630439758 0.600687742 -2.461119413

H	-5.411398888	0.484323680	-3.206884384
C	-1.512840390	1.150815845	0.504162908
O	-0.900896966	1.404021263	2.754253149
C	-1.564832687	0.744424522	1.893185973
O	-2.310628414	-0.296912789	2.269924402
C	-3.306740761	-1.763912201	3.855398655
H	-3.287508488	-2.126656055	4.887893200
H	-4.298768997	-1.348460913	3.652982473
H	-3.143239260	-2.612668991	3.185350895
C	-2.234455109	-0.709737062	3.656685114
H	-1.230165720	-1.102862597	3.846606493
H	-2.385432243	0.162944227	4.298949242
C	-3.490603924	6.286750317	0.801351786
C	-2.306213617	6.839850426	0.286920279
C	-1.124389768	6.200252533	-0.070044033
C	-3.777092934	4.960272789	1.112182260
C	-0.814868271	4.833379745	0.001494143
C	-2.945373058	3.835363150	1.014748931
C	-1.642850280	3.763237000	0.544149458
H	-4.296219826	6.995819569	0.979296565
H	-2.322891474	7.916612625	0.133914083
H	-0.338200063	6.837056637	-0.471813798
H	-4.778561592	4.769885063	1.491334200
H	-3.377970219	2.889730215	1.331738114
C	0.407281637	4.248517990	-0.436924368
H	1.224272132	4.800407887	-0.885276139
C	0.378758550	2.895644188	-0.180101633
H	1.158729315	2.179675102	-0.418920815
C	-0.848725080	2.531898975	0.514525831
H	-0.543411911	2.358224392	1.734983683

==============================

A5

==============================

Cu	-0.567792773	-0.647935629	0.469299585
O	-0.911370516	-2.673683643	0.460852802
O	0.870445609	-0.771198153	-0.885258496
C	-0.322699577	-3.444427490	-0.342306465
C	1.176361322	-1.843715787	-1.476170063
C	0.672613978	-3.140014887	-1.281840444
H	1.064502120	-3.939193964	-1.892490745
C	-0.824639797	-4.898492336	-0.249050856
F	-0.126901194	-5.764747143	-1.008845091
F	-0.768231928	-5.329492092	1.028332233
F	-2.117930889	-4.959715843	-0.638411224
C	2.255212784	-1.646240711	-2.561584711
F	1.816540956	-0.784750938	-3.498917580
F	3.373555660	-1.130192280	-2.017077446
F	2.596636057	-2.793695927	-3.187428713
C	-2.693056822	0.892395079	0.284543335
C	-3.761300087	0.041942313	-0.073773496
C	-2.414863110	1.985881448	-0.564504981
H	-4.005035400	-0.808854461	0.546397924
H	-1.595585823	2.652651548	-0.316140145
C	-4.506829262	0.283583850	-1.227248073
C	-3.164994955	2.220315933	-1.712366462
H	-5.324642658	-0.388507843	-1.476127863
H	-2.920945168	3.071474314	-2.343108416
C	-4.218935490	1.370036364	-2.054124355
H	-4.804903984	1.551480055	-2.950495958
C	-1.830626249	0.716794908	1.501705885
O	-1.324462891	-0.297029972	3.620951414
C	-1.994465113	-0.299042404	2.454346657
O	-2.852197886	-1.299180388	2.330017090
C	-3.437735796	-3.611158848	2.343212128
H	-3.250783205	-4.585961342	2.804346085
H	-4.495488167	-3.361738682	2.472268820
H	-3.205841780	-3.685137272	1.279526234
C	-2.549742937	-2.569839954	2.989647627
H	-1.494935989	-2.794978619	2.820360899
H	-2.737668514	-2.452594519	4.060765266
C	-3.188550472	5.509631157	3.867887259
C	-1.856564879	5.943514824	3.918421030
C	-0.706894934	5.281521797	3.485296726
C	-3.721487522	4.312936306	3.383422852
C	-0.589128196	4.020986557	2.896864891
C	-3.049956083	3.220872641	2.820545197
C	-1.687504649	3.055306435	2.598298073
H	-3.918250084	6.214262962	4.263398647
H	-1.699942112	6.930654526	4.346775532
H	0.230837062	5.820778847	3.616243839
H	-4.802610397	4.216269016	3.449859381
H	-3.677240610	2.390096903	2.502100229
C	0.607552826	3.422775507	2.467164993
H	1.590838075	3.869274378	2.540185928
C	0.304476887	2.165482283	1.923389912
H	1.017083287	1.477043390	1.478199601
C	-1.083620787	1.916882038	2.000576019
H	-0.771887720	0.506744504	3.631193161

==============================

A6

==============================

C	0.930061877	4.954134464	6.812830448
C	2.285334826	4.837170601	6.704264641
C	4.471747875	5.026992321	7.694123268
C	5.026636600	4.877112389	9.097561836
O	2.969889402	4.826082230	5.528371334
O	3.063168049	4.703619003	7.782946587
H	4.956400871	4.344809532	6.987605572
H	4.591866016	6.049453735	7.326677799
H	4.532168388	5.577543736	9.778199196
H	4.864505768	3.862032175	9.471819878
H	6.097669601	5.091310024	9.097583771
C	0.265697837	5.289855957	8.096097946
C	0.780321121	6.274551392	8.955850601
C	-0.970558584	4.704401970	8.416806221
C	0.078545116	6.668240547	10.093166351
C	-1.674316764	5.094244003	9.555922508
C	-1.151962399	6.078146458	10.397981644
H	1.733471751	6.739444256	8.727364540
H	-1.396966815	3.947418690	7.766128540
H	0.486991227	7.449404716	10.725301743
H	-2.622304916	4.614569664	9.780997276
H	-1.699721813	6.387221336	11.284328461
C	0.917021811	1.897162676	3.408402443
C	0.225539729	1.901343703	2.177932262
C	0.851299822	2.818533421	4.444171429
C	-0.738726914	2.779797077	1.700410247
C	0.063627511	3.977778912	4.544678211
C	-1.298189878	3.900119066	2.347558260
C	-0.982871413	4.426229477	3.586673498
H	0.481466591	1.084139943	1.505661130
H	1.601408362	1.065604091	3.556906700

H	1.496607184	2.628671408	5.298153877
H	-1.131748915	2.563598633	0.709968030
H	-2.087104082	4.420551300	1.805655837
C	0.071564347	4.884660244	5.627483845
H	2.443285704	5.312726498	4.872048378
C	-0.960926890	5.840810776	5.390074730
H	-1.144318581	6.682316303	6.046669006
C	-1.602573991	5.586455822	4.153592110
H	-2.210284948	6.288854599	3.593327761
H	0.743152201	7.264030933	5.020704746
C	1.324376345	9.568412781	5.628141403
C	2.115382671	8.602140427	4.992633343
C	1.738712549	11.001158714	5.817299843
C	-0.119905993	9.225769043	5.830575943
O	1.651147604	7.366260052	4.679321766
O	3.328470469	8.839245796	4.486247063
C	4.282795906	7.733358383	4.467013836
C	5.670389175	8.334112167	4.544220924
H	4.085133076	7.084955692	5.321508408
H	4.121091843	7.164015293	3.545667648
H	5.839951992	9.038134575	3.723554850
H	5.804012299	8.846008301	5.499317169
H	6.416222095	7.535200119	4.474010944
C	3.027372837	11.504858017	5.508498669
C	0.794047415	11.904562950	6.357489109
C	3.343935251	12.841823578	5.738654137
H	3.785057306	10.845867157	5.102048874
C	1.117987275	13.239799500	6.581570625
H	-0.204807892	11.555903435	6.606688976
C	2.396290541	13.720640182	6.276133060
H	4.345863342	13.195502281	5.491322517
H	0.362133473	13.905542374	6.999042034
H	2.649679184	14.761900902	6.453326702
C	-3.179245710	8.703020096	2.479439497
C	-1.820066452	9.047927856	2.408559799
C	-0.902959883	9.216746330	3.447746277
H	-1.423181772	9.187600136	1.405591011
C	-3.985172987	8.463500023	3.595194340
H	-3.679925203	8.605311394	1.517427206
C	-1.109006166	9.100863457	4.823280334
H	0.115516163	9.462953568	3.148994923
C	-3.637742519	8.526970863	4.950719357
H	-5.014914036	8.178987503	3.395528316
C	-2.400713444	8.812890053	5.520980358
H	-4.437334538	8.290154457	5.647089958
C	-2.121873379	8.801573753	6.903519154
C	-0.758725524	9.046690941	7.083908081
H	-0.243681431	9.078790665	8.037881851
H	-2.845047235	8.600173950	7.683582306
O	2.117981434	9.306037903	9.230452538
Cu	2.433398485	8.883211136	7.278814316
O	4.356099129	8.360596657	7.590302467
C	3.055588007	9.365082741	10.068099022
C	4.948659897	8.596932411	8.682038307
C	4.414672852	9.039075851	9.896528244
C	2.609472752	9.879446030	11.454023361
C	6.474217892	8.397446632	8.588950157
H	5.083581448	9.170615196	10.733485222
F	3.619698048	9.948321342	12.345765114
F	2.072418690	11.106482506	11.340969086
F	1.666961193	9.062825203	11.972232819
F	6.767625332	7.193924427	8.049250603
F	7.002653599	9.338395119	7.776186466
F	7.101524353	8.476291656	9.776552200
O	-4.876882553	5.837216854	5.544354916
Cu	-3.031688929	5.023028851	5.631014347
O	-3.628612518	3.588104010	6.916179657
C	-5.848580837	5.431150913	6.237989902
C	-4.783359051	3.551084757	7.420787811
C	-5.893685341	4.369039059	7.149775505
C	-7.123009205	6.266284466	6.009844780
C	-4.945796490	2.443324327	8.481715202
H	-6.817737579	4.177787781	7.674386501
F	-8.145994186	5.926036358	6.819683552
F	-6.854943275	7.578696251	6.214602470
F	-7.542698383	6.134870052	4.732758522
F	-4.654936790	1.241104484	7.953634262
F	-4.090886116	2.661806107	9.505152702
F	-6.191022396	2.377183914	8.997719765

================================

TS(A6-A7)

================================

C	0.905006289	5.975621700	6.190042019
C	2.297901392	5.609265327	6.087733746
C	4.420374393	4.985099316	7.036353111
C	4.874619484	4.831153393	8.471974373
O	2.988028526	5.865627289	4.999471664
O	2.949523926	5.049898624	7.062057495
H	4.691749096	4.129629612	6.411230564
H	4.792489052	5.903429985	6.586988449
H	4.562889576	5.693571091	9.067384720
H	4.467181206	3.923303366	8.925478935
H	5.967135429	4.779046059	8.493897438
C	0.301327586	5.999695778	7.568246841
C	1.019596338	6.529819012	8.652575493
C	-1.016273499	5.582325459	7.786514282
C	0.446049988	6.640935421	9.914591789
C	-1.592816472	5.681078434	9.053809166
C	-0.866359115	6.206764221	10.121800423
H	2.038424730	6.875105381	8.512668610
H	-1.616387010	5.200562954	6.970463276
H	1.024895310	7.082838058	10.720521927
H	-2.618440151	5.355900764	9.191792488
H	-1.321359873	6.293073177	11.104896545
C	0.131070107	1.575047255	5.756405354
C	-0.567300081	0.826832533	4.800543785
C	0.323233455	2.955879688	5.817434311
C	-1.274956107	1.252846360	3.672483683
C	-0.141766399	3.943526506	4.946792126
C	-1.491058469	2.557668924	3.221286535
C	-1.027469397	3.754375696	3.766573906
H	-0.563298881	-0.249248832	4.964483738
H	0.588145196	1.001631141	6.559214592
H	0.908243656	3.316057682	6.661398888
H	-1.737766743	0.467356235	3.080209732
H	-2.117605686	2.665455341	2.336958408
C	0.096326999	5.338107586	5.080576897
H	2.511054993	6.585275173	4.512582302
C	-0.610320032	5.996884346	4.059499741
H	-0.595938325	7.062215328	3.894067764
C	-1.305868387	5.057906628	3.247084379
H	-1.705456257	5.251927376	2.255239248
H	1.084968805	7.262042522	5.626849651

C	1.255907416	10.008004189	6.127422810
C	1.928887725	9.255578041	5.136713028
C	1.666018009	11.411316872	6.480265617
C	-0.118440844	9.566477776	6.497205734
O	1.542770386	8.079171181	4.704483032
O	3.019799948	9.787448883	4.550107956
C	4.038161278	8.874724388	4.077885151
C	5.305692196	9.684147835	3.883165121
H	4.198580742	8.104529381	4.836852551
H	3.700094700	8.407567024	3.145681620
H	5.131051540	10.532580376	3.215653419
H	5.666274071	10.055547714	4.845953941
H	6.086565018	9.050898552	3.449390411
C	3.012282372	11.834621429	6.547086716
C	0.676185012	12.349102974	6.822687149
C	3.342677355	13.127622604	6.942589283
H	3.805360794	11.144315720	6.283997059
C	1.010724306	13.645193100	7.214231014
H	-0.367038429	12.054177284	6.783282757
C	2.345093727	14.044605255	7.280778885
H	4.389411926	13.417979240	6.988178730
H	0.219282642	14.345394135	7.468874454
H	2.605754614	15.052570343	7.591215134
C	-3.370985746	8.305540085	3.564279795
C	-2.183222294	9.018939972	3.329421520
C	-1.200163364	9.403170586	4.243286610
H	-1.985011339	9.271577835	2.289925098
C	-3.933471203	7.877002716	4.770518303
H	-3.945743322	8.049621582	2.675519705
C	-1.175510049	9.227069855	5.627594471
H	-0.324619591	9.892478943	3.820359945
C	-3.473172665	8.115779877	6.081365108
H	-4.884870529	7.356582165	4.692347527
C	-2.297005415	8.723338127	6.485742569
H	-4.102194786	7.734817028	6.881749630
C	-1.884872198	8.888691902	7.824643612
C	-0.583907723	9.384199142	7.826939583
H	0.022777960	9.563153267	8.708100319
H	-2.458861351	8.593348503	8.692393303
O	2.756674051	9.089651108	9.558637619
Cu	2.548968315	9.099412918	7.550585747
O	4.417986870	8.240243912	7.282783985
C	3.857001305	8.903679848	10.133252144
C	5.283214092	8.308212280	8.202637672
C	5.102280617	8.561916351	9.567043304
C	3.777268887	9.071760178	11.665132523
C	6.723104000	8.080567360	7.697898865
H	5.963854790	8.520958900	10.216426849
F	4.972598076	8.945806503	12.278944016
F	3.271948099	10.272892952	11.985424995
F	2.956422567	8.125318527	12.174956322
F	6.829255581	6.875729561	7.082440853
F	7.042828083	9.022467613	6.787419796
F	7.651734352	8.112985611	8.670619011
O	-4.909343243	4.828585148	3.467905760
Cu	-3.103237867	5.263555050	4.346825123
O	-3.819537401	5.035872936	6.216057777
C	-5.947196007	4.648963928	4.156285763
C	-5.042364597	4.846155167	6.455079079
C	-6.098673344	4.646616459	5.556062698
C	-7.214920521	4.436016083	3.300951481
C	-5.364015579	4.899847507	7.961189747
H	-7.088614464	4.496756077	5.959367275
F	-8.333274841	4.252537251	4.035027981
F	-7.417805672	5.506947041	2.508503914
F	-7.064297676	3.358275175	2.507437706
F	-4.627687454	3.992665291	8.633924484
F	-5.040815353	6.121998310	8.450983047
F	-6.659228325	4.676556587	8.250222206

==============================

TS(A4-A7)

==============================

Cu	-0.012243948	-0.115077779	0.696818650
O	1.280858994	-1.414027214	-0.100083508
O	1.262284160	1.472416520	0.399560720
C	2.245549917	-1.046406269	-0.825227857
C	2.182881117	1.393440485	-0.459669799
C	2.734178066	0.249226868	-1.055905223
H	3.547459126	0.374977171	-1.754691005
C	2.933297396	-2.220350504	-1.552062750
F	3.925555468	-1.829074860	-2.379838705
F	3.459734440	-3.076602221	-0.656185627
F	2.031302929	-2.900775433	-2.290871620
C	2.688939571	2.772939920	-0.926034510
F	1.675157428	3.426340818	-1.543533444
F	3.080453396	3.522062540	0.120867953
F	3.719520569	2.706516027	-1.793977857
C	-2.457343340	0.809792519	-0.401642978
C	-3.697296858	0.481428444	-0.974805474
C	-1.681957483	1.802588224	-1.017708182
H	-4.313321590	-0.283348978	-0.511652350
H	-0.720545709	2.088830233	-0.604219675
C	-4.139228821	1.112345934	-2.135924816
C	-2.128600121	2.437037706	-2.179746866
H	-5.099722385	0.836115718	-2.563284874
H	-1.502655625	3.196664810	-2.640167952
C	-3.356141090	2.095742464	-2.746033669
H	-3.700452566	2.587513685	-3.651463985
C	-2.083839417	0.139219210	0.912519813
O	-2.391703367	-1.916807175	2.148930311
C	-2.287814379	-1.328455925	1.077925086
O	-2.265174627	-1.959251642	-0.117227279
C	-1.273982525	-3.755302668	-1.331418276
H	-1.142658234	-4.841131210	-1.388518095
H	-1.767109871	-3.417916059	-2.248776913
H	-0.289067179	-3.284209490	-1.270508766
C	-2.103808641	-3.398708105	-0.111066379
H	-1.618498206	-3.697888851	0.821676314
H	-3.101571560	-3.853401184	-0.137717798
C	-6.230047703	0.720591307	4.832189083
C	-5.685266972	-0.207974926	3.947889566
C	-4.541891575	-0.106266156	3.139792442
C	-5.785597801	2.014107943	5.141571522
C	-3.668038845	0.964323461	2.997227192
C	-4.693333626	2.700453520	4.618814468
C	-3.760227442	2.280309677	3.663796663
H	-7.126622677	0.397599638	5.357652187
H	-6.213993549	-1.155595660	3.877691984
H	-4.296966553	-0.992669582	2.569383621
H	-6.379580498	2.555946112	5.873621941
H	-4.549386978	3.715284586	4.985973835
C	-2.515130997	1.032322288	2.131078720
H	-1.423059344	0.392529339	2.141063929

C	-1.976303101	2.358983040	2.259410143
H	-1.092064977	2.689471245	1.726216674
C	-2.709371090	3.091306448	3.172152996
H	-2.511058569	4.107371330	3.488610983

==============================

A7

==============================

C	0.916678071	5.587080956	6.525023937
C	2.428629875	5.438796997	6.303825378
C	4.318012714	4.021746635	6.567211628
C	4.633057594	2.690859079	7.223210335
O	3.124552011	6.281891823	5.782789707
O	2.897709608	4.248370647	6.733489037
H	4.554010868	4.026566505	5.497910500
H	4.863758564	4.850856781	7.029229164
H	4.374557495	2.714774609	8.286120415
H	4.069838524	1.881381750	6.749072075
H	5.701128006	2.469821215	7.131385326
C	0.459534824	5.288634777	7.956583023
C	1.334124804	5.429321289	9.041870117
C	-0.872054517	4.932092190	8.203674316
C	0.891860366	5.197789669	10.344367981
C	-1.314342141	4.699186325	9.506258011
C	-0.432692111	4.826560974	10.580142975
H	2.369763613	5.712176800	8.876424789
H	-1.574866176	4.825813770	7.384403706
H	1.585765719	5.305114269	11.173925400
H	-2.348284006	4.417281628	9.671704292
H	-0.775114298	4.640073776	11.594302177
C	0.364323735	1.059362650	6.199911118
C	-0.221143365	0.258927166	5.214690208
C	0.475338519	2.451362610	6.254839420
C	-0.885221481	0.634297967	4.041284084
C	0.005974267	3.405404329	5.348790646
C	-1.163419485	1.919511318	3.575350761
C	-0.816675007	3.150854588	4.138114452
H	-0.150759369	-0.813785851	5.385238647
H	0.812824845	0.524832428	7.034057617
H	1.014911652	2.844347477	7.108671665
H	-1.248603821	-0.181542575	3.421548605
H	-1.730500340	1.981585026	2.647596836
C	0.173384443	4.813703060	5.454106331
C	-0.509124577	5.412190437	4.385411739
H	-0.530575991	6.478256226	4.190499306
C	-1.139402628	4.424307346	3.571509600
H	-1.479029417	4.572905540	2.550601482
H	0.738089383	6.649370670	6.326323986
O	-4.715995312	5.235862255	3.881453991
Cu	-2.953270912	4.595009804	4.621704578
O	-3.652789593	4.341224670	6.475652695
C	-5.737067223	5.358645916	4.608551502
C	-4.839024544	4.635723114	6.795300484
C	-5.882332325	5.096045971	5.982418537
C	-6.966752052	5.882423878	3.837541342
C	-5.107077122	4.496995926	8.306766510
H	-6.844321251	5.274819851	6.438179493
F	-8.070647240	5.996870041	4.606543064
F	-6.702771664	7.095746517	3.316877127
F	-7.261873722	5.051006794	2.819939613
F	-4.603614330	3.339544535	8.776863098
F	-4.490769863	5.503024578	8.968003273
F	-6.415515900	4.538774014	8.626729965

==============================

TS(A4-A8)

==============================

Cu	-0.128738984	0.011294529	0.422490209
O	0.833727181	-1.025703430	-0.984909117
O	1.454712033	1.235440969	0.766398013
C	2.013908863	-0.766782641	-1.346933484
C	2.536586285	1.143770456	0.123986684
C	2.883293390	0.231303707	-0.883157074
H	3.866025448	0.301397413	-1.323674917
C	2.514340639	-1.707959414	-2.460326910
F	3.784187078	-1.460560799	-2.842592001
F	2.448254108	-2.988851786	-2.048773527
F	1.726872206	-1.587718725	-3.551388502
C	3.575982571	2.205433846	0.536096692
F	3.092885494	3.439867258	0.287747771
F	3.827432156	2.120712757	1.856028080
F	4.750984669	2.085389853	-0.115692586
C	-2.746548653	0.707110703	-0.462973148
C	-3.981626272	0.410511136	-1.061677098
C	-2.063293219	1.858006597	-0.875241160
H	-4.520730495	-0.480615675	-0.753152788
H	-1.120022178	2.130254507	-0.409799784
C	-4.506682396	1.228588581	-2.058654070
C	-2.594056845	2.683885336	-1.868650556
H	-5.457058907	0.973453522	-2.519626856
H	-2.046058416	3.570113659	-2.175784588
C	-3.814923048	2.372469664	-2.464494944
H	-4.224633217	3.013200521	-3.240260839
C	-2.276453257	-0.194947138	0.643155515
O	-2.055791616	-2.483947039	1.326162934
C	-2.142076969	-1.667237639	0.420757264
O	-2.081302881	-1.972156167	-0.887404144
C	-1.287337899	-3.298102379	-2.696380854
H	-0.969414473	-4.297181129	-3.011931896
H	-2.150583982	-3.002110720	-3.300901175
H	-0.471177131	-2.595420599	-2.878291130
C	-1.641674876	-3.311935425	-1.221984506
H	-0.778459668	-3.558918476	-0.596819460
H	-2.447136402	-4.016636372	-0.985914707
C	-5.093822002	4.126017570	2.458282709
C	-3.730178833	4.593104839	2.518528461
C	-2.565887213	3.888688326	2.487729311
C	-5.552687645	2.848130941	2.339459896
C	-2.375485659	2.461070776	2.390300989
C	-4.794964790	1.629055977	2.235816717
C	-3.445064545	1.467294335	2.242200375
H	-5.848247051	4.908029556	2.509257078
H	-3.626941442	5.673415661	2.598864317
H	-1.644907355	4.466212749	2.542379141
H	-6.632701397	2.722328186	2.301906586
H	-5.385288715	0.722863555	2.114793301
C	-1.150376439	1.832366824	2.373315096
H	-0.184493750	2.319113493	2.396325350
C	-1.318599939	0.402196616	2.190567732
H	-0.678591609	-0.369448721	2.617213488
C	-2.780584097	0.131375372	2.062277794
H	-3.210193157	-0.725888014	2.575243473

==============================

A8

==============================

Cu	1.750220060	-0.475895792	3.436032295
O	2.812589645	-1.364003420	1.974039555
O	2.362833977	-1.755100012	4.845873833
C	3.534874916	-2.374680519	2.184926271
C	3.181639671	-2.686438799	4.606378078
C	3.801568747	-3.030404329	3.396803856
H	4.465181828	-3.881441355	3.384467840
C	4.121684551	-2.984282017	0.896009147
F	5.024658203	-3.959955454	1.128769398
F	3.118840456	-3.522522449	0.165561214
F	4.717320919	-2.042438745	0.145856366
C	3.438311100	-3.595424891	5.824337959
F	3.758593082	-2.865906477	6.906157970
F	2.314207792	-4.288471222	6.107239723
F	4.429402351	-4.489726543	5.621887684
C	-1.431369185	0.179797113	2.599745274
C	-0.768732488	0.576602697	1.422326565
C	-0.864615381	0.474347115	3.837871552
H	-1.225508094	0.354815692	0.463725686
H	-1.382439017	0.183643714	4.745989799
C	0.456118494	1.226117134	1.478710532
C	0.376687646	1.142418861	3.922982931
H	0.969950914	1.512253404	0.566993415
H	0.747346938	1.477306724	4.888666630
C	1.050557137	1.506779552	2.728477955
H	1.959508777	2.101118803	2.774593115
C	-2.690209866	-0.624565005	2.519001722
O	-3.467311144	-2.895224571	2.421353102
C	-2.534593344	-2.116370678	2.495563269
O	-1.244463444	-2.493477345	2.565319061
C	0.502600312	-4.113125324	2.641230583
H	0.743691623	-5.180903912	2.641349554
H	1.020724535	-3.643735409	1.801079988
H	0.877007723	-3.675981522	3.569913387
C	-0.996615171	-3.922924042	2.533923864
H	-1.537411451	-4.389510155	3.363541126
H	-1.406731009	-4.326797009	1.602241397
C	-4.031279087	4.133839130	0.003398727
C	-3.976985931	4.399497986	1.423209786
C	-3.960817575	3.530014992	2.467525721
C	-4.050201416	2.935032606	-0.641515434
C	-3.999690533	2.084044218	2.449581146
C	-4.003849506	1.609190702	-0.075788803
C	-3.953800917	1.246110082	1.232192039
H	-4.061026096	5.020841599	-0.625896573
H	-3.953048706	5.455467224	1.684617043
H	-3.927403688	3.966965437	3.464084625
H	-4.090941906	2.968132019	-1.728260636
H	-3.999866962	0.794809580	-0.798730135
C	-4.057773590	1.283387542	3.552799702
H	-4.104889870	1.640111327	4.575190544
C	-3.999766588	-0.152676150	3.187109470
H	-4.556416988	-0.912909031	3.725911856
C	-3.905474186	-0.170863032	1.678102970
H	-4.370389938	-0.949985147	1.084601641

==============================

TS(A8-A9)

==============================

Cu	-0.818836987	-0.461460918	4.708547592
O	1.076110244	-0.785550594	5.216992855
O	-1.542175889	-1.709347129	6.169789791
C	1.420318246	-1.463341355	6.225215435
C	-0.781953931	-2.260991335	7.008056164
C	0.616235256	-2.179181814	7.122863770
H	1.098067164	-2.722348213	7.921315670
C	2.949582100	-1.536006570	6.404642582
F	3.323440313	-2.172066450	7.534808636
F	3.494798660	-2.197847605	5.363362312
F	3.480996609	-0.300985426	6.433547974
C	-1.529996395	-3.187905788	7.989012241
F	-2.545189142	-2.535249472	8.578493118
F	-2.051173925	-4.235928059	7.309581757
F	-0.740230680	-3.689734459	8.960729599
C	-2.712376595	-0.139956638	2.664438248
C	-1.934388280	0.000992454	1.522233963
C	-2.427310467	0.654387534	3.804639101
H	-2.174450874	-0.597145677	0.648782551
H	-3.134630919	0.677257597	4.630054474
C	-0.860502899	0.904379785	1.486589789
C	-1.324659228	1.540895224	3.777364016
H	-0.269331783	1.001599073	0.580761075
H	-1.145049572	2.211393118	4.613691807
C	-0.543991625	1.660366178	2.606929779
H	0.292805612	2.351202488	2.590372801
C	-3.820163965	-1.172622323	2.728434086
O	-4.135418415	-3.425955296	3.534902334
C	-3.366461277	-2.527585983	3.269191742
O	-2.029695272	-2.623023748	3.372918367
C	-0.021188984	-3.884820700	3.630777836
H	0.420184314	-4.808567524	4.017724991
H	0.207331985	-3.812967777	2.563010216
H	0.445570469	-3.037329197	4.139505386
C	-1.518801093	-3.897180796	3.861094475
H	-1.780734658	-3.977860928	4.917500496
H	-2.021436691	-4.701156139	3.316422701
C	-5.971011639	1.912725210	-1.353416324
C	-5.976585865	2.643971205	-0.149825275
C	-5.813441753	2.176399469	1.145415068
C	-5.776969433	0.552222133	-1.543642759
C	-5.507419109	0.882530808	1.614143252
C	-5.420138836	-0.424357712	-0.593607903
C	-5.146940708	-0.292827338	0.760167241
H	-6.180351257	2.490537643	-2.251927614
H	-6.188860893	3.706354141	-0.246023253
H	-5.966438293	2.915059805	1.931683898
H	-5.870637417	0.193485409	-2.566105604
H	-5.268717766	-1.426624298	-0.994783938
C	-5.645563126	0.507724047	2.952173471
H	-6.223414898	1.103297830	3.652209997
C	-5.131323814	-0.756063879	3.319308996
H	-5.559975147	-1.374648213	4.102148056
C	-4.651998997	-1.397182822	1.490494370
H	-4.773557186	-2.414192915	1.134085774

==============================

A9

==============================

Cu	-1.073019505	-0.444106251	4.717714310
O	0.454423189	-1.710838795	4.980227470
O	-1.528055668	-0.349937201	6.686045170
C	0.755545676	-2.176749229	6.113114357

C -0.877569795 -1.000312924 7.548149109
C 0.204469785 -1.878261685 7.366240978
H 0.621033013 -2.363567352 8.235753059
C 1.840363622 -3.271377087 6.052340508
F 2.391704321 -3.546581984 7.251276493
F 1.288242579 -4.419995308 5.583677292
F 2.827520609 -2.927519560 5.211052418
C -1.384628177 -0.773532629 8.988631248
F -1.293212295 0.531549931 9.309058189
F -2.678838968 -1.133525014 9.086442947
F -0.693461597 -1.472399950 9.914840698
C -1.817438245 -0.118034318 2.346525192
C -0.624720573 -0.131642118 1.596401811
C -1.997109652 0.885452151 3.331003904
H -0.485367745 -0.894035816 0.835470855
H -2.970293522 1.021653533 3.794222355
C 0.369407624 0.814353347 1.822145343
C -0.959047914 1.819430113 3.574178934
H 1.280811548 0.786871672 1.232362270
H -1.102611184 2.579502106 4.335465908
C 0.215782270 1.781563997 2.823637724
H 1.005745769 2.501201630 3.014292717
C -2.942750454 -1.127947569 2.006892681
O -2.173643112 -3.195658207 0.940343857
C -2.270099163 -2.520655394 1.940735102
O -1.770700097 -2.854726553 3.143277884
C 0.377978384 -3.923628569 2.584073067
H 0.969364762 -4.826251030 2.767939091
H 0.282648414 -3.782207489 1.505338550
H 0.898918986 -3.074848413 3.031212568
C -0.990019977 -4.084067822 3.226319551
H -0.899717569 -4.262143612 4.297824860
H -1.568472147 -4.886733055 2.760068893
C -7.208999634 1.535222292 -0.727203488
C -7.503936291 1.468726277 0.597881436
C -6.819238663 0.733278155 1.627097368
C -6.125962734 0.879220366 -1.417644382
C -5.641913414 0.033894423 1.611884952
C -5.094947338 0.157457173 -0.911707938
C -4.726711750 -0.173661336 0.464004993
H -7.870754719 2.131299496 -1.351963758
H -8.381499290 2.016040564 0.935093701
H -7.324195385 0.756936491 2.591885567
H -6.135110855 0.994468451 -2.499731064
H -4.390667915 -0.230634123 -1.646071076
C -5.227458954 -0.591333270 2.863409281
H -5.946923256 -0.591182172 3.679432154
C -4.021123886 -1.144171953 3.061502695
H -3.763978004 -1.586950183 4.019744396
C -3.518429041 -0.760344565 0.662606716
H -2.878425121 -0.970694840 -0.187435642

==============================
B1
==============================

Ag 4.737516880 -1.499922633 -2.754229546
O 4.170653343 0.232074648 -4.251132965
S 5.376983166 1.057290792 -3.858992100
O 5.951434135 0.512369633 -2.568652153
O 5.258895397 2.503965616 -3.975548267
C 6.644917011 0.486603200 -5.102655411
F 6.757567883 -0.862291396 -5.009655952
F 7.836501598 1.026552320 -4.851524353
F 6.268358231 0.792645991 -6.343536854

==============================
B2
==============================

Ag 4.090383530 -0.951635838 -1.223330617
O 4.971648693 0.635915160 -2.472155809
S 6.143963337 0.126901120 -3.299804926
O 6.808120251 -1.047919631 -2.686945200
O 6.985831738 1.190082550 -3.841670036
C 5.247097969 -0.624832928 -4.748219013
F 4.419031620 -1.604744792 -4.300838470
F 6.109758377 -1.174386859 -5.608861923
F 4.507208347 0.281690449 -5.390211582
C 3.419701815 -4.123058319 -2.707068920
C 4.122920036 -3.750101089 -1.546128035
C 3.390707493 -3.167513847 -0.472002596
C 5.580836773 -3.903960228 -1.453290462
H 3.953594208 -4.552567959 -3.548769712
C 2.047183037 -3.913374901 -2.806977749
H 3.876434326 -3.011635303 0.484229356
C 2.005999088 -2.942949533 -0.602647841
H 1.533288240 -4.205456257 -3.717849731
C 1.333711982 -3.306465149 -1.769729972
H 1.463342309 -2.517189980 0.236231163
H 0.267188966 -3.130955935 -1.865101576
C 6.458992004 -3.017572165 -0.669348538
N 6.181182384 -4.696821690 -2.320297241
O 6.021644115 -2.146054029 0.076635472
O 7.745836258 -3.267741680 -0.889623284
C 8.714344025 -2.245661259 -0.499991238
C 9.762829781 -2.197101355 -1.593268633
H 10.537674904 -1.471075773 -1.327059746
H 10.236266136 -3.174040318 -1.731516600
H 9.294739723 -1.882973194 -2.528938293
H 8.188399315 -1.295796633 -0.401692927
H 9.123800278 -2.540463448 0.471502036
N 6.693082809 -5.394385338 -3.054366350

==============================
TS(B2-B3)
==============================

Ag 2.172106504 0.044137526 -0.132276982
C 0.031613793 -0.254281938 -0.180340067
C -0.572460473 0.979121268 0.407429427
C -1.091280580 2.217999458 2.367816210
C -1.465875983 1.905029893 3.802499771
O -0.797952473 1.928623080 -0.316829175
O -0.718388796 0.954993904 1.737192273
H -0.230413094 2.891228914 2.298896790
H -1.919619083 2.657886267 1.804610729
H -2.322211981 1.224077106 3.842738390
H -0.626478076 1.446284294 4.333670139
H -1.736971378 2.830788374 4.320798874
C -0.606189132 -0.886029363 -1.344914556
C 0.121437617 -1.820227385 -2.108927488
C -1.920742631 -0.559260428 -1.737153649
C -0.444073737 -2.410618782 -3.233669043
C -2.475744247 -1.134963870 -2.874880791
C -1.741931438 -2.063743830 -3.620604277
H 1.136987448 -2.071056366 -1.813497543

H	-2.484071493	0.176265642	-1.173105001
H	0.128742591	-3.125800133	-3.815956593
H	-3.480258226	-0.860963643	-3.183368444
H	-2.179891586	-2.511273861	-4.508464336
O	4.345363617	0.213719919	-0.271161050
S	4.769362450	1.110481620	0.891202331
O	6.051639557	1.781394124	0.696656227
O	3.614161730	1.895862699	1.393826723
C	5.036303997	-0.163801387	2.223992825
F	5.331192970	0.421039671	3.389039993
F	6.017890930	-1.009349942	1.898275256
F	3.896476030	-0.880959272	2.387453318
N	-0.196860075	-1.467875242	1.144801259
N	-0.057672177	-2.343490601	1.810733557

================================

B3

================================

Ag	-0.585676253	1.867535591	6.766194344
C	0.203600705	2.703396559	5.020934105
C	0.864528596	1.789272785	4.067085266
C	2.673023701	0.284661025	3.727621794
C	3.806716681	-0.276218772	4.561639786
O	0.341431051	1.493912578	3.006715775
O	2.008580685	1.294106364	4.543359280
H	1.936915755	-0.476533204	3.453753233
H	3.026074171	0.760687709	2.806881428
H	4.514200211	0.510666907	4.837533474
H	3.420860767	-0.731774211	5.477894783
H	4.342290878	-1.041310906	3.991889715
C	0.131984979	4.082282066	4.716726780
C	-0.483030915	4.972664356	5.650937080
C	0.673091471	4.623036385	3.507824659
C	-0.533867598	6.333058357	5.389258385
C	0.601383448	5.978863716	3.253956795
C	0.002627851	6.832025051	4.196496010
H	-0.894474030	4.578282356	6.577732086
H	1.112274528	3.951349258	2.777981997
H	-0.992983997	7.006171227	6.105475426
H	1.004166126	6.386630535	2.332215548
H	-0.046573393	7.898338795	3.992203712
O	-1.451243877	1.404730797	8.692293167
S	-1.819018960	2.713030338	9.390481949
O	-2.006308794	2.575291395	10.831252098
O	-1.016878963	3.850746393	8.878520966
C	-3.503673792	3.036246777	8.663085938
F	-4.014970779	4.172621250	9.146438599
F	-4.348152637	2.034328699	8.914260864
F	-3.386921167	3.167002916	7.318152905

================================

TS(B3-B4)

================================

Ag	1.847693443	1.189551473	5.649995804
C	0.354144305	2.540515900	4.985660076
C	-0.634594202	2.847848892	6.052189827
C	-2.552013159	1.927534819	7.120916367
C	-3.516556025	0.780877531	6.889516354
O	-0.576416075	3.715434074	6.907860756
O	-1.618397355	1.919104457	6.011159897
H	-1.989536524	1.816489458	8.053872108
H	-3.056797981	2.898911238	7.151291370
H	-4.051681995	0.909108043	5.943873405
H	-2.981060743	-0.172226787	6.855359554
H	-4.249662876	0.739028454	7.701207161
C	-0.043588705	2.615806341	3.605382919
C	-1.307012081	3.128185987	3.200228930
C	0.849715769	2.188042402	2.589804649
C	-1.658594131	3.181948185	1.862776995
C	0.495506763	2.255351782	1.244933248
C	-0.754973471	2.753019333	0.878575623
H	-2.009825706	3.462083101	3.954646587
H	1.819761038	1.797877550	2.880220175
H	-2.633105278	3.564512730	1.573626161
H	1.194738746	1.916605115	0.486302465
H	-1.034574032	2.800307274	-0.170344442
S	4.736902237	0.197003275	5.593353748
C	5.780623436	-0.060901679	7.111282349
O	3.377592564	-0.289314955	6.087553978
O	5.327622414	-0.637531161	4.543671131
O	4.738494873	1.667314410	5.394558907
F	7.029061794	0.366589695	6.880659580
F	5.819443226	-1.357643127	7.440700531
F	5.275087357	0.632164001	8.143487930
C	0.987035275	5.827692509	1.832901478
C	-0.200890601	6.530014038	1.625128031
C	-1.177983522	6.884433746	2.563198566
C	-1.229315996	6.620556831	3.934586763
C	-0.321255356	5.920332909	4.740190983
C	0.864603817	5.304471016	4.359169483
C	1.483462811	5.291421413	3.020101786
C	2.715529442	4.589426041	3.145433903
C	2.868436813	4.159046173	4.452428341
C	1.712986350	4.521914482	5.205049038
H	1.610113621	5.670097351	0.954394221
H	-0.387099773	6.853449345	0.603918552
H	-2.021494150	7.445775986	2.166318417
H	-2.103330135	7.009899139	4.452006340
H	-0.589042604	5.804605961	5.786592960
H	1.592796803	4.438720226	6.274735928
H	3.708457232	3.580807924	4.819190979
H	3.412526369	4.424196720	2.333626270

================================

B4

================================

Ag	-2.966469526	4.595387459	6.281423092
C	-1.402151465	5.759083748	5.228519440
C	-1.967812419	7.121794224	5.319716454
C	-3.789953947	8.530636787	4.724480629
C	-5.064783573	8.427657127	3.908006191
O	-1.528894544	8.040457726	6.013313770
O	-3.097368479	7.274688244	4.577753067
H	-3.996134281	8.704458237	5.786270618
H	-3.141784191	9.345002174	4.380398750
H	-4.837757587	8.235551834	2.855181217
H	-5.691272736	7.610076427	4.276946068
H	-5.633946419	9.360201836	3.977962494
C	-1.290184498	5.124594688	3.857159376
C	-1.234794378	5.897927761	2.683276892
C	-1.148278475	3.729990959	3.722064257
C	-1.059325933	5.306363106	1.432025433
C	-0.956436634	3.140067339	2.472845554
C	-0.910671949	3.923113346	1.317993402

H	-1.353514671	6.975146294	2.746658325
H	-1.210206866	3.093146801	4.600605011
H	-1.036947370	5.931464195	0.542692900
H	-0.861153722	2.059511662	2.406507969
H	-0.775693178	3.461568117	0.343852609
S	-3.026848078	1.733249068	7.428564548
C	-2.372920275	2.153670073	9.122091293
O	-4.002357483	2.876320124	7.204218864
O	-3.662371397	0.423149735	7.553833961
O	-1.812125564	1.843028545	6.582010269
F	-1.474964142	1.247568965	9.526194572
F	-3.350630045	2.229649782	10.026551247
F	-1.749277711	3.362215757	9.076713562
C	2.256481171	7.802682877	3.772801161
C	3.360070229	7.119576931	3.271022320
C	1.257954359	7.347709179	4.649600506
C	3.776571512	5.805166721	3.544588089
C	1.130730033	6.092891693	5.226157665
C	3.197312832	4.856236458	4.376705647
C	2.015292406	4.966435909	5.132200241
H	3.990558147	7.676497459	2.582205057
H	2.142708302	8.829804420	3.433389425
H	0.472404599	8.053689003	4.908931255
H	4.686391354	5.490830898	3.038138866
H	3.714139938	3.901816845	4.450279713
C	-0.061923616	5.684017658	6.054964066
H	-0.206894189	6.379193306	6.892485619
C	0.312578112	4.314288616	6.516393185
H	-0.316951603	3.709653378	7.158381462
C	1.490629554	3.917718887	5.970580101
H	1.969255686	2.955568314	6.107677460

==============================

TS(B4-B5)

==============================

Ag	-2.724284649	4.466219425	6.131641865
C	-1.238907814	5.745977879	5.030337811
C	-1.869705439	7.000980377	5.397515297
C	-3.724282980	8.488087654	5.428874493
C	-5.080743313	8.545730591	4.755865574
O	-1.333762407	7.713766098	6.302775383
O	-3.002837658	7.387131214	4.813415051
H	-3.804313421	8.292765617	6.502686501
H	-3.145810366	9.407302856	5.292675018
H	-4.976356506	8.717745781	3.680570126
H	-5.621930599	7.607355118	4.906464100
H	-5.673005104	9.361100197	5.181801796
C	-1.311578631	5.098033905	3.681007862
C	-2.228976488	5.464800835	2.677067995
C	-0.413866520	4.050508976	3.389644623
C	-2.239897728	4.809401512	1.445864320
C	-0.428423733	3.401718855	2.158765793
C	-1.343157172	3.776309967	1.173576593
H	-2.941570759	6.256859779	2.863545656
H	0.291426569	3.725312948	4.148757458
H	-2.962514400	5.112856865	0.692777991
H	0.273623586	2.592827320	1.974847317
H	-1.361631393	3.266047478	0.215135679
S	-3.727395296	1.887300372	7.307281971
C	-2.588508606	1.910358906	8.786392212
O	-4.221020699	3.327808380	7.304499626
O	-4.757205486	0.897776246	7.617114544
O	-2.803240776	1.629227161	6.178411484
F	-1.928015828	0.753570795	8.899735451
F	-3.257074594	2.142155170	9.918899536
F	-1.664324522	2.901265621	8.642819405
C	2.356878996	7.624493599	3.196939230
C	3.722501755	7.466535091	3.419267654
C	1.282728553	7.094151020	3.923190594
C	4.377165318	6.720587254	4.412117481
C	1.317286968	6.245295525	5.020274639
C	3.830419064	5.924470425	5.413049698
C	2.478379250	5.670367241	5.685859203
H	4.374853611	7.993254185	2.726389885
H	2.084843397	8.250377655	2.350381136
H	0.289215416	7.363589764	3.573401213
H	5.463587761	6.760871410	4.384653091
H	4.542022228	5.418869495	6.062785625
C	0.124371566	5.768424988	5.720578194
H	-0.243214682	6.820018768	6.367414951
C	0.622138739	4.812258720	6.698185444
H	-0.019877192	4.220957279	7.342424870
C	1.998888016	4.793059349	6.701210976
H	2.632621288	4.221773148	7.368117332

==============================

B5

==============================

Ag	-2.972120523	5.976061821	5.871479034
C	-0.760180533	6.750465870	5.618059158
C	-0.958450735	7.511127949	6.773611546
C	-2.346241713	9.002652168	8.017980576
C	-3.289036751	10.134169579	7.666406631
O	-0.445761979	7.161167145	7.962433338
O	-1.629701972	8.659056664	6.794904709
H	-2.889388561	8.111679077	8.356400490
H	-1.619876266	9.281851768	8.785970688
H	-2.734654188	11.002053261	7.297801495
H	-3.998847485	9.817463875	6.897072315
H	-3.851274967	10.433135986	8.556157112
C	-1.085714102	7.206991196	4.223691463
C	-1.413664222	8.531002998	3.876338959
C	-1.050686359	6.240381718	3.197241783
C	-1.715236783	8.860792160	2.554953814
C	-1.355069399	6.576789379	1.882723212
C	-1.693149567	7.890578747	1.552720785
H	-1.438096166	9.299784660	4.636810780
H	-0.797963202	5.214569092	3.444811583
H	-1.969157696	9.888574600	2.310458899
H	-1.336388350	5.806021214	1.117804527
H	-1.937847257	8.153179169	0.527811706
S	-5.168057442	4.063987732	5.540647507
C	-4.575298309	2.875337362	6.851537228
O	-5.098341465	5.383702755	6.294542313
O	-6.512194633	3.627335072	5.174752235
O	-4.089773178	3.995377064	4.516482353
F	-4.462987900	1.638791084	6.360855103
F	-5.402013779	2.858059883	7.899455547
F	-3.348013878	3.263167143	7.294744968
C	3.515637398	7.012878418	4.360477448
C	4.572217464	6.102493763	4.422843933
C	2.193512440	6.848318100	4.787652969
C	4.598708630	4.792468071	4.918869019
C	1.597628713	5.736297607	5.372582912

C	3.558533430	4.050886154	5.479425430
C	2.229681253	4.423635483	5.690095425
H	5.518359661	6.464341640	4.023828506
H	3.749497652	7.978769302	3.919077873
H	1.537539601	7.701961040	4.629367828
H	5.558403969	4.285338879	4.853538513
H	3.811636448	3.039153099	5.794186592
C	0.236299828	5.642968655	5.767703533
H	0.070850350	6.343842506	7.825623512
C	0.032534495	4.344730377	6.291059494
H	-0.921575606	3.963503838	6.643536568
C	1.229246259	3.613708496	6.253240585
H	1.365822673	2.593374968	6.587913990

==============================

B6

==============================

C	1.417821527	4.891060829	6.398332119
C	2.721633196	4.784565926	5.980540752
C	5.082572460	5.204370499	6.403369427
C	6.047648430	4.574413776	7.389043808
O	3.064003944	4.583425045	4.690056801
O	3.746256351	4.804071903	6.824098587
H	5.263099194	4.869240761	5.380080700
H	5.120546818	6.295563221	6.458764553
H	5.808338642	4.885789871	8.407428741
H	6.005270481	3.482867002	7.331060410
H	7.068058491	4.900132656	7.164288998
C	1.030659795	5.215067863	7.795774937
C	1.874902010	5.942402363	8.677906990
C	-0.237362206	4.845609188	8.297061920
C	1.430209517	6.329717159	9.955886841
C	-0.683304846	5.247265816	9.564126015
C	0.133451179	6.011765957	10.394166946
H	2.896516085	6.154150486	8.395331383
H	-0.877738833	4.182659149	7.725746155
H	2.132888556	6.834823132	10.614126205
H	-1.671947718	4.934791088	9.888600349
H	-0.204131991	6.316658020	11.379788399
C	-0.702516258	0.964167953	5.868573666
C	-1.782168150	0.584513962	5.062868595
C	-0.058549885	2.200056076	5.934029102
C	-2.495598555	1.319293380	4.109176159
C	-0.324859828	3.362002850	5.209562302
C	-2.316824436	2.647587776	3.722192287
C	-1.379194617	3.571894646	4.181377411
H	-2.123418808	-0.439574361	5.203410625
H	-0.327164978	0.199448600	6.543975353
H	0.754335642	2.285470486	6.654205322
H	-3.308164835	0.789317548	3.618513107
H	-3.011564016	3.024381638	2.972521305
C	0.359256417	4.594981194	5.383611679
H	2.239078283	4.557964802	4.176371098
C	-0.252883077	5.533823013	4.494573593
H	0.092673130	6.546849251	4.318736553
C	-1.289701700	4.914915085	3.764815331
H	-1.909569740	5.390179634	3.015102386
Ag	-1.877053022	6.189525127	6.380585194
C	-5.641103268	4.339151859	7.201257229
F	-5.900264740	3.038623333	7.017960072
O	-3.065948725	4.025276661	6.776470184
O	-3.876373768	3.849035740	9.157011032
S	-3.925651550	4.573509216	7.880292892
O	-3.799222946	6.065988541	7.965904713
F	-5.739469051	4.964978695	6.011848450
F	-6.558103561	4.853341579	8.028863907
H	1.502550125	7.218043327	5.784861088
Ag	1.466039300	8.518601418	8.393020630
C	1.006514907	9.714123726	6.290831566
O	4.151900291	8.088915825	7.597396374
C	2.058203697	9.069751740	5.624827862
C	1.164238930	11.125349998	6.783135414
C	-0.374849349	9.238799095	5.984479904
O	2.020014286	7.792292595	5.194618225
O	3.145913839	9.718102455	5.270426273
C	4.318167686	9.006318092	4.762762547
C	5.517251968	9.885717392	5.044595718
H	4.391176701	8.047468185	5.269705296
H	4.154196739	8.842146873	3.691966772
H	5.402778625	10.867952347	4.576785088
H	5.645731449	10.014104843	6.122047901
H	6.415793896	9.410799980	4.636253834
C	2.379902124	11.604565620	7.312315464
C	0.062731735	11.998507500	6.774780750
C	2.479901552	12.895651817	7.817936897
H	3.254766941	10.969890594	7.340196133
C	0.168151096	13.294317245	7.281104565
H	-0.886670530	11.662727356	6.370469093
C	1.375283003	13.751036644	7.808236599
H	3.428937197	13.225033760	8.231474876
H	-0.701243997	13.946385384	7.261608601
H	1.455138803	14.757327080	8.209650040
S	4.557460785	8.871105194	8.802339554
C	5.559899330	7.629096508	9.755972862
O	5.448379040	10.011271477	8.551149368
O	3.396846771	9.133351326	9.716073036
F	5.983448505	8.128799438	10.916935921
F	6.622415066	7.240023136	9.033428192
F	4.804576874	6.536540508	10.007088661
C	-1.962519407	8.633640289	1.735439301
C	-0.718897820	9.109745026	2.171884060
C	-0.262164503	9.303239822	3.477551222
H	-0.009741001	9.359064102	1.386523962
C	-3.092191458	8.269761086	2.474866152
H	-2.070118189	8.548761368	0.655551910
C	-0.925652385	9.075257301	4.683546543
H	0.751842141	9.686865807	3.568470240
C	-3.263211966	8.286278725	3.863248348
H	-3.954601765	7.946519375	1.896909237
C	-2.339129925	8.625248909	4.849528313
H	-4.239729881	7.975467205	4.232960701
C	-2.587575674	8.573811531	6.247440338
C	-1.404173613	8.959121704	6.913621426
H	-1.308134913	9.040788651	7.992005348
H	-3.544068336	8.366209984	6.713070869

==============================

TS(B6-B7)

==============================

C	1.431890726	5.274236202	6.222854137
C	2.767606020	4.900609970	5.792321205
C	4.972700119	4.035779476	6.170363903
C	5.627894878	3.308850288	7.323122025
O	3.231134415	5.280635357	4.629845142

O	3.541027546	4.162319660	6.516268730
H	5.032701015	3.486539125	5.227244854
H	5.354216576	5.048809528	6.039685726
H	5.525352955	3.885318041	8.244043350
H	5.191112041	2.316476107	7.466872692
H	6.694959164	3.192842960	7.109566689
C	1.143847585	5.263185024	7.704390526
C	2.113149166	5.752316952	8.615863800
C	-0.105726130	4.897448063	8.213476181
C	1.800276637	5.929220200	9.969500542
C	-0.412378579	5.060857296	9.569878578
C	0.524887919	5.597629547	10.447875977
H	3.123153210	5.941454411	8.278808594
H	-0.860786915	4.482521534	7.559045315
H	2.571346283	6.300460339	10.639569283
H	-1.397738695	4.771890640	9.921381950
H	0.279952794	5.736537457	11.496703148
C	-0.081848212	1.044191718	6.092269897
C	-1.076323986	0.422995865	5.324477196
C	0.353912354	2.366559982	6.049040794
C	-1.884318948	0.944274783	4.311645508
C	-0.084851824	3.406224012	5.223947048
C	-1.910895705	2.246677399	3.804043531
C	-1.138743520	3.342540503	4.176757336
H	-1.247256756	-0.626779497	5.556590557
H	0.405428648	0.411403269	6.829975605
H	1.134548903	2.632002592	6.758896828
H	-2.592725754	0.249506250	3.867499590
H	-2.638318539	2.436796665	3.015782118
C	0.391182154	4.738863945	5.263614178
H	2.741071939	6.094831944	4.359233856
C	-0.309010208	5.476346970	4.269984722
H	-0.096216835	6.506400108	4.007496357
C	-1.235547185	4.637013435	3.613687754
H	-1.863929987	4.910344601	2.774651289
Ag	-2.331908226	6.084547043	5.686390877
C	-5.469947815	4.865394115	7.513139248
F	-5.973995209	3.674889565	7.170274258
O	-3.053173065	4.157730103	6.832596779
O	-3.695875406	3.807015657	9.232409477
S	-3.704136848	4.715736389	8.080548286
O	-3.310181141	6.126078606	8.331261635
F	-5.512139797	5.672988415	6.423702240
F	-6.238940239	5.405918598	8.463291168
H	1.478767395	6.575604916	5.761855125
Ag	1.715884089	8.240892410	8.168313980
C	1.017731428	9.395699501	6.236765862
O	4.240680695	7.078756809	6.669538975
C	1.927375793	8.728862762	5.351043224
C	1.285818100	10.821935654	6.648599625
C	-0.418144733	9.042325974	5.975274563
O	1.716620684	7.541030884	4.877550125
O	3.002058268	9.389106750	4.931348324
C	3.930149078	8.737409592	4.022115707
C	4.990643501	9.764885902	3.684292793
H	4.356394291	7.871156216	4.530498028
H	3.370951653	8.409152031	3.138275146
H	4.543068886	10.653918266	3.229711294
H	5.525937080	10.050575256	4.591855049
H	5.704632282	9.332618713	2.975230694
C	2.574973106	11.270888329	7.007364273
C	0.232106641	11.749937057	6.721787453
C	2.792033672	12.587127686	7.400928497
H	3.413433075	10.586808205	6.993969917
C	0.453823924	13.068652153	7.122137547
H	-0.775077641	11.440140724	6.465079308
C	1.734990001	13.499409676	7.460628510
H	3.798299789	12.896826744	7.671005726
H	-0.384477854	13.759583473	7.166113853
H	1.908713937	14.525568962	7.773028374
S	4.955158710	8.063964844	7.535117626
C	6.123617172	6.968621731	8.482905388
O	5.802907944	9.029635429	6.826914310
O	4.090227604	8.616163254	8.620460510
F	6.917904854	7.672257900	9.287965775
F	6.888402462	6.255738735	7.632782936
F	5.417670250	6.092589855	9.233899117
C	-2.332048893	9.883260727	1.902981639
C	-1.018756151	10.081123352	2.338572502
C	-0.467034966	9.825104713	3.598514795
H	-0.336882830	10.506191254	1.605632663
C	-3.442506075	9.396927834	2.604786396
H	-2.525920630	10.165720940	0.869438052
C	-1.070737123	9.280870438	4.729673862
H	0.578589797	10.102615356	3.718049288
C	-3.513423204	8.952042580	3.926245928
H	-4.375779152	9.365094185	2.047204256
C	-2.493607283	8.866039276	4.875868797
H	-4.492856503	8.618815422	4.269412994
C	-2.638797522	8.393654823	6.209972858
C	-1.378064632	8.520736694	6.856716633
H	-1.201549172	8.234041214	7.888167858
H	-3.570958138	8.164851189	6.715213776

==============================

B7

==============================

C	1.484213114	8.286153793	4.990913868
C	0.113746554	8.966627121	5.149170876
C	-2.095715046	9.031208992	4.199886322
C	-1.987556815	9.770686150	2.878119469
O	-0.046773266	10.002555847	5.753377438
O	-0.845018983	8.321069717	4.453529835
H	-2.849947214	8.246560097	4.151756763
H	-2.288091898	9.703638077	5.038491249
H	-1.169097662	10.496527672	2.905926943
H	-1.818454266	9.064759254	2.062393188
H	-2.920473099	10.308412552	2.678787470
C	2.018444061	8.797254562	3.651096821
C	3.035166502	9.756139755	3.609719753
C	1.463771224	8.337742805	2.450348139
C	3.508541822	10.229665756	2.383300304
C	1.931513906	8.811601639	1.227461576
C	2.960307121	9.756838799	1.190184236
H	3.459794998	10.131031036	4.537506104
H	0.657813728	7.616029263	2.474165916
H	4.303635597	10.969848633	2.362975597
H	1.485066652	8.438832283	0.310369611
H	3.328948498	10.126382828	0.237400711
C	4.440719604	5.166776180	3.371825457
C	4.423061371	3.761650562	3.386529446
C	3.521312237	6.066934586	3.898603439
C	3.484660864	2.894049644	3.937395334
C	2.325809956	5.814496040	4.592639446

C	2.302165031	3.217440605	4.618221760
C	1.778038979	4.467693329	4.915143490
H	5.268564224	3.283993244	2.895200253
H	5.296607018	5.616141319	2.874316692
H	3.767174006	7.113607883	3.756117821
H	3.688605070	1.832669139	3.822211504
H	1.704901576	2.372894287	4.959001541
C	1.429917097	6.776329994	5.114532948
C	0.396967441	6.076309681	5.791071892
H	-0.382886082	6.548836708	6.377464294
C	0.566460788	4.676806927	5.639956474
H	0.022550510	3.903194189	6.169116497
Ag	-1.019861221	5.309754848	3.890310764
C	-1.656691670	6.367352486	0.222866416
F	-1.584848285	5.704183102	-0.931633055
O	-1.813158512	4.270782948	1.823886871
O	-3.959308386	5.158020496	0.811345220
S	-2.687839508	5.439678192	1.470506787
O	-2.720695496	6.408612251	2.626456499
F	-0.399171114	6.524272919	0.699688435
F	-2.166934967	7.585150719	-0.003838168
H	2.101253271	8.708088875	5.792044640

================================

TS(B4-B8)

================================

Ag	-2.040835381	4.719064236	6.498893738
C	-1.199069023	6.235005379	4.789865017
C	-2.583719969	6.782707691	4.717831612
C	-4.844698429	6.238265514	4.151582241
C	-5.586309910	4.929444790	3.943842173
O	-2.915193319	7.856398106	5.196777344
O	-3.426979542	5.927079678	4.101077557
H	-5.070149899	6.691151619	5.123053074
H	-5.060605526	6.978668690	3.370651007
H	-5.316670895	4.476613998	2.982848167
H	-5.345667362	4.229689121	4.750377655
H	-6.665669441	5.112716675	3.949049711
C	-0.719417989	5.225030899	3.790137291
C	-0.811897099	5.573705196	2.432405472
C	-0.131041586	3.999182224	4.125591278
C	-0.358454674	4.708178997	1.440417528
C	0.333016872	3.134206295	3.132250309
C	0.218952894	3.484104395	1.787457705
H	-1.263749599	6.523100376	2.157165766
H	-0.016302384	3.718614101	5.169866085
H	-0.458346456	4.988527775	0.395191699
H	0.776470840	2.183452129	3.414386272
H	0.572191596	2.807717562	1.014365077
S	-3.697399855	2.976213217	8.160349846
C	-3.455152512	1.219587326	7.590650558
O	-2.278364182	3.408974648	8.446884155
O	-4.615668774	2.935932636	9.295694351
O	-4.179138660	3.656977892	6.906650066
F	-4.615423203	0.685065389	7.194523335
F	-2.941663027	0.468341172	8.571340561
F	-2.599492311	1.209023237	6.549122810
C	3.136432648	7.346607685	3.191526413
C	4.133190632	6.567282200	3.707123041
C	1.814072490	7.566054821	3.703966141
C	4.121405602	5.763701916	4.899965763
C	1.244039893	7.047112465	4.826498508
C	3.128744602	5.584501266	5.818678856
C	1.812278032	6.163944721	5.844460487
H	5.063724995	6.539597034	3.144262075
H	3.361673594	7.867834091	2.263327122
H	1.182150006	8.218319893	3.104245663
H	5.044685841	5.219912529	5.090115070
H	3.349847317	4.917039871	6.650089264
C	-0.184939295	7.309194088	5.219437599
H	-0.563733757	8.324159622	5.107390881
C	-0.345477760	6.622319698	6.525178909
H	-1.097431779	6.955455303	7.232420444
C	0.869025767	5.926458836	6.831428528
H	1.010993361	5.284759998	7.692838669

================================

B8

================================

C	1.752487183	7.590105057	4.834985256
C	2.623753309	6.434519291	4.496223450
C	3.583359480	4.411929131	5.272109509
C	3.671356440	3.682119608	6.596818447
O	2.994049549	6.147254944	3.348483801
O	2.999868393	5.716310978	5.550473213
H	2.923190832	3.902304411	4.565994263
H	4.561817646	4.558666229	4.804686069
H	4.297063828	4.228368282	7.309048653
H	2.673853159	3.558714867	7.027432919
H	4.107232571	2.689980030	6.442632198
C	1.299036384	8.371576309	3.639023304
C	0.071474396	8.057950020	3.023402929
C	2.128227949	9.330307961	3.048018456
C	-0.292975396	8.674525261	1.816140532
C	1.758088470	9.951135635	1.853965998
C	0.554531515	9.616857529	1.227029920
H	-0.615349293	7.368139744	3.505493402
H	3.073420525	9.575581551	3.523426294
H	-1.244913220	8.422608376	1.357684493
H	2.415390491	10.690649033	1.405550122
H	0.272366375	10.096179008	0.294321418
C	-2.899961472	8.435867310	6.183546066
C	-3.102192879	9.779116631	6.289746284
C	-1.655426145	7.714584351	6.108710289
C	-2.125810623	10.840336800	6.364462376
C	-0.392039537	8.214043617	6.146038055
C	-0.767998934	10.771336555	6.371286392
C	0.080745883	9.602639198	6.318516731
H	-4.138095379	10.110978127	6.315505505
H	-3.789409876	7.812746048	6.124487877
H	-1.745534182	6.642235279	5.954527855
H	-2.549557686	11.841019630	6.423372746
H	-0.232033804	11.717331886	6.433970451
C	0.814117789	7.349511623	6.043153763
H	0.775554299	6.324122906	6.386769295
C	2.003990173	8.256886482	6.221460342
H	2.901596308	7.937530994	6.740640163
C	1.444921017	9.617635727	6.385484219
H	2.053021669	10.506932259	6.501905918
O	0.428158134	4.551462173	4.445916176
Ag	1.224894404	5.945337296	1.839021564
C	-1.292048335	2.632536173	3.821162701
S	-0.784701705	4.407428741	3.603793859
O	-1.948882937	5.215877056	4.004871368

O	-0.475329995	4.460372448	2.121219635
F	-2.365994215	2.356777191	3.075505733
F	-1.584002972	2.403863430	5.108314991
F	-0.288627267	1.822659850	3.457726955

==============================

TS(B8-B9)

==============================

C	1.704613805	7.655993938	4.818887711
C	2.613268137	6.481903076	4.484773159
C	3.646081924	4.523887157	5.342413425
C	3.682313919	3.829350710	6.688529968
O	2.968006611	6.173512936	3.345916748
O	2.996370792	5.810982704	5.558920383
H	3.050822496	3.974792004	4.610068798
H	4.644476414	4.706125736	4.932504654
H	4.233864307	4.421504021	7.425000668
H	2.665141821	3.670476913	7.056588173
H	4.171984673	2.855654716	6.589185238
C	1.342635155	8.419528008	3.554555178
C	0.129352391	8.154539108	2.892443657
C	2.244136095	9.324500084	2.988226652
C	-0.145467475	8.763273239	1.654198527
C	1.963417530	9.937151909	1.766649365
C	0.775654376	9.649416924	1.087734818
H	-0.614231229	7.515368462	3.364518881
H	3.172021151	9.541050911	3.509973526
H	-1.089685321	8.556521416	1.158480167
H	2.676615000	10.635673523	1.338148355
H	0.561450779	10.123490334	0.134574413
C	-2.910981655	8.417537689	6.508304119
C	-3.097343683	9.790125847	6.546022415
C	-1.715877891	7.698924541	6.302110195
C	-2.135757685	10.819128036	6.479282856
C	-0.431603909	8.164821625	6.062017441
C	-0.759795547	10.709370613	6.368732452
C	0.054258190	9.565787315	6.230182171
H	-4.127124786	10.123737335	6.663502216
H	-3.806564093	7.808094501	6.601184368
H	-1.828683376	6.619129658	6.227861404
H	-2.524968863	11.831758499	6.557409286
H	-0.210555688	11.649662018	6.409298420
C	0.584496379	7.235013962	5.732329845
H	0.492479235	6.181842804	5.962470531
C	2.152782440	8.431979179	6.026525497
H	3.086200953	8.153617859	6.505698204
C	1.441820979	9.605870247	6.362605572
H	1.946944952	10.458560944	6.806004524
O	0.598387003	4.350761414	4.381458759
Ag	1.156683326	6.046311855	1.776216865
C	-1.360412240	2.664831161	3.798929214
S	-0.660251260	4.374874592	3.598413467
O	-1.702752352	5.301350594	4.074577808
O	-0.417612463	4.438596725	2.104110718
F	-2.491151571	2.535044193	3.098618746
F	-1.622389674	2.441739559	5.093919277
F	-0.476214975	1.753969073	3.374993801

==============================

B9

==============================

C	1.643830180	8.080124855	4.547975540
C	2.554370642	6.824453354	4.557686806
C	3.372204065	5.068241596	5.917736053
C	3.399082184	4.758479595	7.400167942
O	2.946252108	6.225803852	3.555906534
O	2.816612005	6.412825584	5.783773899
H	2.721298456	4.391037464	5.357784271
H	4.368763447	5.057723522	5.465709686
H	4.027337074	5.472874641	7.940457821
H	2.388621092	4.798357487	7.815817833
H	3.800724983	3.752936363	7.558955193
C	1.542228580	8.574028969	3.086115360
C	0.339451015	8.465215683	2.365650177
C	2.669219017	9.097480774	2.432494402
C	0.276440859	8.859172821	1.018989801
C	2.600726843	9.500837326	1.100688696
C	1.405589342	9.377057076	0.385113358
H	-0.554254651	8.101773262	2.863935947
H	3.613362074	9.171216965	2.963410616
H	-0.662705123	8.764034271	0.481918961
H	3.485072851	9.903682709	0.615055859
H	1.355691791	9.685781479	-0.654834092
C	-2.768141270	8.203162193	7.081380367
C	-2.881511450	9.454858780	7.786314487
C	-1.740433574	7.722218513	6.336678982
C	-1.931152344	10.416790009	7.934601307
C	-0.445171982	8.309935570	5.997183800
C	-0.583223283	10.418924332	7.436635017
C	0.079485565	9.548284531	6.612781048
H	-3.842806101	9.637166023	8.262788773
H	-3.643379688	7.559875488	7.143671513
H	-1.904077530	6.752137184	5.869937897
H	-2.203665018	11.292671204	8.519403458
H	0.006660273	11.272222519	7.769287109
C	0.308762938	7.627747536	5.097095966
H	-0.068863437	6.687095165	4.719583511
C	2.217467070	9.175674438	5.421839714
H	3.257559061	9.449162483	5.268586159
C	1.474161148	9.849986076	6.311522007
H	1.929562688	10.674749374	6.855411530
O	0.402360290	4.373991489	4.189402580
Ag	1.291907310	5.943752289	1.868790507
C	-1.455181479	2.646092892	3.401024103
S	-0.842888892	4.401113033	3.381661654
O	-1.943302274	5.222034931	3.912486553
O	-0.559242427	4.624804974	1.907023787
F	-2.573496819	2.534607172	2.677901745
F	-1.712051034	2.277476072	4.662141800
F	-0.523610294	1.828945637	2.893802166

D. Vibrational frequencies

```
==============================
DCE
==============================
   122.85  219.57  299.68  707.47  751.37  791.04
   1014.64  1064.02  1149.89  1269.19  1303.19  1351.98
   1503.10  1506.49  3107.31  3114.85  3167.29  3189.10
==============================
Et-ph-diazoacetate
==============================
   23.98   35.61   77.22   98.19  101.64  148.27
   149.50  222.49  265.68  301.44  309.09  338.05
   400.70  412.89  495.82  503.83  552.16  557.22
   634.58  680.15  706.99  730.49  774.77  823.22
   823.75  856.87  886.21  925.53  978.79 1005.81
   1009.95  1013.03  1060.57  1084.41  1123.43  1140.99
   1185.15  1199.90  1203.83  1235.33  1296.52  1301.19
   1348.08  1363.36  1380.43  1409.29  1443.49  1494.86
   1498.77  1512.85  1532.53  1543.67  1631.25  1660.96
   1786.75  2214.02  3055.83  3062.76  3104.03  3133.33
   3139.29  3176.64  3184.47  3194.52  3207.13  3255.87
==============================
N2
==============================
   2456.83
==============================
azulene
==============================
   166.72  172.45  321.59  342.37  414.68  430.91
   500.81  574.63  610.76  685.76  729.18  745.94
   749.80  786.84  800.20  832.26  880.77  913.69
   941.49  968.96  978.70  996.42 1008.28 1036.74
   1071.82  1090.42  1196.23  1250.67  1253.30  1314.33
   1334.72  1344.73  1434.87  1439.58  1497.38  1502.83
   1549.38  1593.40  1649.32  1657.99  3143.26  3144.99
   3153.73  3174.00  3183.28  3213.00  3232.21  3240.87
==============================
A1
==============================
   9.75   15.35   20.28   22.85   26.31   51.98
   77.81   83.49   87.87   96.53  117.25  153.18
   153.72  170.43  180.47  254.32  261.62  263.62
   270.64  312.72  351.75  377.60  420.94  470.15
   497.74  504.22  517.88  577.50  582.97  634.96
   655.67  669.38  736.75  745.11  790.48  817.14
   840.87  894.97  957.69  958.70 1043.44 1114.71
   1166.81  1190.10  1203.59  1206.40  1226.35  1230.74
   1235.39  1291.05  1317.04  1352.44  1356.39  1474.86
   1480.63  1553.89  1588.70  1684.97  3099.21  3104.82
   3167.54  3179.49  3273.61
==============================
A2
==============================
   2.37   11.52   13.51   23.85   25.89   27.92
   32.25   54.85   60.52   78.66   82.41   88.38
   94.30  100.09  113.12  127.59  138.58  154.46
   159.39  168.72  202.71  225.55  254.01  261.55
   262.74  268.04  282.45  313.74  332.43  351.72
   372.24  379.89  399.89  414.72  469.42  474.36
   500.22  504.01  508.85  517.91  544.84  560.31
   580.92  587.37  631.23  669.70  671.87  705.91
   736.88  737.72  744.92  772.50  790.33  815.16
   817.74  825.01  841.35  851.33  877.99  924.89
   958.73  975.96  994.46 1005.04 1012.57 1050.87
   1070.93  1110.42  1115.96  1137.18  1155.60  1184.09
   1187.25  1202.20  1203.87  1206.51  1227.36  1229.68
   1235.31  1239.29  1288.90  1293.36  1304.26  1339.97
   1359.37  1377.81  1406.43  1442.46  1490.89  1498.69
   1512.17  1529.69  1537.06  1547.98  1585.29  1630.85
   1653.41  1683.92  1806.34  2232.36  3056.10  3069.18
   3112.80  3133.93  3142.17  3179.51  3188.47  3198.01
   3209.71  3234.13  3272.53
==============================
TS(A2-A3)
==============================
   -464.26   6.47    7.32   14.23   26.19   29.60
   32.66   38.42   62.80   69.62   74.27   88.27
   94.75   97.17  104.68  110.37  120.10  136.69
   148.82  168.73  175.43  195.22  247.33  256.93
   262.00  266.48  273.99  287.47  308.55  313.22
   339.18  348.89  357.44  373.76  382.57  415.79
   460.08  465.11  501.44  504.28  518.16  557.69
   578.62  585.24  617.02  627.58  667.58  687.81
   704.28  736.97  745.54  786.11  790.82  793.03
   816.85  825.03  839.21  860.94  867.79  893.30
   954.65  962.99  985.51  995.97 1012.54 1016.19
   1054.77  1068.00  1121.52  1127.83  1140.73  1184.33
   1185.70  1201.31  1204.21  1208.20  1219.94  1228.18
   1232.60  1237.74  1286.91  1302.54  1320.16  1343.61
   1362.12  1373.16  1407.75  1443.52  1487.56  1499.24
   1512.08  1529.90  1534.63  1549.36  1588.16  1627.96
   1650.89  1689.44  1780.59  2322.73  3053.79  3064.57
   3107.50  3131.57  3138.42  3175.87  3186.96  3199.57
   3210.36  3228.11  3274.42
==============================
A3
==============================
   12.69   14.48   19.61   21.71   27.91   28.61
   48.60   64.41   71.14   86.93   94.15   96.22
   108.17  121.35  145.06  149.81  158.40  185.66
   198.33  250.44  262.71  262.75  269.14  281.44
   299.57  315.78  323.60  352.66  377.90  381.73
   403.48  447.26  470.24  499.81  504.51  519.32
   568.43  582.64  588.40  617.53  626.67  669.24
   682.18  710.77  738.19  745.60  789.25  794.81
   799.98  819.06  823.82  839.43  856.71  875.05
   931.81  966.95  971.87  993.14  999.02 1012.74
   1021.55  1052.51  1061.29  1127.89  1132.88  1140.53
   1182.54  1183.47  1202.25  1209.17  1212.27  1227.56
   1228.94  1238.02  1266.54  1284.71  1300.88  1346.52
   1364.03  1376.38  1381.82  1410.42  1444.14  1481.84
   1498.46  1511.09  1528.17  1530.42  1545.77  1588.83
   1609.32  1647.01  1690.50  1758.08  3055.34  3061.82
   3105.79  3131.55  3141.28  3188.11  3194.16  3205.11
   3214.66  3225.12  3279.11
==============================
TS(A3-A4)
==============================
   -212.00   4.18    9.61   19.47   21.60   24.03
   28.50   31.79   51.40   59.37   66.49   70.78
   83.56   84.71   86.23   98.78  102.07  113.67
   117.12  124.72  130.33  144.26  165.33  187.42
   200.24  212.96  222.94  248.80  259.52  261.26
   269.39  275.18  309.40  312.03  320.46  334.08
   345.23  351.81  367.66  390.37  413.96  415.27
   419.32  456.48  460.77  488.52  503.10  512.48
   516.85  543.45  575.46  581.56  587.06  601.28
   607.56  629.56  665.28  689.89  695.65  706.15
   735.13  735.97  743.57  756.40  764.39  782.29
   788.62  791.51  815.08  824.34  825.95  831.31
   845.24  854.37  872.44  891.02  899.98  910.74
   939.18  946.99  963.84  973.48  977.48  982.79
   986.68  995.58 1000.30 1014.78 1016.12 1027.83
   1059.82  1069.46  1074.66  1082.44  1121.35  1124.27
   1126.16  1140.07  1184.61  1185.46  1193.65  1196.81
   1197.45  1200.75  1208.00  1224.43  1231.16  1234.82
   1260.52  1263.46  1285.18  1286.59  1299.67  1312.86
   1331.79  1339.23  1361.50  1371.84  1385.77  1403.30
   1421.32  1432.87  1440.85  1473.28  1482.51  1491.91
```

1499.53 1511.65 1531.20 1532.40 1553.62 1560.99
1589.02 1593.38 1620.73 1632.76 1648.30 1649.52
1694.44 1740.42 3050.14 3058.10 3100.89 3126.48
3135.08 3159.31 3165.66 3174.49 3177.33 3182.47
3187.98 3191.81 3196.33 3203.23 3232.39 3236.35
3247.24 3274.84 3276.09

==============================

A4

==============================

7.17 18.25 21.52 22.03 29.19 32.24
35.58 45.68 56.59 61.71 64.82 75.87
85.77 90.05 96.87 104.97 116.66 121.59
127.86 146.18 154.93 162.43 183.05 198.78
209.63 225.40 245.29 246.82 261.80 271.14
273.79 298.86 309.78 314.55 327.02 337.67
365.88 371.26 386.46 405.53 418.18 426.62
436.97 442.57 487.95 503.73 507.08 516.52
519.82 570.36 575.15 585.30 589.56 624.82
635.02 662.69 676.80 691.08 720.93 728.91
732.51 744.87 750.99 768.27 778.80 788.64
789.35 802.33 811.31 824.14 825.22 832.07
861.83 870.77 889.82 909.16 913.97 938.46
955.12 962.17 975.48 979.79 982.97 999.09
1004.13 1013.15 1020.19 1036.77 1051.49 1058.79
1061.63 1070.45 1089.27 1115.23 1124.62 1130.31
1141.57 1147.82 1165.39 1173.84 1184.12 1185.11
1195.23 1202.79 1224.56 1229.71 1234.79 1237.70
1242.29 1266.87 1272.18 1282.39 1286.33 1297.19
1309.11 1329.55 1357.39 1360.10 1375.28 1402.54
1415.24 1440.36 1466.02 1477.53 1483.86 1486.04
1497.89 1512.16 1531.24 1537.57 1555.34 1573.23
1583.55 1599.21 1624.69 1627.61 1641.49 1653.47
1690.79 1694.72 3047.28 3051.68 3066.15 3088.15
3127.05 3135.81 3165.01 3167.53 3171.72 3179.92
3180.93 3187.37 3194.09 3194.41 3201.42 3221.37
3231.81 3254.46 3270.65

==============================

TS(A4-A5)

==============================

-839.00 9.61 15.66 20.14 23.72 27.33
28.97 37.82 48.13 49.35 60.32 62.86
74.29 83.77 89.05 96.89 101.41 120.59
130.82 136.45 140.34 149.01 157.51 180.09
189.60 218.73 232.86 252.42 260.69 261.03
271.37 294.91 311.96 312.15 322.68 336.72
347.32 368.72 385.84 411.68 416.15 417.67
432.58 461.63 488.18 494.59 503.42 516.94
525.76 556.16 576.48 579.49 584.50 597.39
634.45 666.44 677.36 680.24 698.58 710.96
733.32 735.83 743.24 755.89 764.71 776.88
786.36 789.89 797.64 815.36 826.15 832.53
836.74 853.22 855.67 896.04 903.65 913.58
924.96 962.14 968.34 974.85 985.80 991.05
997.33 1010.12 1022.67 1024.12 1025.50 1034.32
1067.68 1072.97 1082.04 1116.80 1121.44 1129.02
1139.31 1157.55 1183.65 1186.93 1196.48 1197.52
1204.26 1211.20 1224.25 1230.88 1235.18 1258.86
1267.63 1280.32 1287.29 1300.96 1303.32 1325.80
1328.89 1352.30 1358.30 1361.36 1375.83 1418.47
1433.87 1438.18 1449.08 1471.52 1488.51 1489.58
1498.46 1513.21 1528.81 1536.42 1557.68 1564.38
1588.36 1590.91 1598.68 1621.38 1638.58 1648.98
1654.63 1692.38 1722.48 3053.71 3063.42 3108.91
3130.15 3140.36 3160.58 3167.65 3168.56 3175.65
3178.14 3187.25 3189.11 3194.17 3201.48 3219.60
3239.33 3254.27 3272.54

==============================

A5

==============================

11.23 13.33 15.93 20.51 23.06 36.23
40.53 45.37 53.22 63.91 78.58 83.91
85.71 93.48 95.01 106.78 120.92 129.92
137.99 148.98 163.78 173.00 176.02 187.57
214.00 226.71 251.58 261.53 264.51 291.30
297.18 310.33 312.33 326.66 328.46 350.51
371.43 374.19 377.09 410.52 413.99 425.29
454.81 465.04 485.28 498.88 502.85 516.33
522.62 563.86 566.59 578.54 584.79 591.78
619.25 635.13 653.90 666.92 680.65 702.76
708.65 719.72 734.98 740.18 744.16 765.73
771.99 782.50 790.24 799.33 815.06 830.19
835.35 838.55 858.59 880.45 886.21 916.47
925.64 935.06 961.68 970.72 977.77 985.91
997.48 999.90 1004.49 1012.16 1016.58 1026.81
1055.51 1065.66 1079.92 1085.21 1115.62 1131.39
1136.83 1179.54 1185.57 1188.34 1198.46 1202.09
1207.28 1210.72 1226.20 1229.34 1240.03 1253.15
1258.36 1261.67 1286.74 1310.91 1330.09 1337.03
1338.69 1359.84 1364.19 1370.94 1389.37 1425.79
1438.23 1446.20 1448.33 1469.58 1486.66 1494.29
1500.95 1508.25 1526.06 1532.78 1554.40 1556.27
1586.09 1587.95 1600.47 1623.59 1645.71 1652.58
1655.91 1692.40 3058.75 3077.42 3126.00 3145.28
3149.40 3155.35 3164.46 3170.31 3172.47 3178.61
3179.81 3186.27 3199.01 3199.24 3207.29 3242.29
3261.13 3271.53 3658.49

==============================

A6

==============================

9.43 16.60 20.45 22.66 23.28 24.58
26.36 27.54 30.20 32.94 37.26 39.63
40.43 43.79 45.34 48.88 52.47 55.50
60.80 65.09 68.33 72.08 76.44 78.76
82.57 84.36 86.94 92.92 94.02 96.41
97.96 103.67 106.62 111.50 117.59 118.76
120.81 126.69 129.18 138.18 143.17 145.98
147.20 153.58 156.76 158.85 162.27 166.46
175.99 192.71 193.92 200.46 204.42 215.47
220.30 238.86 250.67 255.77 260.25 260.99
264.83 266.76 268.63 285.13 290.64 296.82
299.56 310.38 312.61 314.49 315.43 320.27
332.94 342.03 347.81 350.43 354.46 365.39
370.16 375.40 378.00 388.44 397.01 411.86
416.58 421.26 423.75 430.15 432.81 455.23
460.00 463.22 464.61 465.96 474.61 481.88
495.41 495.66 501.99 503.84 515.66 517.12
522.44 531.63 541.85 549.05 557.40 577.18
578.17 582.77 584.85 591.98 597.96 615.88
628.53 634.15 638.58 654.14 665.45 668.01
672.11 673.74 686.18 700.46 704.66 711.48
712.76 723.42 732.60 733.75 736.40 740.23
741.77 744.26 745.07 769.12 771.91 774.13
775.84 786.55 790.31 790.67 791.94 811.62
812.17 815.53 818.21 829.17 836.00 838.39
839.83 841.51 848.68 869.92 873.53 879.80
887.63 889.00 891.16 913.70 917.18 933.84
937.57 949.04 951.54 961.20 961.24 966.01
967.66 980.07 986.90 988.58 993.15 1001.55
1005.97 1007.62 1008.61 1008.94 1009.41 1015.51
1016.62 1025.06 1026.78 1029.37 1040.14 1059.75
1063.53 1064.81 1070.45 1076.46 1077.27 1094.76
1106.75 1112.81 1113.72 1125.70 1130.85 1132.55
1141.54 1171.65 1177.84 1179.33 1184.03 1185.13
1187.39 1190.22 1194.01 1195.52 1198.40 1199.98
1200.74 1206.86 1222.16 1225.37 1226.66 1228.04
1230.89 1238.09 1238.76 1242.16 1253.66 1254.01
1258.94 1260.25 1280.94 1284.89 1286.74 1311.68
1315.07 1318.23 1323.61 1334.37 1335.99 1342.34
1346.12 1355.66 1357.44 1360.15 1363.85 1364.60
1368.80 1379.48 1393.14 1411.24 1415.23 1429.53

1434.24 1445.03 1446.65 1452.36 1455.68 1468.80
1472.32 1485.37 1487.97 1495.09 1496.76 1497.28
1502.17 1512.29 1515.52 1524.44 1530.00 1533.24
1535.58 1538.87 1549.92 1551.67 1554.46 1573.65
1581.28 1582.53 1586.66 1588.09 1611.53 1627.34
1629.35 1641.93 1649.19 1652.80 1653.47 1656.37
1676.15 1687.49 1690.54 3054.73 3057.08 3063.90
3067.57 3118.23 3122.31 3139.42 3144.84 3149.35
3151.18 3151.21 3151.60 3152.39 3156.56 3158.07
3165.64 3174.92 3177.63 3180.85 3184.33 3186.69
3189.94 3192.93 3197.65 3201.58 3202.05 3203.14
3214.04 3221.05 3224.60 3230.18 3237.15 3242.74
3244.76 3269.71 3271.66 3644.20 3734.58

================================

TS(A6-A7)

================================

-1457.92 7.68 15.49 18.43 19.07 22.69
24.61 25.00 27.98 29.46 31.34 31.67
33.77 36.08 39.88 45.55 49.41 52.74
53.45 60.72 62.66 68.45 73.11 73.94
78.36 79.23 82.97 89.71 91.17 94.98
98.40 103.43 107.26 111.49 112.80 115.57
117.64 120.34 123.46 136.43 140.23 141.40
145.21 148.23 150.51 156.71 159.18 163.13
177.15 179.38 180.54 199.08 200.25 204.79
212.20 225.96 240.23 247.89 253.11 258.04
259.86 263.17 265.27 268.19 273.61 281.57
291.13 293.88 299.05 308.46 312.44 313.42
313.78 327.85 328.19 336.43 339.03 346.39
347.47 368.81 371.71 374.28 383.95 389.28
403.58 417.20 421.42 424.19 428.67 435.85
451.37 455.47 462.25 464.13 478.06 479.09
486.21 489.96 496.83 503.31 504.51 516.47
517.16 530.13 537.02 566.78 573.71 575.05
581.62 583.77 592.84 595.39 598.01 626.41
631.90 637.64 638.77 666.07 666.55 667.40
671.54 690.04 695.04 704.65 712.09 713.05
724.12 727.23 734.38 735.15 743.85 744.17
746.96 748.97 756.41 763.00 773.35 776.24
780.98 785.66 789.20 790.45 794.38 803.37
804.75 813.77 814.35 815.81 830.77 834.24
836.50 839.39 853.98 856.01 858.78 879.13
883.17 885.58 890.13 893.12 917.75 920.57
927.91 935.85 945.26 946.37 960.09 960.17
965.27 971.85 975.29 976.71 984.59 991.98
994.08 994.15 1003.19 1007.47 1012.16 1013.41
1016.15 1016.50 1019.19 1021.81 1022.47 1041.41
1052.67 1055.64 1066.97 1069.03 1074.12 1089.02
1092.28 1094.84 1109.53 1111.15 1129.88 1132.37
1134.96 1139.58 1169.44 1175.98 1185.05 1187.04
1188.99 1189.16 1195.72 1196.21 1197.14 1199.24
1201.11 1204.55 1225.12 1226.50 1230.04 1231.26
1233.85 1237.03 1241.94 1242.84 1250.76 1259.22
1261.29 1264.32 1276.81 1286.32 1288.58 1300.49
1312.65 1319.84 1324.39 1333.02 1337.77 1341.47
1343.73 1348.04 1353.91 1355.08 1356.25 1368.44
1370.17 1377.67 1393.04 1406.95 1420.08 1425.34
1436.53 1438.66 1444.31 1448.45 1453.29 1462.64
1468.01 1476.36 1484.06 1491.11 1493.10 1493.41
1496.93 1498.74 1508.02 1509.60 1513.83 1520.48
1532.46 1536.64 1541.27 1545.61 1554.67 1557.43
1560.34 1567.15 1580.42 1581.80 1582.99 1585.82
1609.58 1627.06 1633.54 1636.70 1638.80 1648.00
1652.91 1656.20 1661.80 1688.99 1691.43 3048.94
3056.30 3060.56 3088.37 3120.17 3131.08 3138.91
3144.00 3149.32 3149.47 3156.00 3160.44 3161.01
3170.78 3171.19 3174.18 3179.43 3180.76 3180.87
3183.39 3190.02 3191.69 3195.65 3195.85 3196.99
3197.11 3207.69 3208.75 3215.72 3218.48 3222.80
3245.96 3253.46 3268.73 3271.26 3296.37 3356.91

================================

TS(A4-A7)

================================

-1296.32 9.46 11.85 16.63 25.94 33.27
37.47 39.99 44.95 55.16 62.66 70.45
71.75 80.17 89.04 97.95 103.18 111.98
120.21 140.32 154.08 164.47 166.42 182.70
196.44 210.34 235.43 249.25 254.58 259.68
270.41 290.30 299.72 313.96 316.72 321.85
346.63 370.40 373.47 393.43 415.95 421.17
426.58 436.44 458.81 492.49 503.66 508.44
516.88 531.09 565.00 575.12 582.13 592.30
627.10 638.26 656.15 665.45 695.20 712.03
726.68 733.35 733.96 746.66 758.66 775.22
777.51 790.82 791.95 812.77 813.83 823.01
835.59 842.06 864.83 876.89 891.56 908.97
938.84 948.22 958.33 960.52 977.80 984.78
988.30 995.78 1012.93 1013.51 1016.42 1026.25
1059.79 1061.28 1075.88 1103.09 1113.87 1115.46
1134.41 1135.51 1167.60 1181.47 1188.60 1190.92
1197.18 1199.31 1222.01 1226.68 1229.35 1236.26
1238.46 1262.97 1270.92 1286.45 1298.18 1308.32
1332.41 1337.96 1355.98 1359.02 1372.59 1402.66
1413.59 1439.45 1440.88 1466.14 1483.65 1491.82
1499.71 1520.84 1534.23 1535.79 1547.59 1567.30
1585.48 1585.76 1630.51 1633.53 1651.40 1651.94
1686.97 1735.35 1854.96 3048.12 3051.02 3111.24
3126.23 3148.12 3157.90 3164.33 3173.57 3179.52
3183.55 3189.18 3194.79 3202.67 3210.98 3227.74
3244.09 3246.22 3270.12

================================

A7

================================

4.40 13.30 19.36 23.22 24.07 30.77
32.17 36.50 47.25 52.47 60.49 64.16
74.99 82.58 89.29 91.22 106.32 116.56
144.71 148.26 154.43 167.97 172.88 182.28
200.84 246.71 258.88 260.65 263.72 277.33
281.00 301.66 311.34 324.88 336.21 352.19
360.97 377.37 378.23 403.59 417.75 429.00
468.29 473.22 488.90 499.95 503.39 514.66
516.96 579.60 585.37 591.10 608.14 626.87
639.29 662.12 667.64 702.88 716.07 728.53
736.08 742.67 743.18 753.30 763.30 778.48
789.39 801.25 816.71 820.33 825.07 836.70
864.23 866.70 881.15 897.09 900.22 908.54
941.73 961.25 966.33 974.37 981.50 986.01
1008.79 1011.53 1017.59 1022.21 1030.34 1057.67
1063.14 1064.66 1089.67 1117.25 1126.17 1140.81
1184.91 1185.69 1188.63 1188.86 1200.55 1207.14
1209.85 1217.21 1225.79 1232.74 1242.14 1250.51
1263.65 1286.29 1287.49 1299.53 1305.04 1325.97
1343.86 1347.20 1359.64 1370.66 1375.88 1402.27
1429.07 1440.19 1448.87 1462.01 1491.38 1493.35
1499.18 1513.00 1530.61 1541.58 1550.06 1554.26
1580.65 1587.56 1638.34 1640.22 1647.99 1660.97
1689.84 1822.63 3052.54 3062.34 3065.55 3103.36
3130.14 3135.52 3155.42 3160.81 3173.79 3177.38
3187.31 3188.56 3198.86 3200.75 3215.71 3227.63
3229.14 3230.06 3271.47

================================

TS(A4-A8)

================================

-98.68 10.26 14.38 21.31 23.45 33.70
38.89 51.24 59.45 63.23 67.62 73.33
79.77 85.19 87.72 97.92 104.92 112.02
134.73 142.28 149.52 158.85 180.06 198.14
209.05 220.24 235.15 255.14 262.82 265.16
279.56 289.99 313.55 333.66 342.86 351.28
363.78 370.90 377.85 408.42 414.73 434.81

464.85 469.18 495.35 499.48 503.46 517.01
517.61 560.76 579.75 585.35 591.77 598.42
634.73 665.88 668.42 669.34 711.24 732.63
736.29 745.08 747.55 771.96 779.65 789.60
791.61 811.35 816.81 819.10 826.87 831.55
838.97 854.18 877.57 885.25 901.11 918.80
931.99 939.74 943.20 962.72 969.59 975.11
997.03 1006.04 1014.18 1018.02 1021.80 1038.15
1050.93 1067.94 1068.59 1096.09 1118.40 1120.41
1141.11 1148.94 1173.23 1183.80 1187.32 1194.98
1197.73 1203.29 1224.87 1227.08 1236.74 1239.86
1261.48 1268.93 1282.34 1287.71 1300.63 1305.38
1311.62 1334.29 1340.95 1354.08 1362.31 1366.78
1404.15 1435.97 1440.96 1455.36 1484.20 1497.52
1497.87 1514.30 1530.28 1538.66 1551.54 1570.00
1590.16 1598.10 1633.75 1655.54 1657.85 1691.02
1700.63 1755.08 3054.19 3057.49 3107.89 3126.51
3152.63 3152.87 3157.56 3160.89 3164.81 3166.90
3173.94 3175.52 3178.24 3186.33 3187.90 3195.87
3205.80 3251.52 3273.91

==============================

A8

==============================

2.38 12.32 25.04 27.01 29.29 36.17
40.41 47.14 57.62 63.22 73.31 89.56
93.65 100.81 101.12 109.15 110.80 136.74
141.52 156.91 164.11 165.69 179.06 208.09
220.08 240.95 257.88 260.83 263.78 267.67
276.31 296.28 314.59 329.26 351.25 357.54
369.81 377.65 381.71 410.39 428.42 434.38
467.12 480.85 498.32 504.80 504.86 518.17
554.18 572.44 578.18 582.88 595.88 623.34
656.50 668.09 668.64 708.61 724.95 735.96
738.54 746.50 759.76 774.32 787.82 792.99
793.71 801.81 817.05 820.78 837.46 839.28
862.25 866.43 869.47 881.35 897.26 914.35
927.84 934.40 947.34 959.41 960.72 973.07
988.13 1000.55 1001.28 1014.25 1019.67 1026.31
1036.59 1060.47 1093.24 1095.59 1104.86 1118.27
1142.78 1145.91 1172.77 1183.96 1185.72 1188.45
1195.80 1200.32 1207.35 1233.66 1233.84 1240.11
1268.62 1275.22 1289.03 1290.76 1301.69 1326.15
1333.02 1342.32 1348.35 1360.27 1362.40 1365.55
1406.67 1433.67 1442.25 1452.52 1470.02 1495.47
1500.16 1513.53 1520.54 1530.13 1552.56 1589.09
1592.96 1597.04 1619.81 1644.45 1663.61 1689.85
1707.17 1789.56 3061.25 3063.88 3106.02 3136.97
3148.48 3151.75 3158.79 3161.47 3172.66 3181.92
3184.00 3191.02 3194.58 3204.98 3207.08 3210.09
3219.96 3220.59 3273.19

==============================

TS(A8-A9)

==============================

-556.52 7.81 12.13 17.07 25.15 30.37
36.80 42.13 50.64 67.43 68.97 76.16
90.62 100.43 104.84 113.55 117.38 140.41
148.52 153.45 157.35 169.04 179.46 200.34
206.14 219.05 244.75 254.54 260.01 266.81
291.81 307.61 311.13 313.64 315.13 349.08
363.97 375.65 379.74 386.28 423.38 433.48
438.43 464.25 472.11 497.54 505.15 518.21
519.36 554.27 575.13 576.76 584.32 598.88
621.16 648.05 668.52 672.87 697.12 714.97
728.18 737.02 738.65 747.19 751.36 752.38
769.49 790.25 793.52 801.78 817.18 831.21
837.65 838.86 864.99 876.72 878.96 886.15
906.58 928.23 961.56 968.70 971.15 976.10
989.76 996.45 998.52 1005.22 1018.10 1038.16
1045.04 1060.48 1099.69 1105.63 1114.74 1117.28
1141.36 1148.34 1181.74 1187.39 1193.06 1195.58
1203.14 1204.15 1226.39 1232.47 1241.12 1248.53
1268.51 1280.74 1284.63 1288.07 1307.29 1309.49
1337.04 1340.41 1360.60 1363.14 1382.80 1409.34
1412.61 1441.10 1444.70 1464.11 1469.63 1478.20
1499.61 1514.49 1519.55 1524.56 1530.14 1556.72
1569.15 1587.54 1620.78 1632.49 1634.31 1649.75
1691.42 1815.45 3057.46 3088.83 3125.60 3140.72
3145.58 3146.48 3156.23 3163.44 3173.48 3182.13
3183.45 3189.30 3190.99 3195.87 3203.77 3204.84
3208.32 3215.10 3273.63

==============================

A9

==============================

7.06 13.59 17.66 23.55 25.61 31.83
40.07 50.43 58.68 66.95 79.15 86.54
87.07 94.69 101.71 115.19 125.11 130.56
146.03 156.40 161.36 172.29 184.95 207.06
230.94 241.63 250.71 261.66 264.04 274.00
294.14 304.23 312.18 319.77 328.96 351.06
374.52 380.18 392.22 408.19 429.18 443.95
460.58 467.00 468.85 489.78 496.91 504.15
517.58 526.40 577.59 577.96 583.04 591.99
626.11 632.51 664.05 668.20 687.52 712.68
734.24 738.99 742.49 744.91 762.99 776.80
788.02 790.59 803.81 811.78 815.28 837.72
851.85 854.84 869.69 877.01 878.89 904.64
931.89 940.47 951.23 959.86 967.79 982.02
997.74 1001.47 1004.77 1007.91 1009.47 1014.54
1046.12 1048.20 1093.53 1103.35 1113.04 1129.18
1142.35 1170.43 1174.36 1179.73 1188.46 1196.53
1206.08 1206.35 1212.42 1230.08 1240.91 1245.92
1266.81 1285.20 1287.46 1290.72 1303.68 1333.63
1335.15 1358.60 1361.61 1369.77 1401.61 1431.06
1434.03 1441.70 1467.85 1472.61 1499.45 1502.47
1505.66 1516.19 1528.88 1556.30 1585.91 1588.14
1607.50 1633.08 1641.24 1648.06 1686.58 1690.55
1718.92 1818.79 3063.90 3085.36 3137.61 3144.80
3146.85 3156.02 3160.34 3167.70 3171.04 3173.07
3184.71 3186.96 3192.80 3195.41 3198.86 3204.10
3206.83 3216.82 3271.60

==============================

B1

==============================

47.93 56.03 127.12 193.20 193.59 209.09
299.50 326.71 354.76 486.31 519.05 550.56
570.11 607.72 753.47 947.90 1061.50 1170.35
1231.58 1264.67 1310.25

==============================

B2

==============================

9.41 27.14 33.60 40.77 50.73 58.45
63.31 67.34 78.39 82.97 88.33 112.81
130.91 136.27 148.30 183.07 196.55 199.21
223.11 232.07 279.15 298.46 302.94 331.48
348.31 352.75 354.63 404.52 423.39 480.53
494.49 515.65 518.40 554.25 555.92 561.51
571.19 609.82 629.69 677.17 713.00 737.26
750.97 774.21 808.98 836.09 867.94 874.09
926.24 953.33 979.75 1003.05 1005.61 1010.83
1046.55 1073.70 1110.82 1133.62 1139.00 1169.27
1187.36 1200.47 1224.88 1235.20 1237.31 1251.36
1302.54 1305.88 1325.02 1346.14 1367.62 1380.63
1410.47 1440.40 1480.29 1498.08 1513.68 1523.92
1532.09 1611.52 1643.27 1732.95 2233.24 3054.83
3073.76 3125.17 3150.71 3165.78 3190.94 3196.81
3207.29 3217.64 3227.66

==============================

TS(B2-B3)

==============================

-410.52 5.96 13.08 19.44 32.55 40.21

44.58 53.57 63.38 65.04 78.12 81.18
93.79 96.72 109.96 146.46 173.50 191.57
197.17 204.84 245.06 265.25 278.93 298.17
303.84 313.16 328.09 343.21 355.50 380.42
412.26 460.90 490.77 521.24 553.64 561.11
574.11 597.20 609.38 625.86 673.62 699.29
750.89 782.81 791.60 827.05 857.92 867.26
880.67 945.59 958.55 987.49 995.60 1015.04
1018.60 1048.59 1061.48 1122.60 1125.45 1138.92
1175.16 1184.61 1202.12 1220.26 1231.68 1237.67
1248.88 1307.09 1308.07 1338.77 1342.63 1371.01
1411.84 1445.21 1486.24 1499.89 1512.93 1528.11
1530.74 1623.65 1647.97 1779.98 2366.13 3050.32
3068.86 3114.28 3127.60 3139.30 3181.31 3189.71
3201.78 3211.81 3220.17

==============================

B3

==============================

5.51 20.16 24.09 34.90 45.05 50.77
55.48 62.46 77.18 84.02 96.94 121.91
147.87 156.78 195.02 200.69 202.55 254.28
260.94 275.22 298.76 321.76 330.89 356.94
375.04 391.86 435.84 491.74 523.65 548.73
554.31 578.73 602.17 610.95 614.62 670.82
688.87 751.97 787.47 800.75 823.51 857.86
868.59 902.63 944.54 987.69 998.03 1007.47
1010.49 1034.43 1046.34 1054.32 1127.18 1135.67
1139.57 1175.92 1181.39 1206.53 1231.07 1234.29
1252.89 1280.26 1301.76 1310.11 1348.08 1391.88
1394.79 1424.03 1447.85 1477.27 1497.91 1511.25
1528.00 1532.44 1601.18 1647.98 1758.49 3059.13
3066.96 3114.20 3137.73 3144.67 3183.81 3192.55
3207.57 3220.11 3224.12

==============================

TS(B3-B4)

==============================

-113.08 3.81 16.09 24.59 34.84 37.71
43.06 52.09 63.19 70.48 73.93 78.04
87.68 90.58 103.44 104.92 117.57 131.75
140.00 173.61 187.44 195.13 203.89 214.92
226.11 254.75 273.51 289.82 306.97 325.76
335.32 338.68 348.77 373.91 383.07 413.90
418.45 421.30 469.02 493.24 509.61 515.42
550.61 555.94 566.05 579.90 590.93 603.98
619.89 623.54 689.10 690.48 696.55 735.94
756.39 757.47 763.73 783.57 789.65 823.81
827.70 842.03 847.23 873.11 881.70 897.58
910.37 915.35 942.56 955.56 971.73 978.87
985.59 992.67 1003.44 1005.89 1011.51 1013.14
1027.40 1053.04 1059.84 1076.39 1082.13 1118.06
1124.77 1127.86 1138.18 1182.43 1197.40 1199.35
1210.81 1218.57 1226.44 1227.26 1238.43 1259.80
1272.36 1287.50 1290.17 1297.51 1332.54 1335.82
1342.97 1374.23 1382.83 1402.38 1426.70 1436.63
1440.81 1478.29 1483.35 1491.31 1498.23 1511.48
1525.13 1529.51 1545.32 1592.89 1608.65 1635.35
1645.72 1650.17 1757.98 3054.34 3059.66 3102.07
3131.22 3138.83 3158.17 3164.52 3176.62 3181.81
3188.38 3193.30 3201.67 3202.99 3211.73 3221.88
3234.35 3247.36 3279.08

==============================

B4

==============================

23.04 27.26 32.08 33.65 38.85 46.29
51.86 56.47 63.42 70.23 71.67 84.39
90.10 101.65 117.26 122.86 139.23 158.68
185.45 197.16 198.45 199.20 215.17 227.32
243.09 274.15 297.17 298.37 313.69 327.35
331.98 351.48 370.75 389.32 405.45 418.16
422.72 437.34 492.88 505.16 518.06 519.41
554.11 570.67 571.43 589.60 609.70 626.58
635.77 675.25 694.95 718.48 727.72 749.22
754.97 767.30 781.37 792.60 804.46 823.95
836.65 861.33 870.63 890.82 909.26 916.67
933.27 955.19 963.50 974.89 977.25 987.42
1004.21 1006.74 1010.10 1017.12 1041.44 1052.06
1058.66 1063.85 1070.91 1091.76 1125.52 1131.30
1139.37 1141.67 1150.79 1162.66 1164.93 1184.88
1196.19 1230.76 1232.86 1238.27 1243.48 1250.46
1264.18 1271.91 1287.54 1298.09 1301.17 1310.79
1328.20 1358.74 1375.76 1403.47 1417.08 1440.76
1478.22 1481.95 1485.46 1486.53 1498.21 1511.47
1532.16 1538.50 1573.69 1607.33 1626.33 1629.92
1645.93 1653.24 1705.09 3048.25 3048.88 3052.59
3089.91 3128.66 3135.04 3167.76 3169.01 3174.06
3178.91 3182.98 3188.75 3189.81 3195.11 3195.95
3202.95 3233.91 3255.76

==============================

TS(B4-B5)

==============================

-965.27 10.48 16.56 26.44 28.18 34.50
41.27 46.01 50.07 55.51 60.00 70.11
74.46 85.82 100.14 109.91 138.84 143.19
148.63 182.21 189.94 198.11 201.14 227.85
232.42 272.87 292.51 294.56 310.11 320.53
327.92 334.60 349.26 377.47 403.83 413.08
415.73 432.25 489.28 491.52 517.49 520.45
544.22 551.92 569.62 579.29 596.20 607.93
632.21 674.83 680.79 684.55 711.06 730.92
746.56 754.29 766.57 775.99 785.57 795.40
825.60 834.45 849.91 854.05 894.53 903.45
918.67 921.45 951.91 970.26 973.58 983.66
988.00 993.92 1011.73 1021.09 1024.97 1025.29
1036.63 1067.32 1073.65 1079.64 1108.71 1130.44
1136.23 1138.71 1154.61 1157.12 1183.04 1198.47
1208.28 1231.98 1233.65 1247.39 1256.83 1269.47
1276.48 1301.11 1305.81 1307.22 1327.18 1332.35
1353.77 1359.00 1376.82 1418.55 1429.83 1440.67
1448.88 1473.10 1488.66 1490.96 1498.32 1511.56
1528.41 1538.40 1564.48 1591.94 1611.30 1627.24
1638.63 1648.65 1657.37 1707.27 3056.74 3065.50
3110.40 3135.38 3141.95 3164.47 3171.18 3174.26
3180.57 3181.00 3188.80 3193.18 3195.59 3205.05
3225.84 3243.89 3247.13

==============================

B5

==============================

5.36 10.91 21.03 24.86 34.98 38.92
43.73 47.57 49.27 58.87 69.02 74.05
83.30 86.20 103.51 114.50 129.64 149.89
171.76 189.55 191.39 202.89 223.65 231.25
274.94 294.62 297.06 305.78 318.26 327.65
328.29 350.93 368.55 374.41 408.85 414.21
424.75 453.98 486.73 489.11 516.59 519.55
551.62 559.38 569.40 581.22 591.87 607.90
619.66 634.02 652.25 682.49 705.05 710.15
718.01 741.08 748.70 765.76 771.77 784.41
802.54 831.51 836.44 859.46 883.13 887.79
917.96 932.13 950.72 952.72 973.19 979.88
987.76 998.77 1002.82 1006.67 1014.51 1018.68
1030.81 1058.64 1065.86 1084.16 1088.77 1120.16
1131.11 1134.81 1158.35 1181.41 1199.53 1204.96
1208.79 1229.01 1232.36 1252.58 1255.65 1261.37
1265.27 1305.62 1308.41 1333.99 1340.48 1340.69
1365.09 1372.42 1390.55 1429.03 1441.64 1445.51
1451.61 1471.88 1488.44 1496.83 1502.44 1511.01
1524.51 1534.73 1554.89 1588.86 1595.87 1628.47
1647.48 1654.31 1656.94 3047.56 3057.44 3112.90
3136.50 3143.73 3151.92 3157.46 3167.93 3180.18
3181.11 3187.41 3190.72 3203.09 3203.30 3212.03

3242.74 3247.46 3639.26
==============================
B6
==============================
8.17 10.26 17.95 19.89 21.40 25.90
26.42 30.02 31.77 34.22 36.41 37.68
38.07 41.32 46.27 49.63 51.34 54.49
60.41 61.27 65.12 66.35 70.77 72.11
80.17 83.43 86.60 93.16 94.63 102.53
104.26 107.48 111.64 116.42 124.81 128.82
140.61 143.37 145.73 146.94 159.11 165.15
175.24 184.00 190.45 197.18 204.08 205.09
206.56 209.61 212.88 216.93 225.37 232.57
268.31 278.65 280.58 293.05 304.68 305.50
314.56 319.08 323.62 327.98 330.28 331.98
336.09 337.46 344.60 347.95 354.62 364.49
379.38 382.82 392.00 416.17 419.60 422.30
429.40 439.25 442.62 452.38 458.84 473.06
474.58 491.12 493.00 504.39 504.49 509.00
534.12 538.86 551.67 555.04 555.67 558.19
558.96 560.27 588.48 591.66 614.55 617.68
618.32 622.43 631.43 632.14 664.63 668.08
682.81 685.33 702.77 703.95 707.38 714.01
726.33 734.95 742.20 746.17 753.26 755.15
765.20 766.78 773.62 775.24 779.05 793.31
796.68 816.20 823.05 827.82 841.31 847.15
862.95 878.68 882.28 883.38 888.77 902.35
911.18 917.14 921.09 927.15 933.51 949.04
969.86 970.50 972.69 979.56 981.27 987.94
989.29 994.96 1001.52 1005.15 1006.97 1009.17
1009.54 1011.18 1012.75 1018.28 1021.49 1024.61
1028.77 1033.89 1055.48 1063.78 1064.12 1065.38
1076.50 1081.60 1091.97 1095.43 1124.79 1126.60
1131.73 1134.30 1138.74 1149.42 1167.32 1181.70
1185.67 1189.66 1196.70 1198.51 1203.11 1204.47
1210.64 1214.18 1217.39 1221.10 1229.32 1232.31
1240.92 1242.17 1247.95 1249.35 1251.27 1259.76
1260.96 1268.48 1269.17 1272.72 1313.18 1321.32
1325.88 1328.42 1330.65 1333.17 1337.61 1343.66
1352.98 1355.17 1371.28 1376.51 1385.42 1390.64
1425.44 1430.08 1432.35 1434.93 1441.48 1443.51
1451.07 1457.98 1463.87 1464.15 1472.20 1484.85
1494.10 1497.49 1499.84 1501.01 1503.97 1521.77
1527.92 1529.36 1529.95 1532.96 1535.69 1541.59
1566.70 1582.40 1584.49 1606.77 1625.01 1629.74
1636.96 1644.58 1648.06 1649.91 1657.61 1660.88
3053.39 3061.05 3063.95 3085.19 3124.17 3130.36
3146.44 3152.73 3154.12 3155.88 3156.10 3159.72
3162.37 3168.17 3169.54 3174.83 3181.67 3181.88
3185.65 3187.85 3189.24 3189.33 3193.25 3198.00
3199.96 3201.35 3206.45 3208.48 3214.76 3220.91
3238.27 3241.36 3256.08 3264.36 3695.66 3717.06
==============================
TS(B6-B7)
==============================
-1275.95 3.00 13.62 20.74 24.87 29.25
31.73 34.56 36.28 36.57 38.09 41.76
46.09 47.97 49.42 51.71 59.91 61.23
62.67 68.89 71.82 77.30 78.00 81.25
85.99 87.87 94.46 98.96 99.88 103.01
107.08 110.46 115.93 119.63 131.76 136.91
141.81 149.94 153.68 158.89 162.31 176.66
180.84 183.07 193.05 195.09 196.92 205.24
208.93 213.98 218.55 220.91 225.92 236.55
266.16 284.69 286.55 292.78 296.75 300.85
303.41 308.66 316.75 326.25 333.03 333.31
336.61 339.09 339.66 344.74 346.37 373.97
380.02 388.07 400.55 416.38 419.80 425.16
430.82 435.45 448.41 454.14 468.06 487.54
492.43 496.47 499.94 502.55 506.55 528.88
536.62 554.16 556.66 559.28 560.88 576.29
591.21 594.19 599.45 612.75 615.67 627.16
628.84 637.38 638.20 670.46 671.70 685.86
699.95 708.69 712.79 720.17 731.25 740.28
743.73 748.20 750.56 750.99 756.32 760.44
770.04 775.21 782.86 785.55 795.33 799.91
814.64 823.46 838.63 848.08 849.18 853.60
858.27 881.74 884.09 891.75 893.26 898.38
915.65 919.07 924.36 931.43 951.15 959.69
963.62 972.03 972.21 974.13 985.63 986.96
989.61 998.39 999.42 1005.98 1007.22 1009.18
1012.81 1016.10 1016.22 1017.85 1020.70 1021.19
1027.07 1036.41 1051.63 1057.05 1066.88 1067.73
1073.89 1079.31 1081.04 1098.64 1128.95 1135.06
1139.11 1139.97 1144.20 1159.83 1173.32 1177.86
1183.06 1190.42 1195.12 1198.07 1199.54 1206.09
1209.57 1214.81 1229.30 1230.35 1236.08 1246.66
1247.45 1249.11 1251.22 1254.62 1260.65 1262.15
1273.11 1276.08 1279.96 1310.80 1312.48 1316.41
1318.73 1327.53 1336.37 1340.59 1343.58 1351.19
1353.54 1361.55 1373.92 1378.38 1387.03 1400.68
1420.96 1427.44 1430.10 1433.94 1440.18 1449.98
1456.02 1458.45 1461.52 1478.22 1484.03 1491.05
1494.27 1498.56 1498.61 1498.95 1511.81 1515.26
1521.97 1523.67 1535.23 1537.00 1539.87 1540.76
1549.52 1575.95 1583.96 1586.20 1620.87 1628.53
1631.91 1639.91 1644.77 1647.65 1647.97 1652.83
1657.10 3049.78 3055.22 3064.07 3091.94 3121.54
3133.72 3146.50 3147.33 3153.29 3154.97 3159.17
3163.49 3164.88 3166.55 3169.60 3170.68 3178.07
3178.58 3180.72 3183.49 3184.36 3185.55 3190.07
3196.26 3200.78 3208.62 3212.18 3217.44 3224.10
3229.14 3237.54 3242.21 3247.13 3258.64 3424.46
==============================
B7
==============================
8.58 18.52 23.29 30.54 41.92 44.81
52.64 58.59 60.41 64.86 71.18 73.79
80.61 89.46 98.70 117.89 141.92 163.58
176.72 188.95 195.22 203.19 229.86 241.13
257.92 274.97 295.86 297.77 321.39 324.52
340.70 349.20 369.89 397.56 419.06 435.18
448.23 454.47 488.20 506.02 514.42 519.34
552.51 564.73 583.14 598.20 611.02 623.06
634.10 674.91 697.74 713.05 734.53 738.48
751.57 758.36 777.08 788.32 795.49 802.42
827.88 864.01 865.76 868.61 886.85 899.51
909.69 930.35 944.30 965.35 974.28 982.98
989.54 1006.87 1011.82 1015.33 1021.82 1027.93
1042.55 1053.88 1073.93 1079.38 1107.32 1118.88
1121.96 1174.59 1194.25 1197.12 1202.56 1205.24
1212.60 1218.87 1225.75 1244.21 1249.25 1264.34
1279.41 1291.57 1301.35 1323.99 1336.55 1344.35
1352.13 1365.82 1374.77 1407.66 1418.76 1436.59
1451.08 1463.63 1494.78 1497.11 1501.48 1506.76
1513.90 1535.28 1543.06 1580.63 1641.02 1641.35
1654.71 1657.75 1826.31 3055.46 3061.45 3101.76
3125.72 3155.15 3156.92 3163.11 3175.38 3178.79
3179.96 3185.71 3190.90 3197.46 3207.29 3213.92
3222.85 3237.23 3249.71
==============================
TS(B4-B8)
==============================
-143.24 4.35 10.02 19.16 24.83 33.42
43.55 47.40 51.22 64.76 70.92 74.32
77.61 80.57 93.72 103.05 127.03 141.57
152.34 180.76 195.00 198.41 212.62 212.94
219.06 277.00 298.82 302.20 320.67 324.44
342.42 352.86 362.58 374.78 408.80 413.64
434.41 460.85 488.10 495.21 510.90 514.55

532.59 553.39 564.73 588.87 594.97 613.66
633.96 667.49 671.02 711.20 733.36 746.52
754.68 770.53 777.19 791.04 810.23 819.35
826.77 839.08 851.93 879.54 884.82 901.44
920.20 931.43 946.99 949.12 961.62 968.90
979.64 998.98 1011.77 1013.24 1016.01 1026.26
1036.69 1057.75 1066.57 1082.65 1095.64 1099.07
1117.43 1139.35 1151.57 1179.10 1191.35 1197.79
1199.87 1220.39 1223.31 1240.59 1243.28 1251.50
1270.67 1284.82 1295.97 1301.94 1308.35 1323.64
1338.00 1345.78 1361.37 1368.57 1406.02 1437.93
1444.10 1460.51 1483.12 1496.93 1502.63 1518.00
1532.11 1536.13 1561.22 1586.48 1631.51 1650.36
1656.24 1693.98 1755.97 3042.67 3044.49 3097.39
3121.05 3138.87 3146.15 3153.88 3158.78 3166.07
3167.71 3173.97 3176.03 3185.92 3186.98 3194.21
3206.35 3213.81 3232.08

===============================

B8

===============================

21.98 25.13 26.78 33.01 35.82 45.87
47.87 49.13 63.19 66.48 77.13 83.60
87.54 104.52 109.16 130.05 132.79 152.66
181.43 197.14 204.33 218.14 240.31 243.45
275.43 282.82 296.39 312.14 330.71 335.29
349.66 367.16 383.34 387.13 415.91 432.04
442.37 494.87 494.94 500.16 505.30 540.35
556.59 559.64 573.32 595.84 621.05 630.70
659.07 663.56 723.68 725.23 739.46 753.65
760.08 768.32 787.90 789.82 797.07 819.47
830.52 865.30 869.52 876.49 883.85 891.72
910.61 944.31 949.32 959.01 968.15 970.06
998.90 1006.75 1013.29 1014.23 1019.95 1023.42
1037.07 1051.31 1066.33 1094.53 1099.76 1109.92
1139.48 1143.25 1154.74 1179.43 1185.93 1194.79
1212.79 1219.80 1231.23 1237.41 1242.74 1261.63
1269.42 1290.52 1297.54 1313.11 1328.84 1332.41
1347.55 1361.58 1363.49 1374.88 1414.23 1440.33
1447.14 1459.17 1478.71 1498.80 1501.84 1511.49
1522.85 1538.56 1591.16 1612.24 1627.75 1650.92
1664.97 1677.34 1706.23 3054.67 3073.53 3123.44
3133.07 3146.39 3147.39 3158.11 3170.97 3181.17
3181.63 3189.97 3192.06 3192.83 3198.11 3203.89
3210.69 3222.39 3235.40

===============================

TS(B8-B9)

===============================

-557.77 15.86 21.47 28.56 36.05 44.20
46.71 48.59 54.66 63.54 69.97 77.91
83.09 88.03 107.33 120.21 127.26 139.72
168.79 177.42 191.67 204.23 213.81 244.97
253.72 284.65 287.31 309.70 315.12 326.84
332.01 360.75 373.42 384.46 396.02 424.24
427.92 435.50 490.29 494.13 497.00 520.15
552.37 556.89 558.61 567.12 598.27 621.90
628.96 647.72 677.89 704.00 724.48 728.97
745.39 753.56 760.41 766.92 768.63 797.04
803.78 829.71 843.20 859.40 875.27 883.12
887.52 906.67 949.62 969.83 971.55 979.97
995.54 999.96 1007.02 1011.00 1011.82 1022.41
1045.37 1052.82 1064.63 1108.02 1109.22 1125.53
1142.01 1143.54 1153.99 1185.95 1194.76 1213.00
1220.25 1231.59 1234.74 1241.24 1252.58 1261.88
1277.70 1284.45 1300.19 1311.09 1336.62 1341.38
1350.00 1364.65 1386.43 1411.67 1413.89 1445.11
1453.54 1467.36 1479.96 1483.25 1498.42 1511.56
1522.49 1524.70 1534.18 1571.30 1628.50 1634.54
1648.27 1652.34 1716.68 3055.71 3073.63 3126.71
3137.06 3143.34 3152.79 3153.14 3169.87 3175.41
3177.06 3181.68 3187.07 3187.54 3191.26 3198.85
3201.07 3209.52 3243.74

===============================

B9

===============================

9.16 17.21 20.65 22.93 32.45 38.91
49.05 50.97 60.83 65.05 71.88 79.71
83.21 90.35 103.66 113.23 133.83 156.48
169.45 182.02 200.63 219.85 239.42 254.92
269.89 273.82 293.66 310.33 312.48 328.90
338.25 363.78 375.88 390.98 410.91 414.55
420.39 443.23 472.49 484.11 493.92 502.10
538.64 556.43 558.68 589.92 603.40 622.02
627.78 637.59 675.73 696.38 715.51 727.47
760.61 762.48 769.01 784.53 811.97 825.60
833.06 849.88 855.05 873.83 875.76 898.76
907.99 938.98 941.98 958.23 964.78 986.57
987.95 994.19 999.91 1007.47 1013.77 1023.43
1039.50 1043.65 1059.57 1096.53 1110.84 1135.25
1137.01 1143.65 1176.76 1185.29 1196.95 1197.64
1217.96 1220.59 1233.78 1243.27 1264.02 1270.99
1274.78 1287.71 1307.84 1318.89 1331.02 1338.85
1364.00 1387.00 1411.22 1436.09 1445.74 1446.49
1480.37 1483.62 1497.92 1508.86 1512.60 1524.77
1530.10 1585.50 1626.84 1639.23 1648.43 1652.02
1685.94 1711.65 1725.59 3056.50 3072.18 3123.33
3134.72 3142.16 3147.44 3153.57 3162.56 3164.49
3174.21 3181.34 3183.37 3188.20 3192.17 3198.85
3204.41 3211.52 3251.35

References

(1) Thanh, N. C. *et al.* Synthesis of N,N,N0,N0-tetrasubstituted 1,3-bis(4-aminophenyl)azulenes and their application to a hole-injecting material in organic electroluminescent devices. *Tetrahedron* **62**, 11227-11239 (2006)

(2) Park, S., Yong, W.-S., Kim, S., Lee, P. H. Diastereoselective *N*-sulfonylamino-alkenylation of azulenes from terminal alkynes and azides via *N*-sulfonyl-1,2,3-triazoles. *Org. Lett.* **16**, 4468–4471 (2014).

(3) Seo, B., Jeon, W. H., Kim, J., Kim, S., Lee, P. H. Synthesis of fluorenes via tandem copper-catalyzed [3 + 2] cycloaddition and rhodium-catalyzed denitrogenative cyclization in a 5-*exo* mode from 2-ethynylbiaryls and *N*-sulfonyl azides in one pot. *J. Org. Chem.* **80**, 722–732 (2015).

(4) Briquet, A. A., Hansen, H.-J. New results in the synthesis of styrylazulene derivatives: application of the 'anil synthesis' to the preparation of azulenes substituted with styryl groups at the seven-membered ring. *Hel. Chim. Acta* **77**, 1921–1939 (1994).

(5) Zhu, C., Xu, G., Ding, D., Qiu, L., Sun, J. Copper-Catalyzed Diazo Cross-/Homo-Coupling toward Tetrasubstituted Olefins and Applications on the Synthesis of Maleimide Derivatives. *Org. Lett.* **17**, 4244–4247 (2015)

^{1}H and ^{13}C NMR spectra

^{1}H NMR (400 MHZ, C_6D_6)

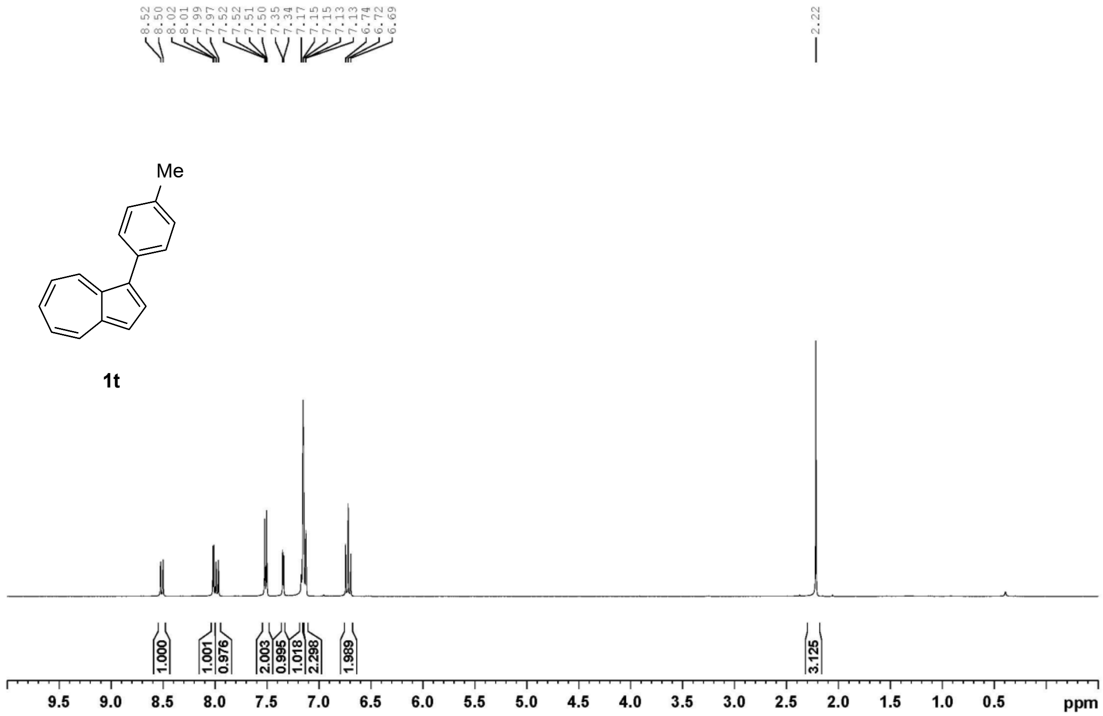

^{13}C{^{1}H}NMR (100 MHz, C_6D_6)

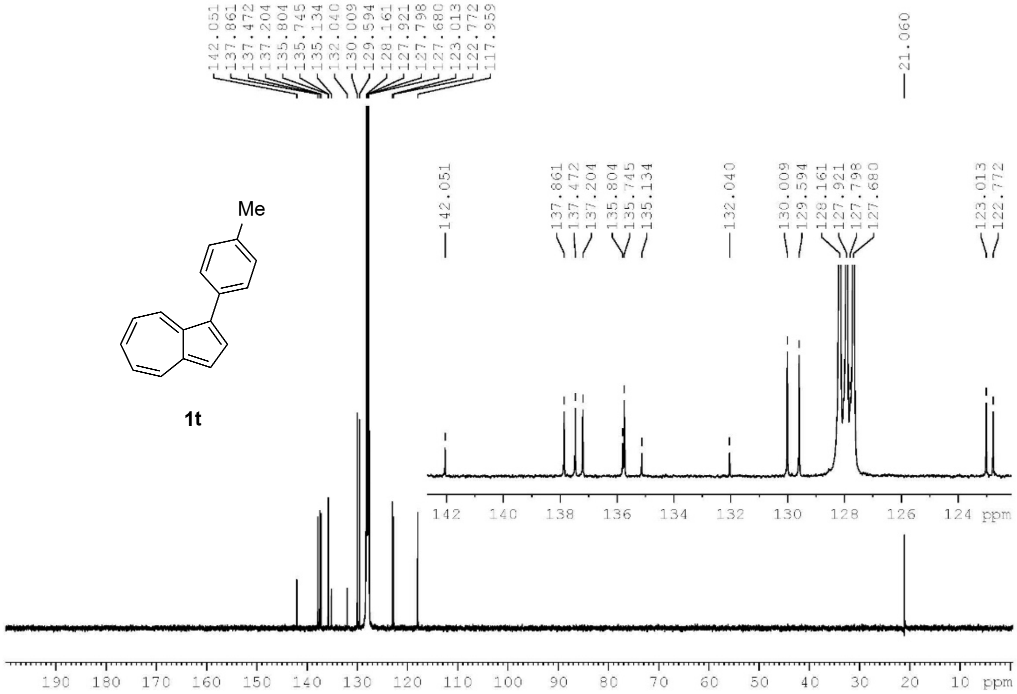

^{1}H NMR (400 MHZ, C_6D_6)

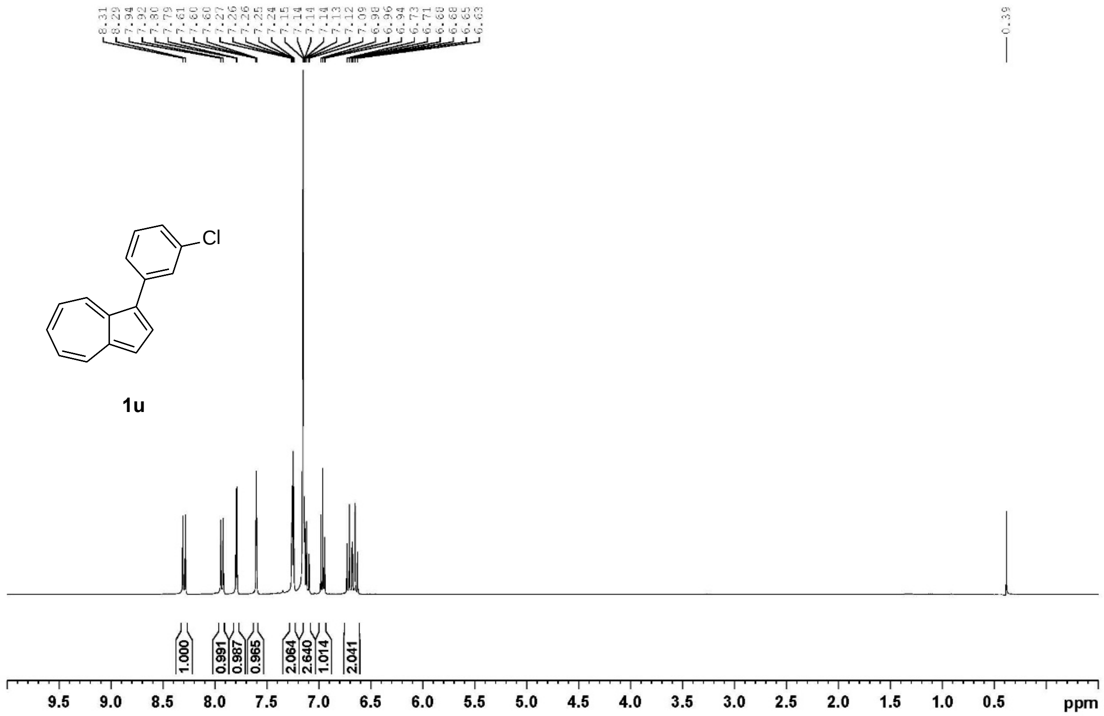

^{13}C{^{1}H}NMR (100 MHz, C_6D_6)

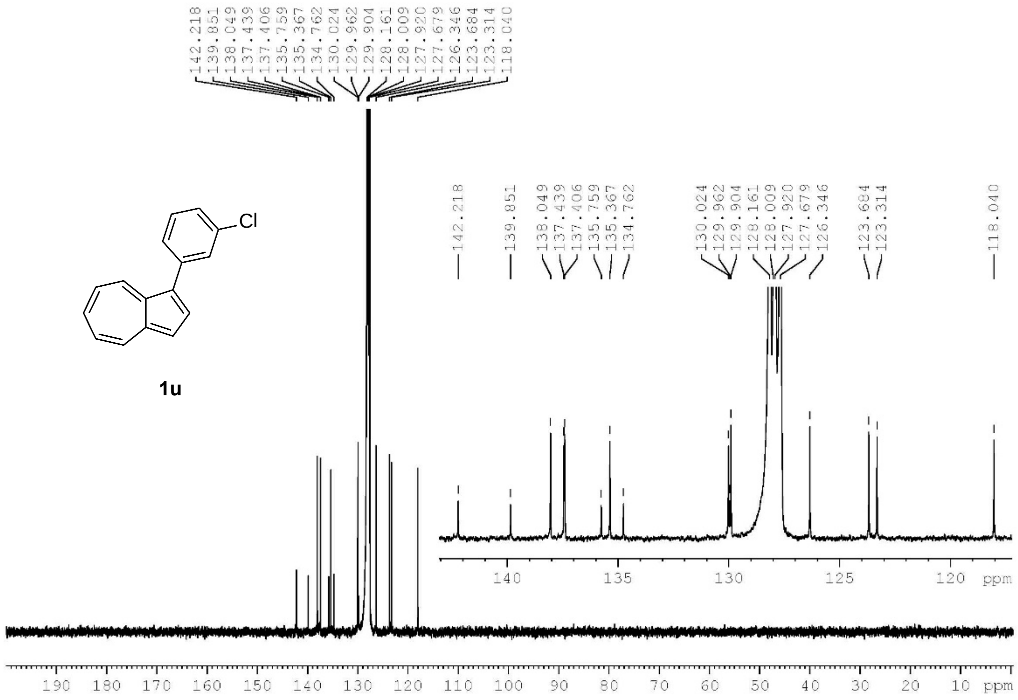

^{1}H NMR (400 MHz, $CDCl_3$)

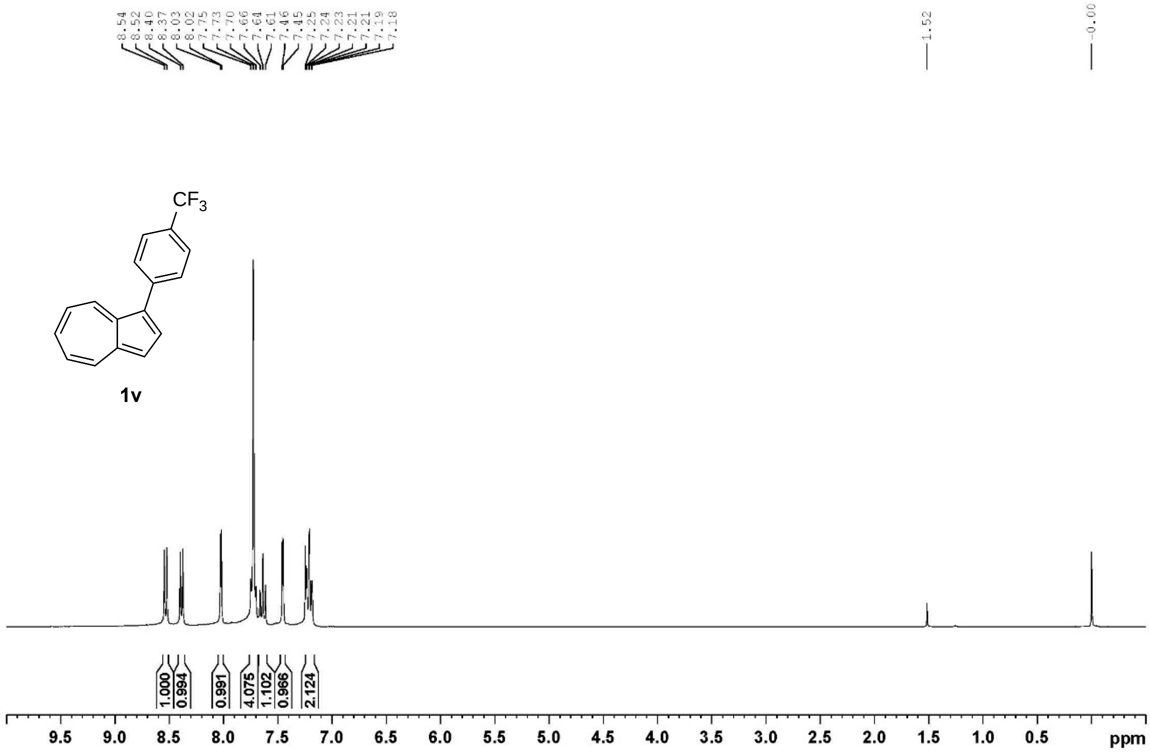

$^{13}C\{^{1}H\}$ NMR (100 MHz, $CDCl_3$)

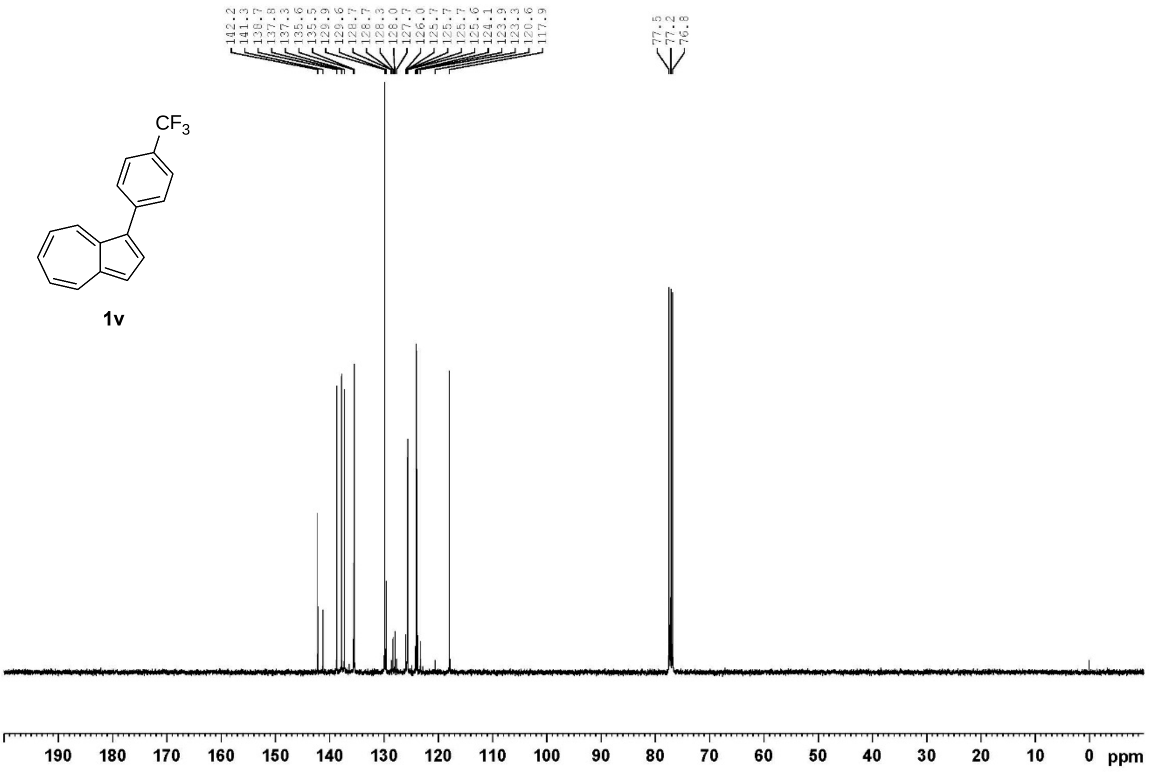

^{1}H NMR (400 MHz, $CDCl_3$)

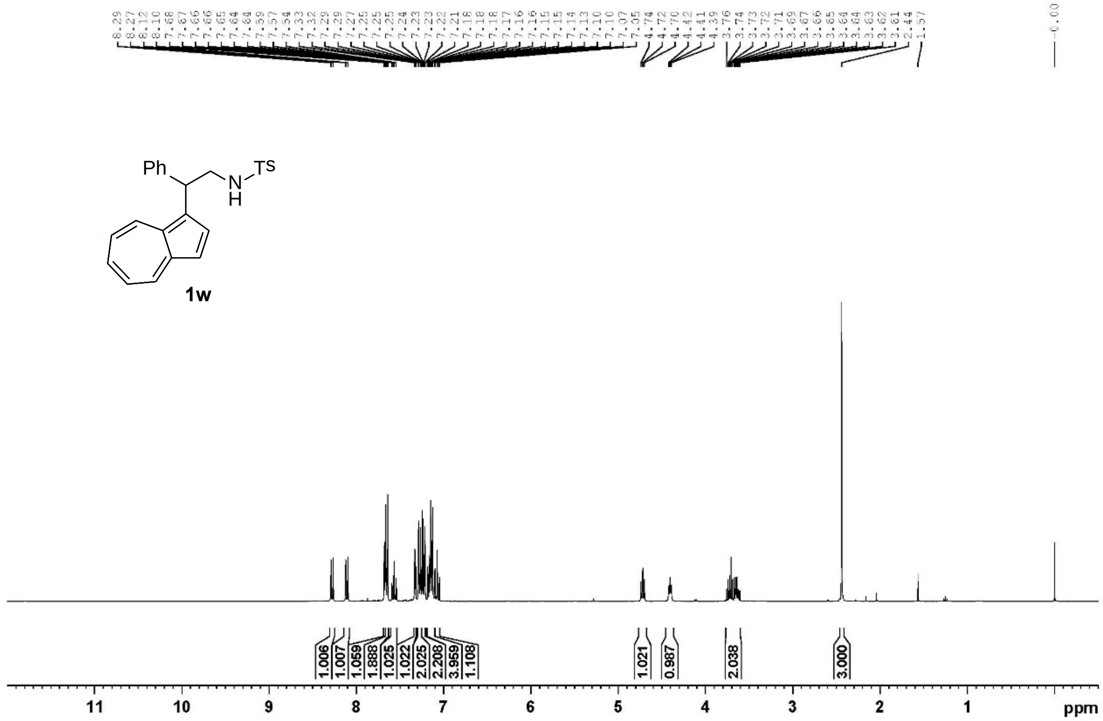

$^{13}C\{^{1}H\}$ NMR (100 MHz, $CDCl_3$)

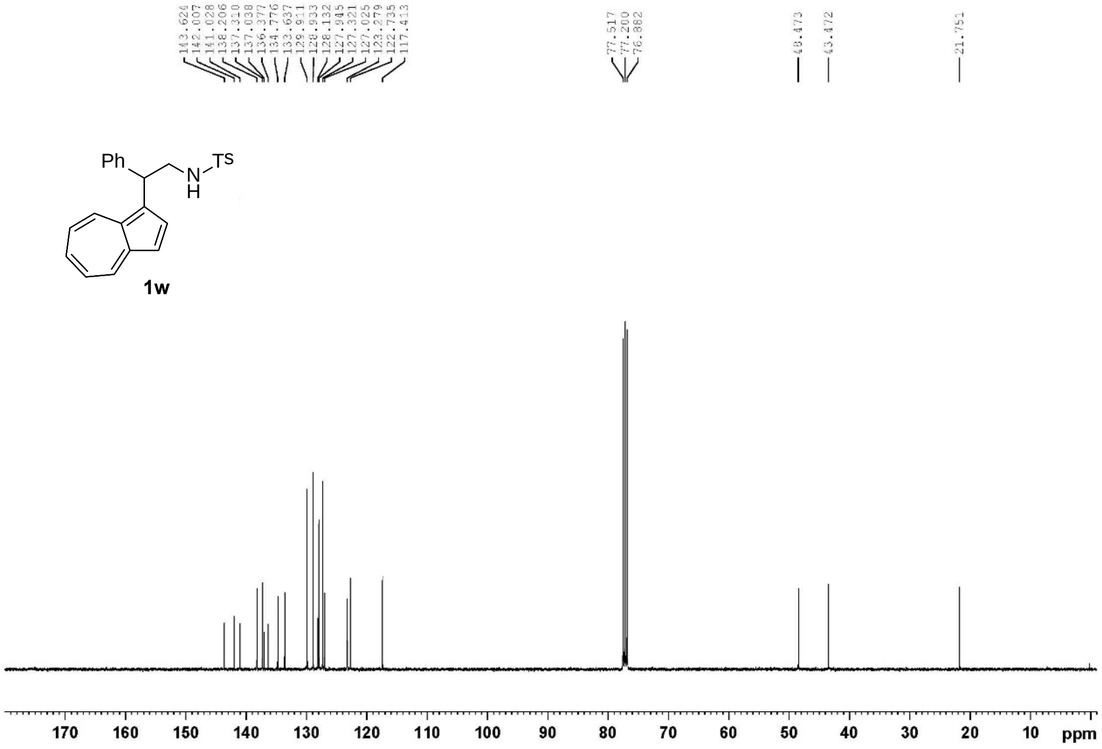

^{1}H NMR (400 MHz, $CDCl_3$)

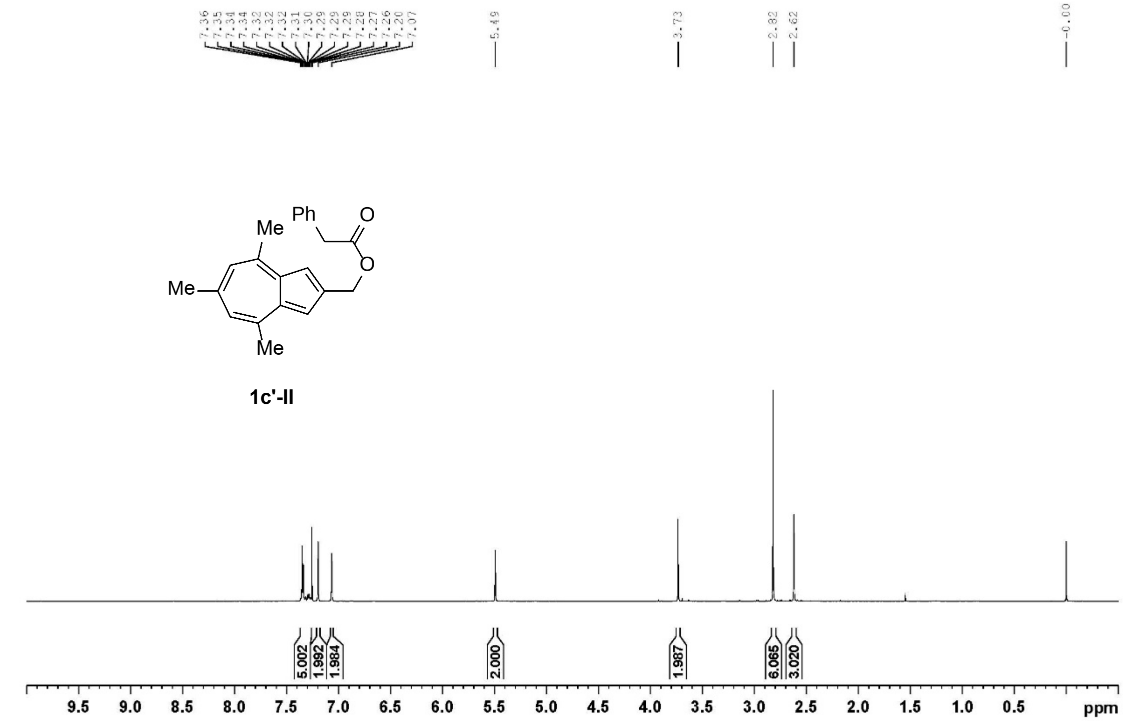

^{13}C{^{1}H} NMR (100 MHz, $CDCl_3$)

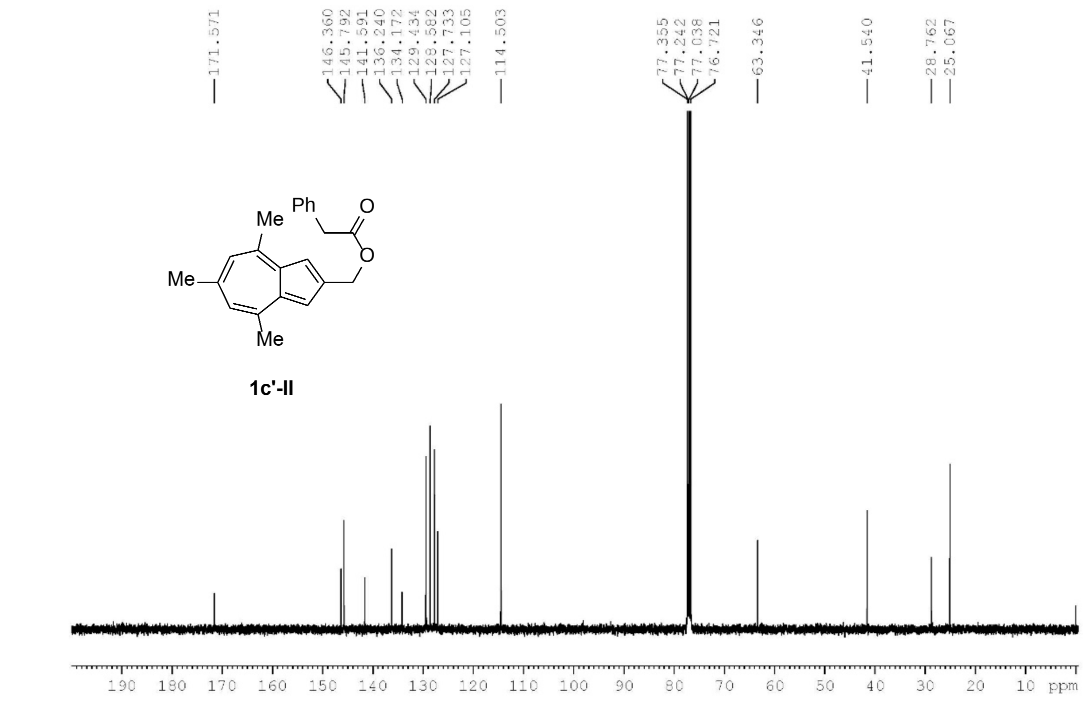

^{1}H NMR (400 MHz, $CDCl_3$)

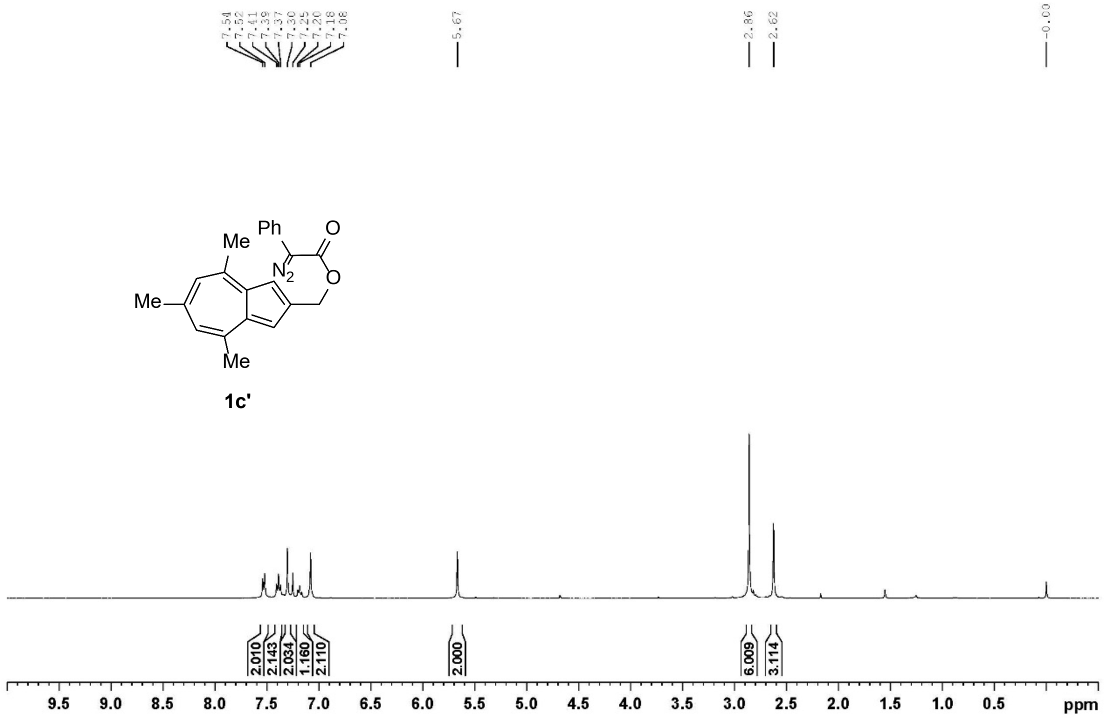

1c'

^{13}C{^{1}H} NMR (100 MHz, $CDCl_3$)

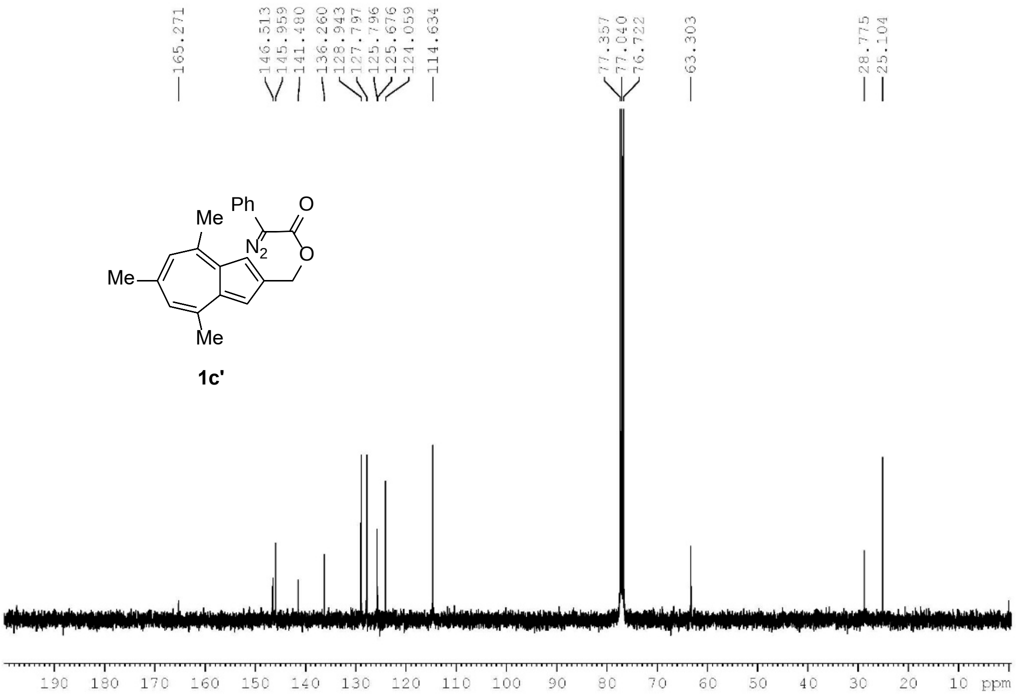

1c'

^{1}H NMR (400 MHZ, C_6D_6)

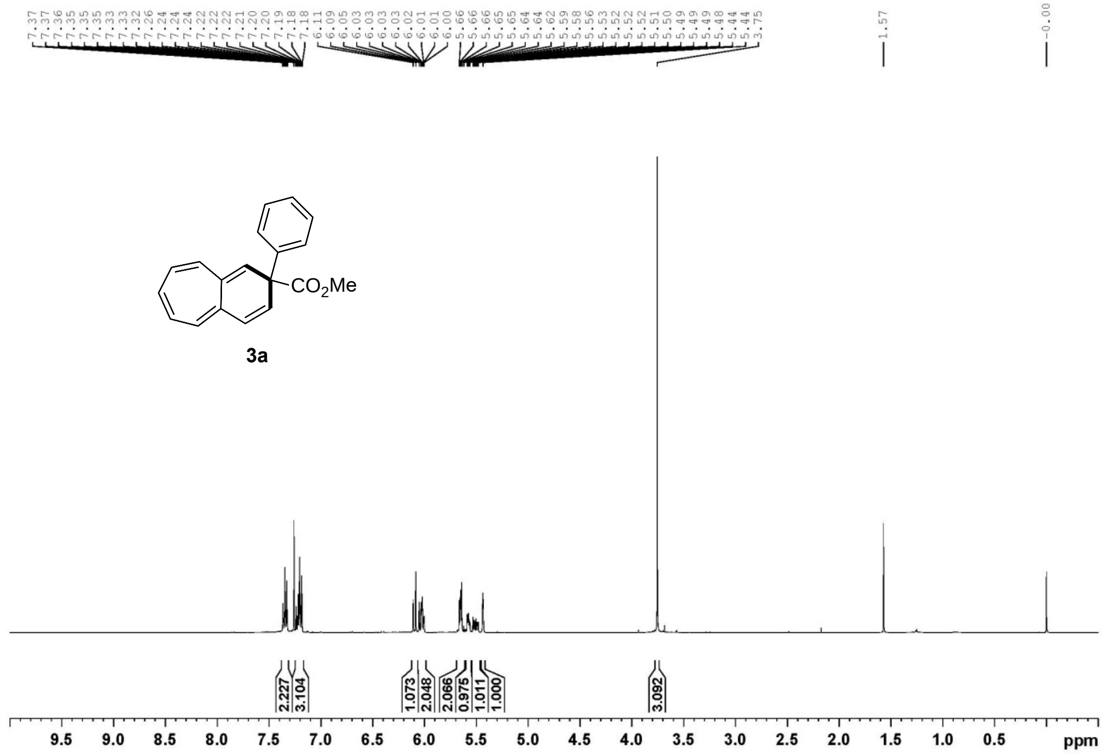

$^{13}C\{^{1}H\}$ NMR (100 MHz, $CDCl_3$)

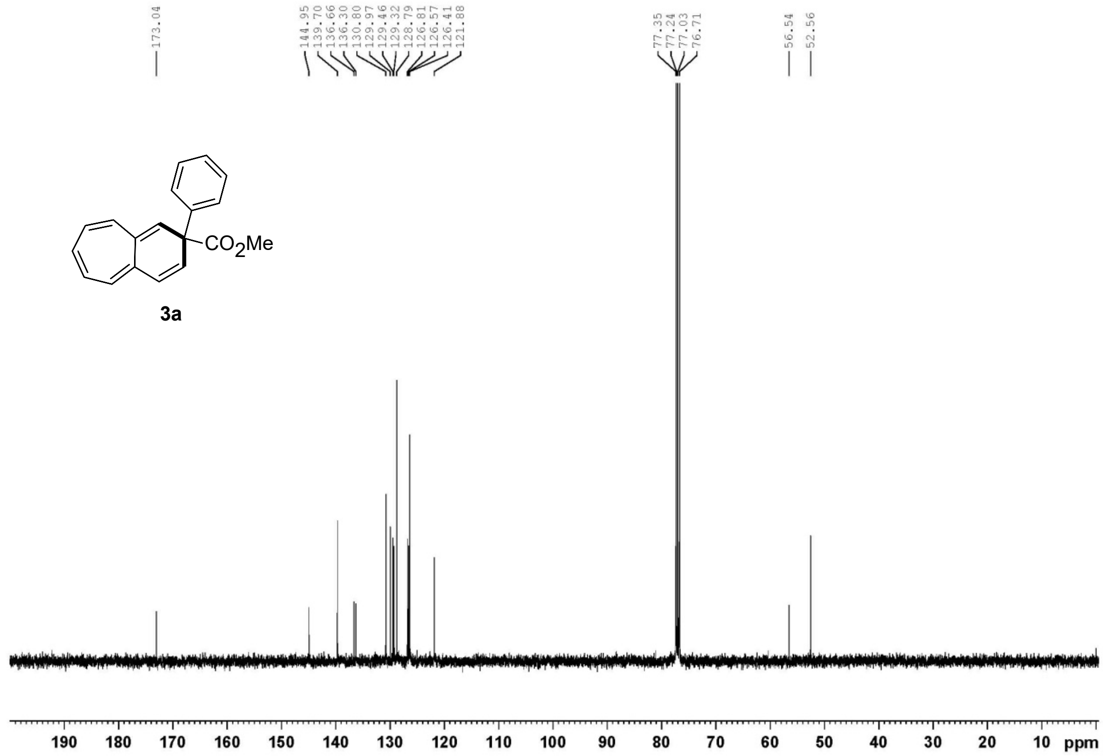

^{1}H NMR (400 MHZ, C_6D_6)

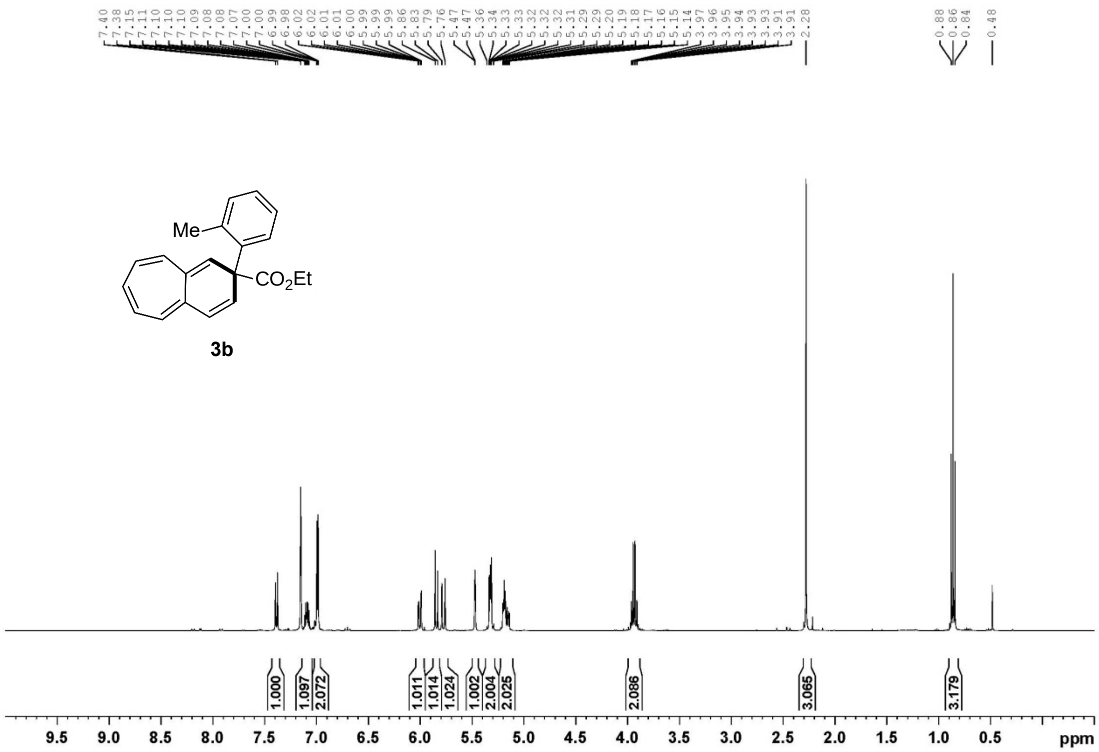

$^{13}C\{^{1}H\}$NMR (100 MHz, C_6D_6)

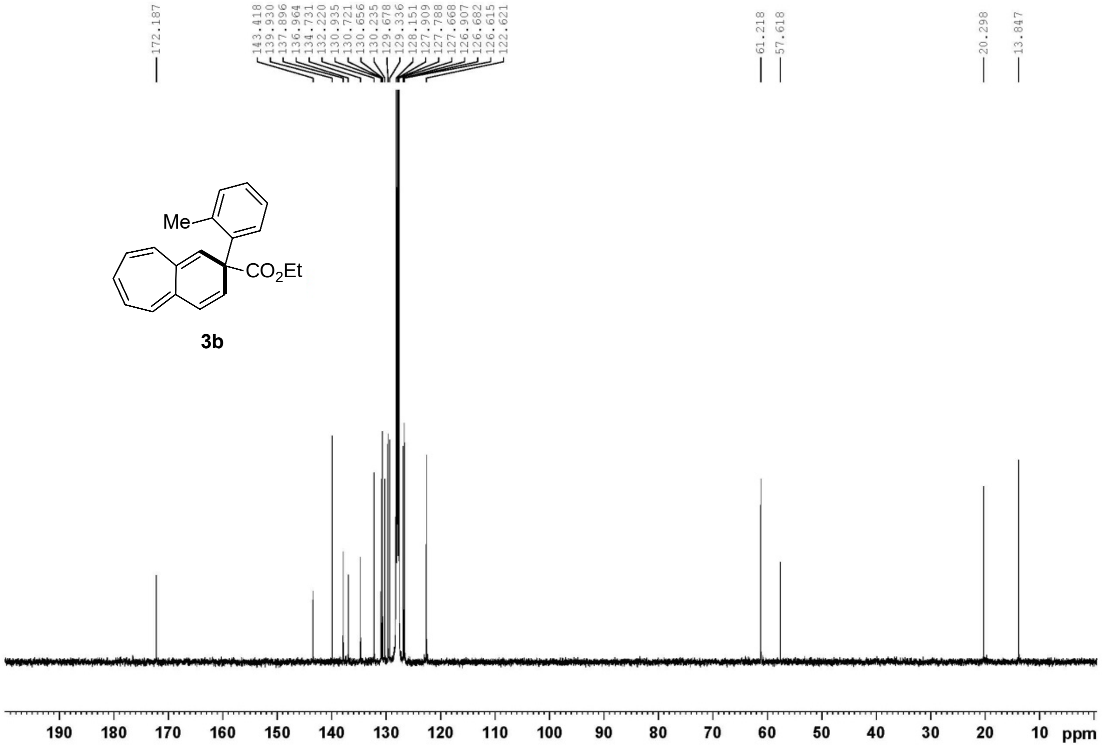

^{1}H NMR (400 MHZ, C_6D_6)

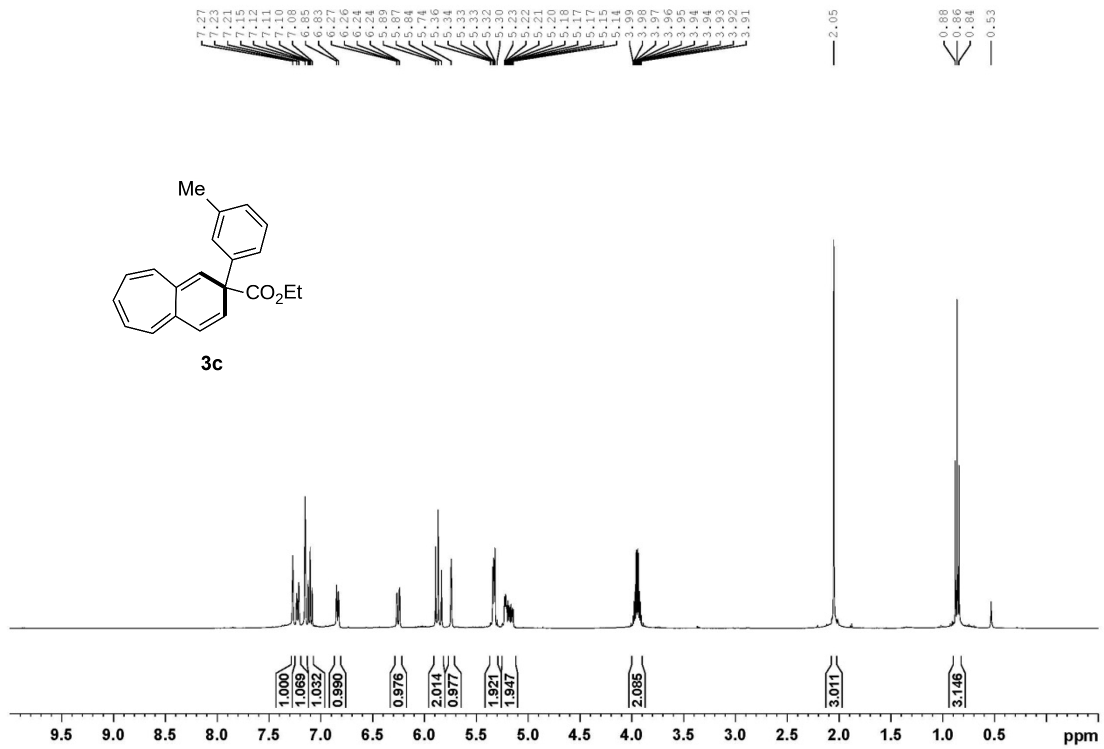

^{13}C{^{1}H}NMR (100 MHz, C_6D_6)

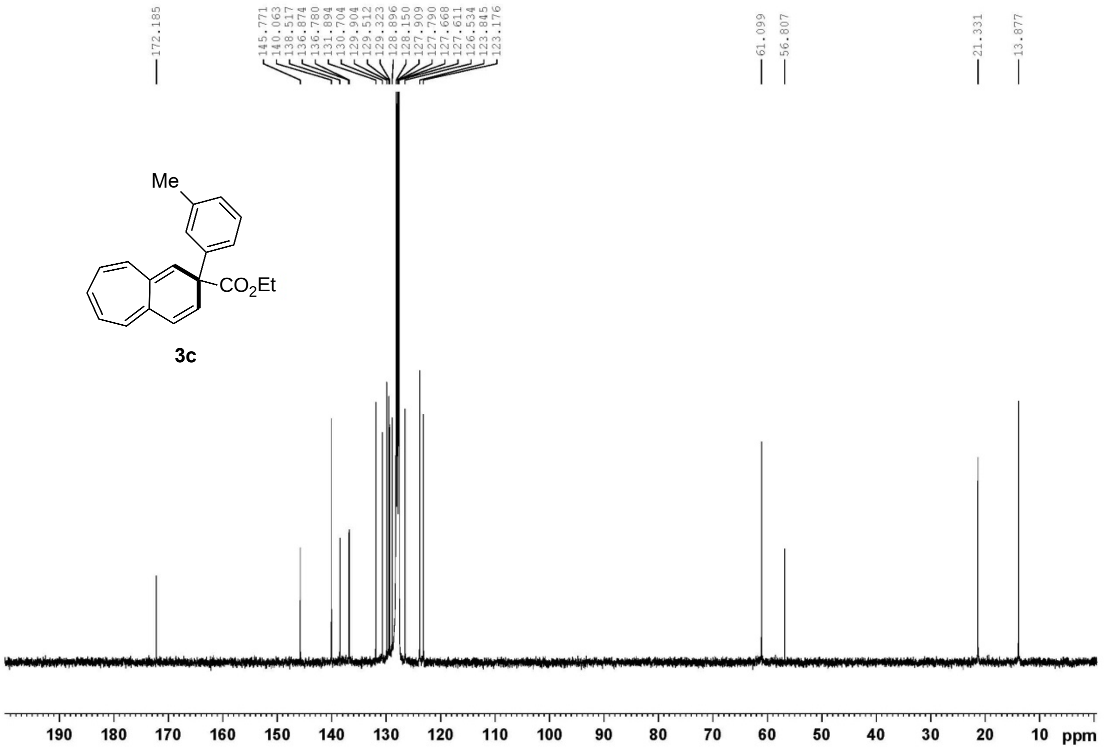

^{1}H NMR (400 MHZ, C_6D_6)

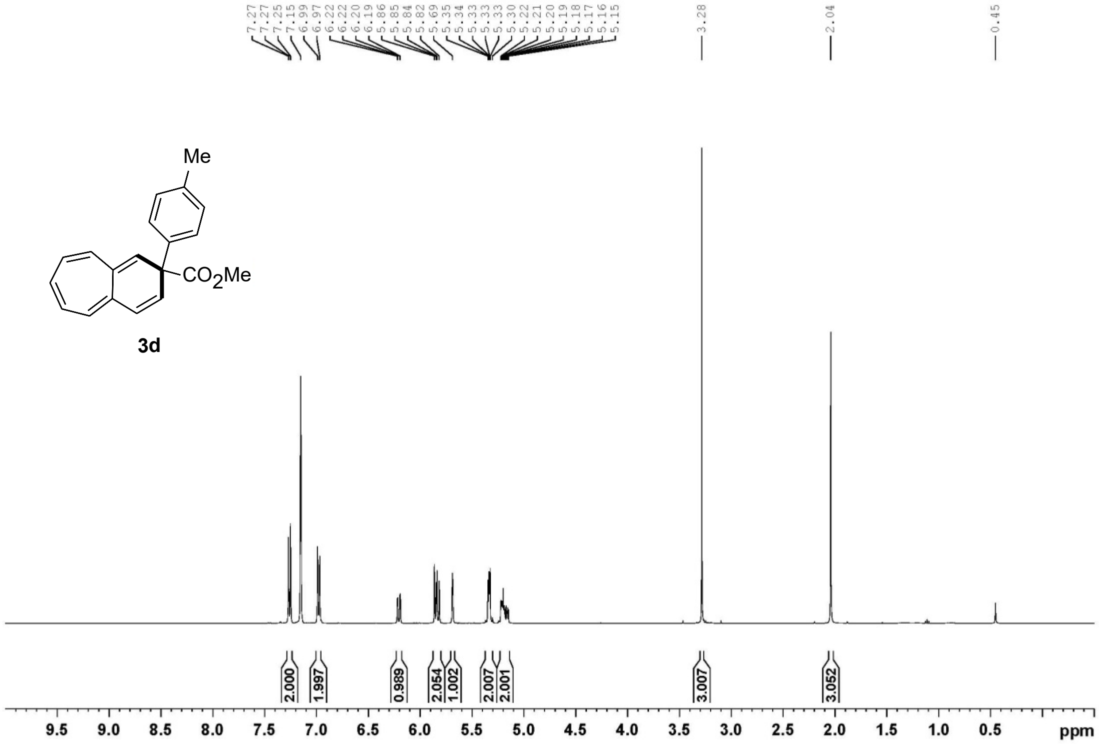

^{13}C{^{1}H}NMR (100 MHz, C_6D_6)

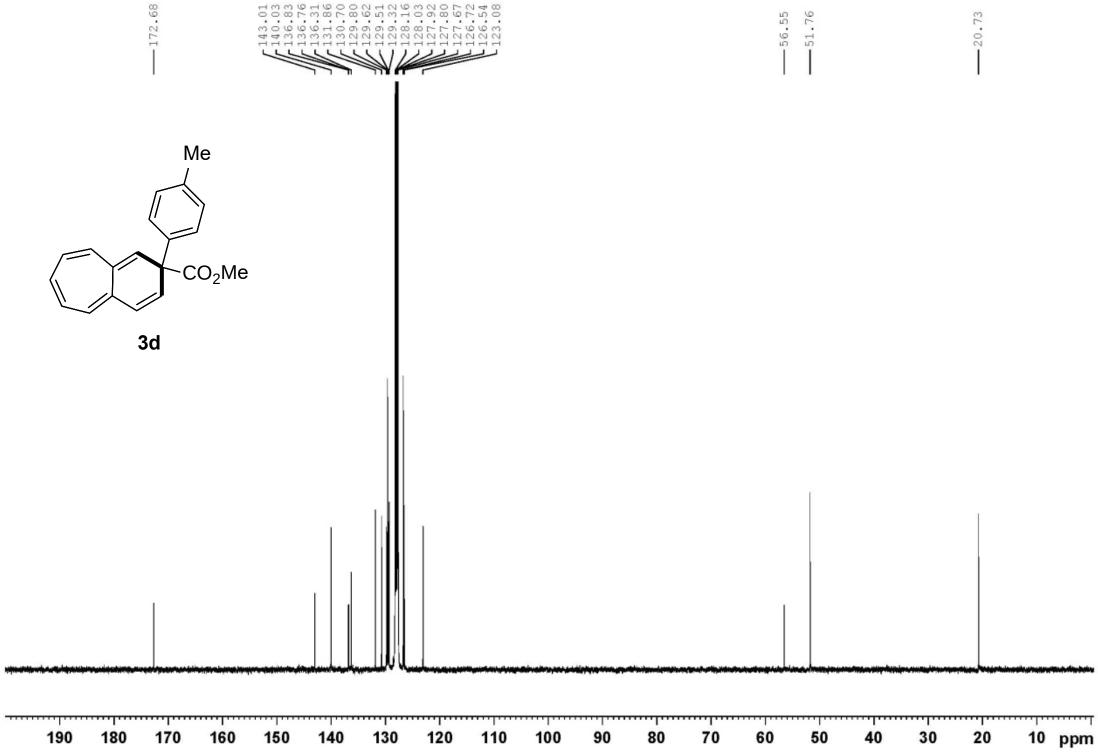

^{1}H NMR (400 MHZ, C_6D_6)

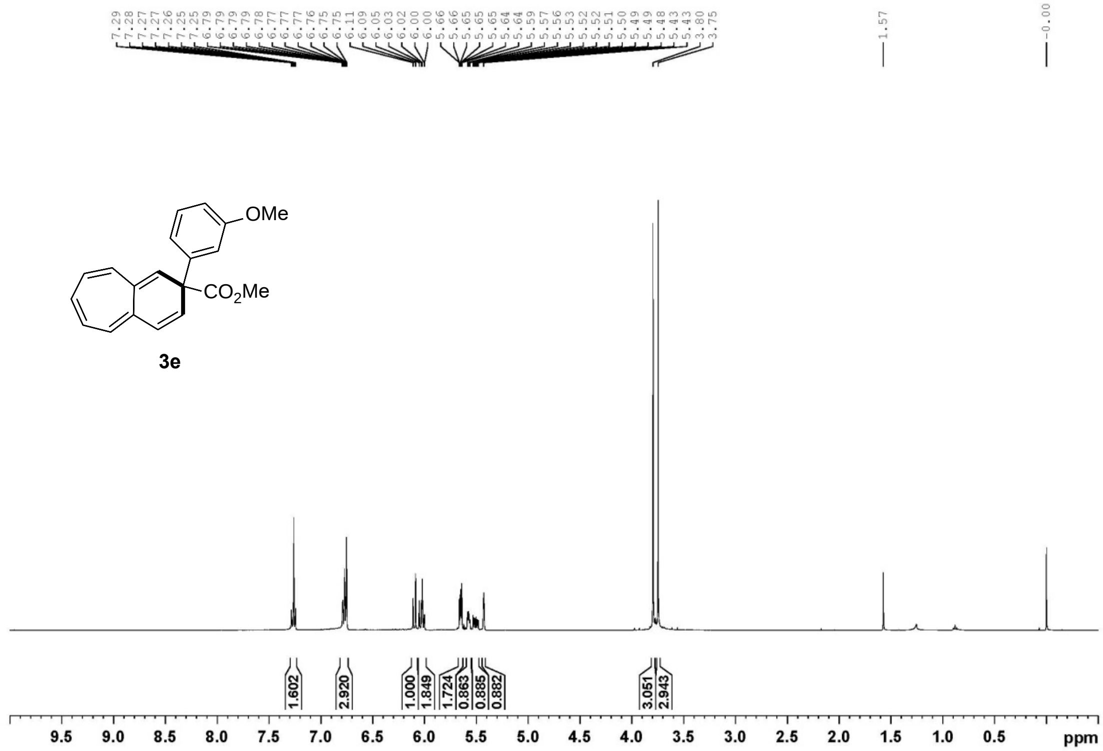

^{13}C{^{1}H} NMR (100 MHz, $CDCl_3$)

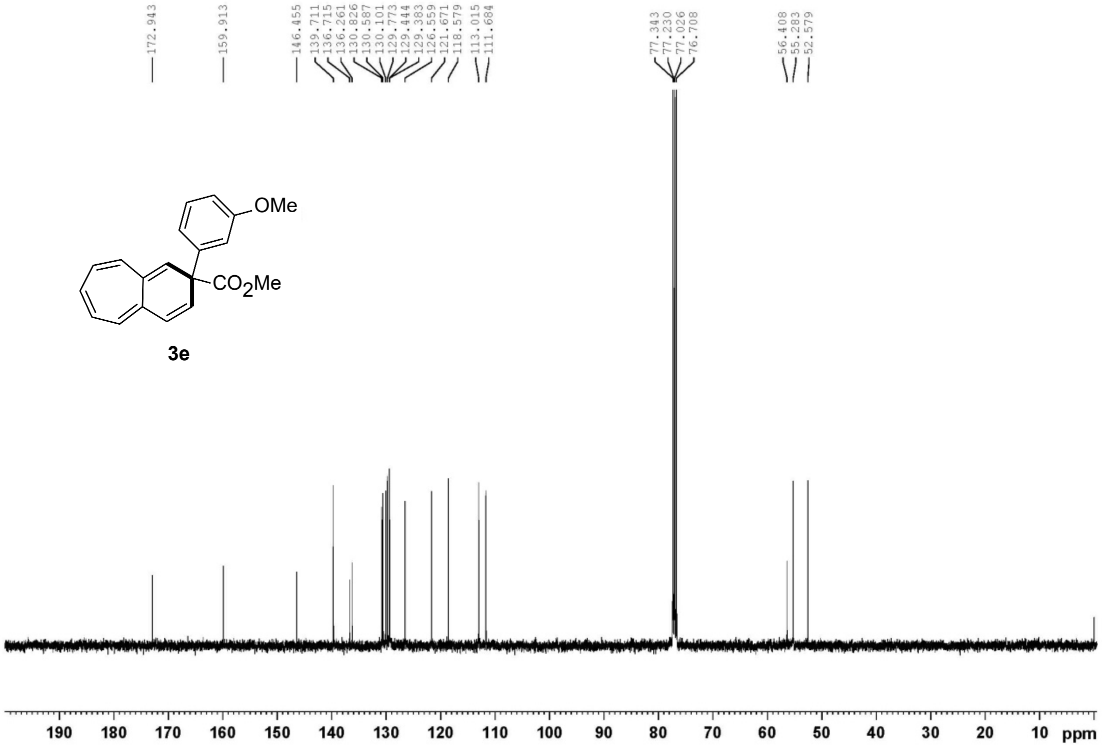

^{1}H NMR (400 MHZ, C_6D_6)

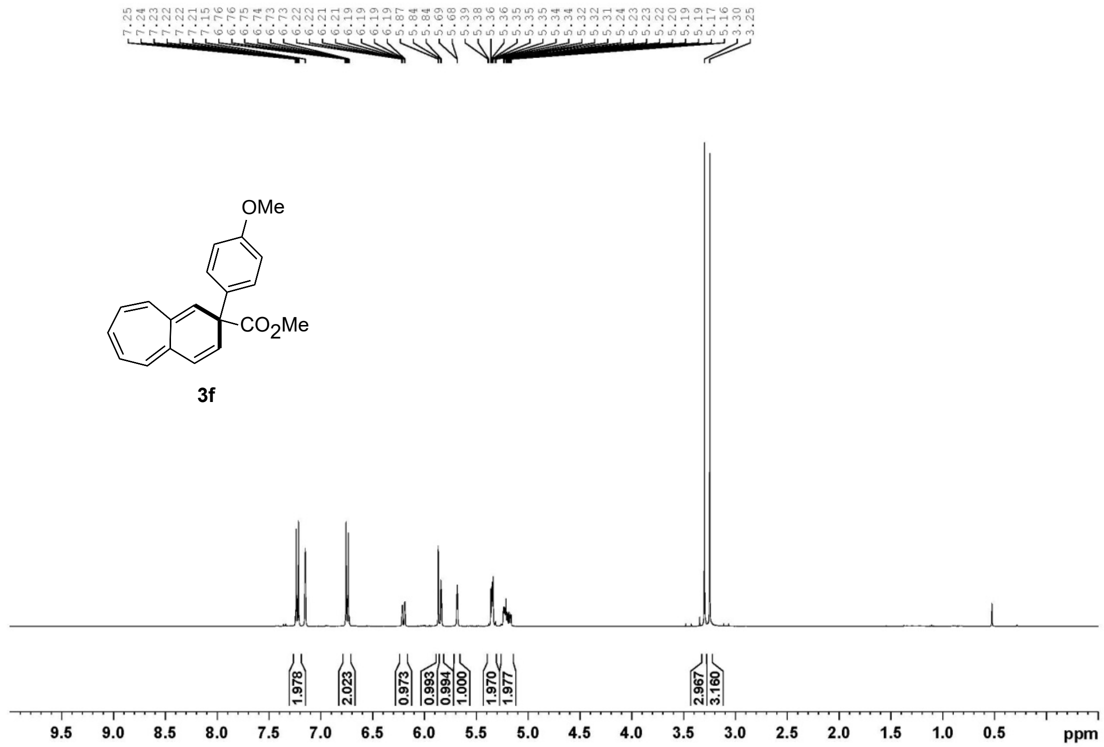

^{13}C{^{1}H} NMR (100 MHz, $CDCl_3$)

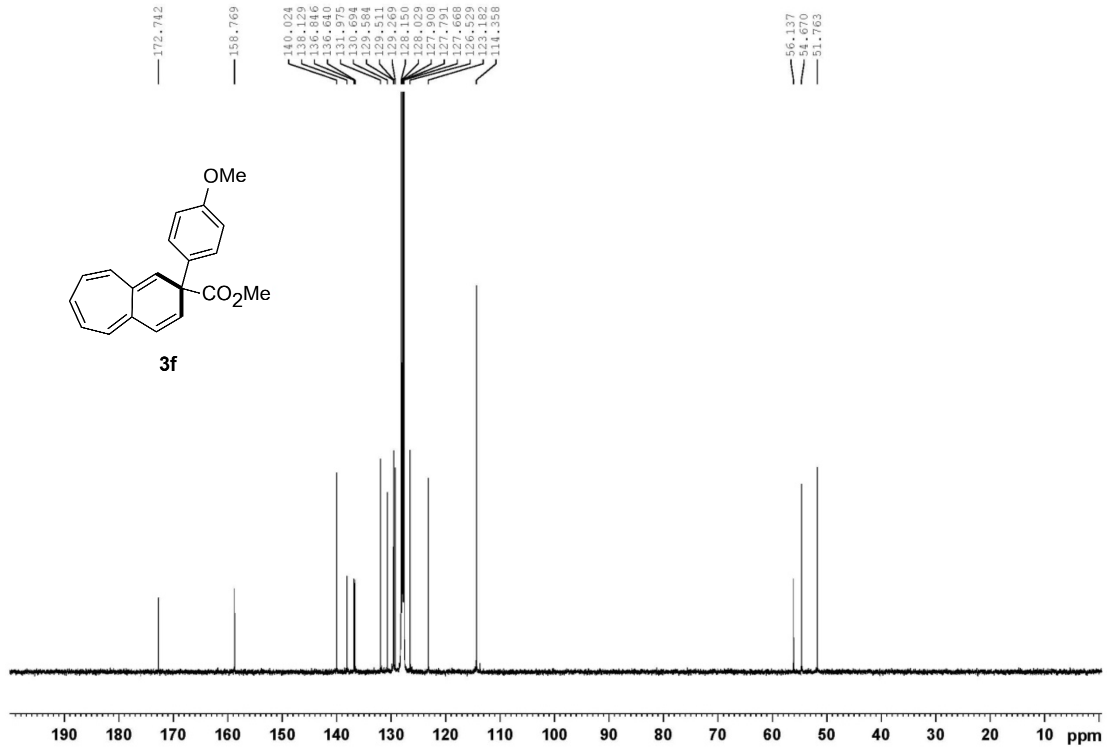

^{1}H NMR (400 MHZ, C_6D_6)

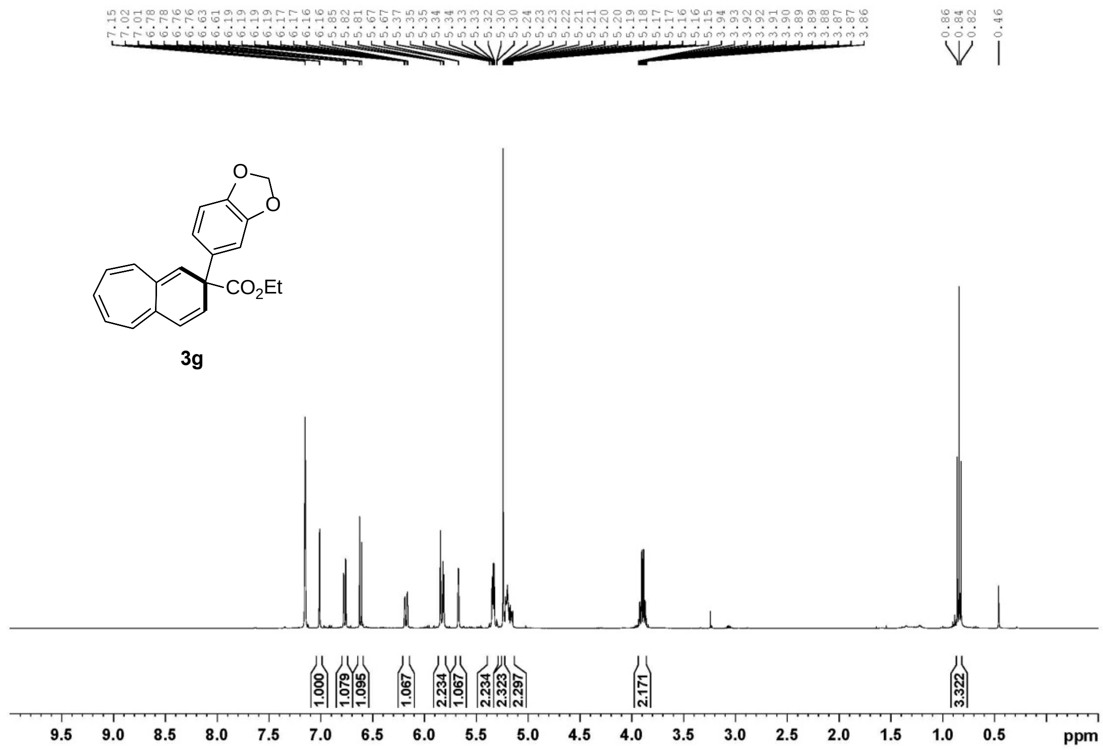

$^{13}C\{^{1}H\}$ NMR (100 MHz, $CDCl_3$)

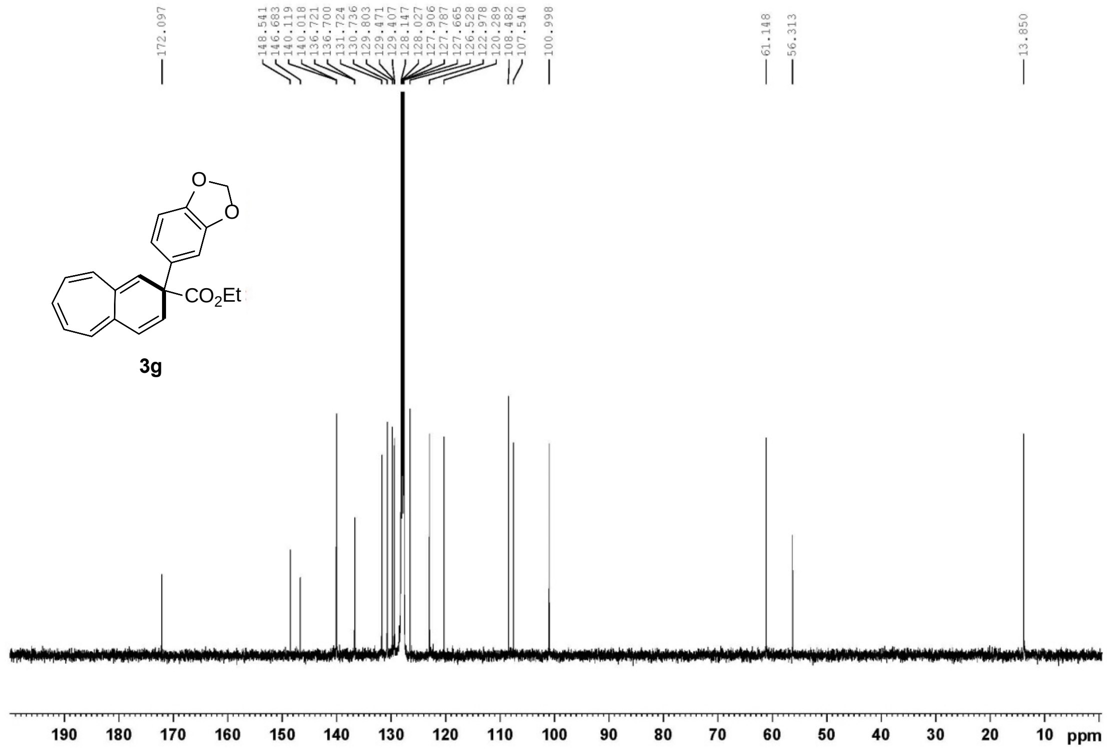

^{1}H NMR (400 MHZ, C_6D_6)

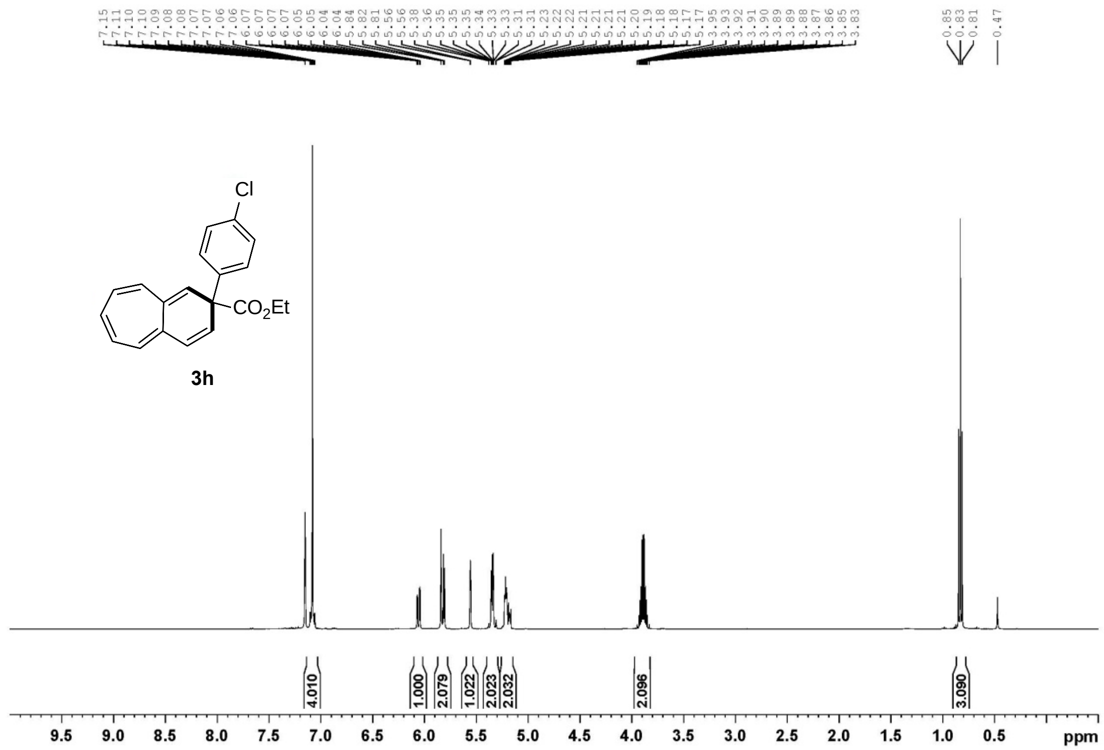

$^{13}C\{^{1}H\}$ NMR (100 MHz, $CDCl_3$)

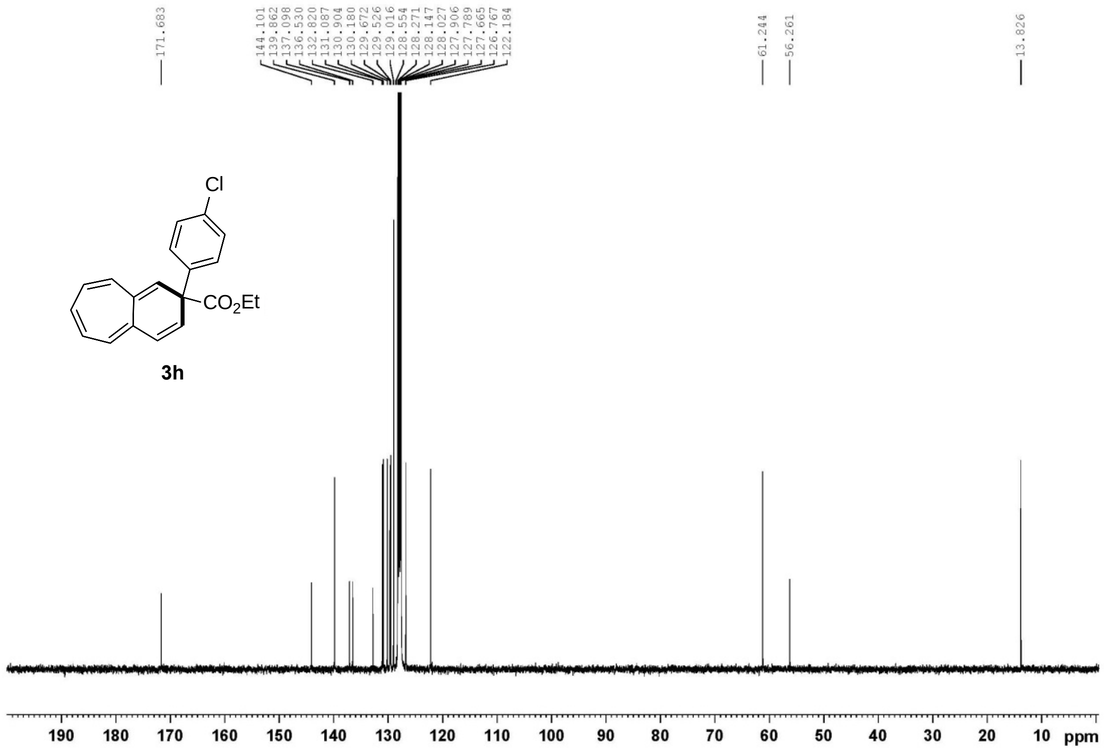

^{1}H NMR (400 MHZ, C_6D_6)

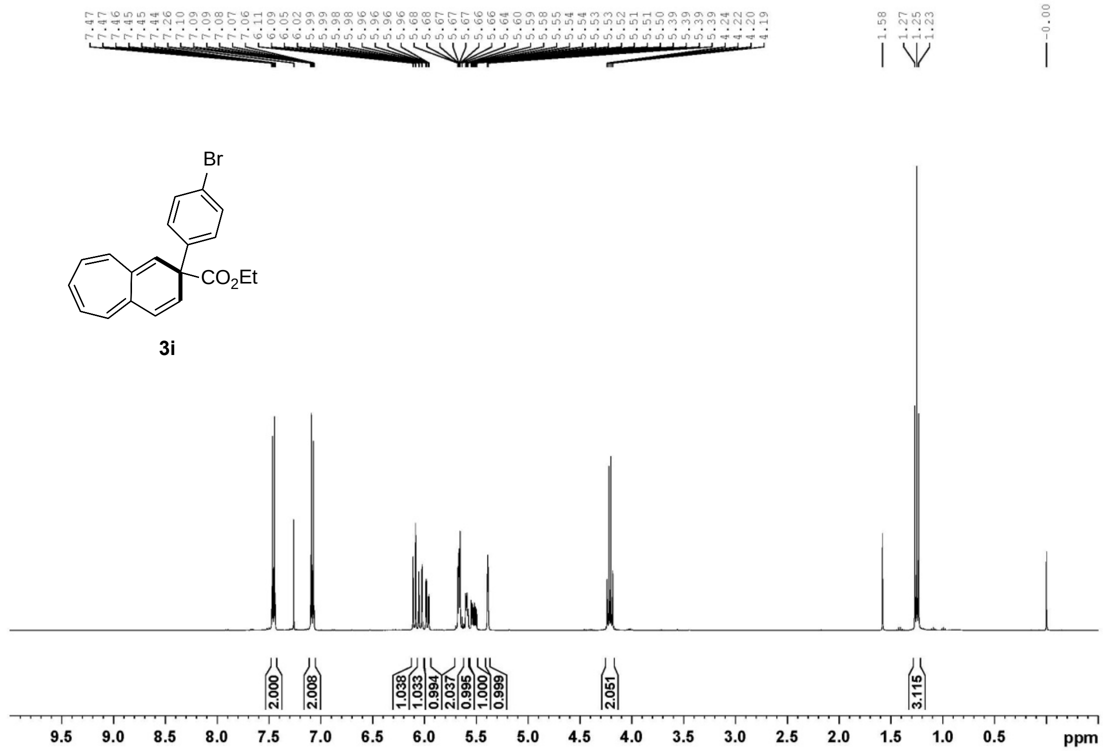

^{13}C{^{1}H} NMR (100 MHz, $CDCl_3$)

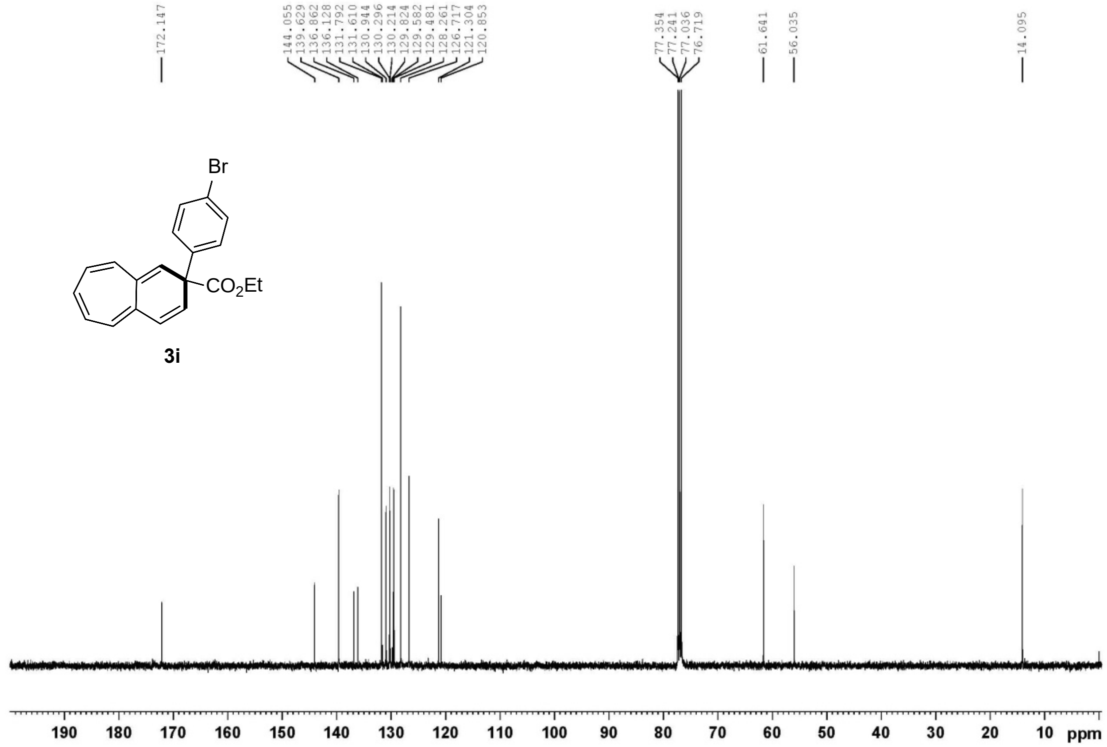

^{1}H NMR (400 MHZ, C_6D_6)

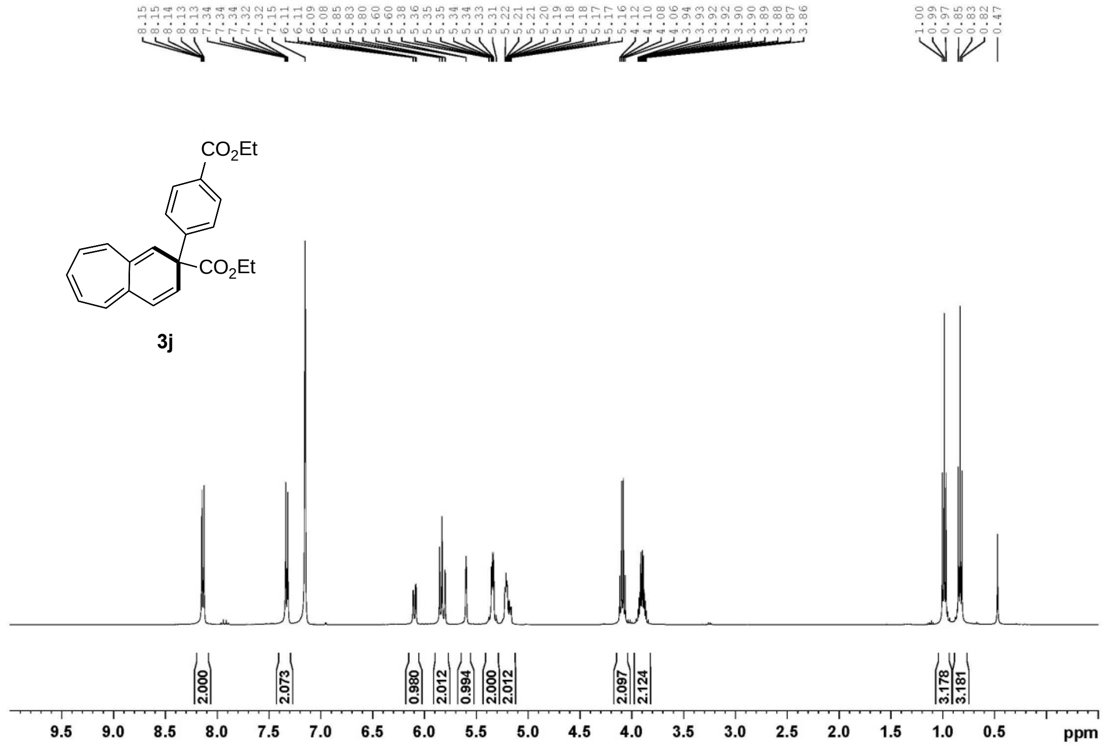

$^{13}C\{^{1}H\}$ NMR (100 MHz, $CDCl_3$)

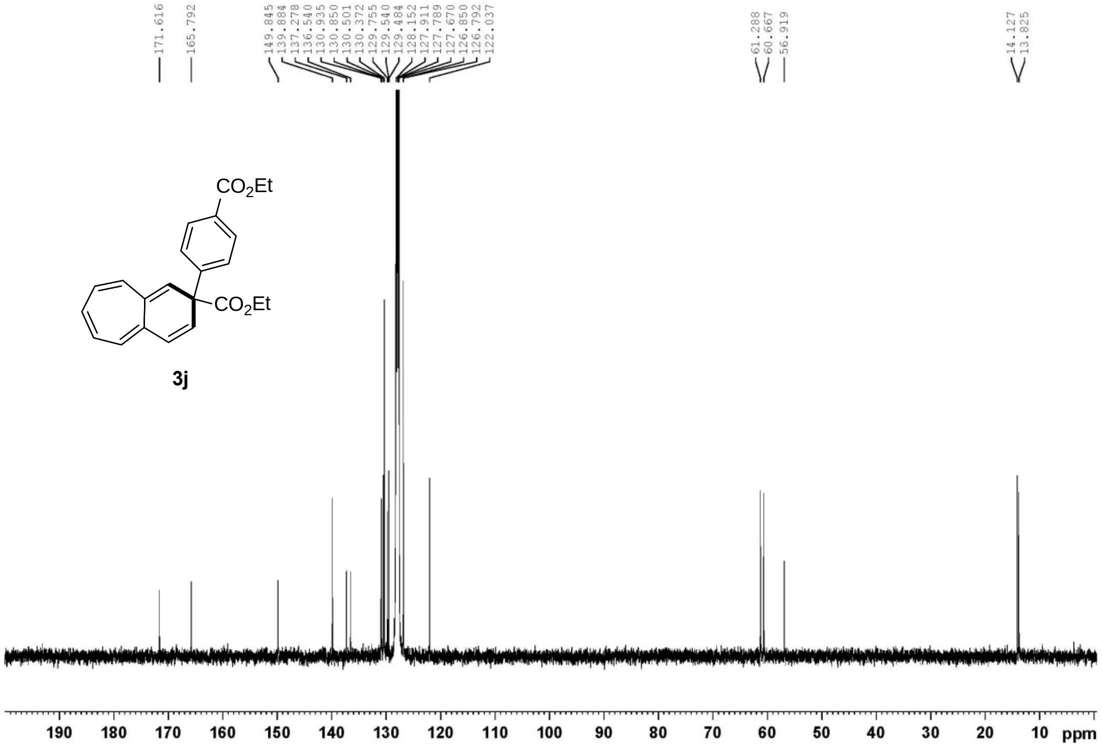

^{1}H NMR (400 MHZ, C_6D_6)

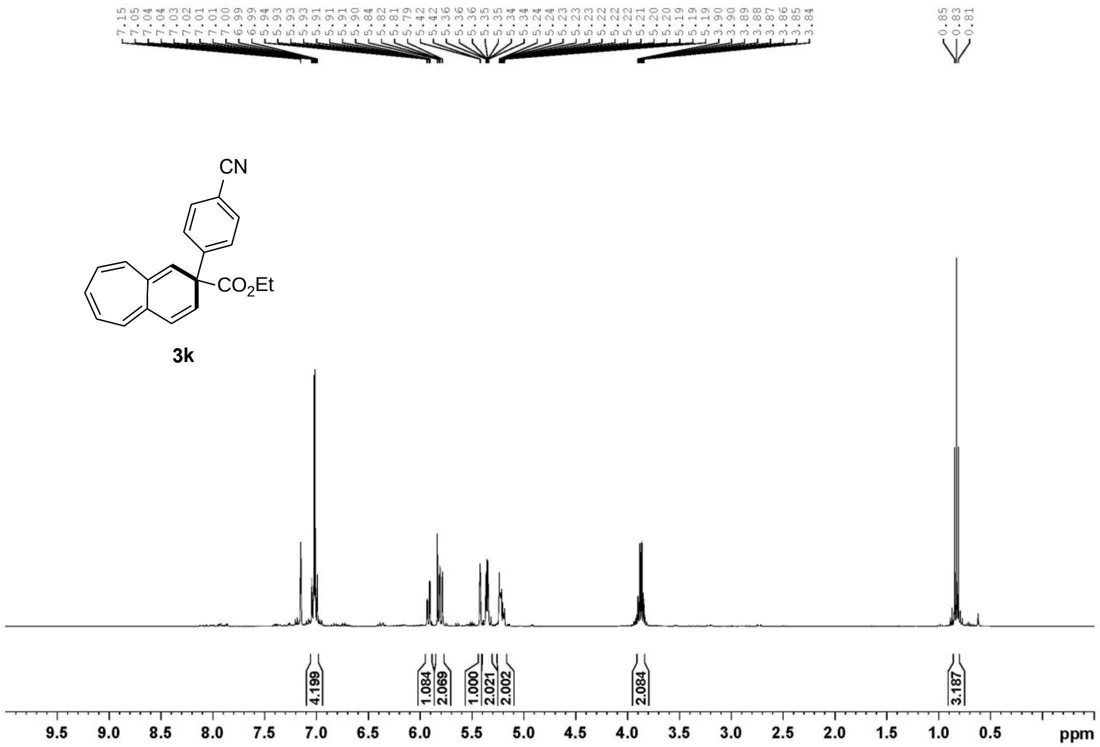

^{13}C{^{1}H} NMR (100 MHz, $CDCl_3$)

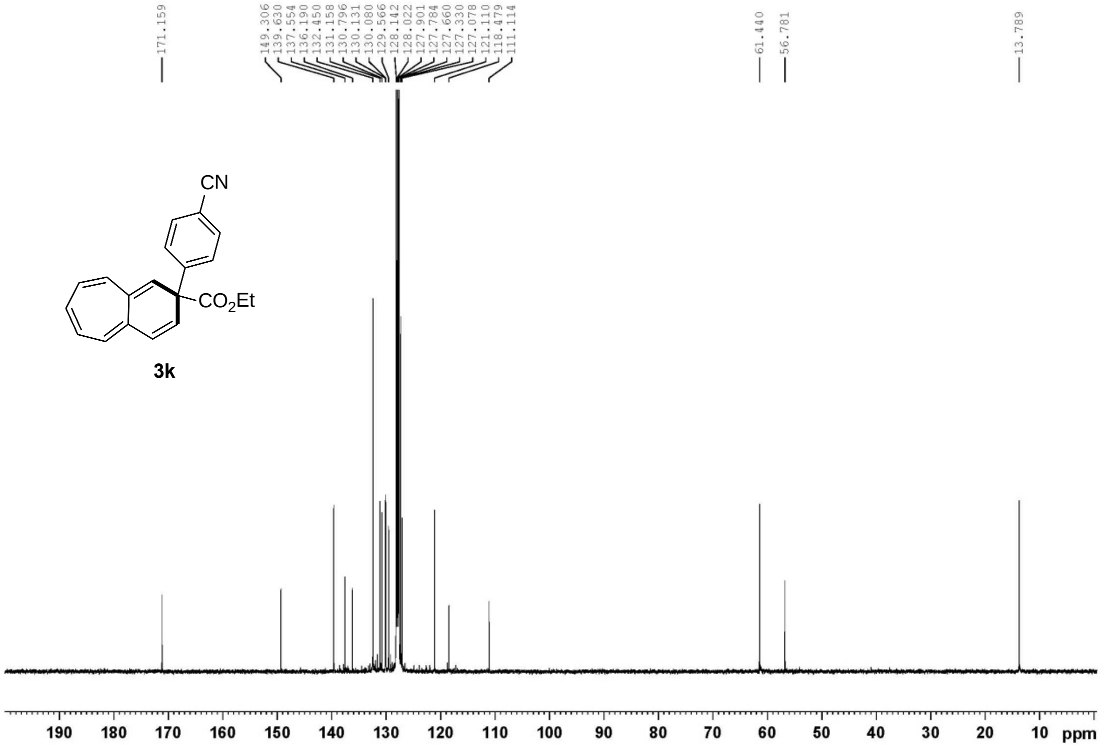

^{1}H NMR (400 MHZ, C_6D_6)

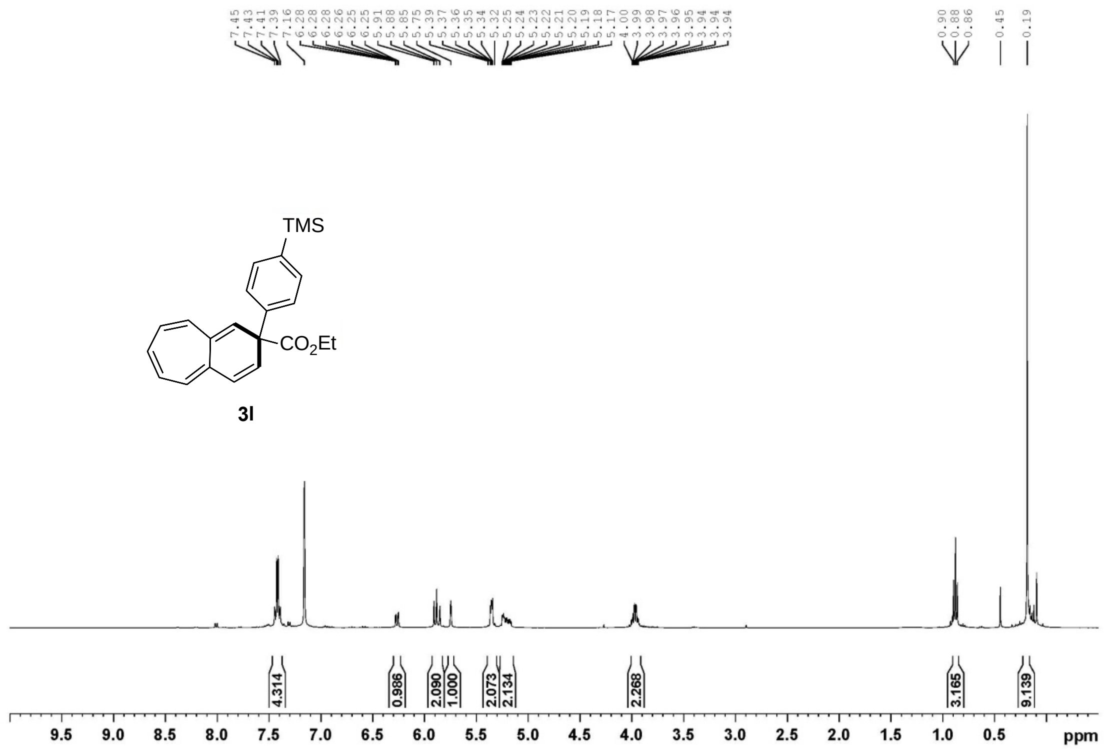

$^{13}C\{^{1}H\}$ NMR (100 MHz, $CDCl_3$)

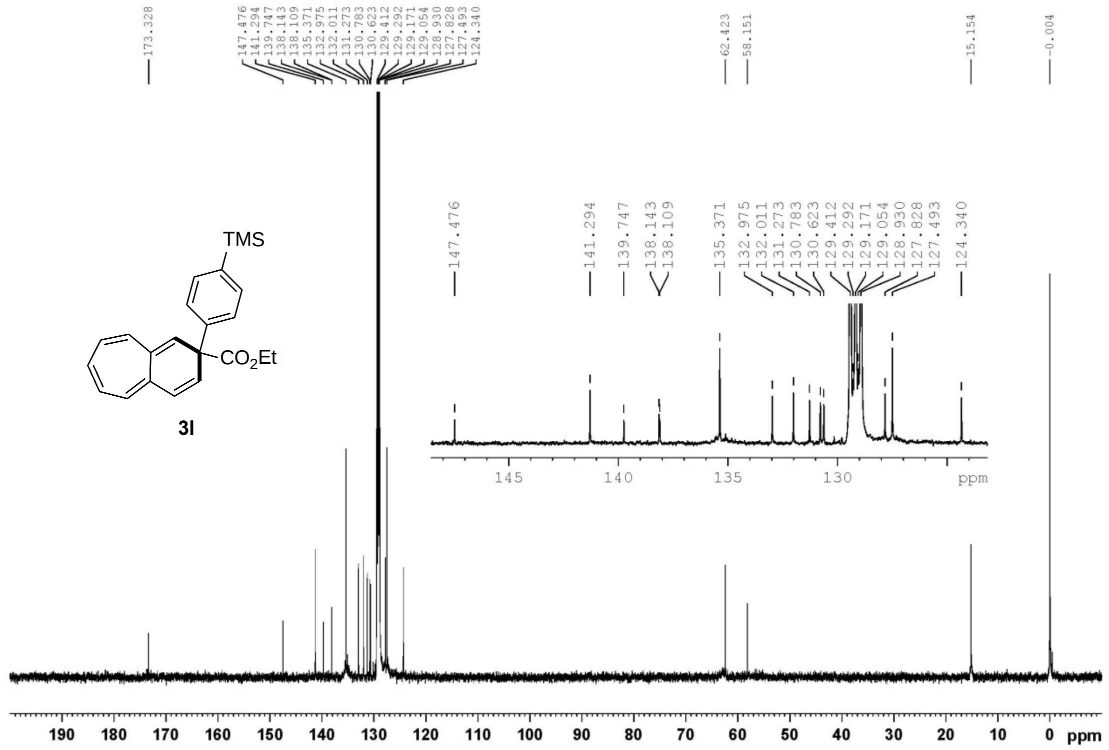

^{1}H NMR (400 MHZ, C_6D_6)

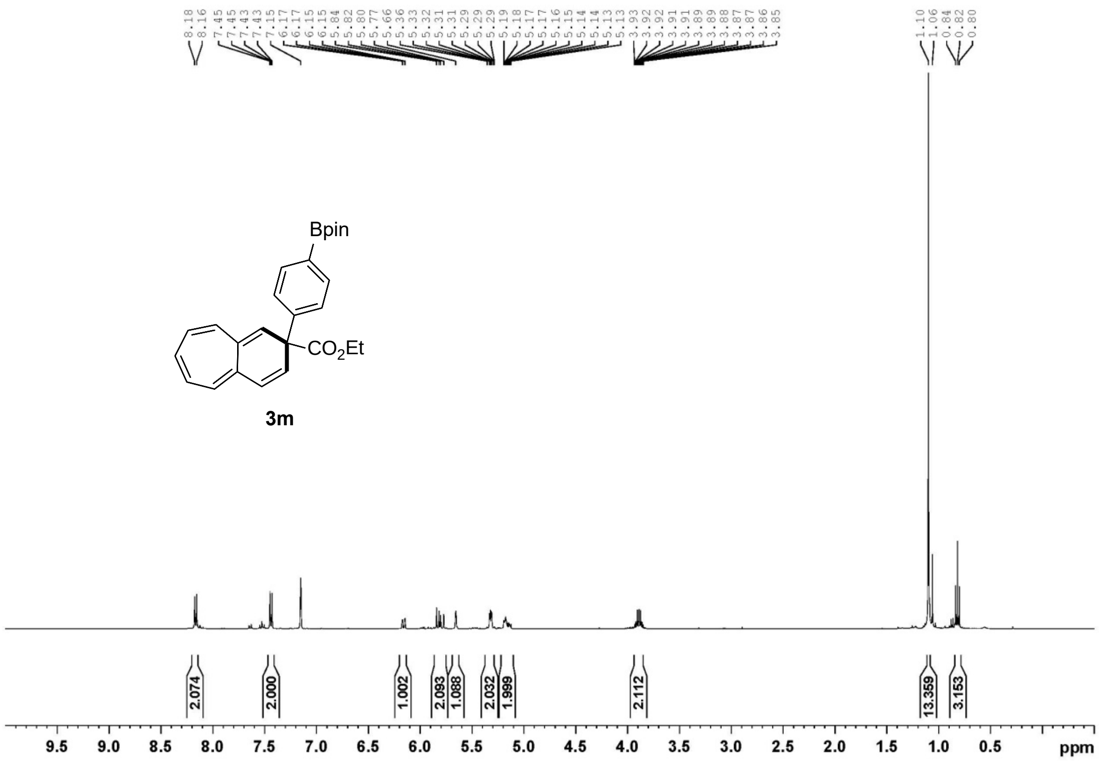

^{13}C{^{1}H} NMR (100 MHz, $CDCl_3$)

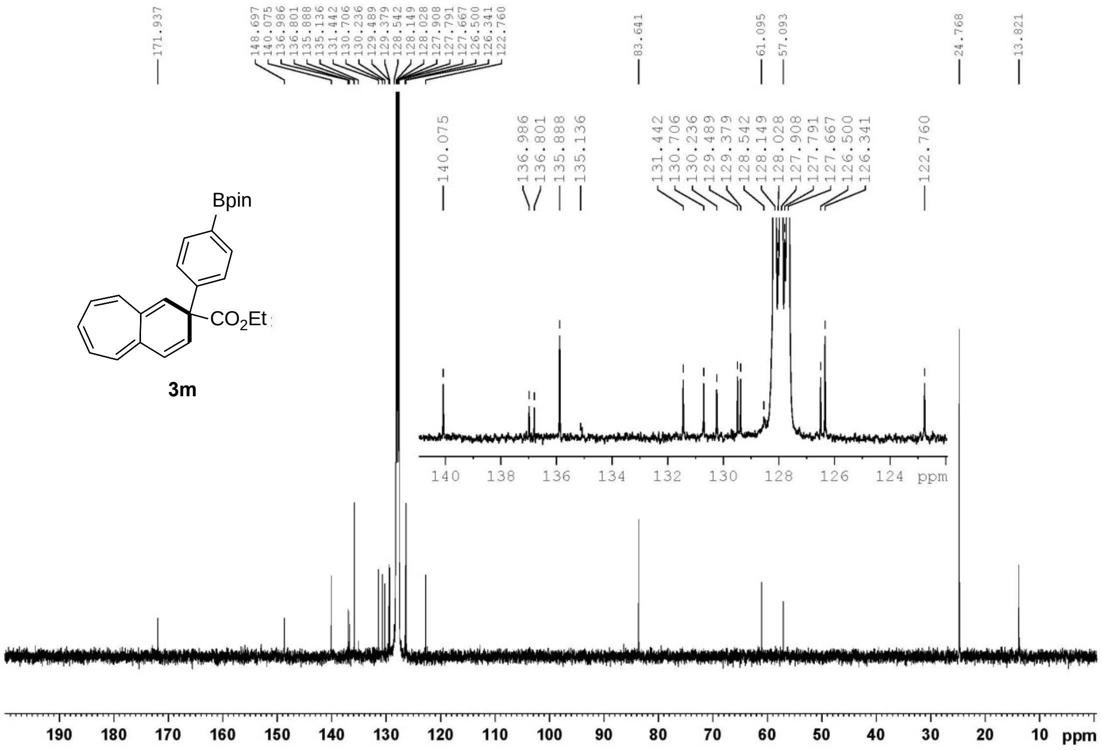

^{1}H NMR (400 MHZ, C_6D_6)

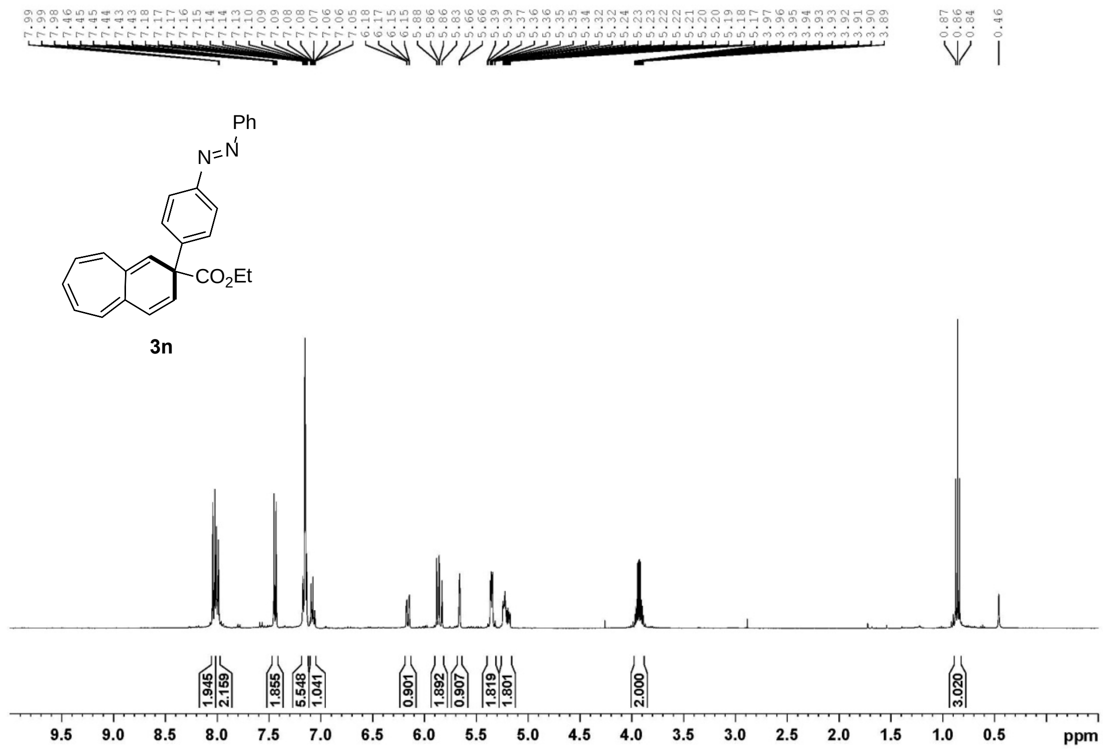

$^{13}C\{^{1}H\}$ NMR (100 MHz, $CDCl_3$)

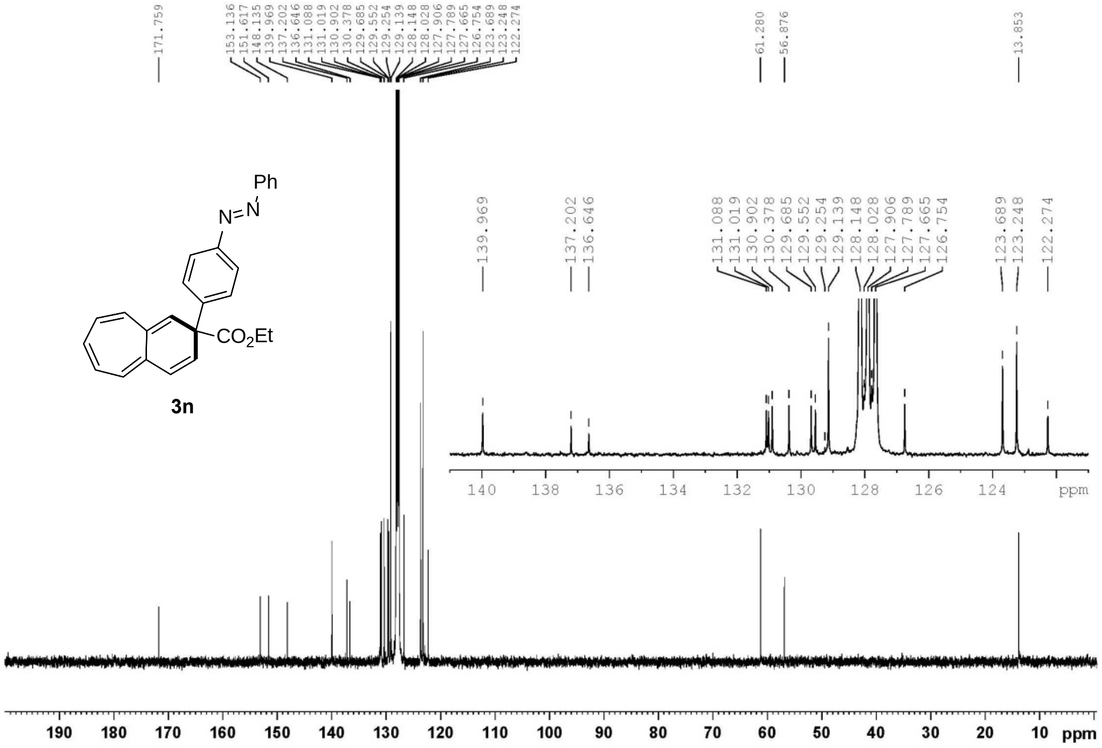

^{1}H NMR (400 MHZ, C_6D_6)

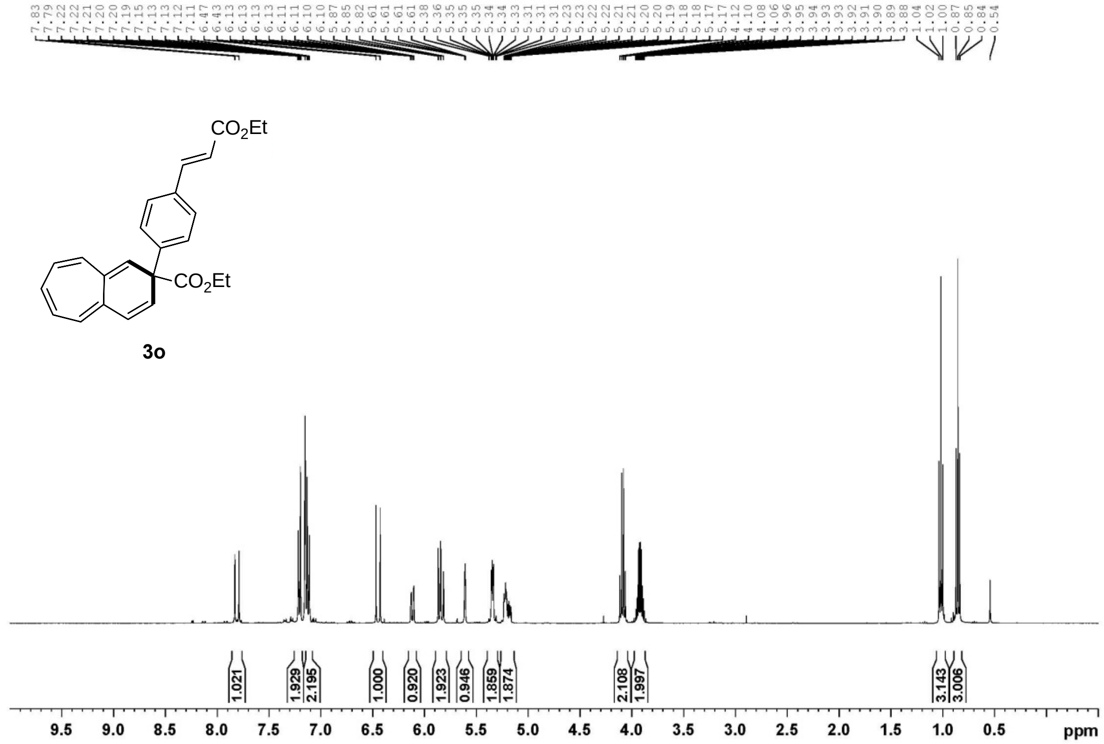

^{13}C{^{1}H} NMR (100 MHz, $CDCl_3$)

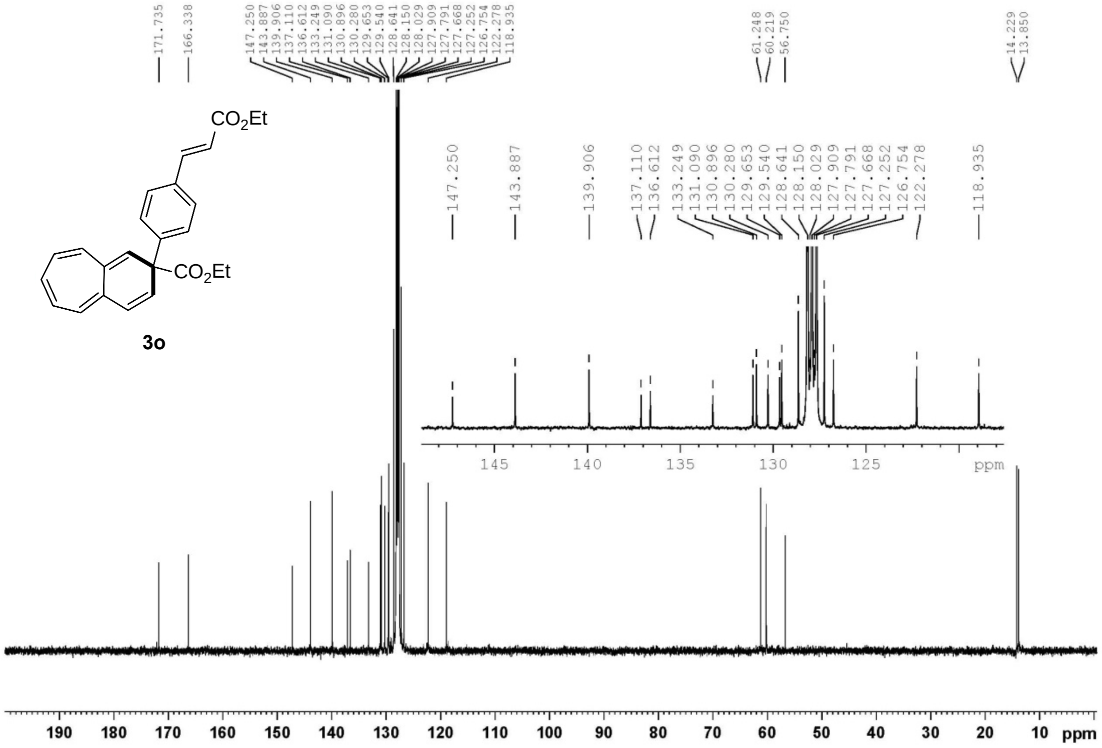

^{1}H NMR (400 MHZ, C_6D_6)

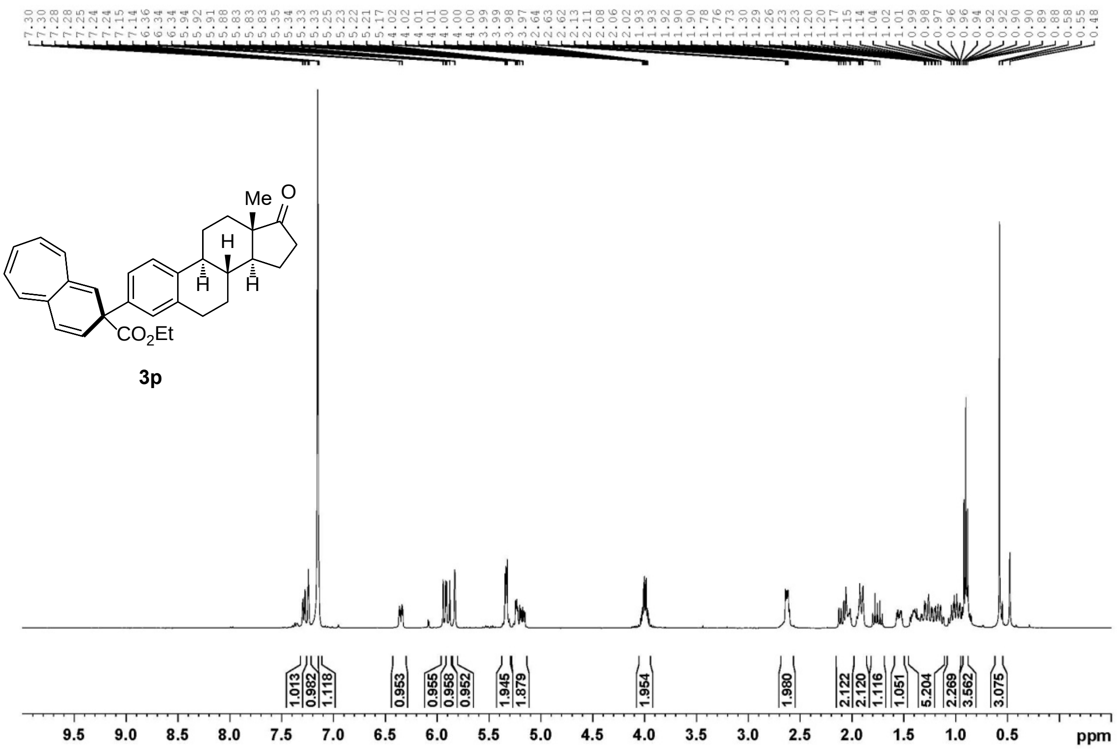

^{13}C{^{1}H}NMR (100 MHz, C_6D_6)

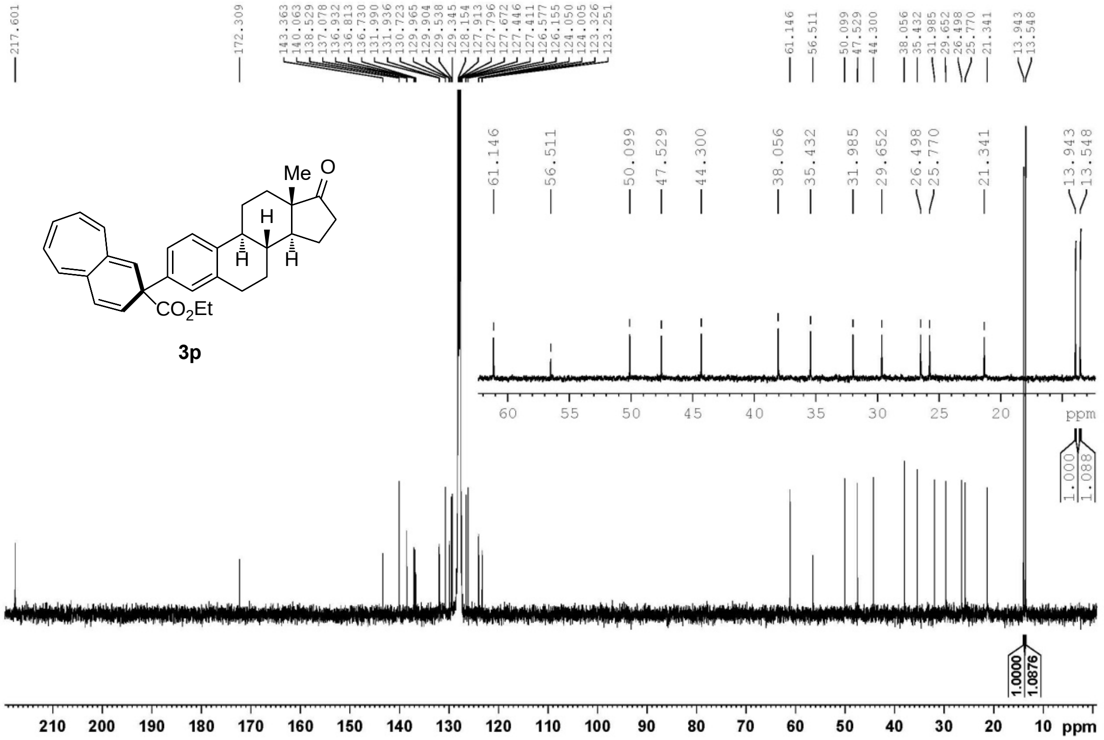

^{1}H NMR (400 MHZ, C_6D_6)

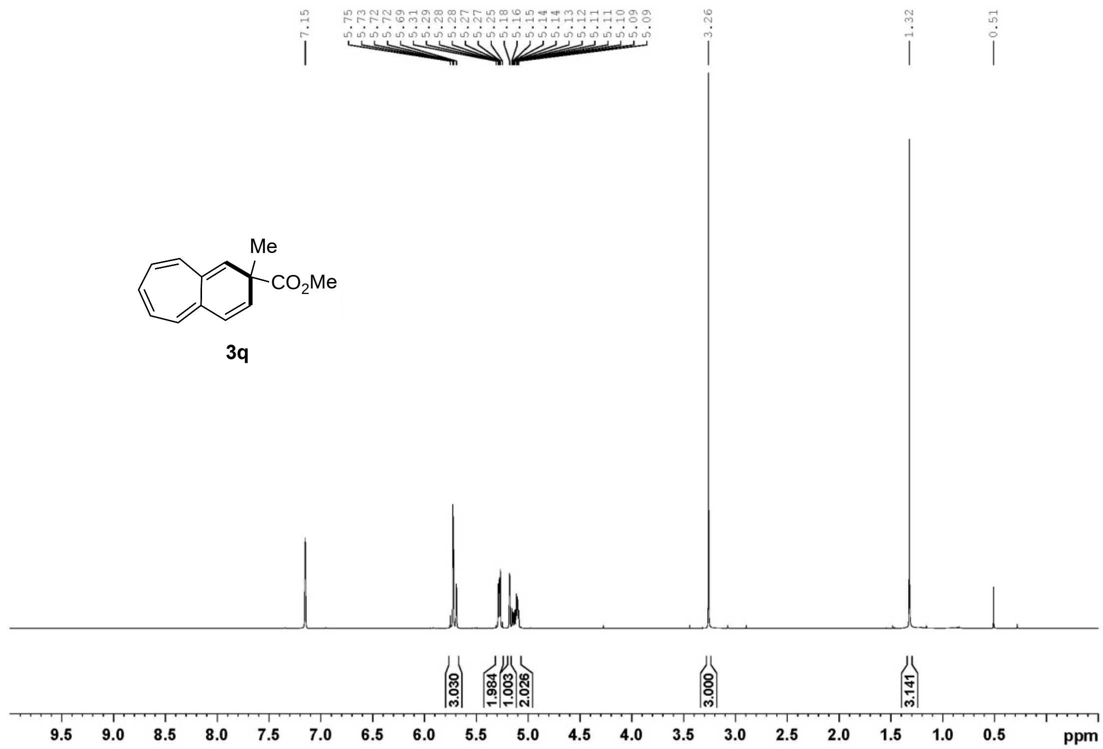

^{13}C{^{1}H} NMR (100 MHz, $CDCl_3$)

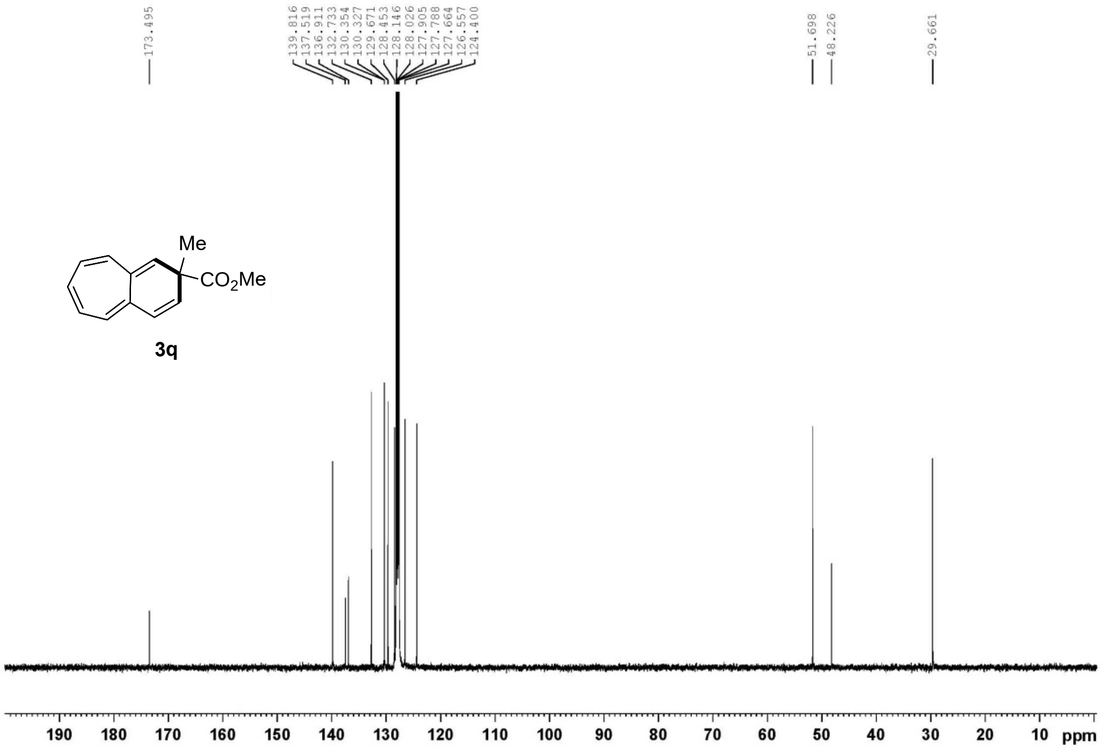

^{1}H NMR (400 MHZ, C_6D_6)

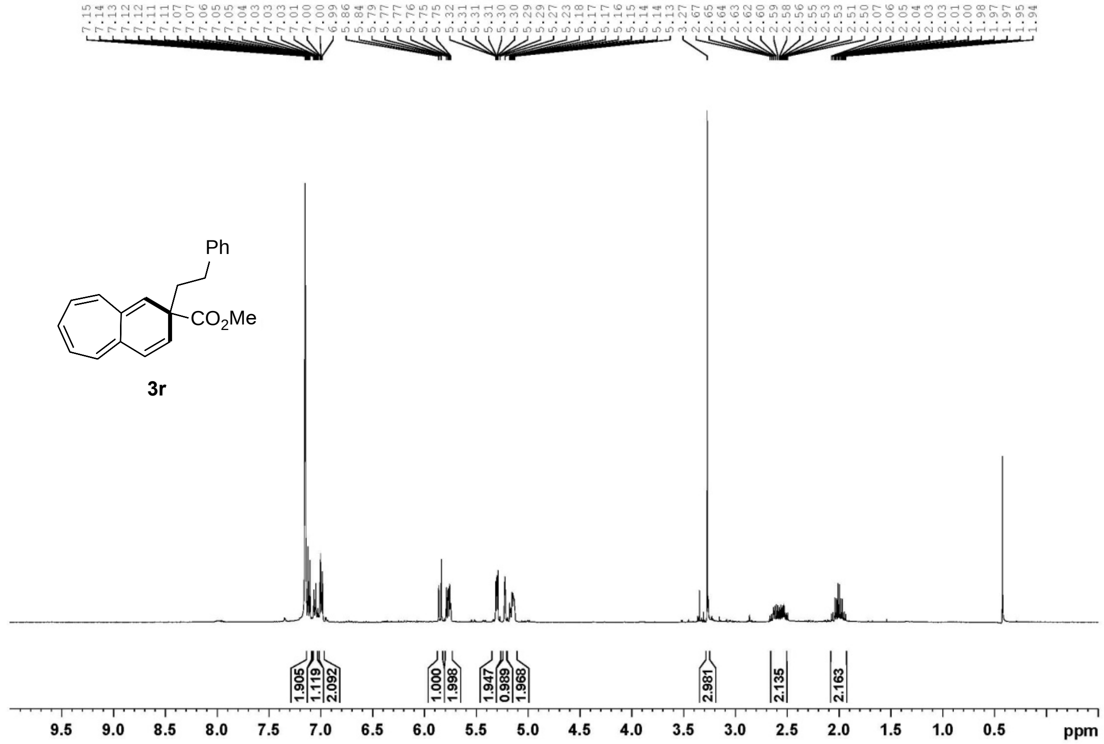

$^{13}C\{^{1}H\}$ NMR (100 MHz, $CDCl_3$)

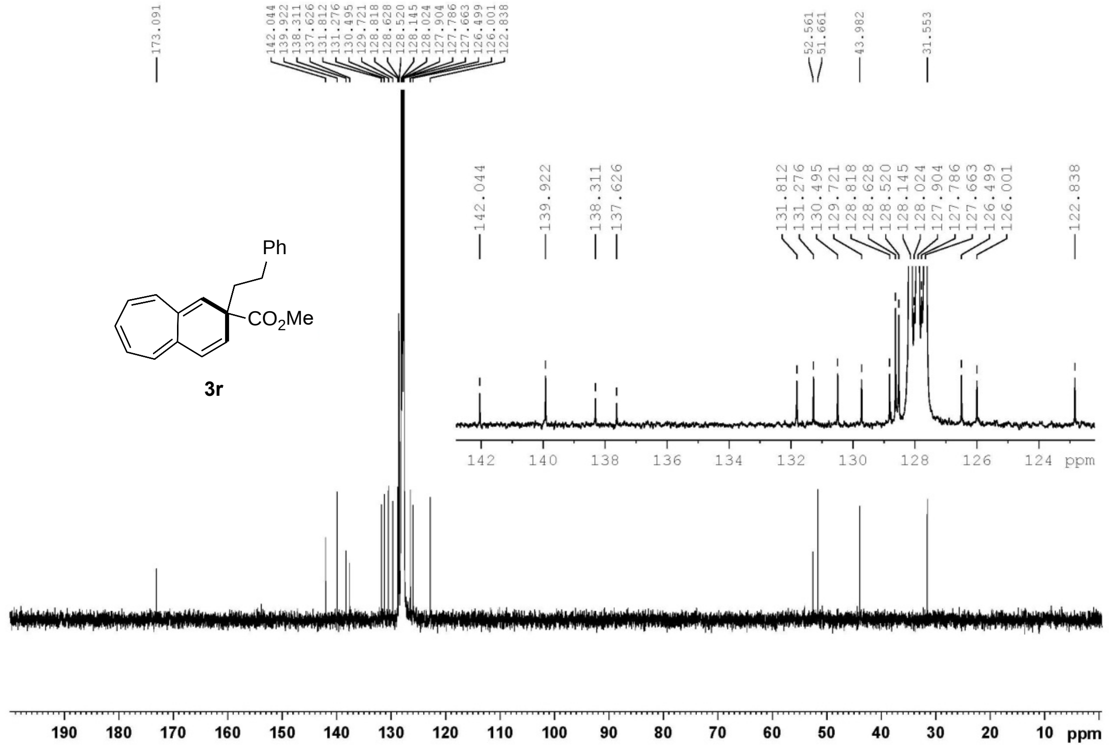

^1H NMR (400 MHZ, C_6D_6)

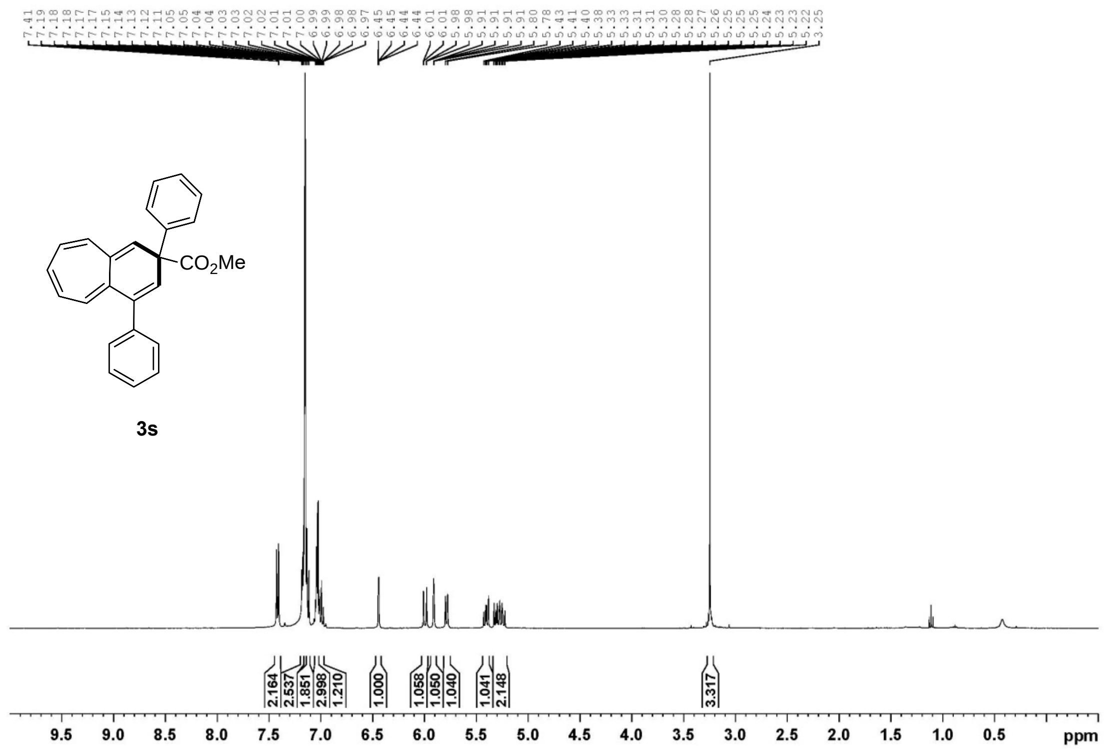

$^{13}C\{^1H\}$NMR (100 MHz, C_6D_6)

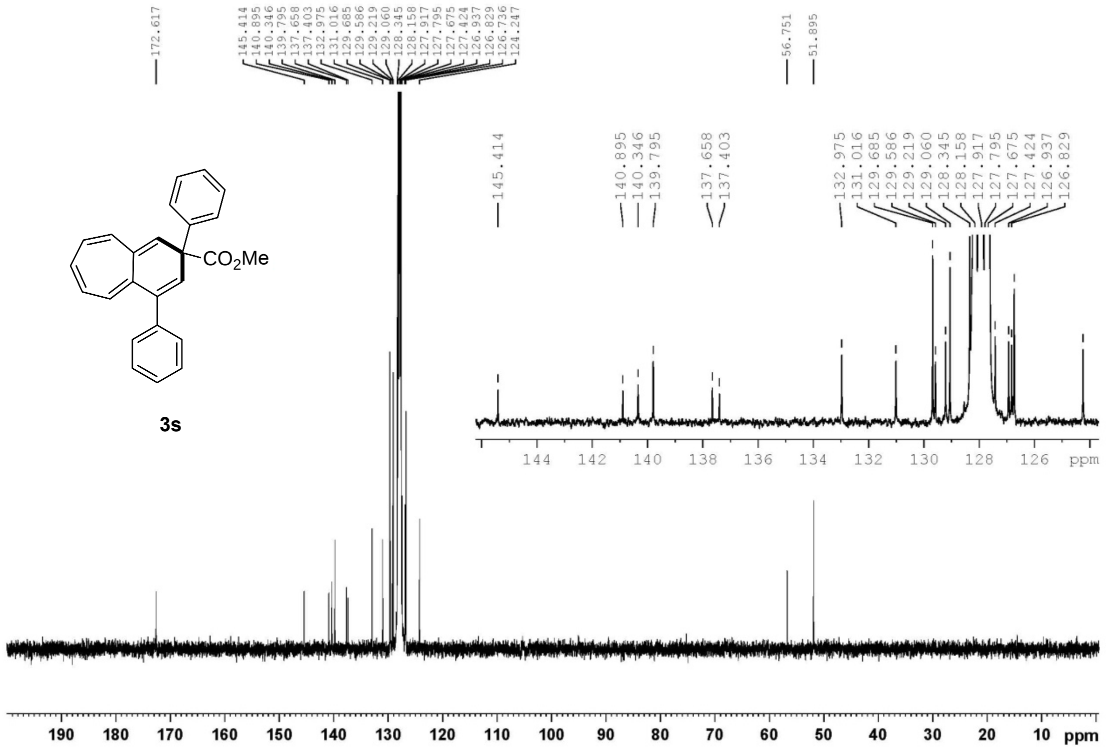

^{1}H NMR (400 MHZ, C_6D_6)

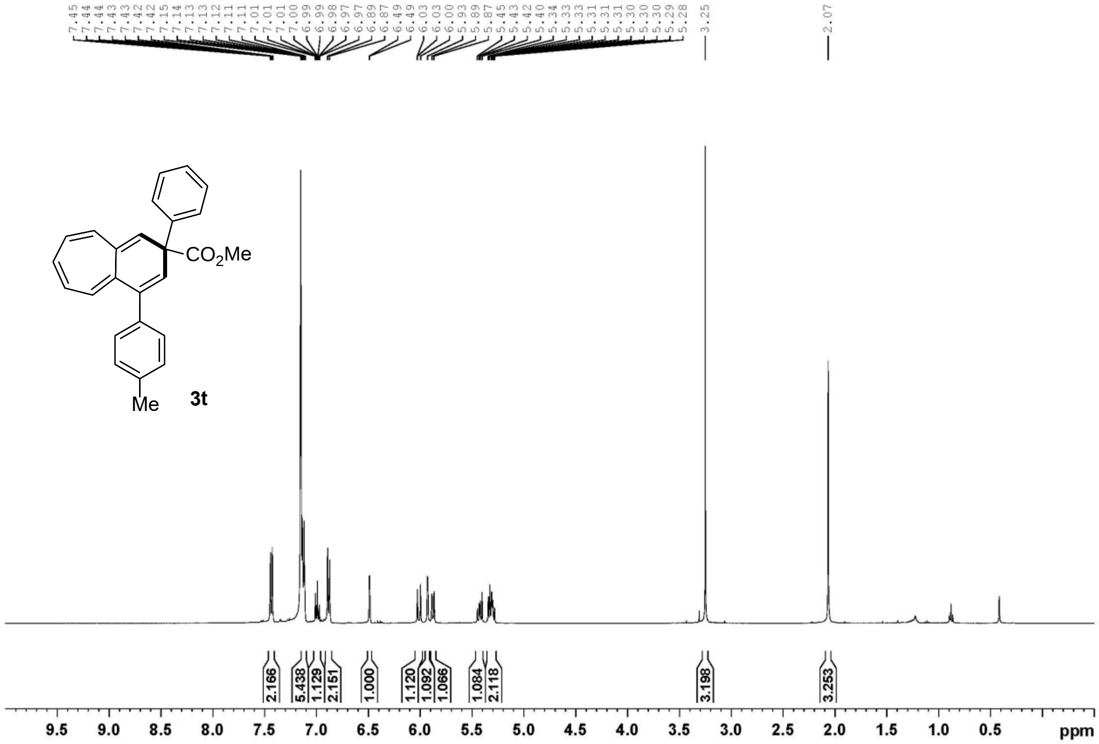

^{13}C{^{1}H}NMR (100 MHz, C_6D_6)

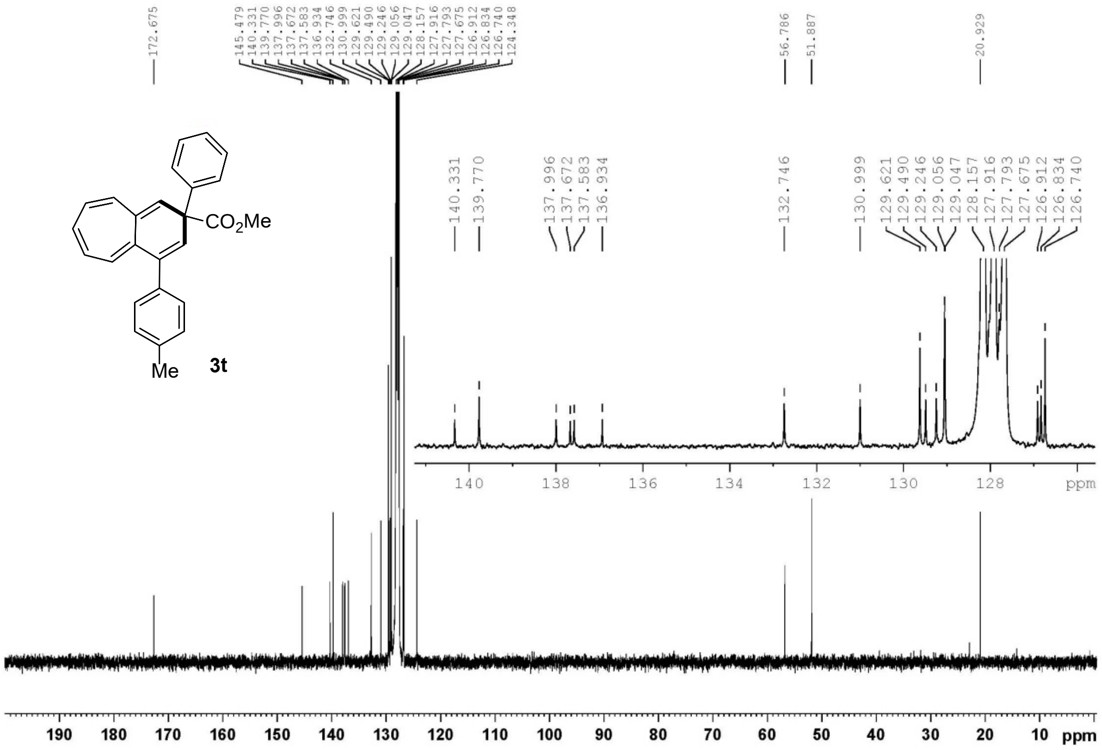

^{1}H NMR (400 MHZ, C_6D_6)

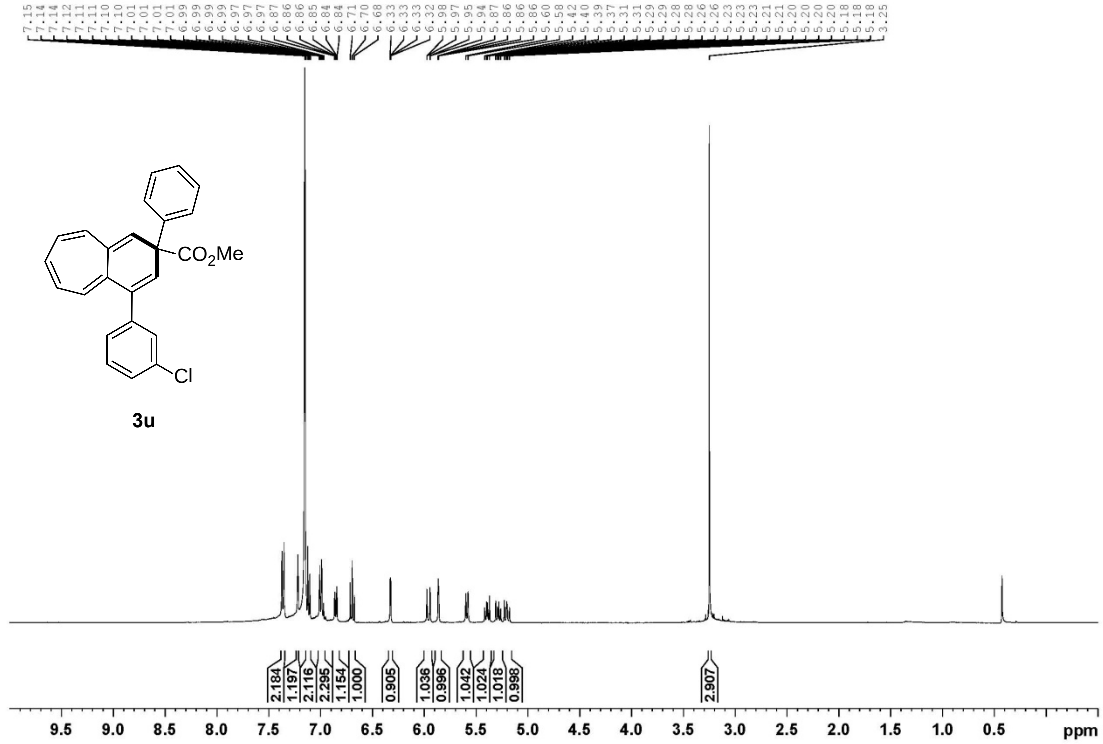

^{13}C{^{1}H}NMR (100 MHz, C_6D_6)

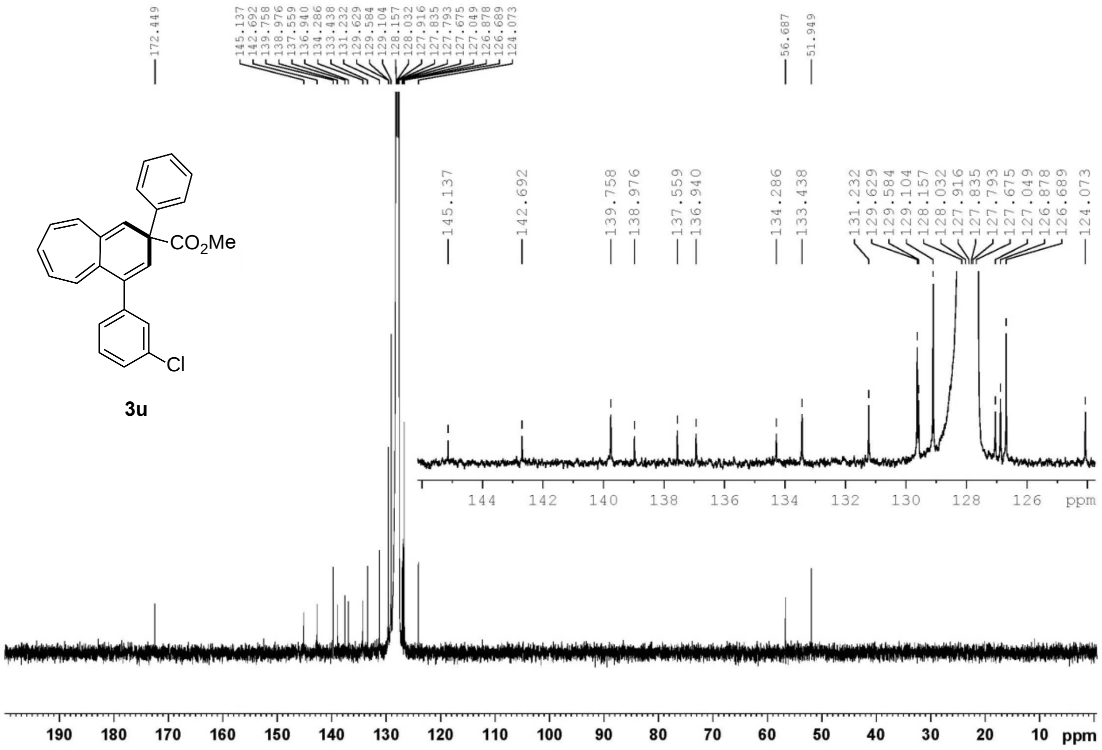

^{1}H NMR (400 MHz, $CDCl_3$)

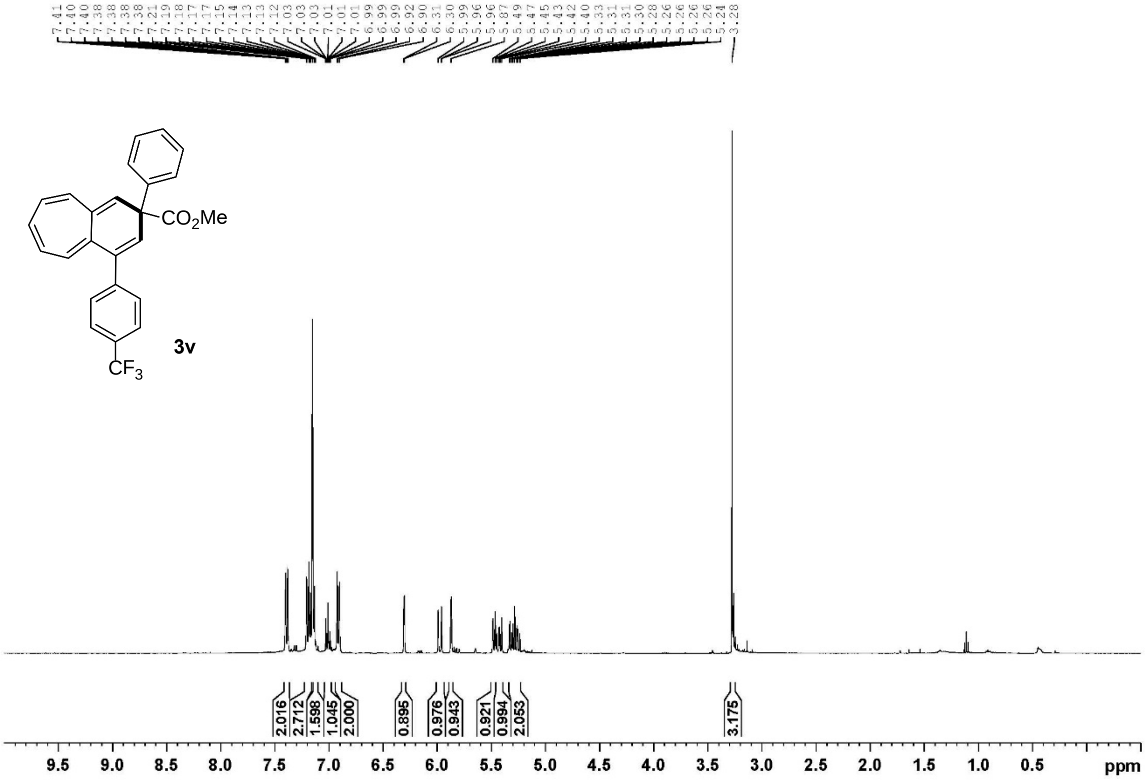

$^{13}C\{^{1}H\}$ NMR (100 MHz, $CDCl_3$)

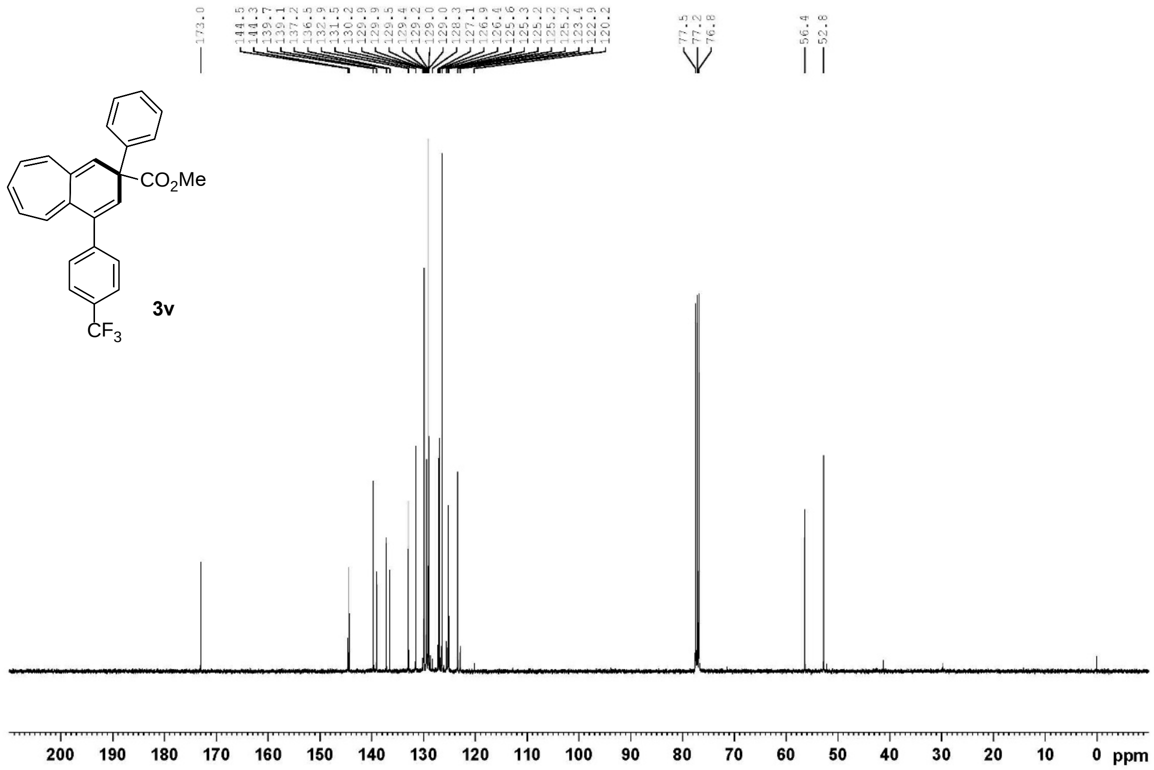

^{1}H NMR (400 MHZ, C_6D_6)

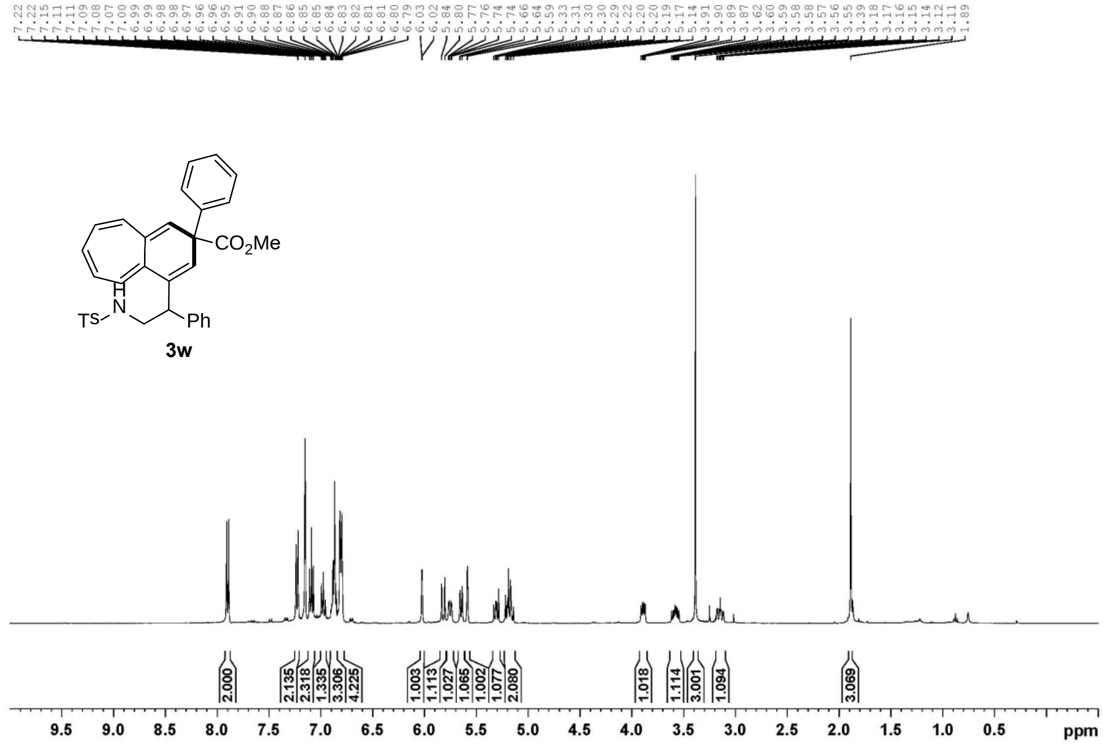

$^{13}C\{^{1}H\}$NMR (100 MHz, C_6D_6)

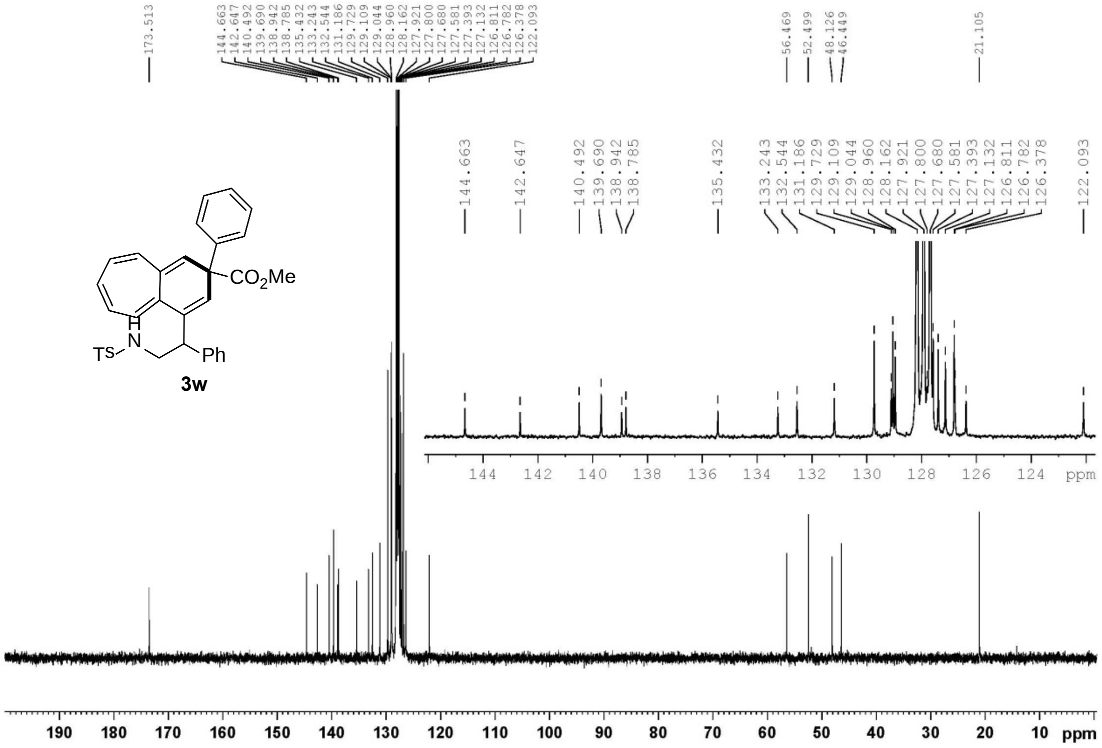

^{1}H NMR (400 MHZ, C_6D_6)

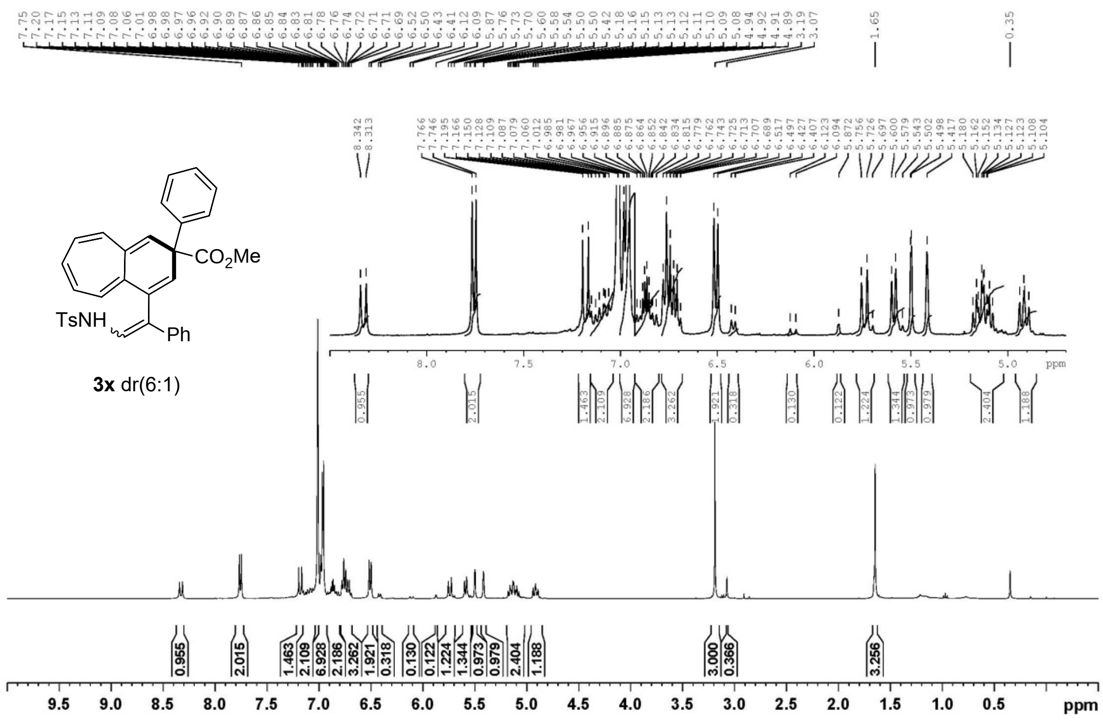

$^{13}C\{^{1}H\}$NMR (100 MHz, C_6D_6)

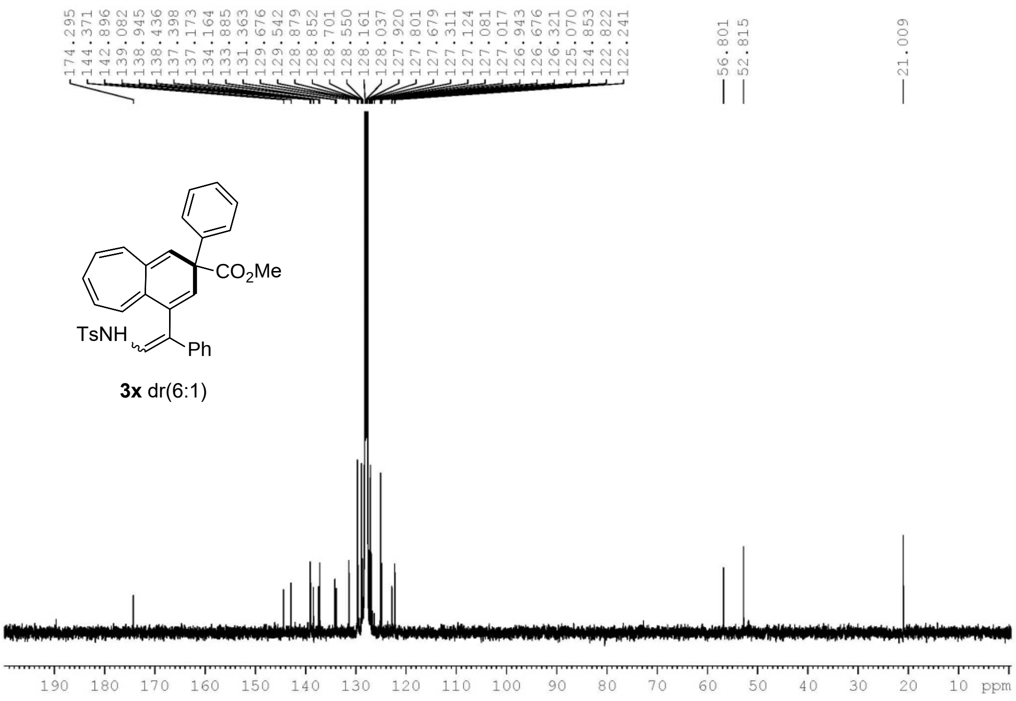

^{1}H NMR (400 MHz, $CDCl_3$)

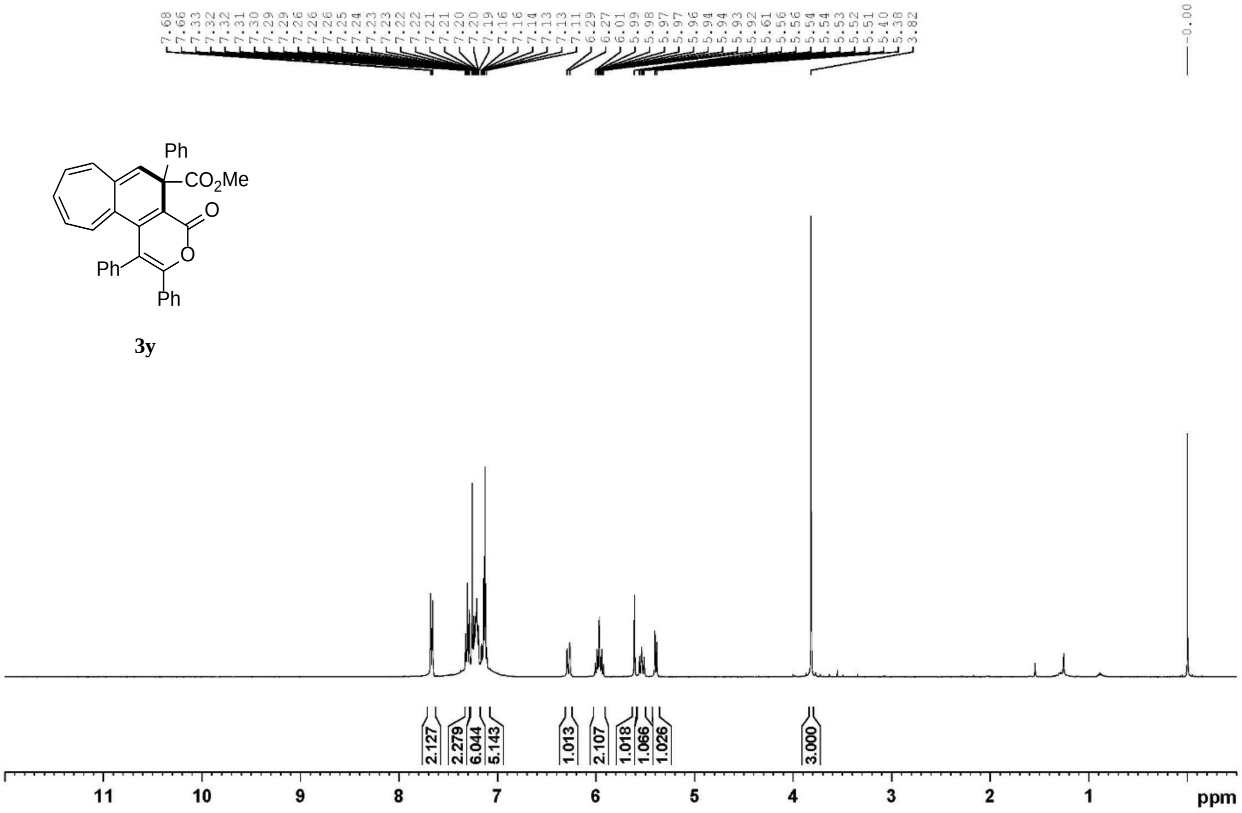

$^{13}C\{^1H\}$ NMR (100 MHz, $CDCl_3$)

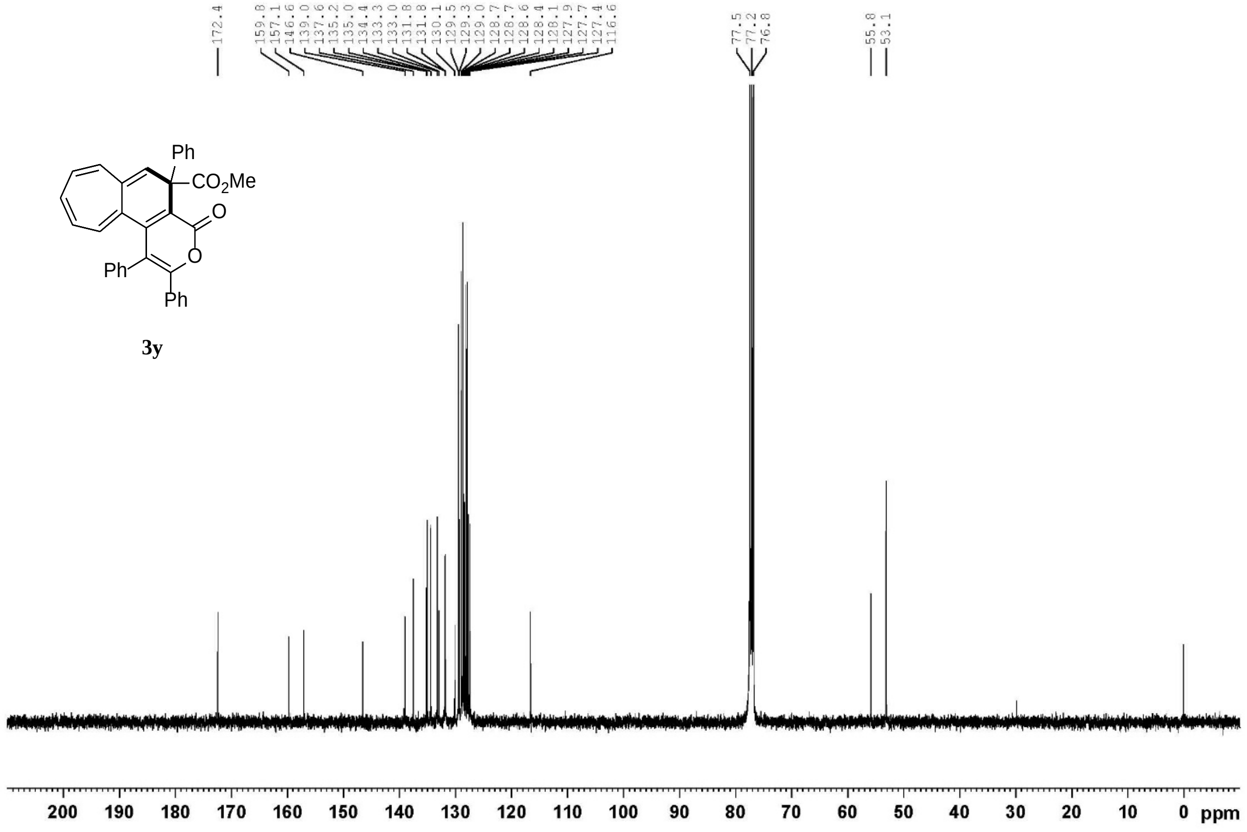

^1H NMR (400 MHZ, C_6D_6)

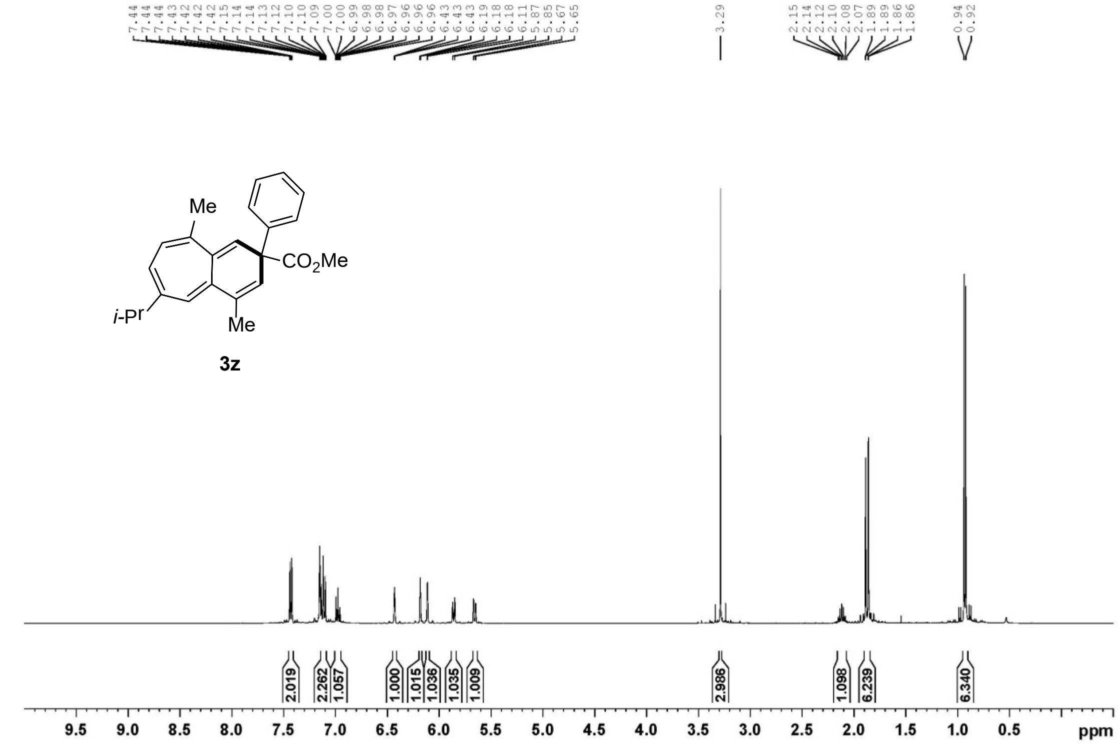

$^{13}C\{^1H\}$NMR (100 MHz, C_6D_6)

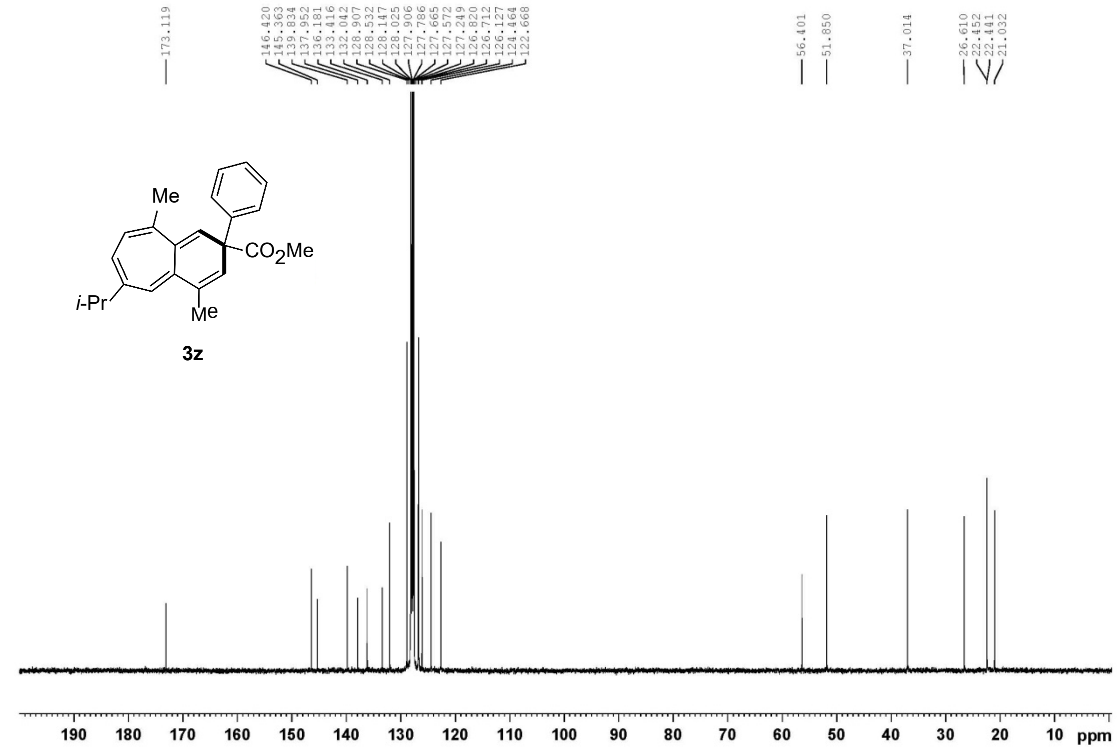

^{1}H NMR (400 MHZ, C_6D_6)

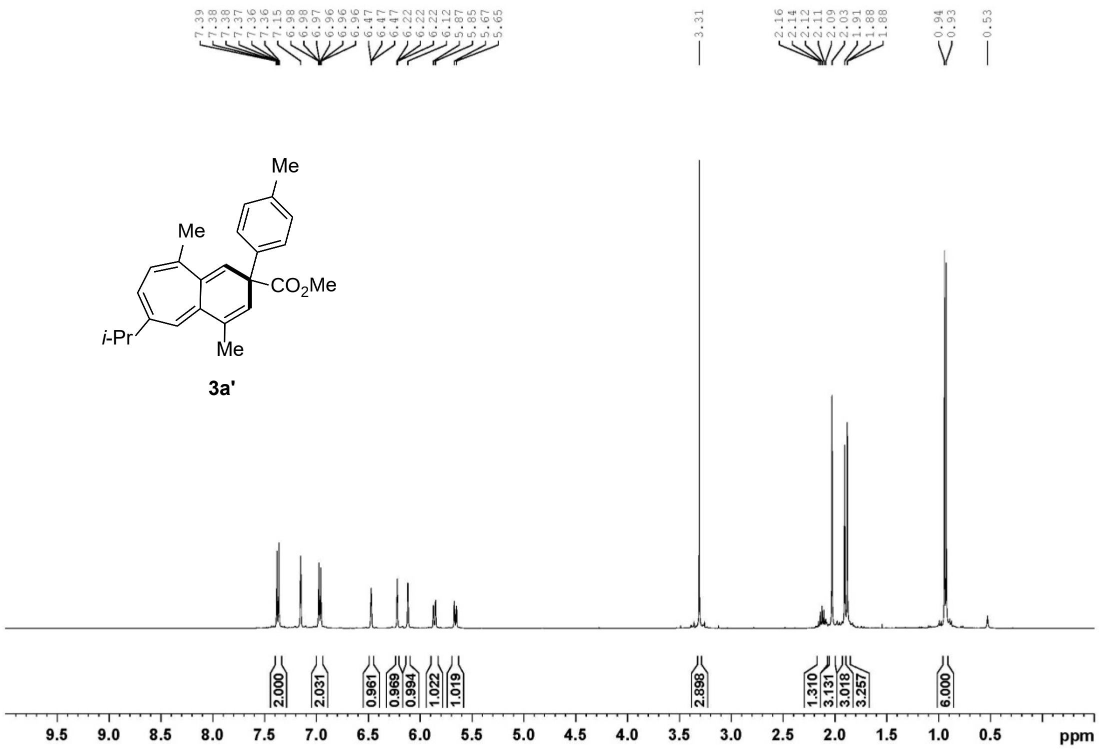

$^{13}C\{^{1}H\}$NMR (100 MHz, C_6D_6)

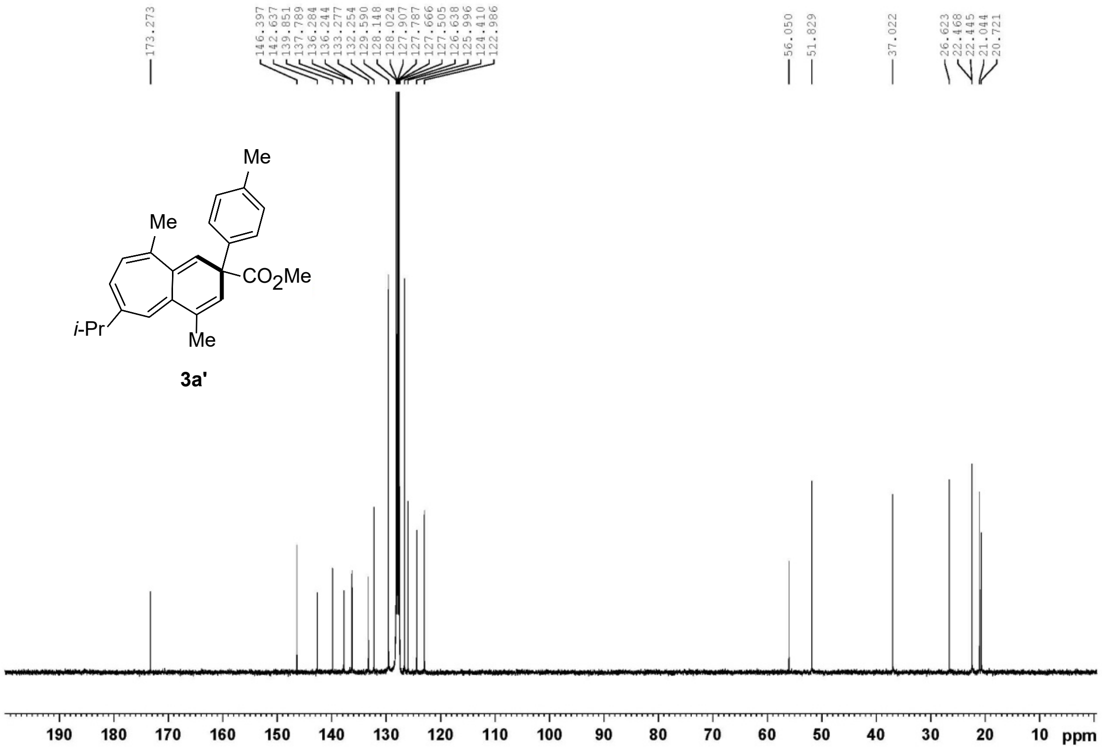

^{1}H NMR (400 MHZ, C_6D_6)

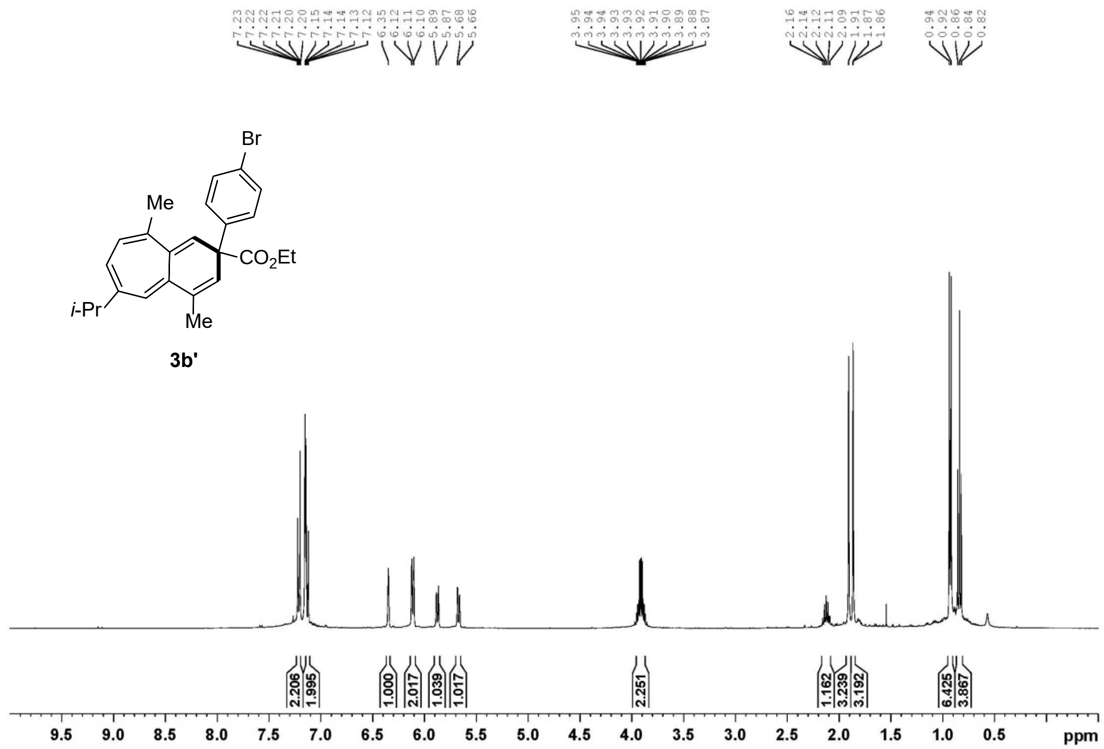

^{13}C{^{1}H}NMR (100 MHz, C_6D_6)

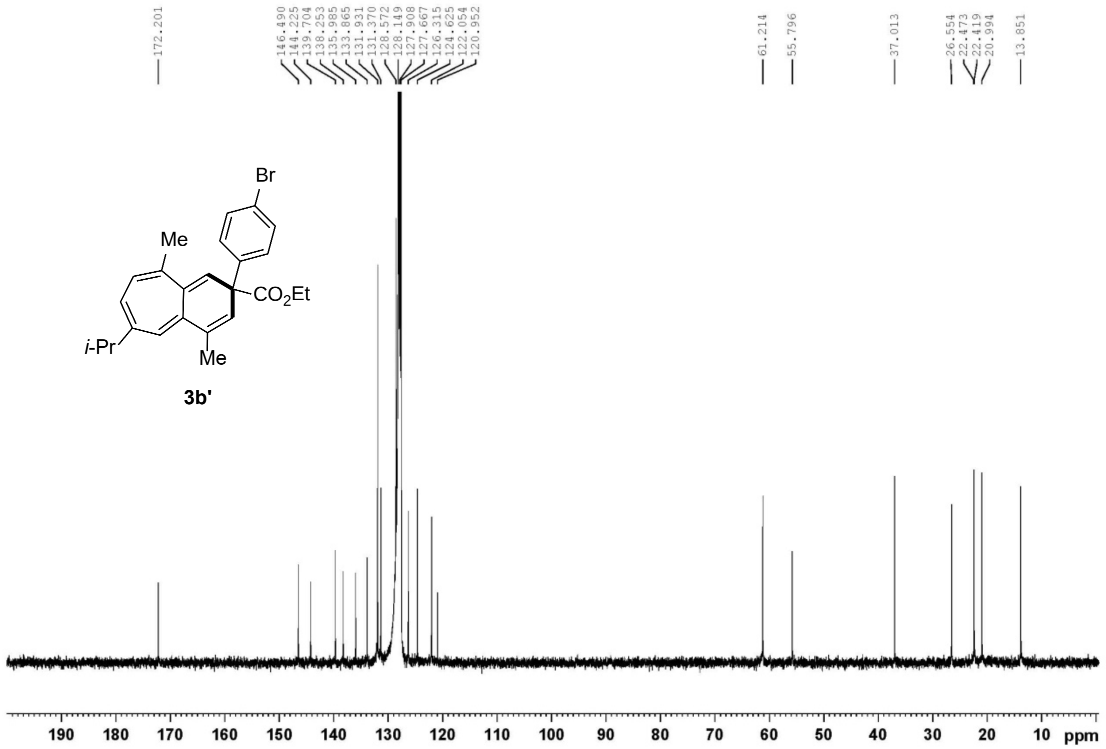

^1H NMR (400 MHZ, C_6D_6)

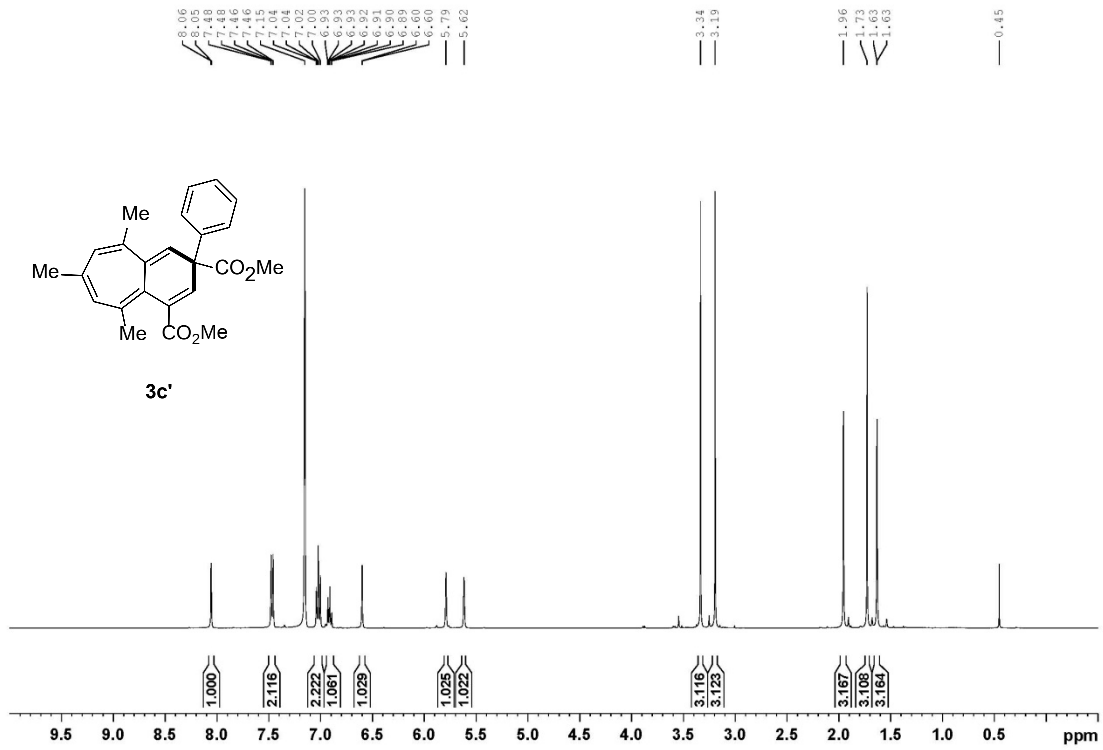

$^{13}C\{^1H\}$NMR (100 MHz, C_6D_6)

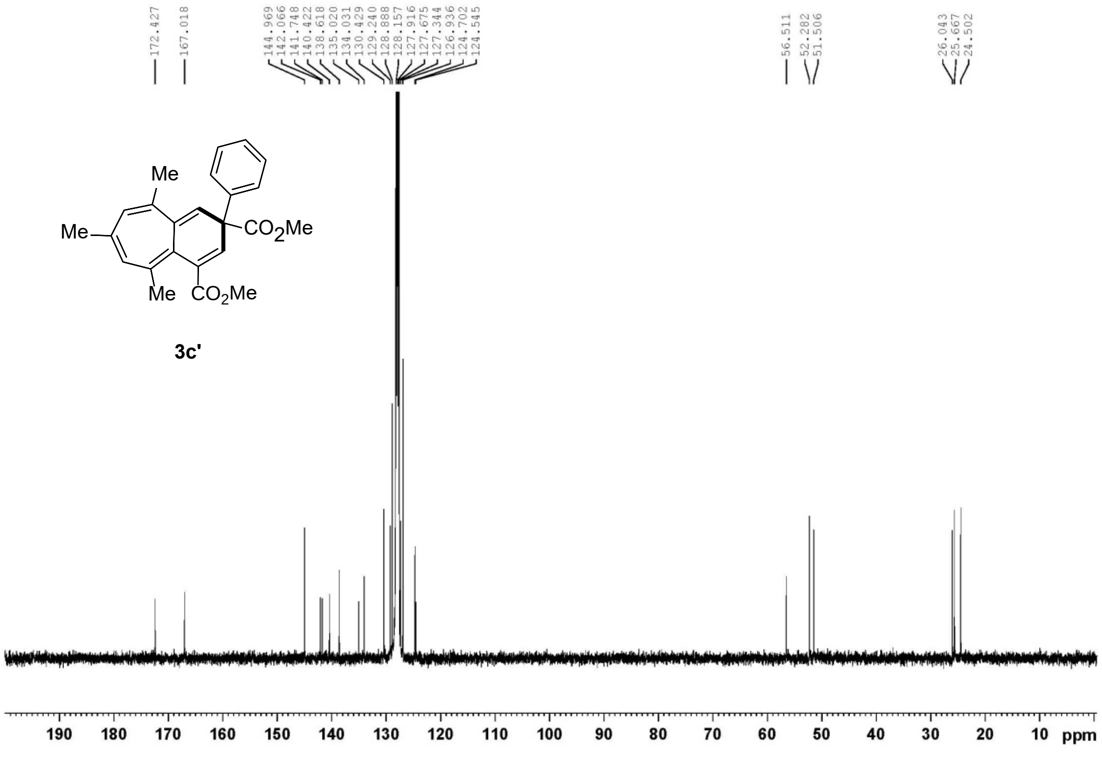

^{1}H NMR (400 MHZ, C_6D_6)

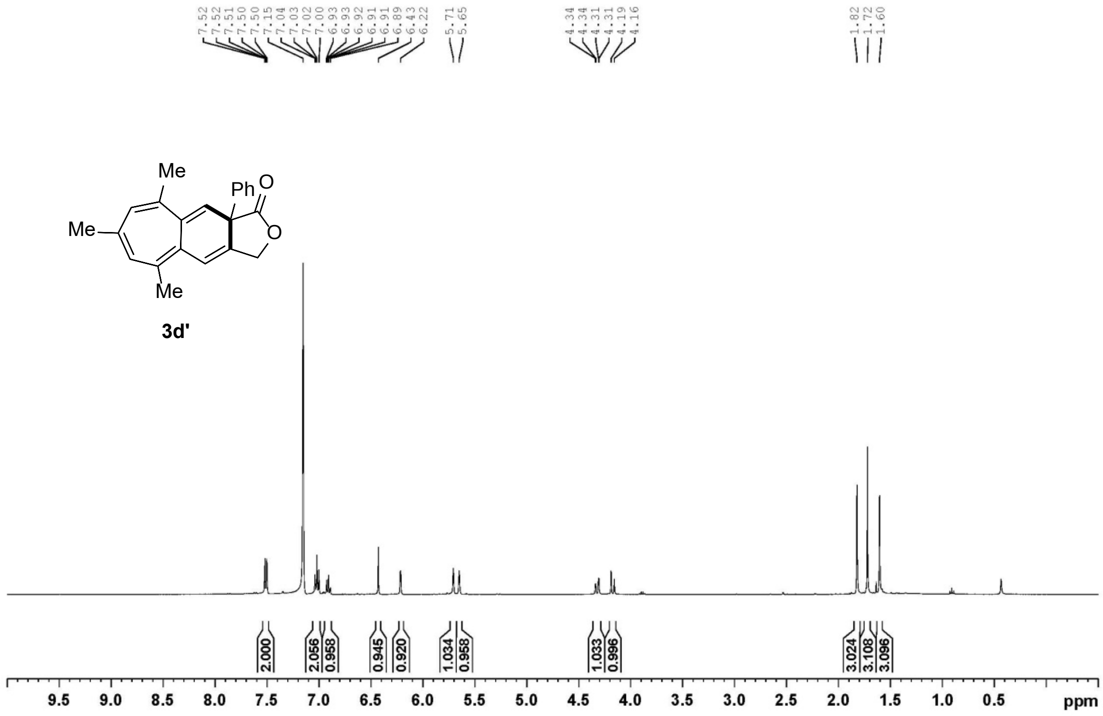

$^{13}C\{^{1}H\}$NMR (100 MHz, C_6D_6)

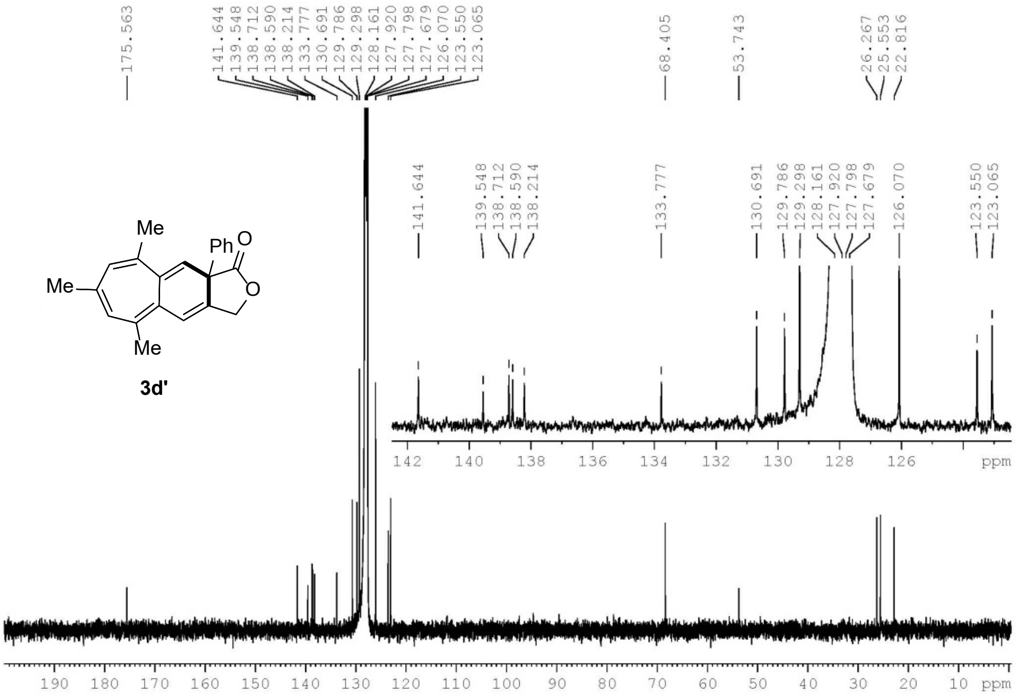

^{1}H NMR (400 MHz, $CDCl_3$)

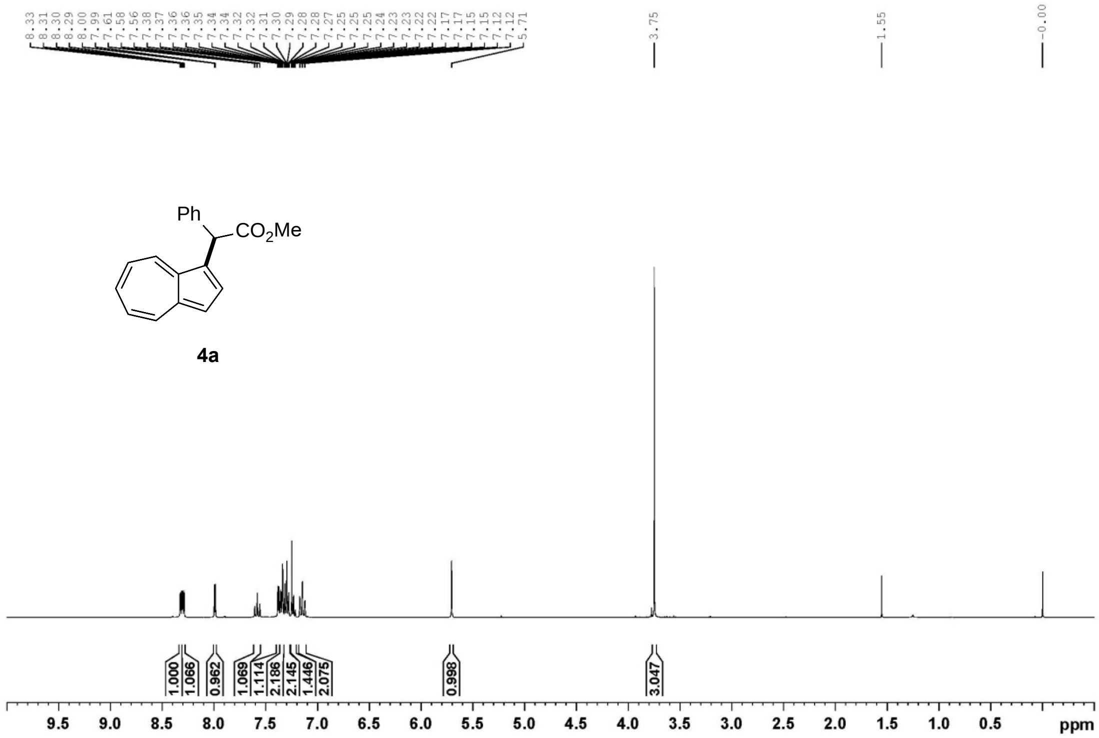

$^{13}C\{^{1}H\}$ NMR (100 MHz, $CDCl_3$)

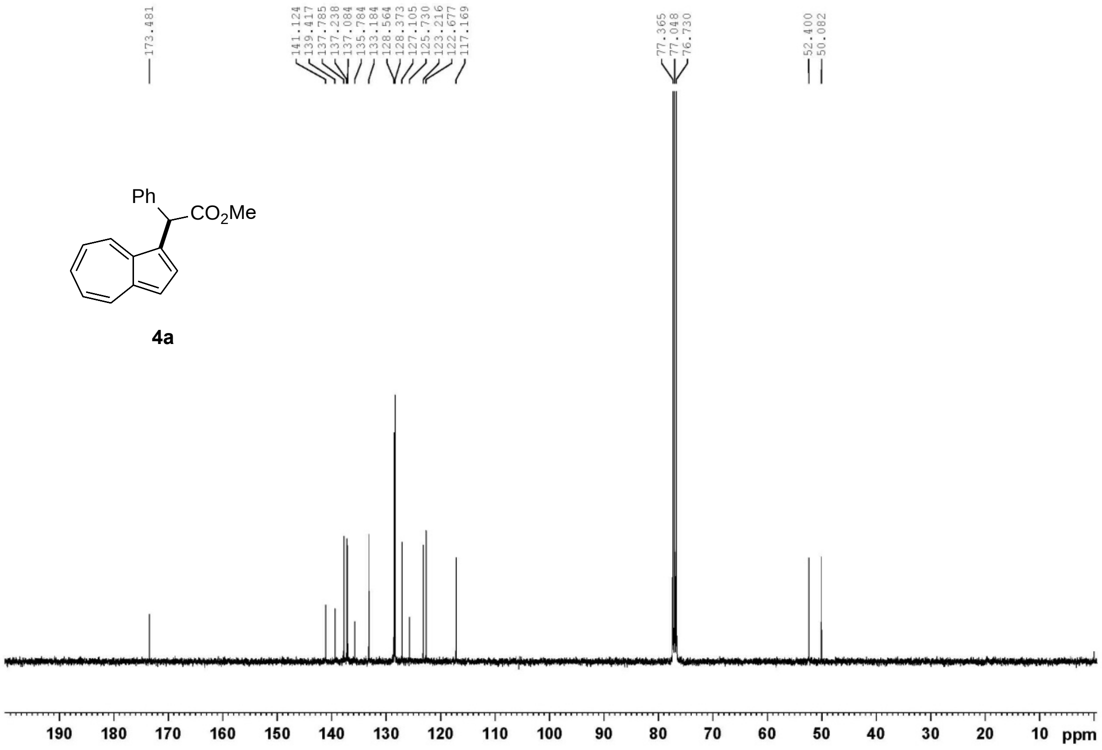

^{1}H NMR (400 MHz, $CDCl_3$)

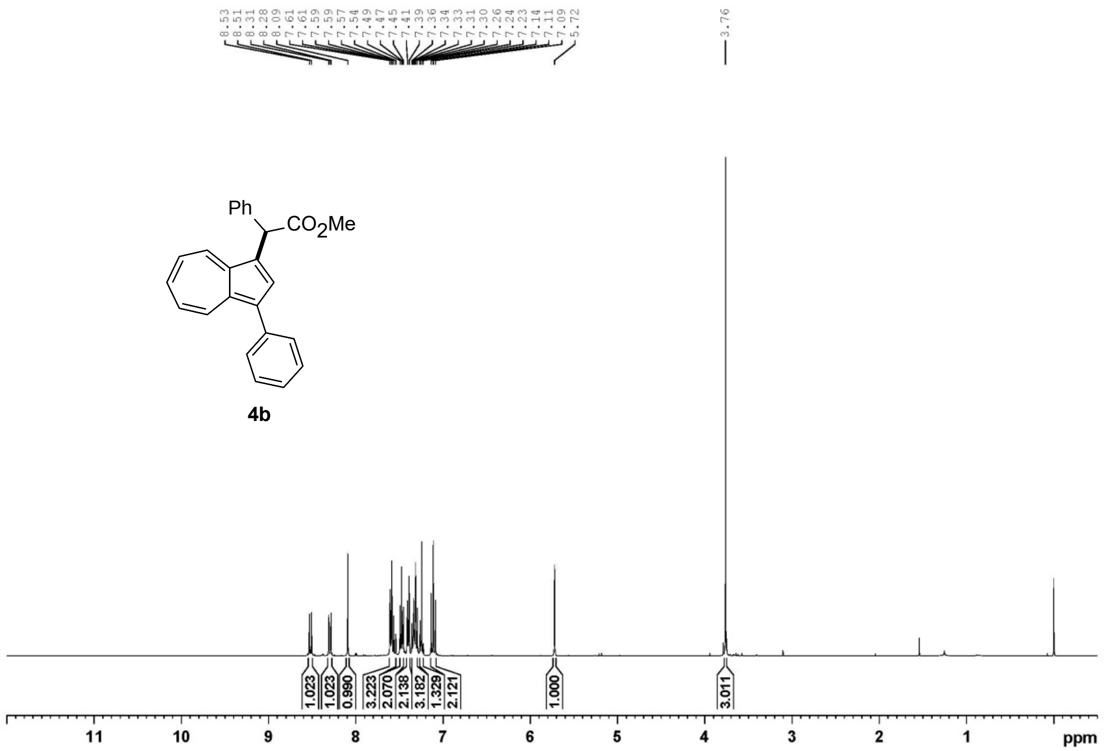

$^{13}C\{^{1}H\}$ NMR (100 MHz, $CDCl_3$)

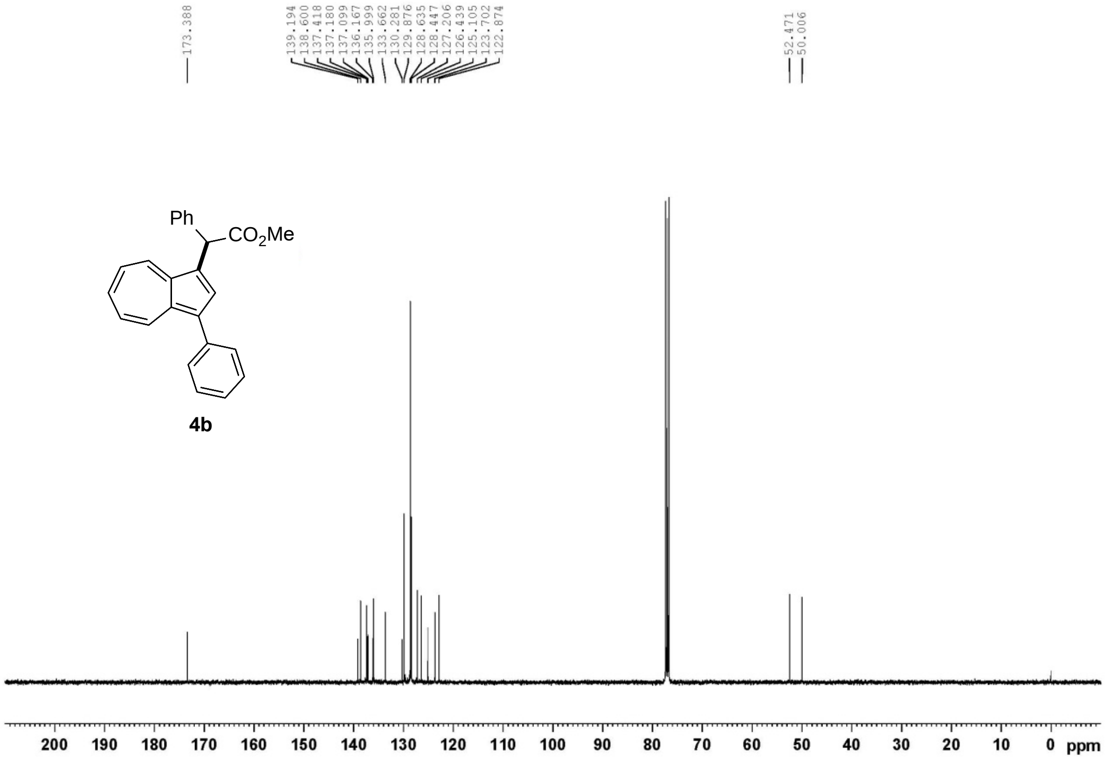

^{1}H NMR (400 MHz, $CDCl_3$)

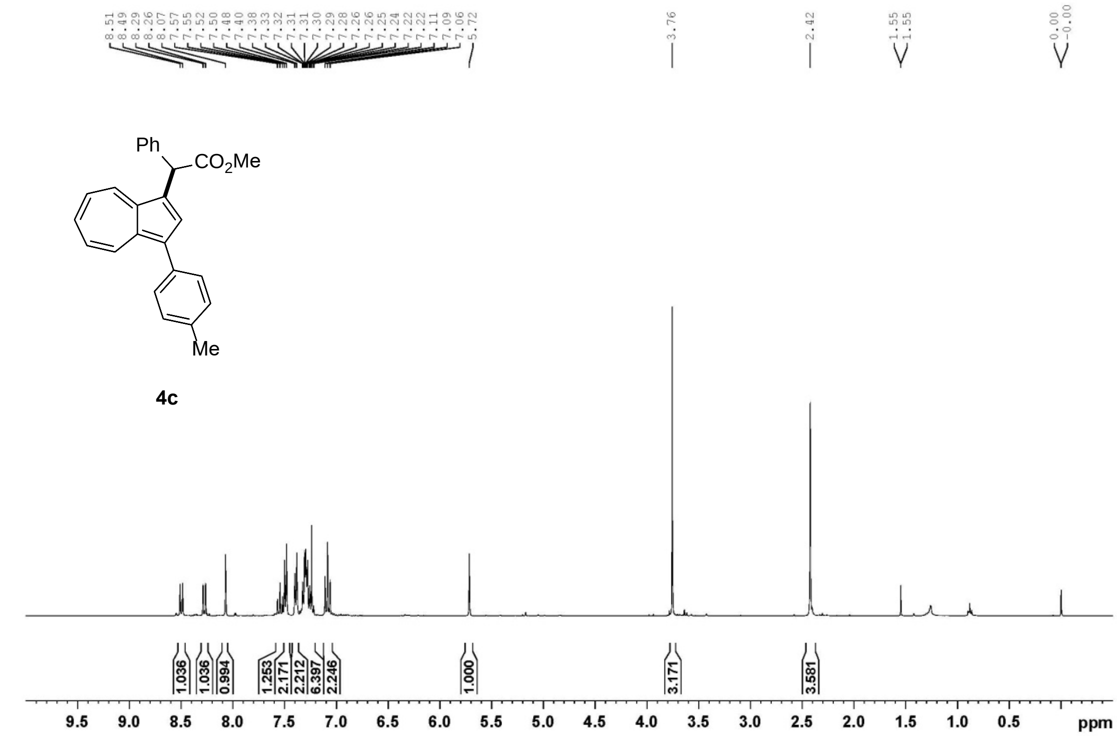

$^{13}C\{^{1}H\}$ NMR (100 MHz, $CDCl_3$)

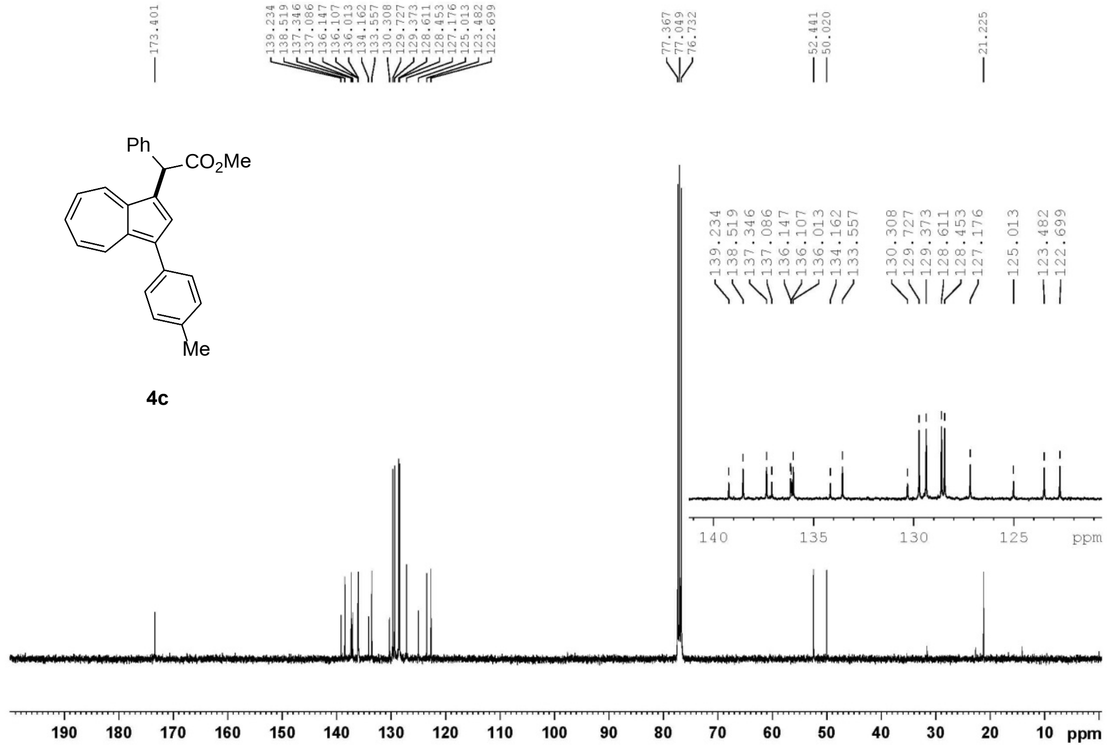

^{1}H NMR (400 MHz, $CDCl_3$)

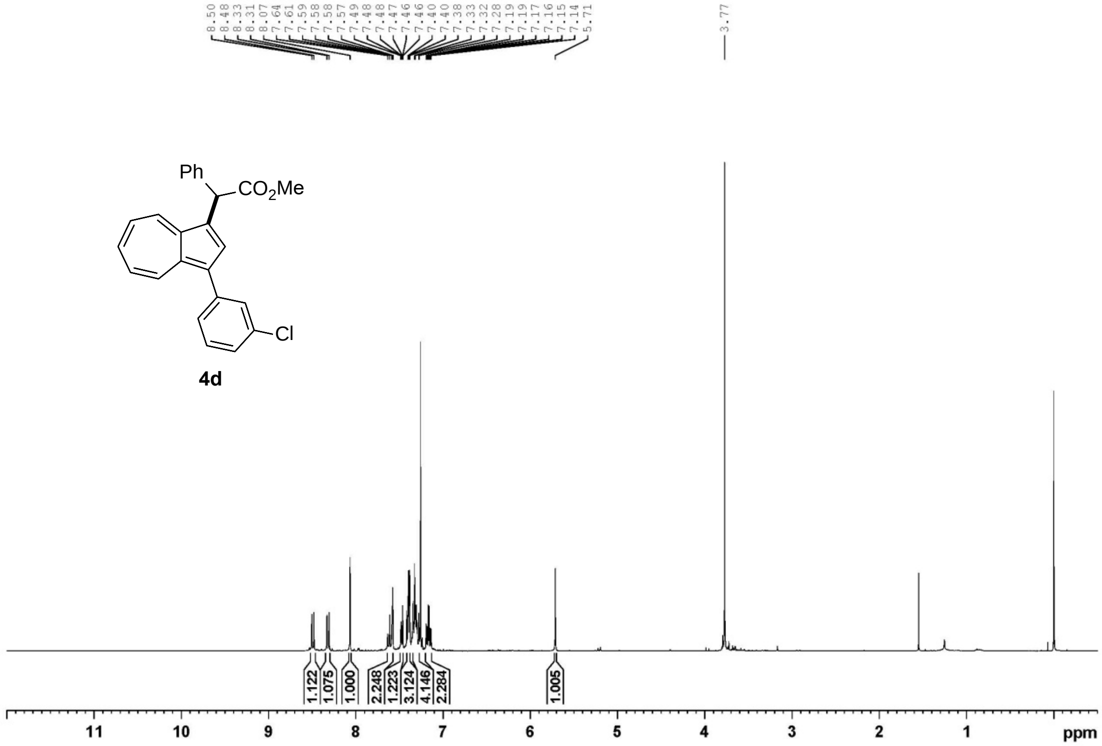

$^{13}C\{^1H\}$ NMR (100 MHz, $CDCl_3$)

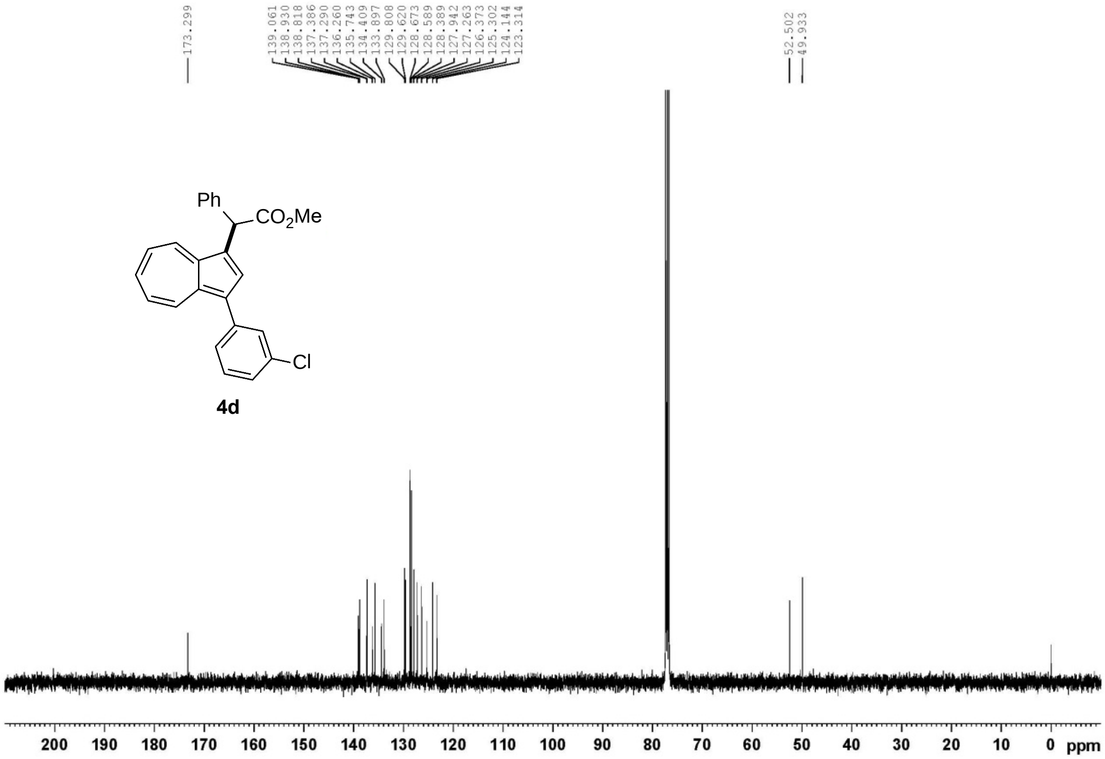

^1H NMR (400 MHz, $CDCl_3$)

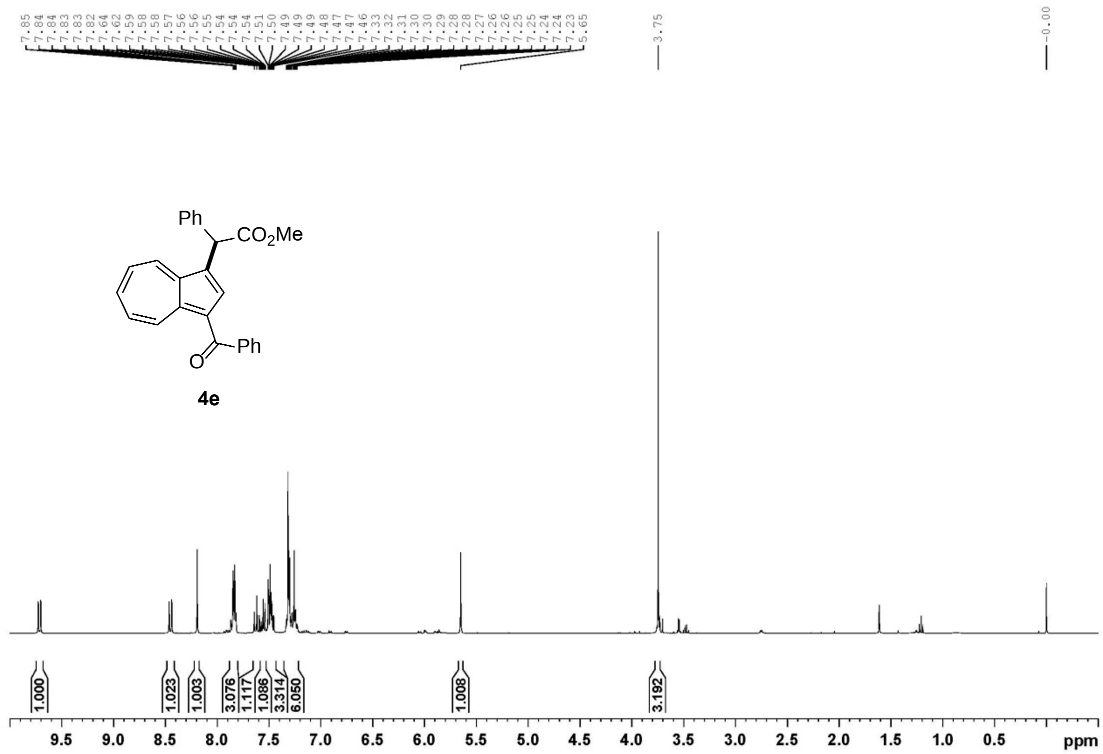

$^{13}C\{^1H\}$ NMR (100 MHz, $CDCl_3$)

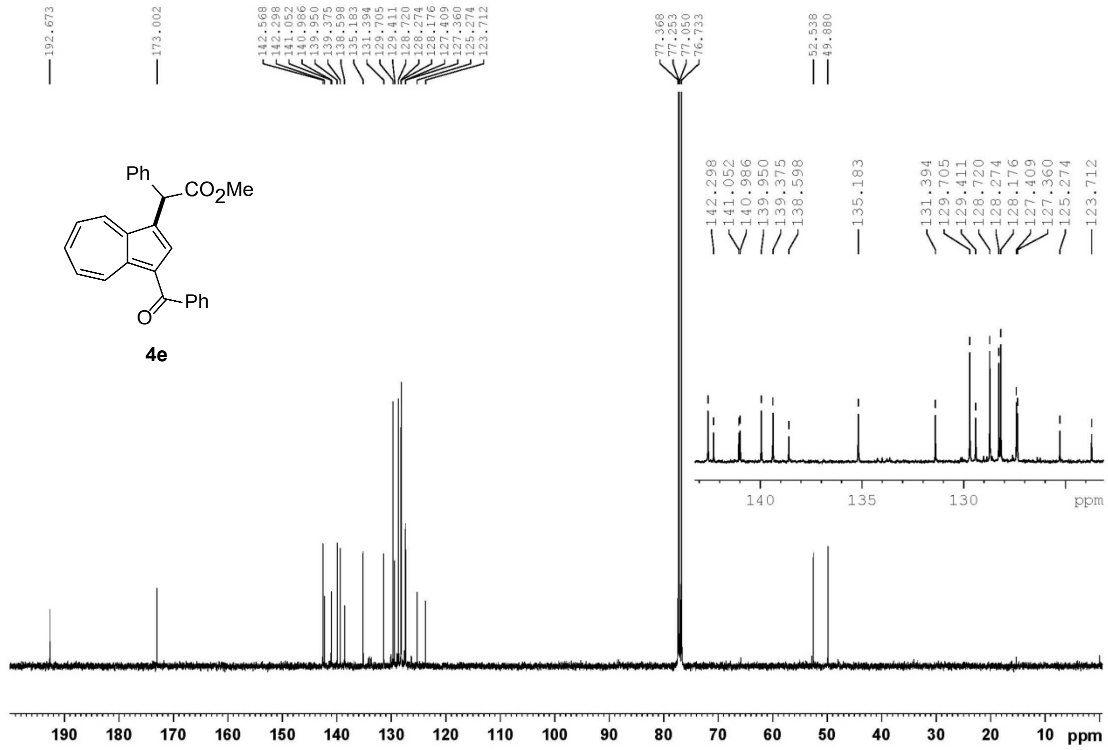

^1H NMR (400 MHz, $CDCl_3$)

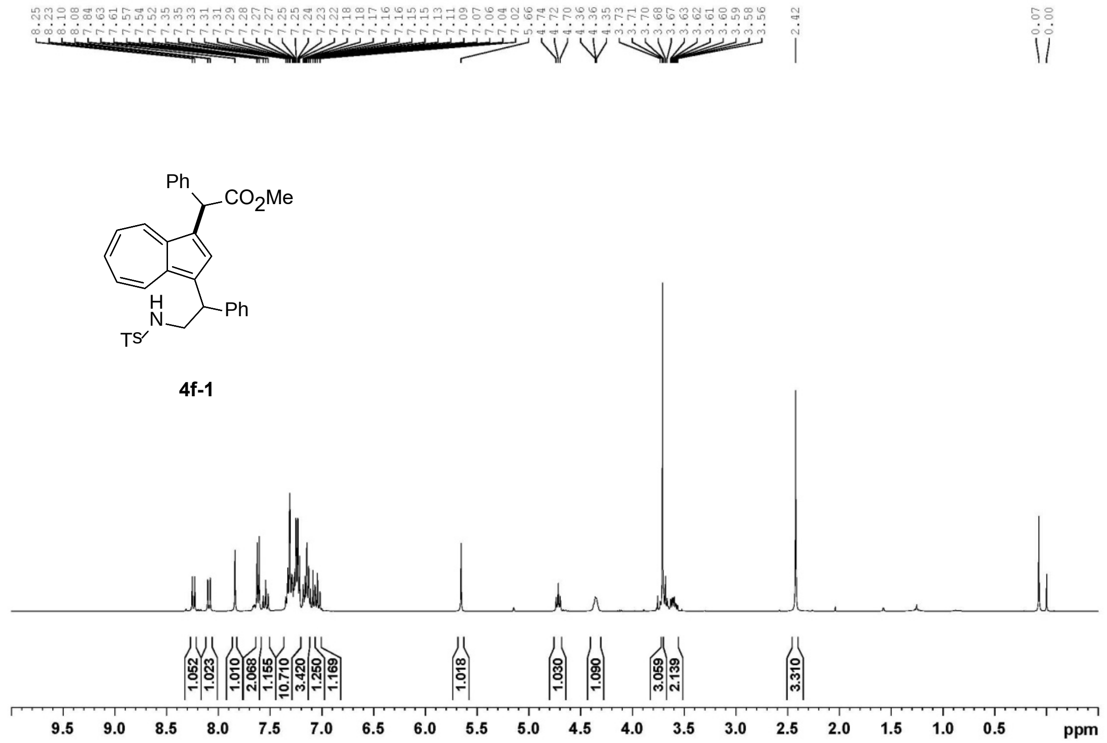

$^{13}C\{^1H\}$ NMR (100 MHz, $CDCl_3$)

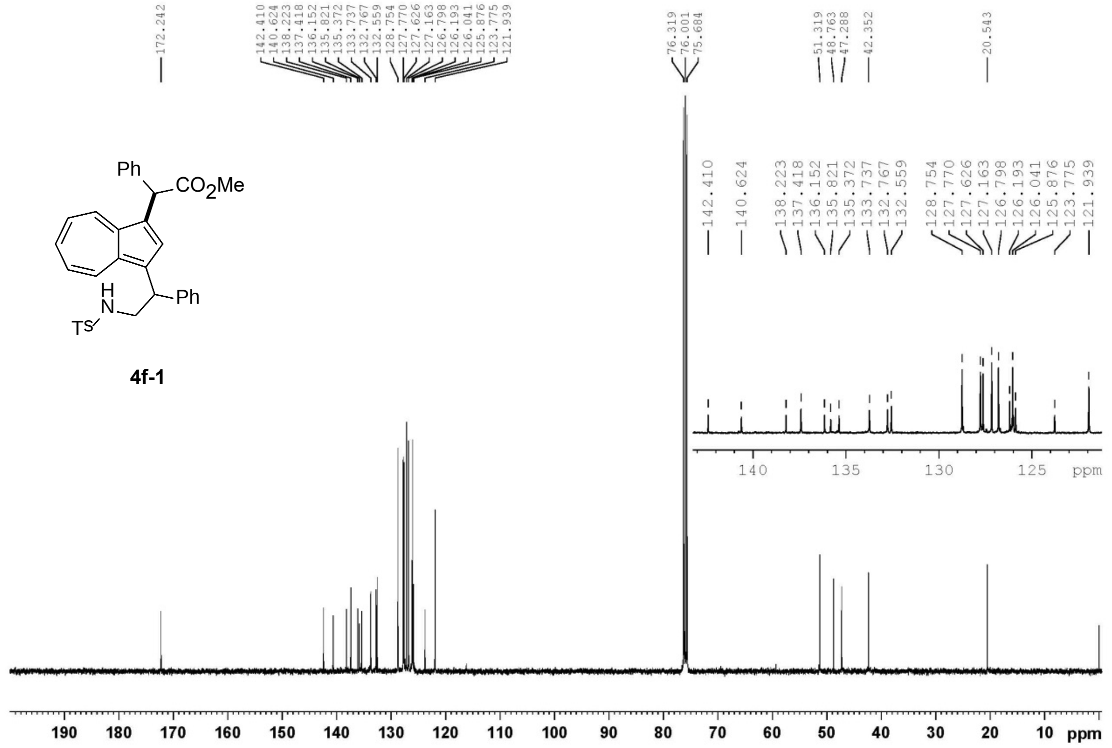

^{1}H NMR (400 MHz, $CDCl_3$)

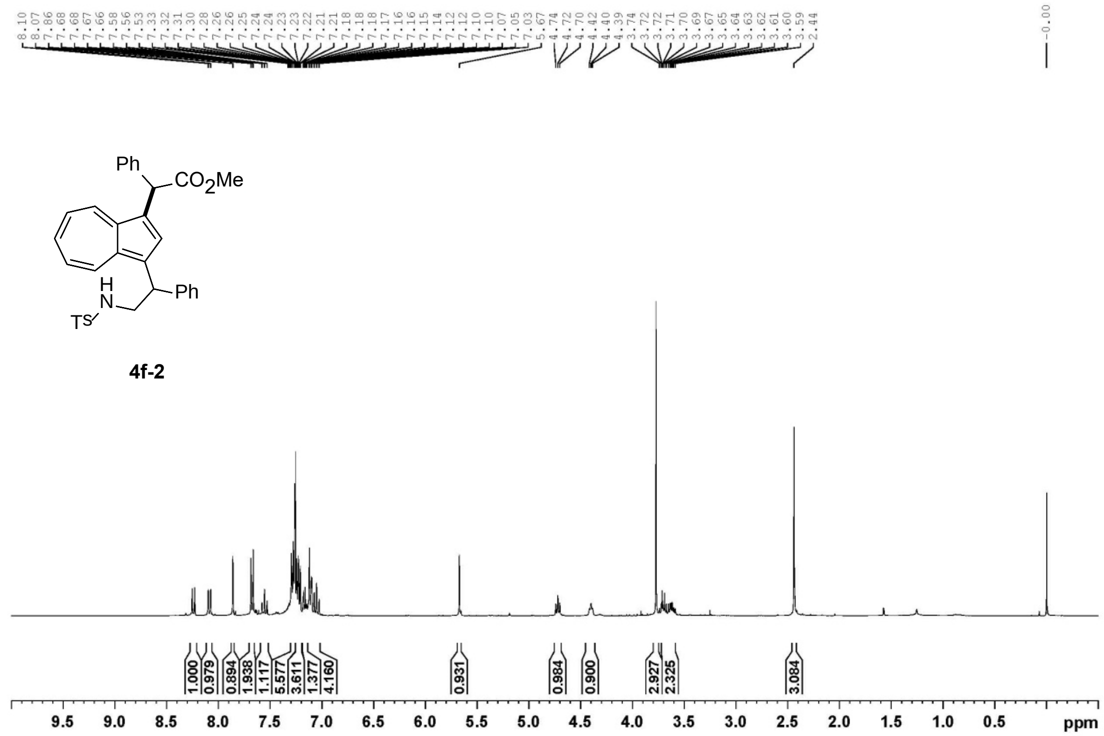

^{13}C{^{1}H} NMR (100 MHz, $CDCl_3$)

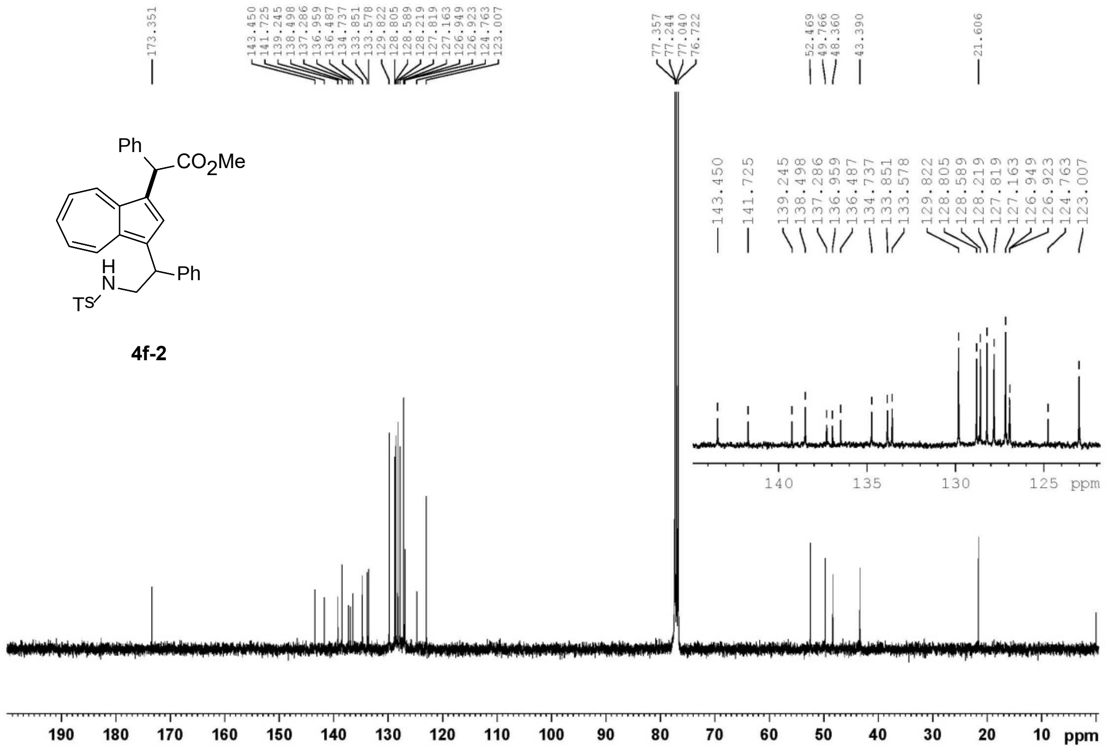

^{1}H NMR (400 MHz, $CDCl_3$)

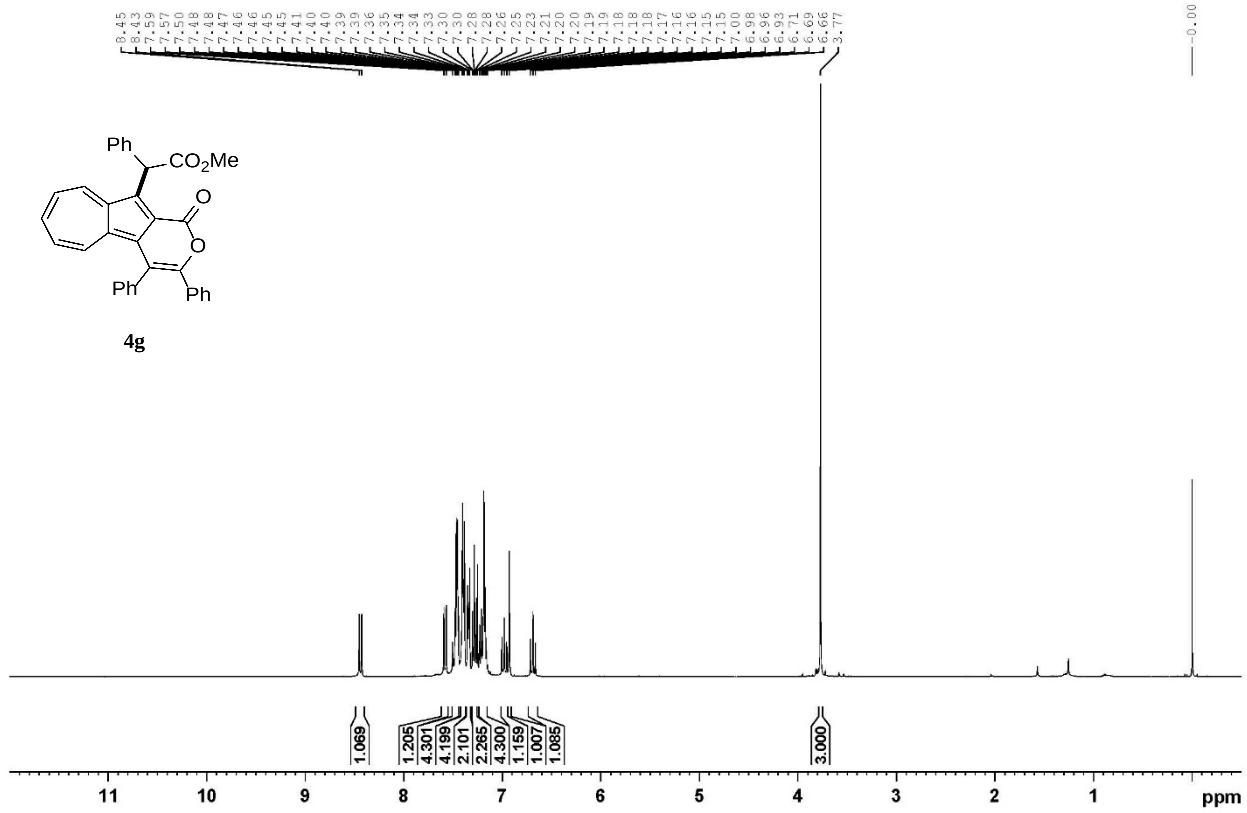

^{13}C{^{1}H} NMR (100 MHz, $CDCl_3$)

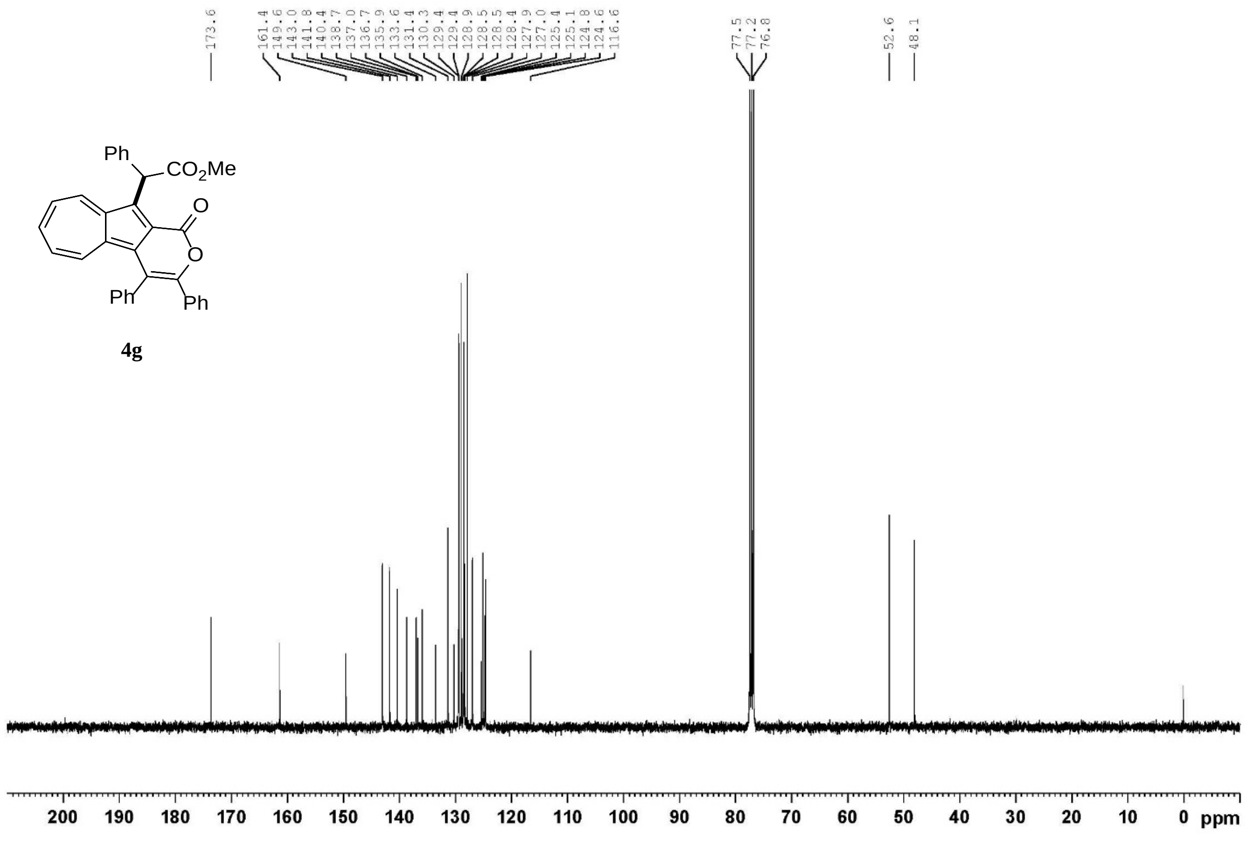

^{1}H NMR (400 MHz, CDCl$_3$)

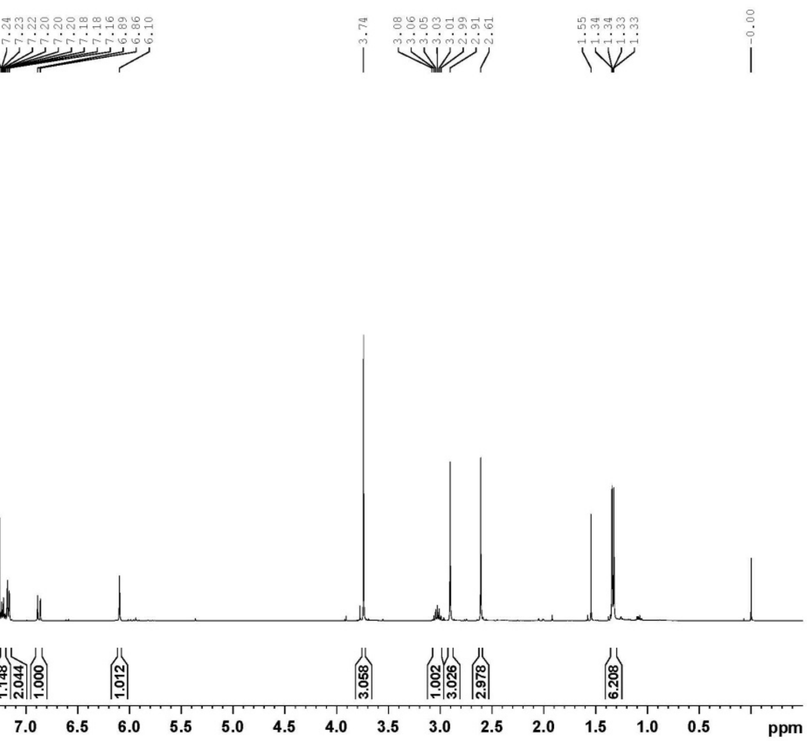

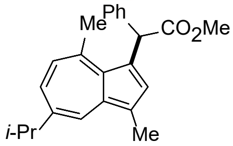

4h

^{13}C{^{1}H} NMR (100 MHz, CDCl$_3$)

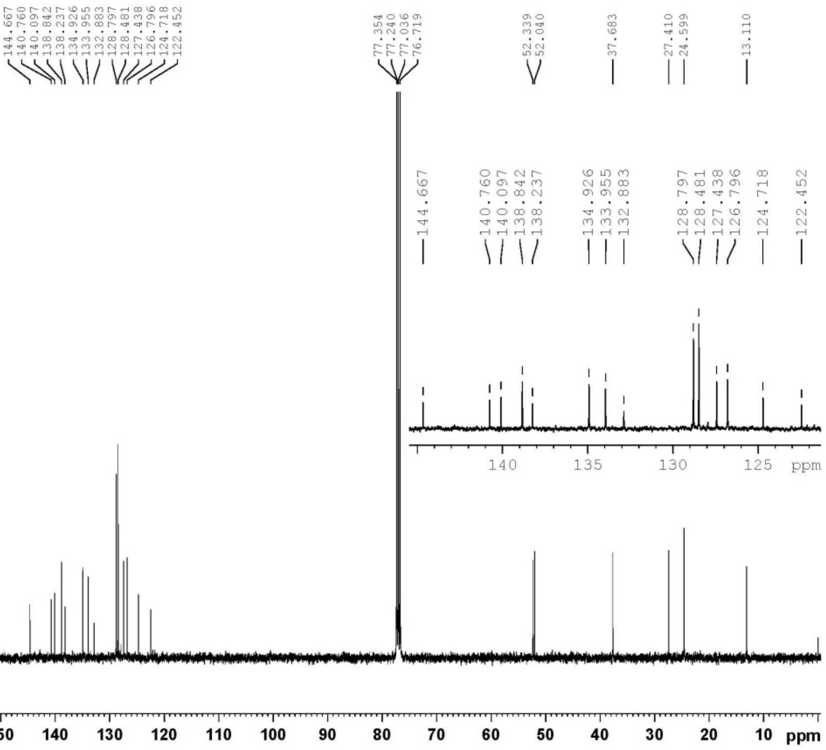

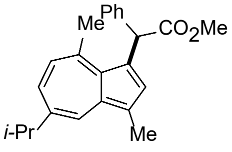

4h

^{1}H NMR (400 MHz, $CDCl_3$)

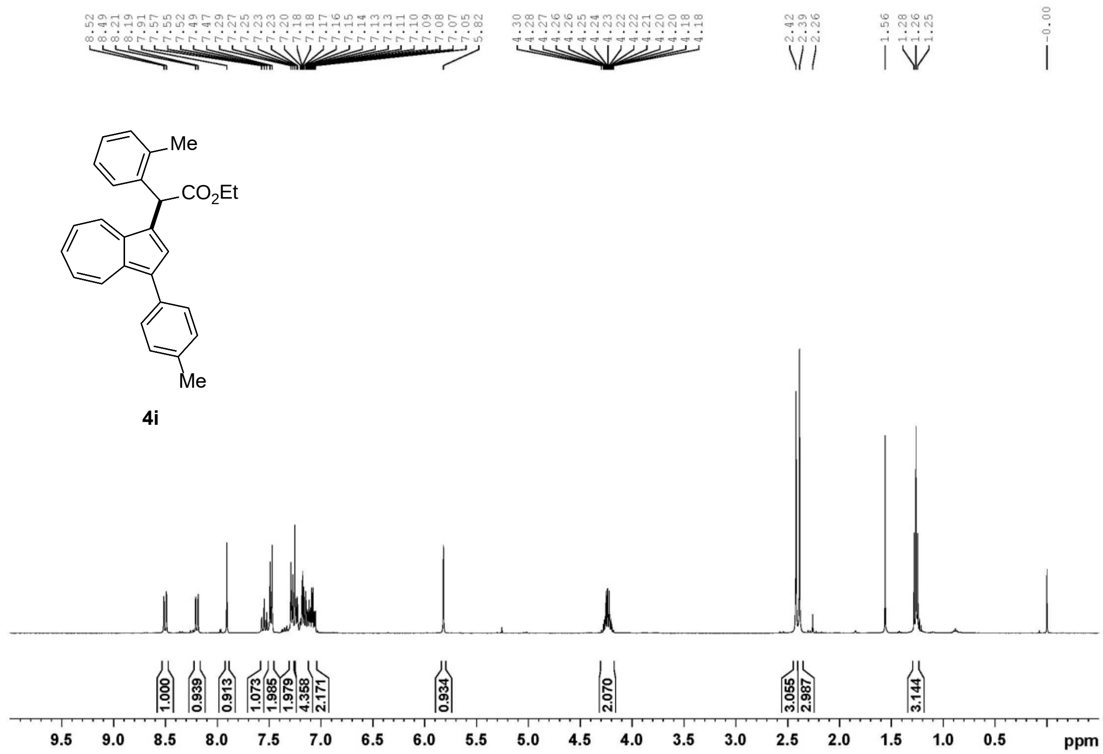

$^{13}C\{^{1}H\}$ NMR (100 MHz, $CDCl_3$)

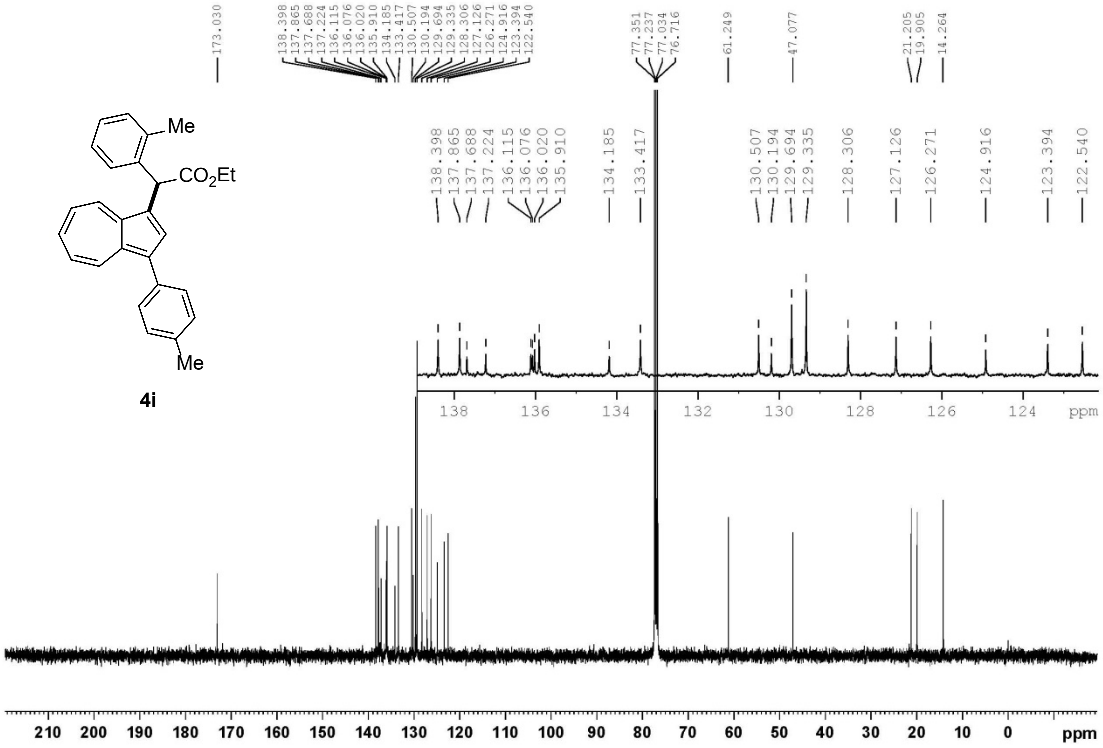

^{1}H NMR (400 MHz, $CDCl_3$)

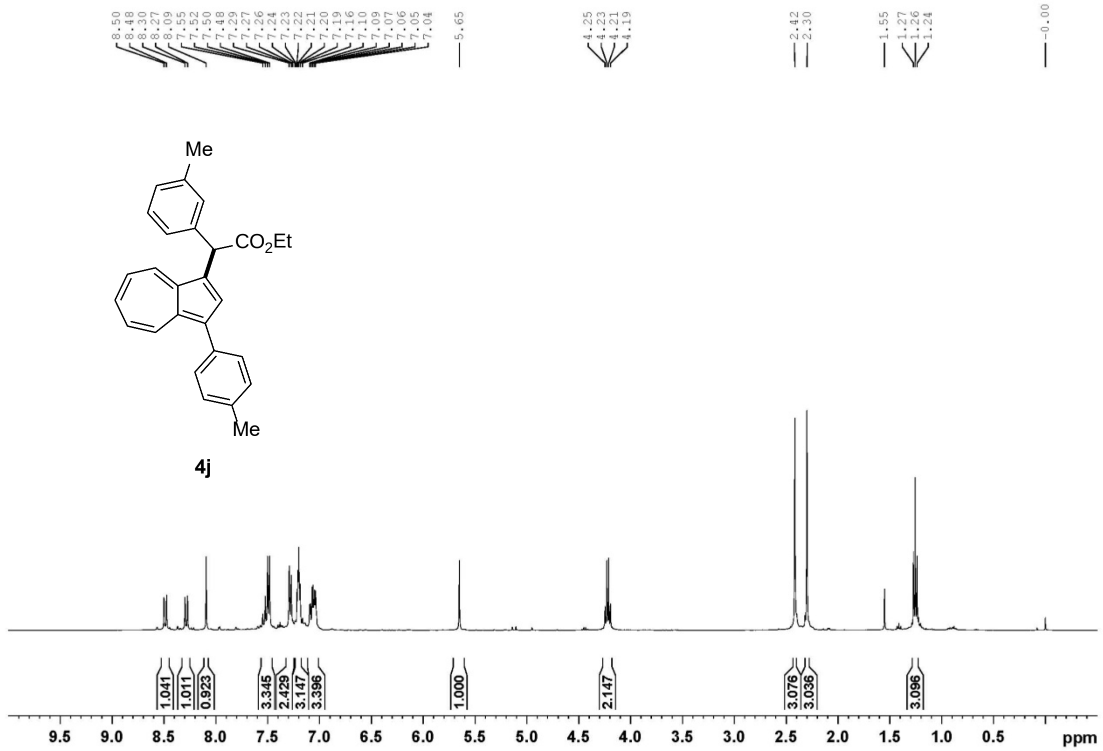

$^{13}C\{^{1}H\}$ NMR (100 MHz, $CDCl_3$)

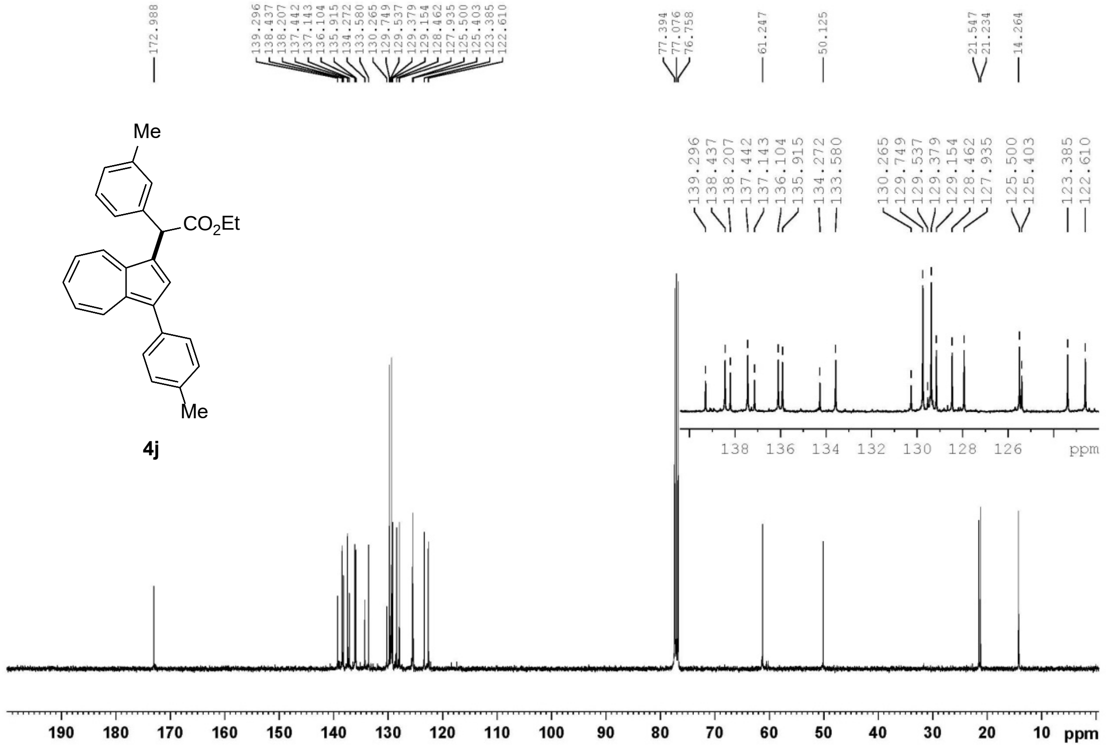

^{1}H NMR (400 MHz, $CDCl_3$)

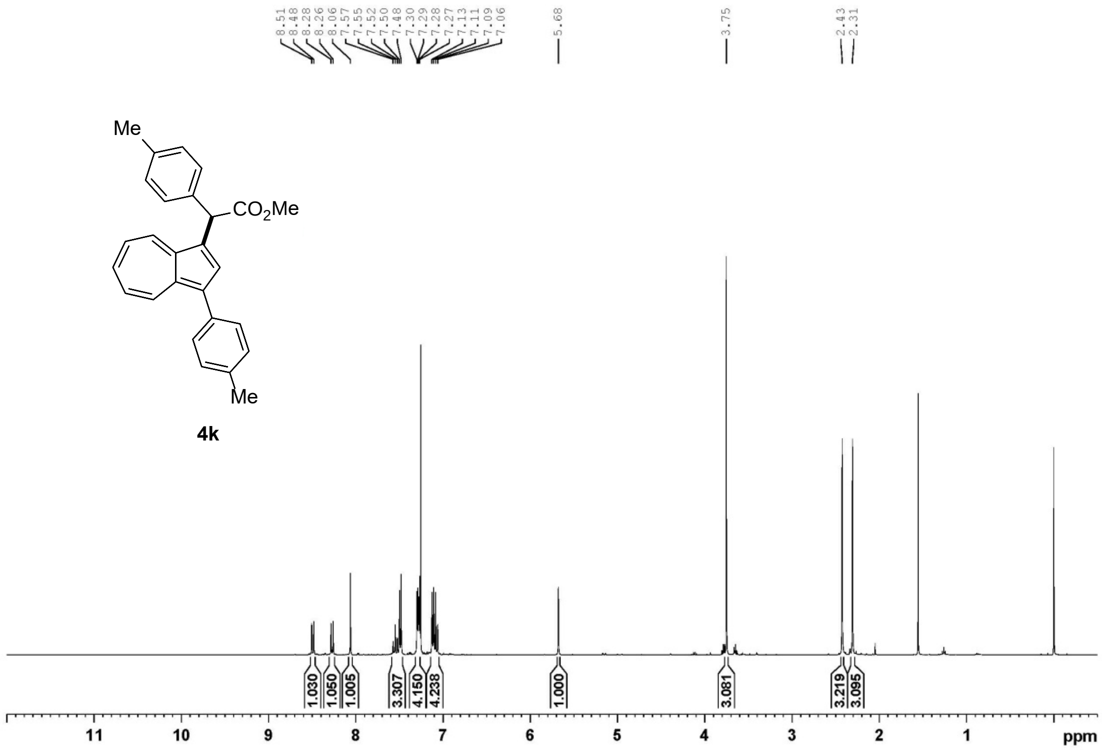

4k

^{13}C{^{1}H} NMR (100 MHz, $CDCl_3$)

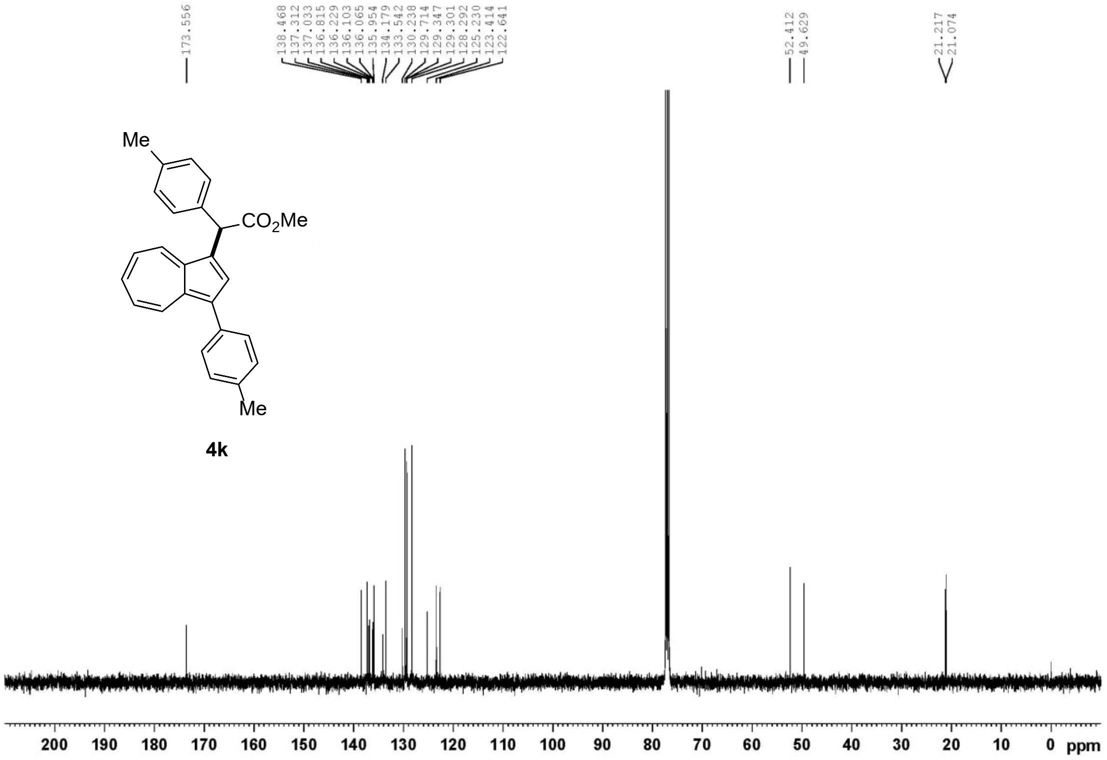

4k

^{1}H NMR (400 MHz, $CDCl_3$)

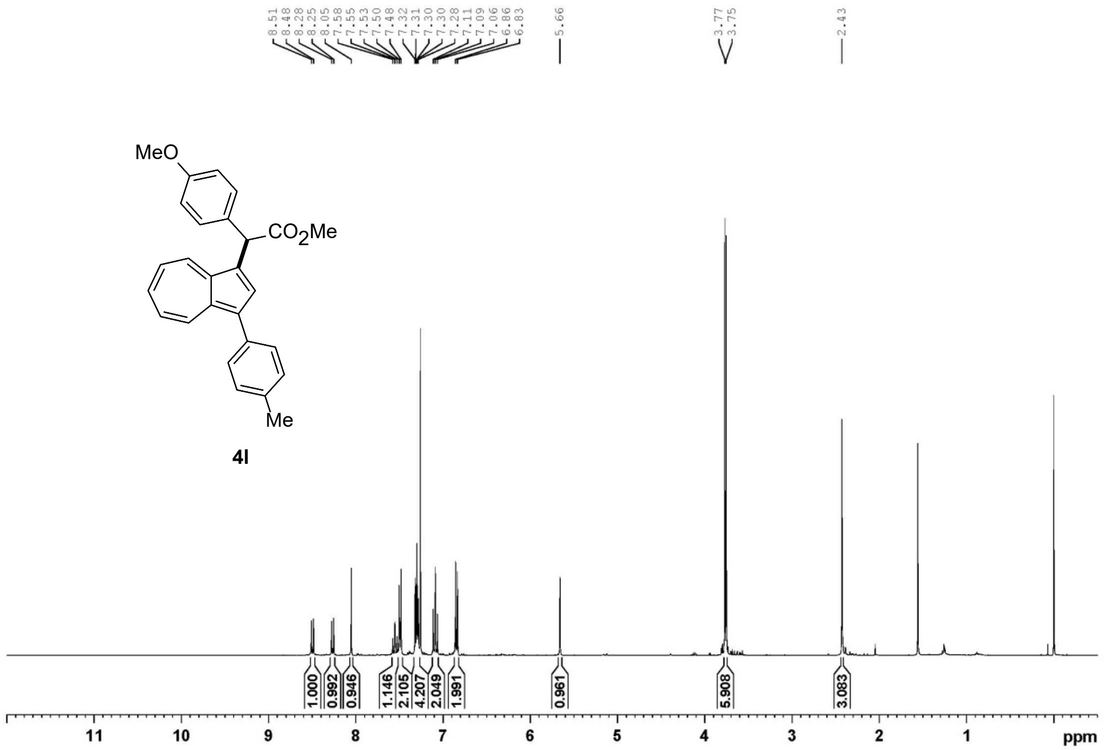

$^{13}C\{^1H\}$ NMR (100 MHz, $CDCl_3$)

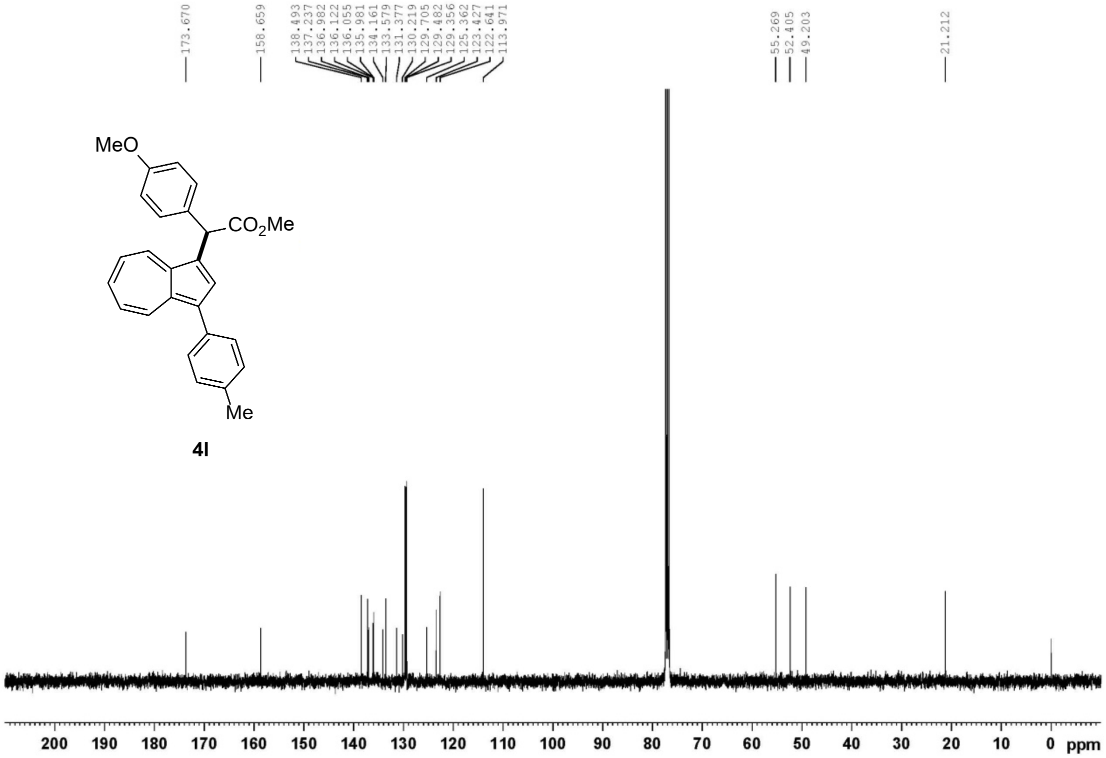

^1H NMR (400 MHz, $CDCl_3$)

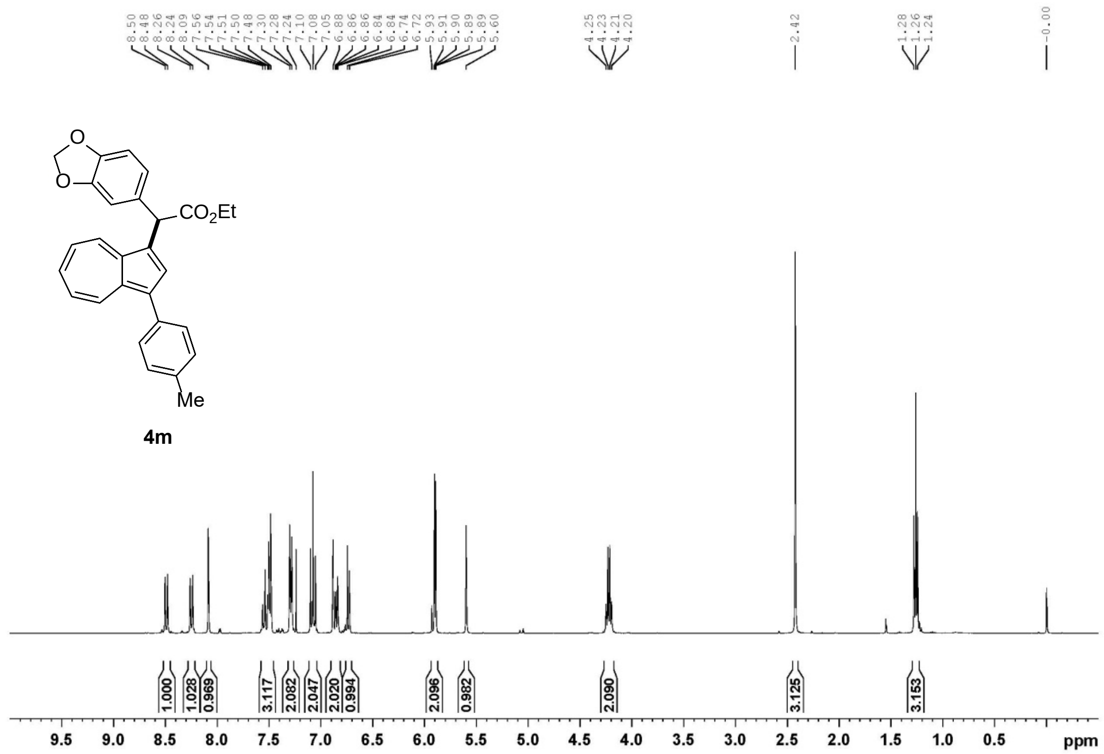

$^{13}C\{^1H\}$ NMR (100 MHz, $CDCl_3$)

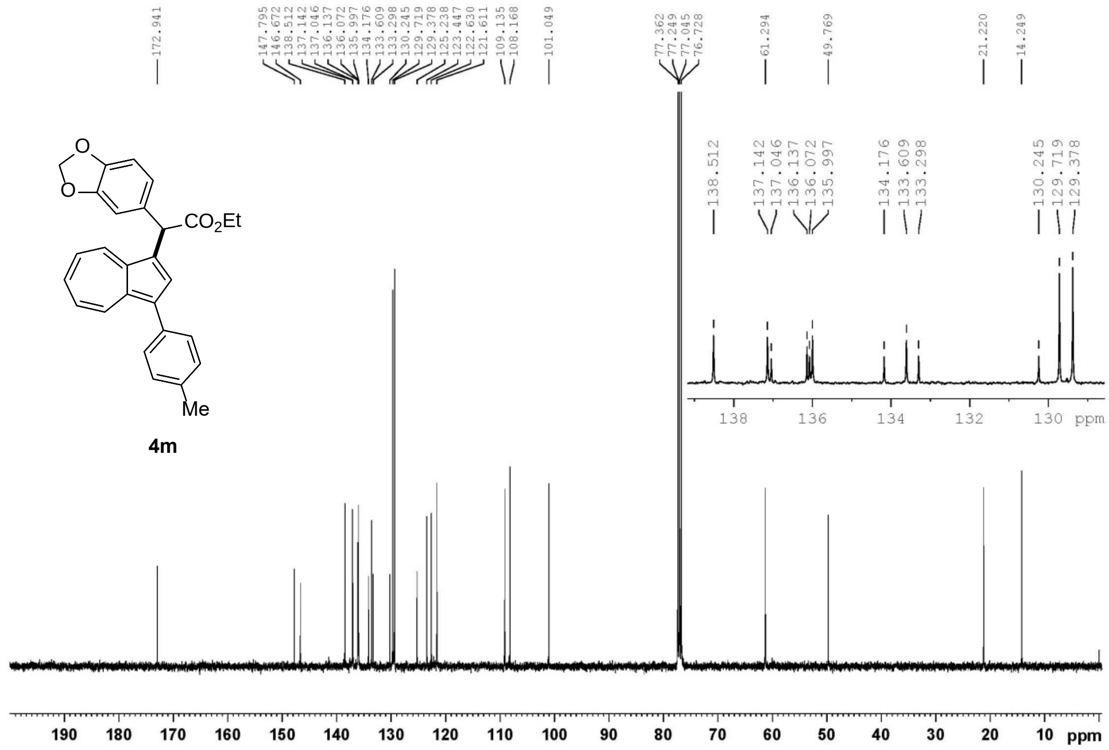

^{1}H NMR (400 MHz, $CDCl_3$)

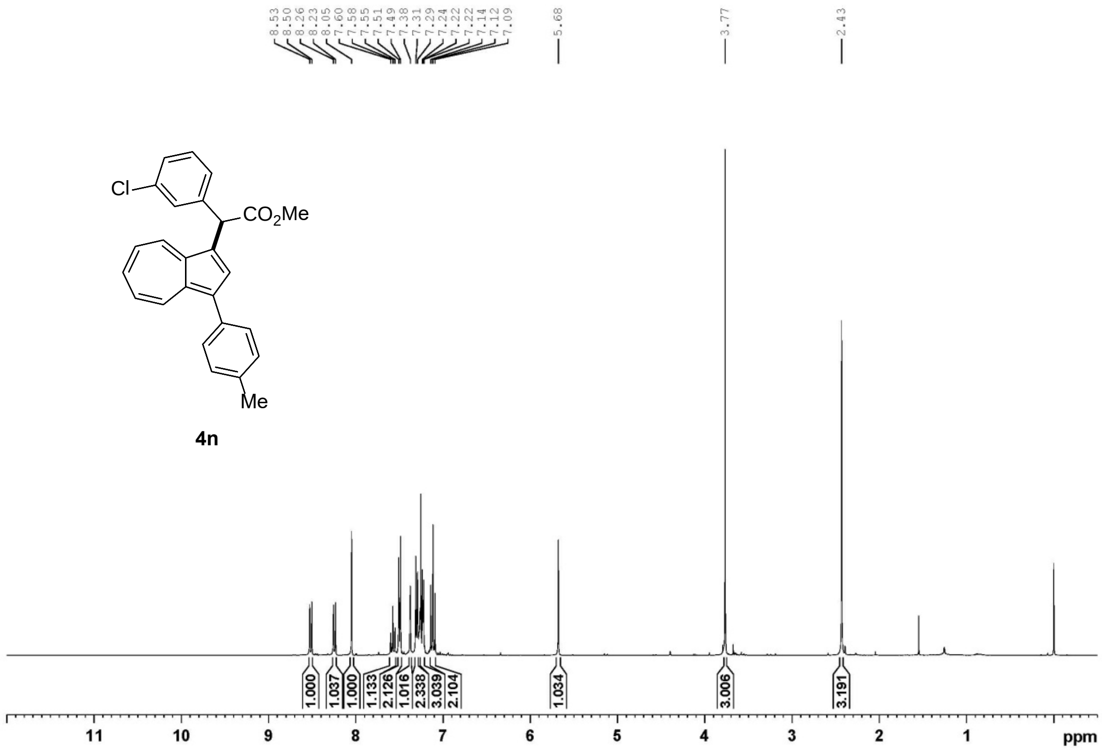

^{13}C{^{1}H} NMR (100 MHz, $CDCl_3$)

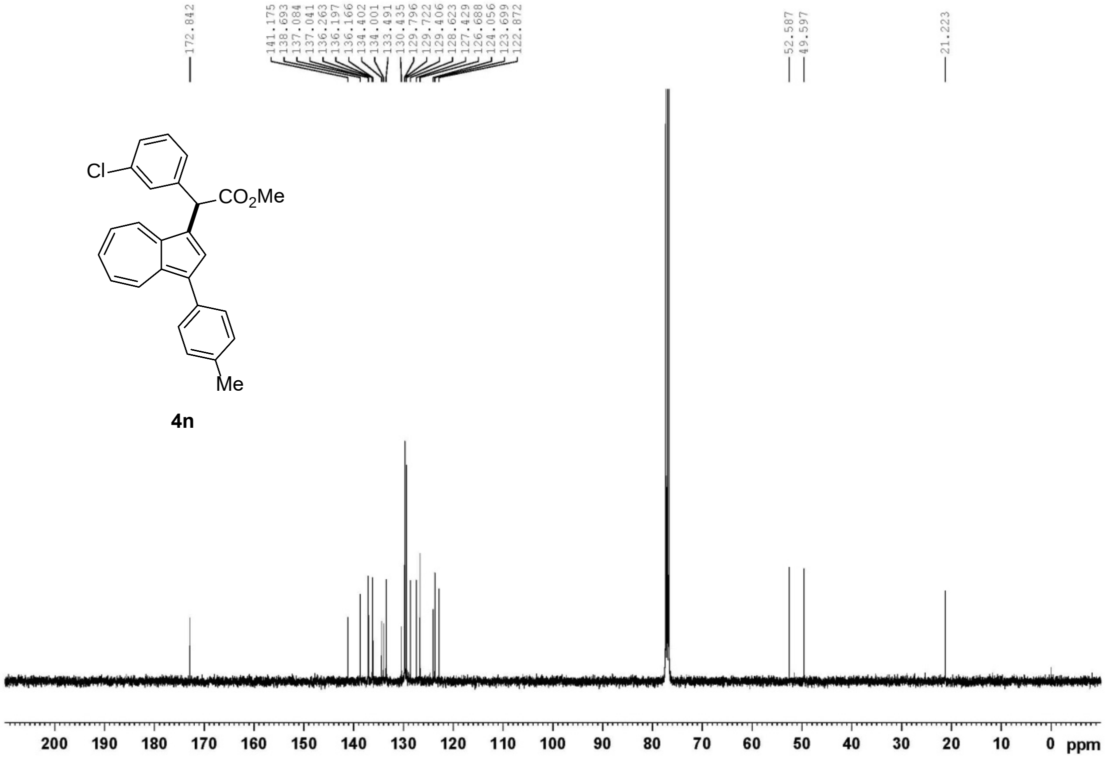

^{1}H NMR (400 MHz, $CDCl_3$)

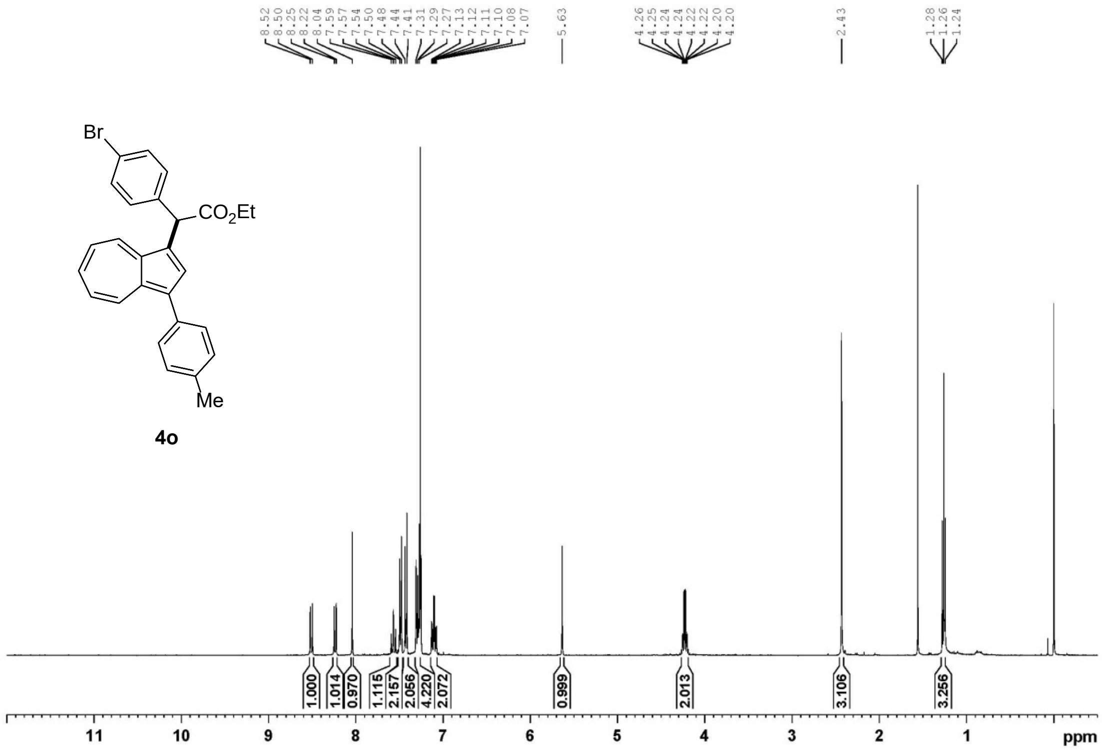

$^{13}C\{^{1}H\}$ NMR (100 MHz, $CDCl_3$)

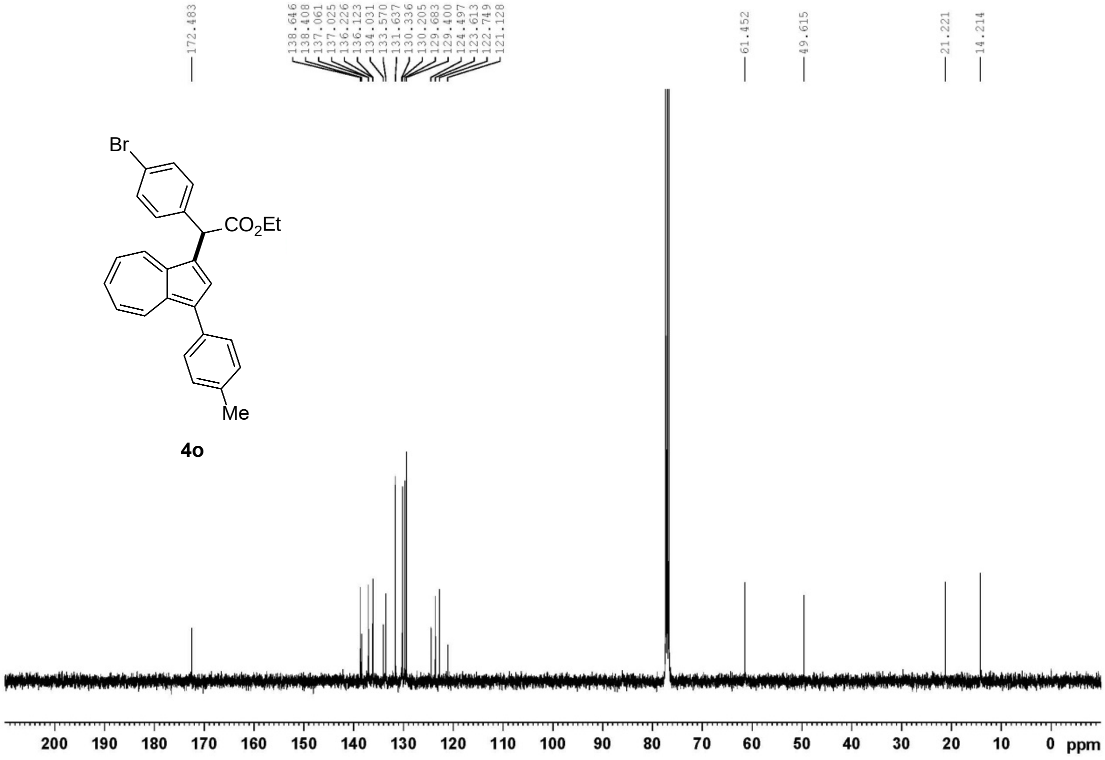

^{1}H NMR (400 MHz, $CDCl_3$)

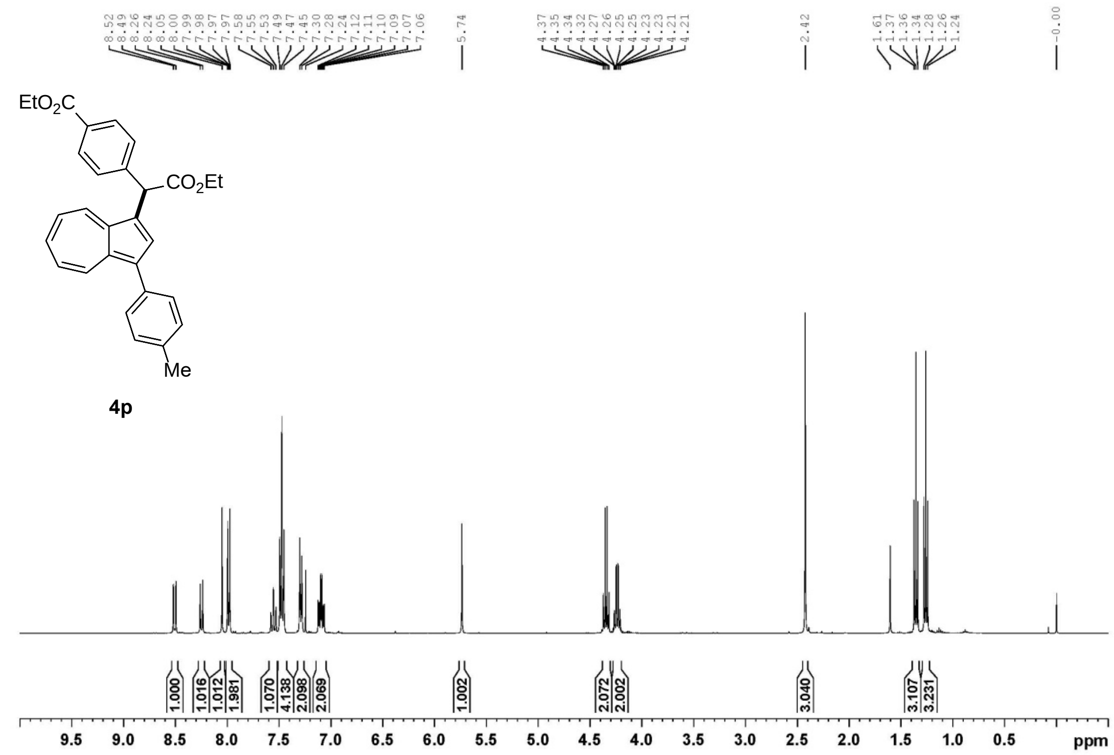

$^{13}C\{^1H\}$ NMR (100 MHz, $CDCl_3$)

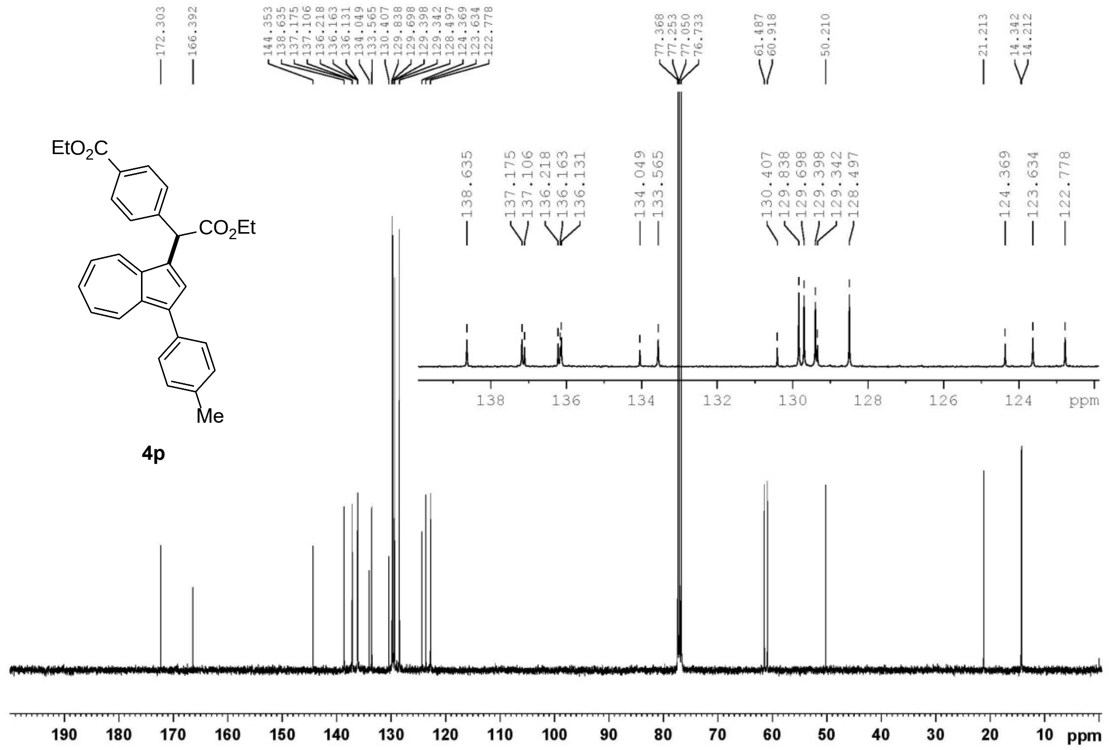

^1H NMR (400 MHz, $CDCl_3$)

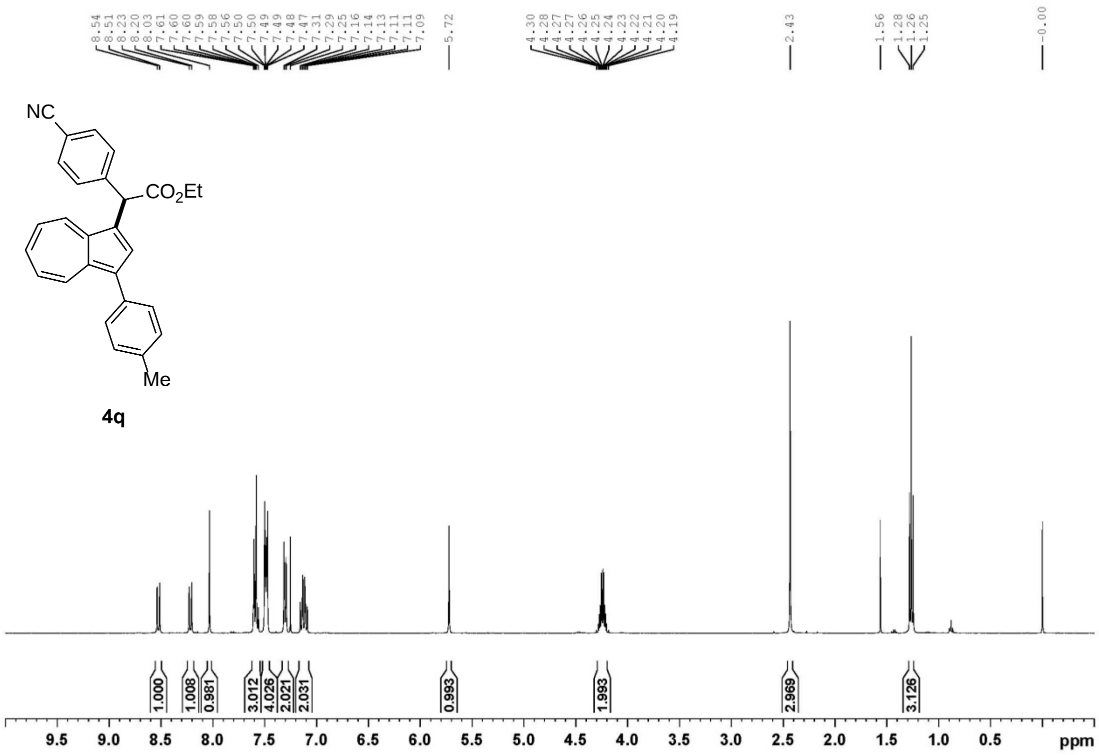

$^{13}C\{^1H\}$ NMR (100 MHz, $CDCl_3$)

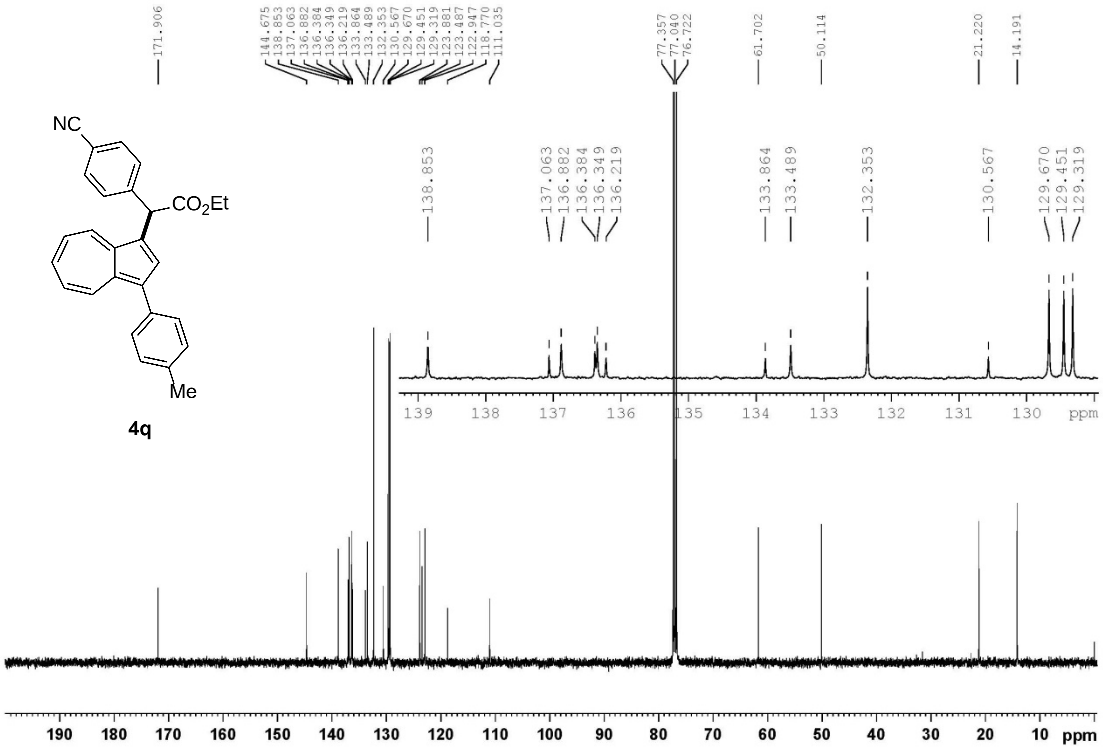

^{1}H NMR (400 MHz, $CDCl_3$)

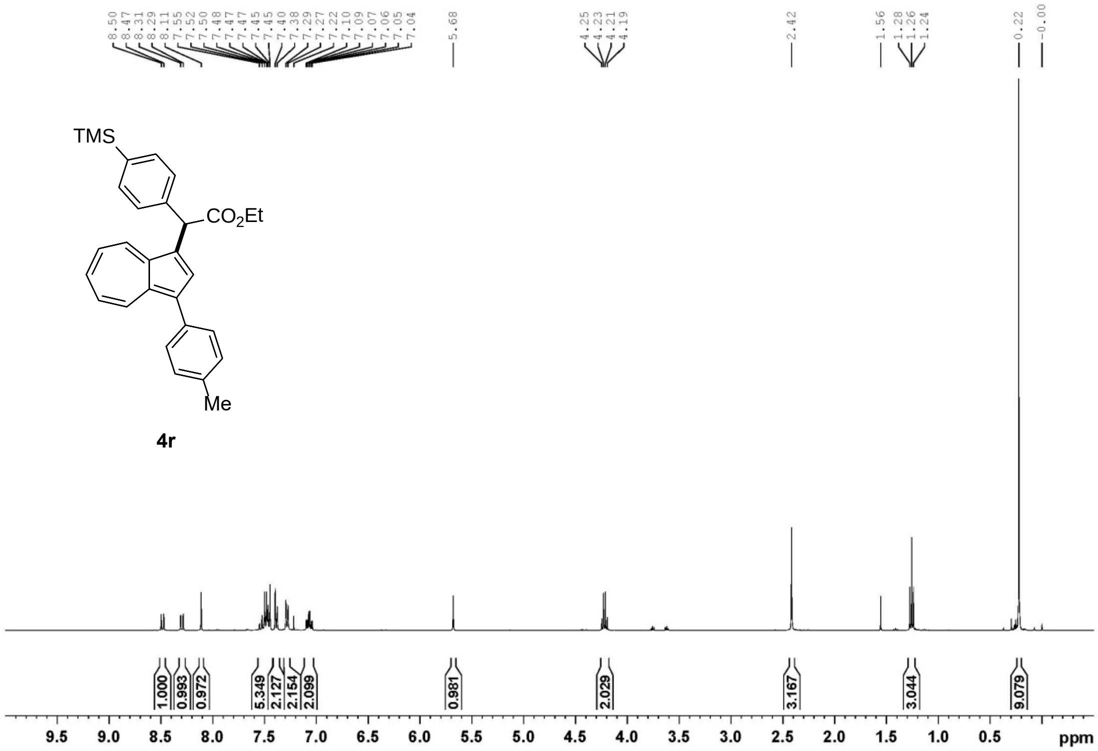

$^{13}C\{^1H\}$ NMR (100 MHz, $CDCl_3$)

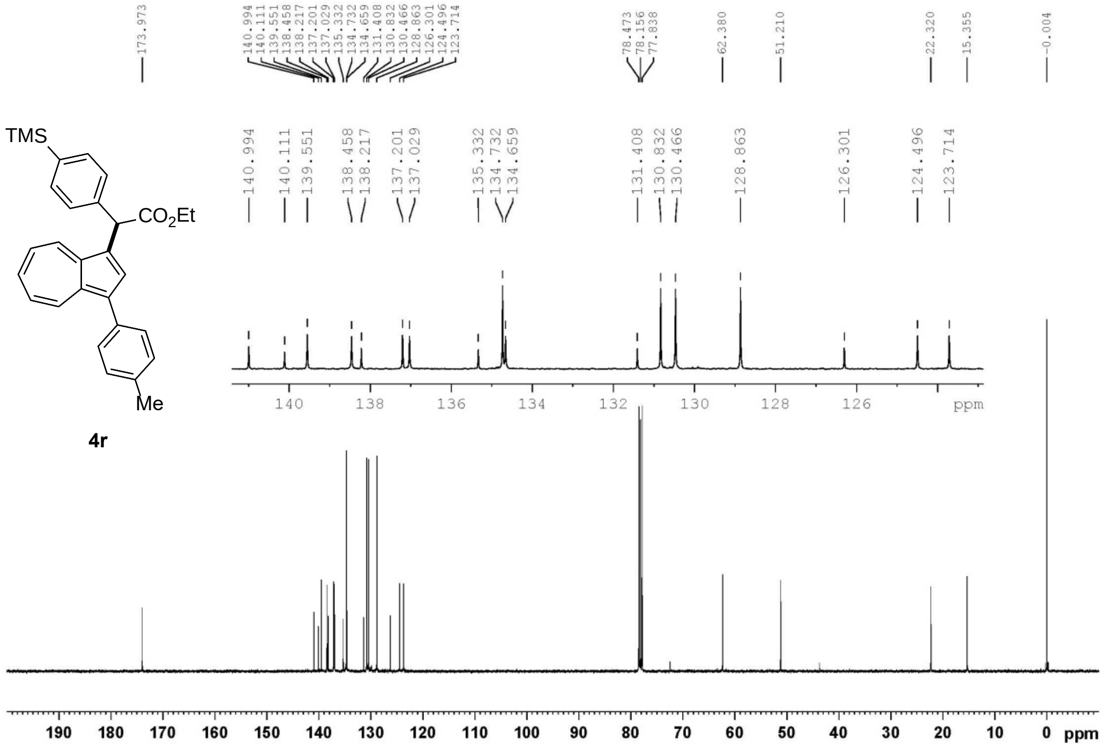

^{1}H NMR (400 MHz, $CDCl_3$)

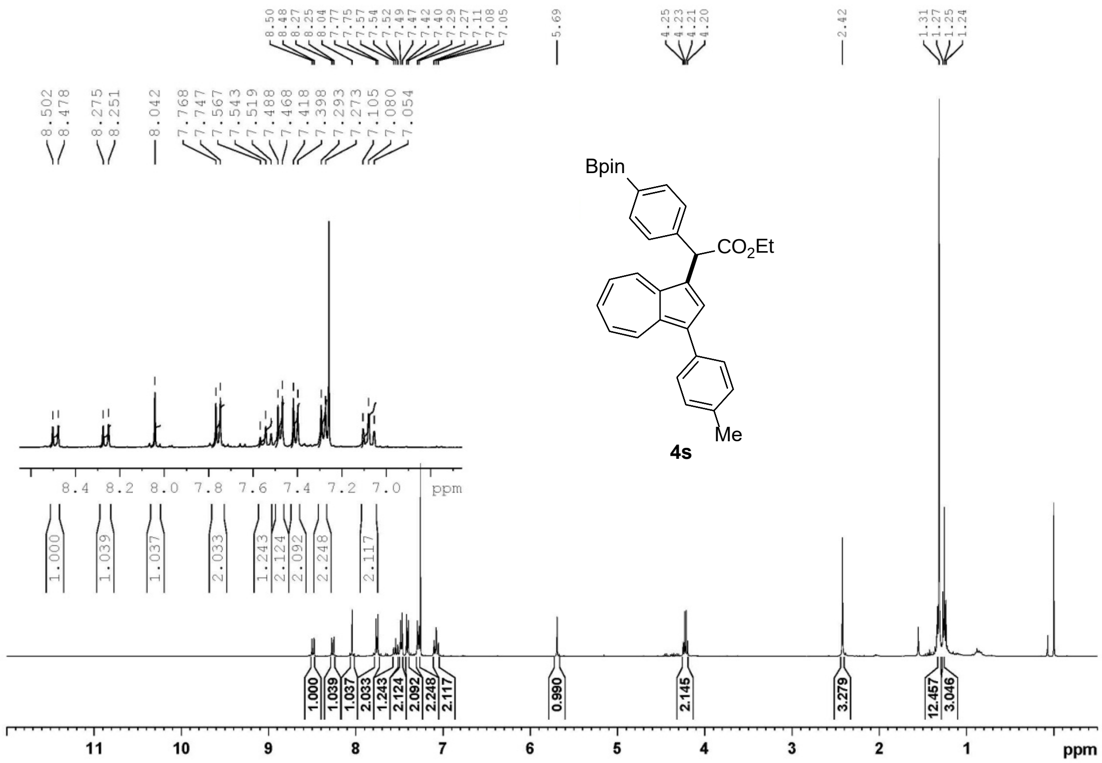

$^{13}C\{^1H\}$ NMR (100 MHz, $CDCl_3$)

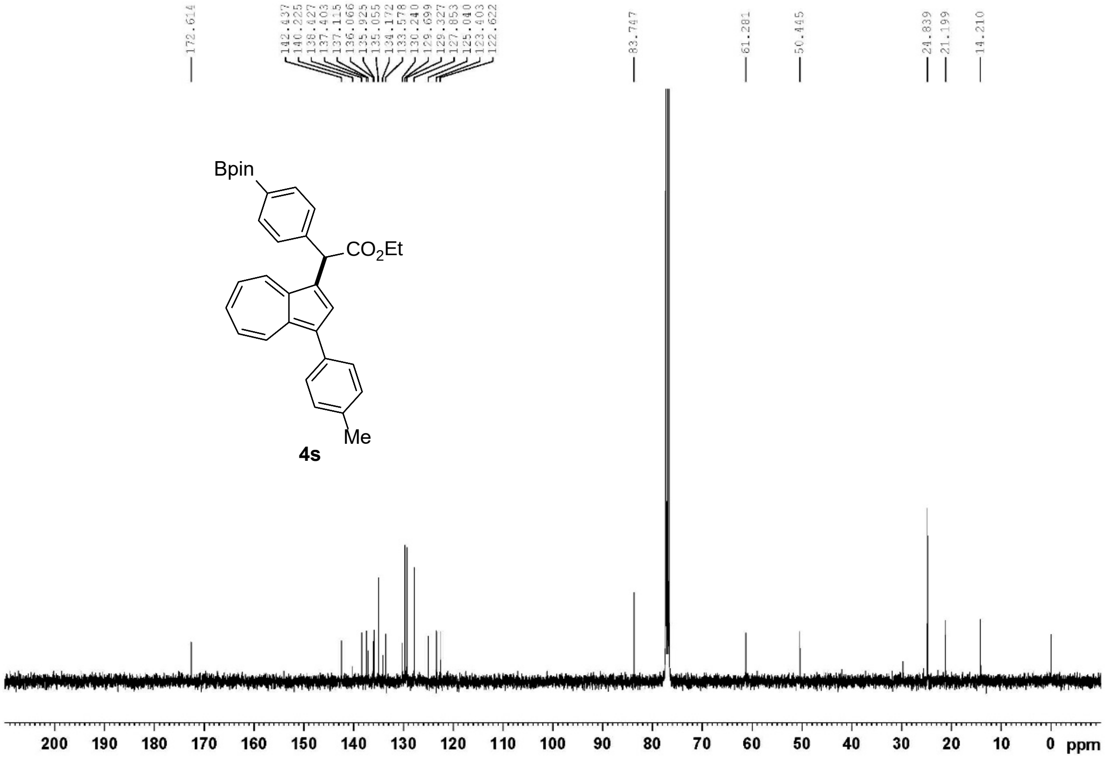

^{1}H NMR (400 MHz, $CDCl_3$)

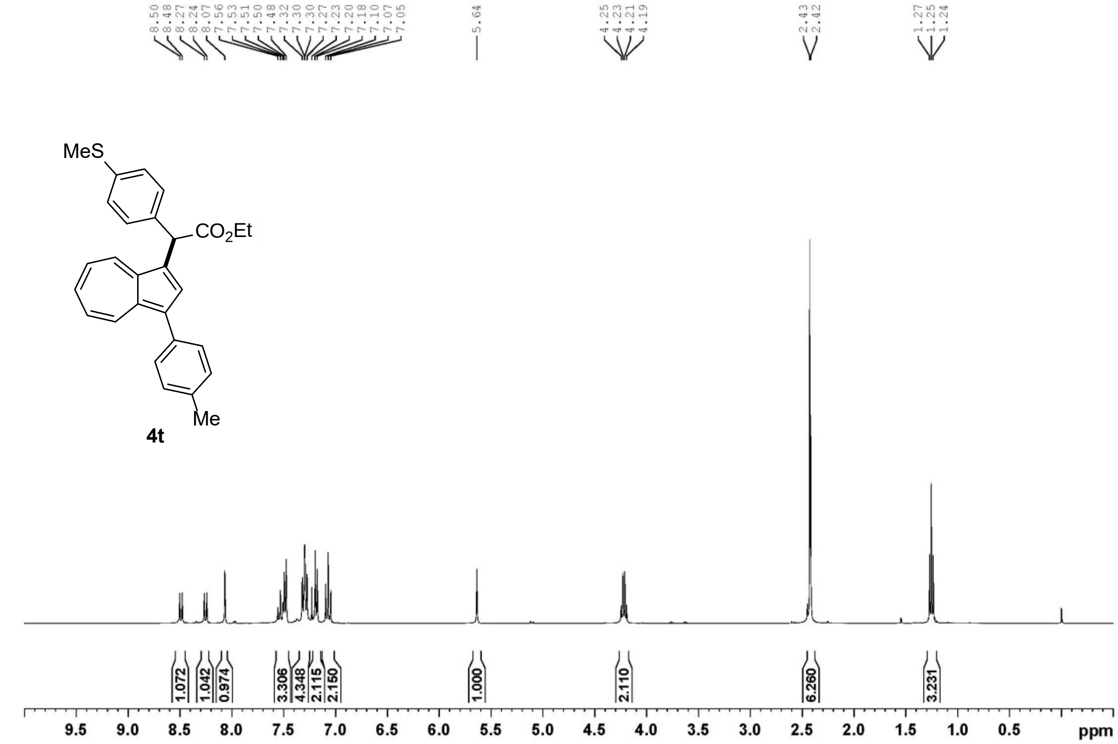

$^{13}C\{^{1}H\}$ NMR (100 MHz, $CDCl_3$)

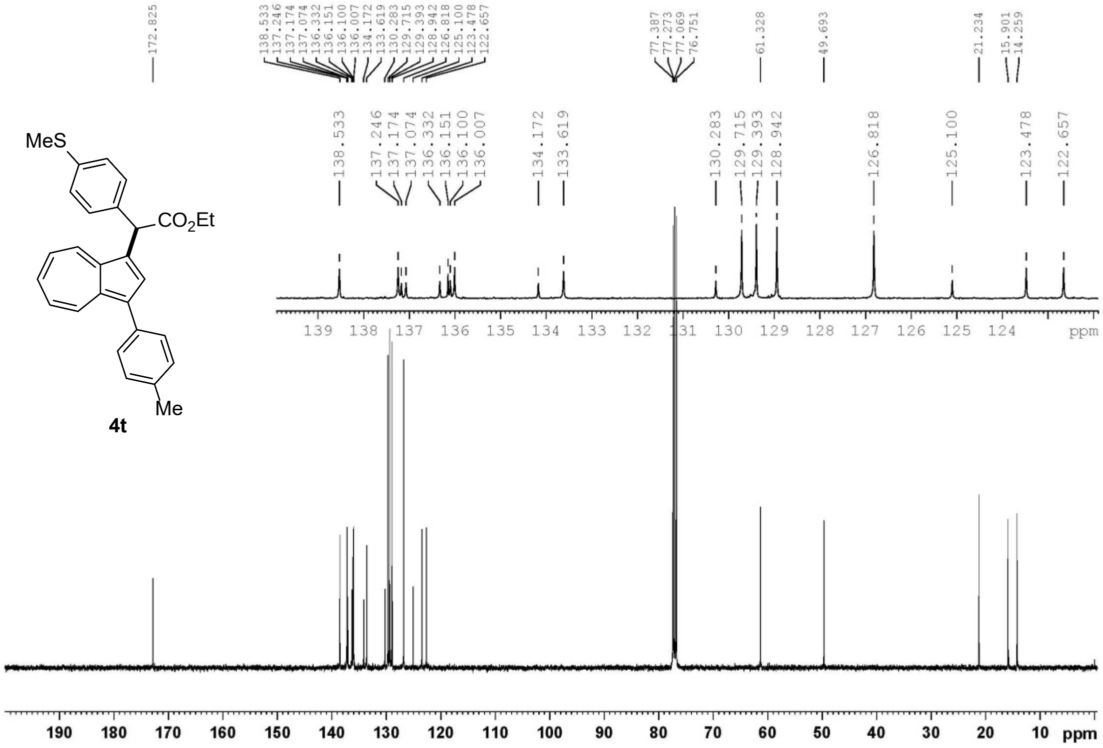

^{1}H NMR (400 MHz, $CDCl_3$)

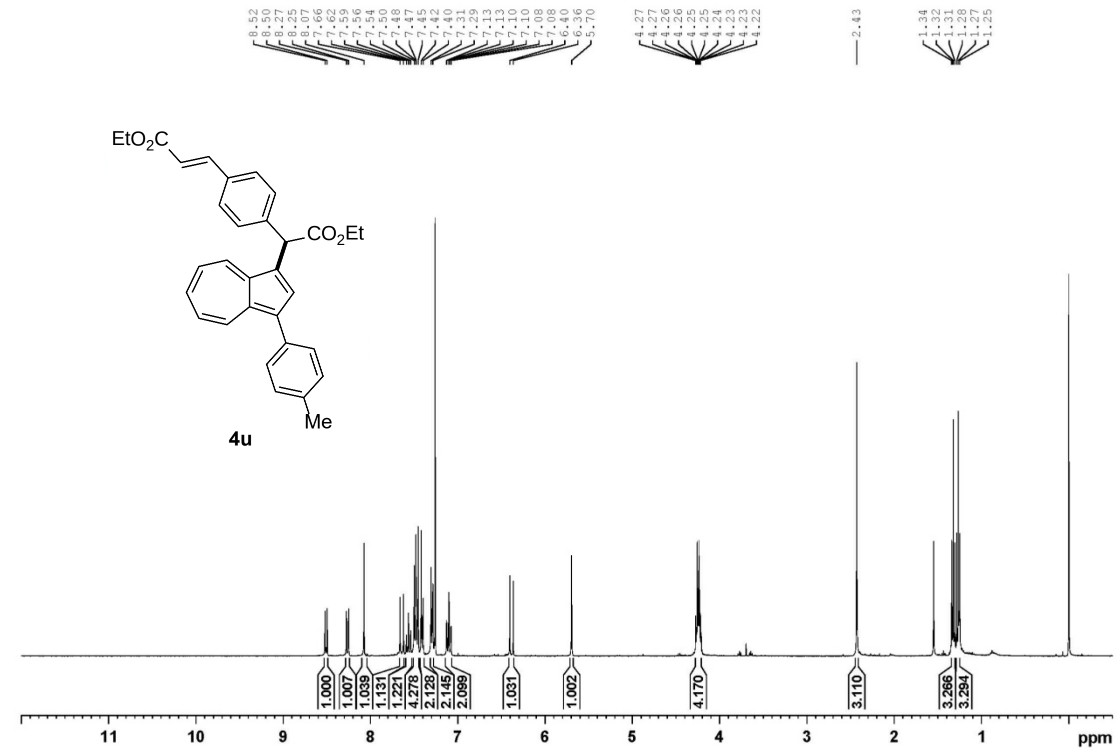

$^{13}C\{^{1}H\}$ NMR (100 MHz, $CDCl_3$)

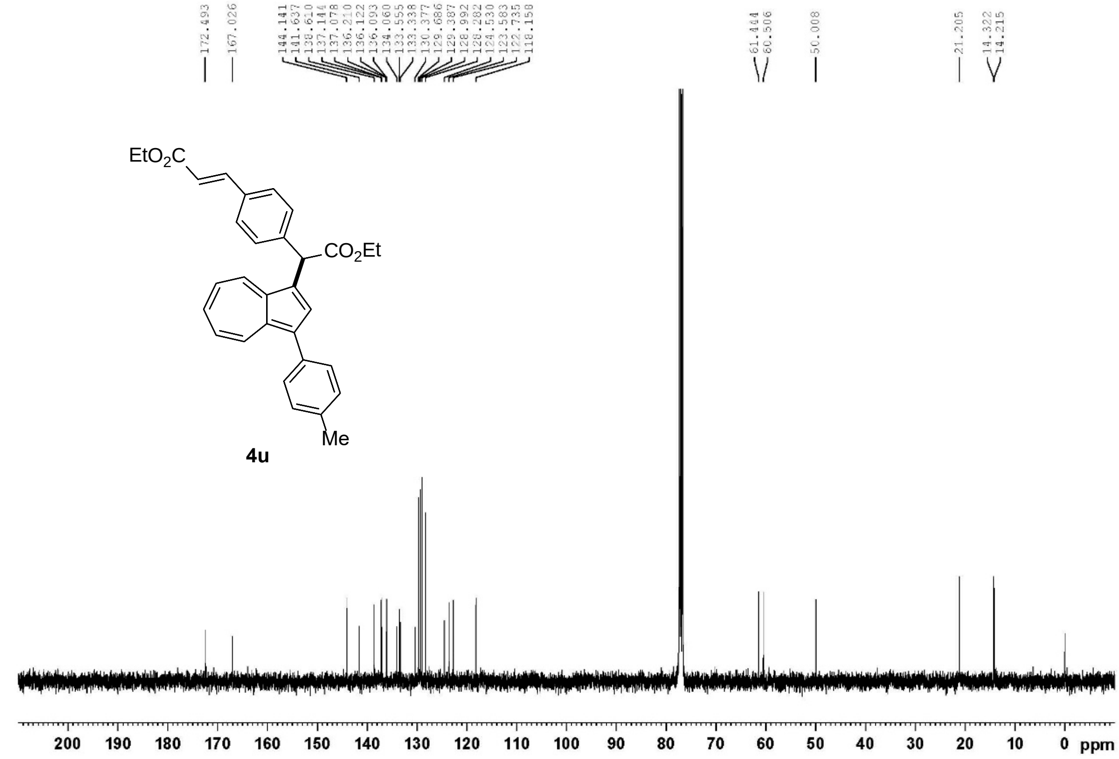

^{1}H NMR (400 MHz, $CDCl_3$)

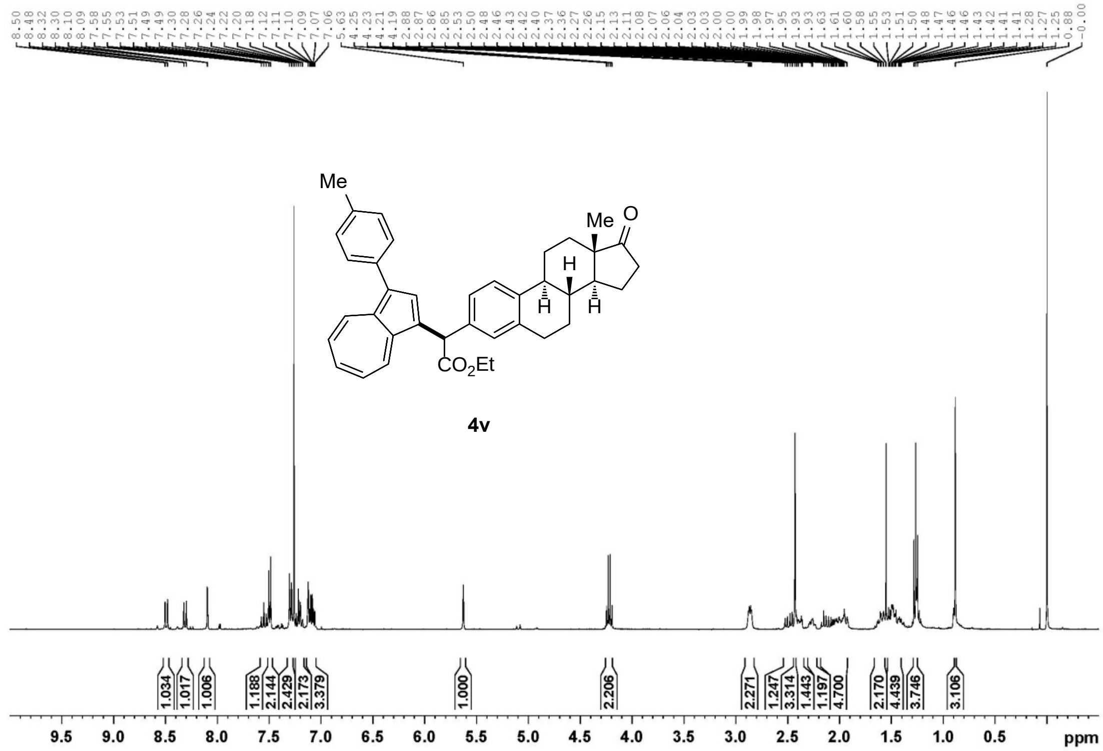

$^{13}C\{^{1}H\}$ NMR (100 MHz, $CDCl_3$)

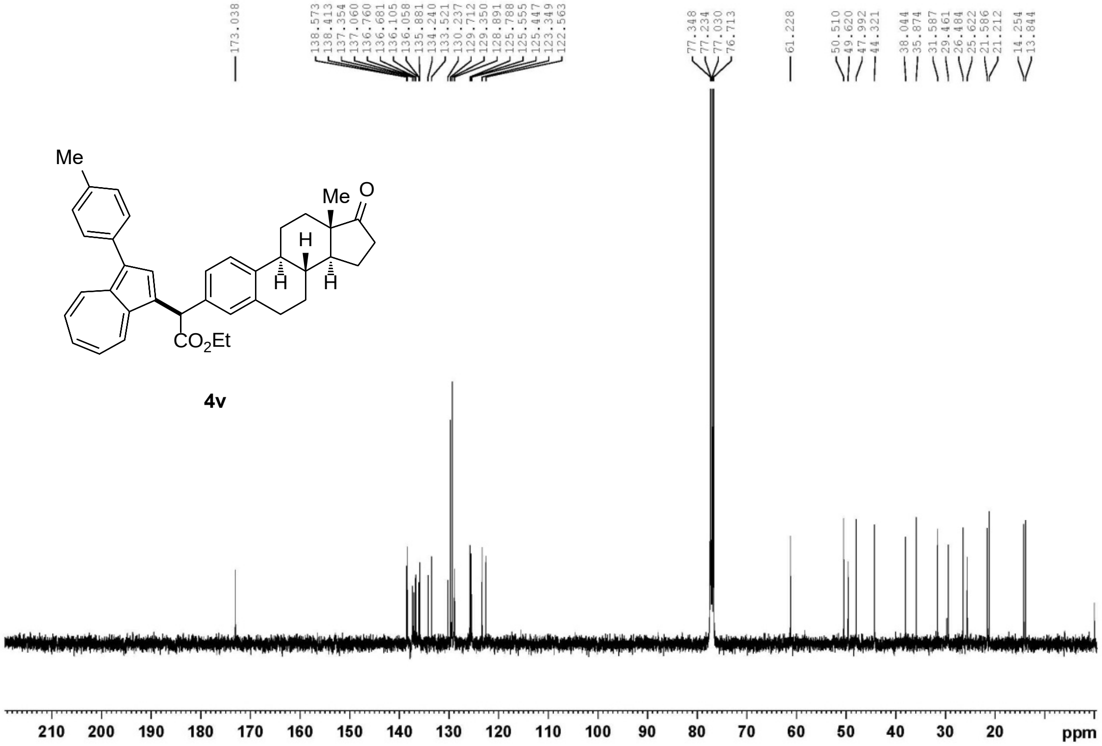

Supplement: Supplementary file 1 — Supplementary Information [file 41467_2023_43200_MOESM1_ESM.pdf]
